# An *H*‑Phosphonate-Mediated Synthesis of Nucleotide-Pyranose Glycoconjugates

**DOI:** 10.1021/acs.orglett.6c02135

**Published:** 2026-06-10

**Authors:** Thibault Guillaume, Ningwu Huang, Mark Smith, Gavin J. Miller

**Affiliations:** † School of Chemical and Physical Sciences, 4212 Keele University, Keele, Staffordshire ST5 5BG, U.K.; ‡ Riboscience LLC, 3160 Porter Drive, Palo Alto, California 94158, United States; § Manchester Institute of Biotechnology & Department of Chemistry, 5292 University of Manchester, Manchester, M1 7DN, U.K.

## Abstract

We explore a synthesis of nucleotide-pyranose conjugates, successfully developing an *H*-phosphonate-mediated coupling of pyranoses with nucleosides that favors making the reactive phosphorus­(III) species within the pyranose (C6-OH or C1-OH) and affords, after oxidation to the free phosphate, glycoconjugates in good overall yields (65–84%). Following deprotection and final purification, the materials are evaluated in U87-MG and PANC-1 cells, with activities comparable to the parent drugs, indicating such conjugates worthy of deeper validation as prodrugs.

## Graphical abstract

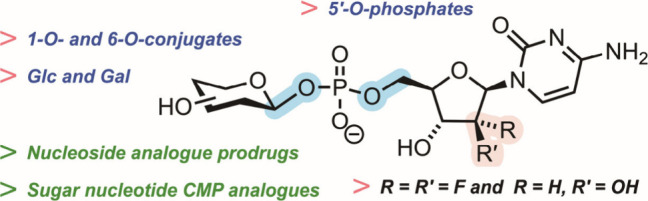

Synthetic analogues of canonical nucleosides are an established strategy for therapeutics targeting diverse biological processes, including DNA replication, transcription, translation and cell signaling.^1–5^ Nucleoside and nucleotide analogue therapeutics may contain modifications to the D-ribose sugar, heterobase or 5′-*O*-phosphate that confer favorable pharmacological profiles. One important innovation, pioneered by McGuigan, is the masked 5′-*O*-phosphate phosphoramidate.^6,7^ These prodrugs improve membrane permeability and can overcome the often limiting first intracellular phosphorylation step. This approach has been transformative in drugs such as sofosbuvir and remdesivir (Figure 1a), and has also been extended by Serpi and colleagues to masked nucleotide triphosphates.^8^

**Figure 1:**
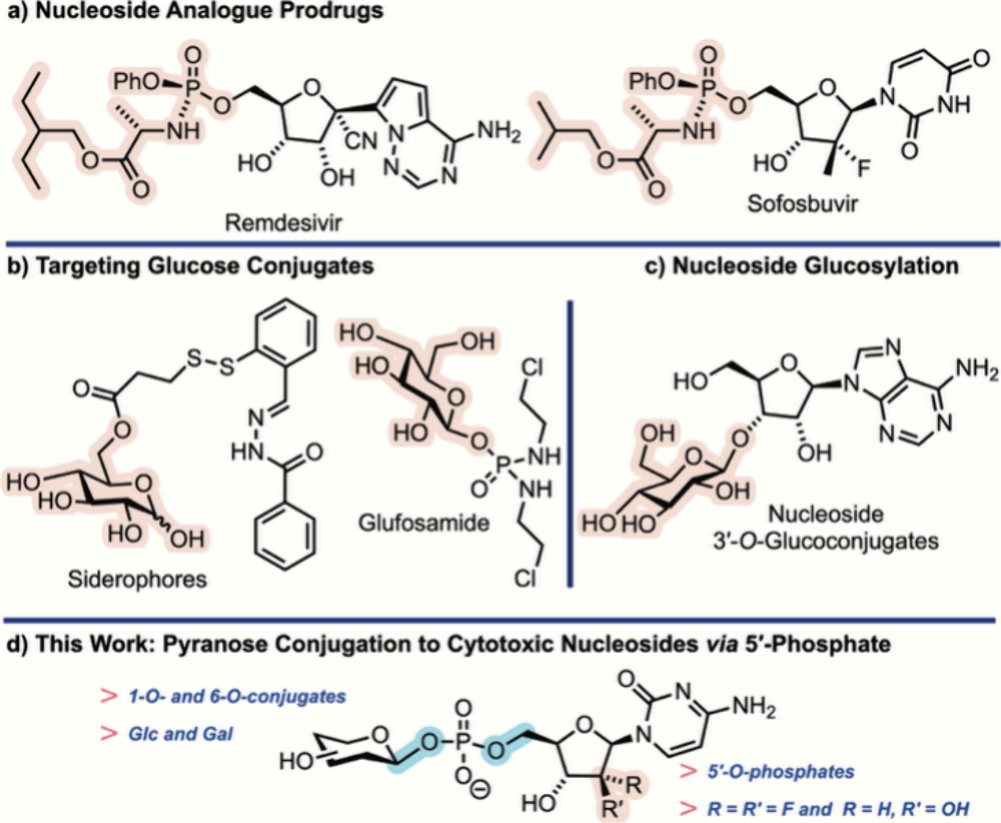
a) Structures of nucleoside analogue prodrugs. b) Examples of glucoconjugates for a siderophore and a mustard agent. c) Example of 3′-*O*-glucosylated adenosine, accessed *via* enzymatic glycosylation. d) Work herein to assemble monophosphate glycoconjugates of the cytotoxic nucleoside analogues.

Recently, we became interested in novel nucleoside architectures and alternative prodrug strategies for therapeutic delivery.^9–12^ We focused on glucose transport as some tumors overexpress this pathway in the cancer microenvironment, and glucose conjugation may also improve solubility. Glycoconjugation has been explored for other cytotoxic agents, including arylhydrazone prochelators, DNA alkylators, platinum-based drugs and the glucose-modified mustard Glufosfamide, often with favorable therapeutic effects (Figure 1b).^13–16^ We have previously used chemical and enzymatic methods to conjugate glucose to nucleoside analogue therapeutics (Figure 1c),^17,18^ and here extend this to cytotoxic nucleoside monophosphates. This enables direct comparison of the biological activity of each nucleotide glycoconjugate with its parent nucleoside analogue. Accordingly, we develop herein an *H*-phosphonate-mediated coupling to introduce a bridging phosphate through either the nucleoside or pyranose component of the final glycoconjugate, followed by an initial *in vitro* biological evaluation (Figure 1d).

Previously, Ferrero, Gotor and colleagues introduced a strategy toward glucose conjugated nucleotides using a phosphoramidite reagent to access biologically active nucleoside glycoconjugates through the 6-position of glucose.^19^ Here we chose to explore addition of an *H*-phosphonate to a cytotoxic nucleoside analogue (gemcitabine or cytarabine, Scheme 1), which could then undergo coupling of an appropriately protected pyranoside, followed by oxidation to the 5′-phosphodiester. Relatedly, such chemistry has been explored in synthesizing phosphodiester linkages in glycosyl phosphosaccharides.^20^ Accordingly, our synthesis started from protected gemcitabine and arabinocytidine 5′-position alcohols **1** and **2** (Scheme 1 and see Supporting Information for details of synthesis of **1** and **2**). Treatment of alcohol **1** with diphenyl phosphite followed by addition of water showed complete conversion of the nucleoside to the desired *H*-phosphonate. However, complete removal of a phenyl *H*-phosphonate impurity was unsuccessful during purification using silica gel chromatography. As any traces of this impurity in the next step would have led to the formation of unwanted side products, we instead coupled **1** to phosphorous acid using PivCl (Scheme 1). Addition of triethylamine at the end of the reaction neutralized excess acid, forming triethylammonium hydrogenphosphonate, which precipitated in cold THF and was easily removed by filtration. This method allowed complete elimination of phosphorylated impurities without a need of silica gel chromatography, affording gemcitabine *H*-phosphonate derivative **3** in 79% yield. For arabinocytidine 5′-position alcohol **2** we returned to using diphenyl phosphite (Scheme 1), first substituting one of the phenyl groups with this nucleoside. The second phenyl group was then substituted through the addition of water,^21^ and subsequent addition of Et_3_N generated the triethylammonium salt of product **4** in 97% yield. Here we noted that while purification of **4** from phenol and phenyl *H*-phosphonate reaction impurities remained tedious and required a very gradual column gradient, this method could be utilized to access the material, unlike for **3**. Overall, two alternative methods to install 5′-position *H*-phosphonates onto nucleoside analogues were established.

**Scheme 1:**
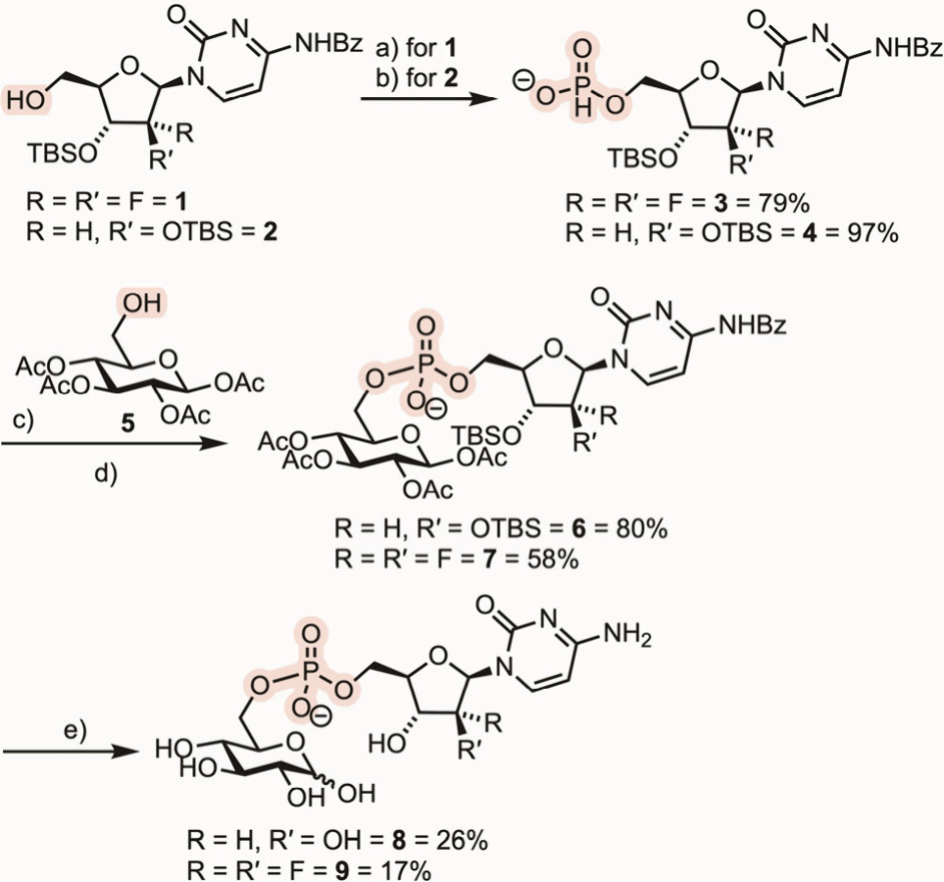
Reagents and Conditions^a^

a (a) (i) H_3_PO_3_, PivCl, pyridine, 2 h, rt; (ii) Et_3_N, H_2_O, 10 min, rt. (b) (i) Diphenyl phosphite, pyridine, 1.5 h, rt; (ii) Et_3_N, H_2_O, 30 min, rt; (c) Piv-Cl, pyridine, MeCN, 2 h, rt; (d) I_2_, pyridine, H_2_O, 30 min, rt; (e) (i) HF/pyridine, pyridine, 20 h, rt; (ii) NH_4_OH, MeOH, 20 h, rt.

We next attempted coupling of a pyranose to *H*-phosphonate **4**, selecting 6′–OH glucose tetraacetate **5** (see Supporting Information for details of synthesis of **5**) and using PivCl as the coupling reagent (Scheme 1). We determined that the addition of a further 1.5 equiv. PivCl after 1.5 h improved conversion and an *H*-phosphonate intermediate was obtained in 84% yield after 3 h. This material was subsequently oxidized using iodine and confirmed by NMR spectroscopy (disappearance of characteristic ^1^H *H*-phosphonate chemical shifts: δ = 6.95 ppm, ^1^ *J*_*H–P*_ = 723 Hz and δ = 6.88 ppm, ^1^ *J*_*H–P*_ = 729 Hz). The acetate protecting groups were stable to this transformation and phosphodiester **6** was isolated in 80% yield over two steps (95% yield for the oxidation). At this juncture, a one-pot procedure was also developed to improve the overall process of the glycoconjugation. This afforded conjugate **6** in 72% yield compared to 80% using the two-step method. Despite this slight decrease in yield, the one-pot method was preferred, to extend the reaction to other targets as it reduced the time each reaction took as well as the total number of workup and purification steps. Accordingly, a similar procedure for gemcitabine *H*-phosphonate **3** yielded phosphodiester **7** in 58% yield.

Next, the nucleoside ring hydroxyl groups were deprotected using HF·pyridine with 91% conversion observed over 72 h. To eliminate the need for an aqueous work up, the reaction was quenched using ethoxytrimethylsilane, which reacted with excess HF to form volatile fluorotrimethylsilane and ethanol. These byproducts were removed *in vacuo*, affording the crude, material that underwent final deesterification using methanolic ammonia to afford crude arabinocytidine glucoconjugate **8**. This material was purified by preparative HILIC chromatography, passed through an Amberlite IR-120 sodium exchange resin, lyophilized and target **8** obtained as a white foam in 26% yield over the two deprotection steps. ^1^H NMR analysis of the main impurities isolated during chromatographic purification indicated degradation of **8**, cleaving the phosphate from either the glucose or the cytarabine component and accounted for the lower-than-expected isolated yield; degradation also occurred slowly over time at room temperature, which meant **8** was stored < −20 °C. To explore increasing the final yield of material, the deprotection order was switched. Compound **7** was solubilized in MeOH and treated with methanolic ammonia, affording a material that could be precipitated using DCM and used without further purification. Upon addition of HF·pyridine to this, OTBS mono deprotection was noted after 24h, however, we were unable to complete full desilylation due to low solubility of the monodeprotected intermediate in pyridine. Based on these observations, the previous ordering for deprotection was retained and gemcitabine glucoconjugate **9** was isolated in 17% yield.

We also wanted to evaluate conjugation of glucose through another pyranose ring position and therefore sought to apply the chemistry developed in Scheme 1 but substitute an appropriate hemiacetal in place of **5**. Using this approach, a C1-*O*-glucoside *H*-phosphonate was shown to form between **4** and **10** (21% by crude NMR), but this material was significantly less stable than its 6′–OH counterpart; during purification, cleavage of the pyranose was observed, regenerating the starting material and with only traces of the desired product isolated. As such, we switched strategy, seeking instead to install the *H*-phosphonate at the glucose anomeric position and then couple the desired nucleoside analogue **1** or **2** (Scheme 2).

**Scheme 2:**
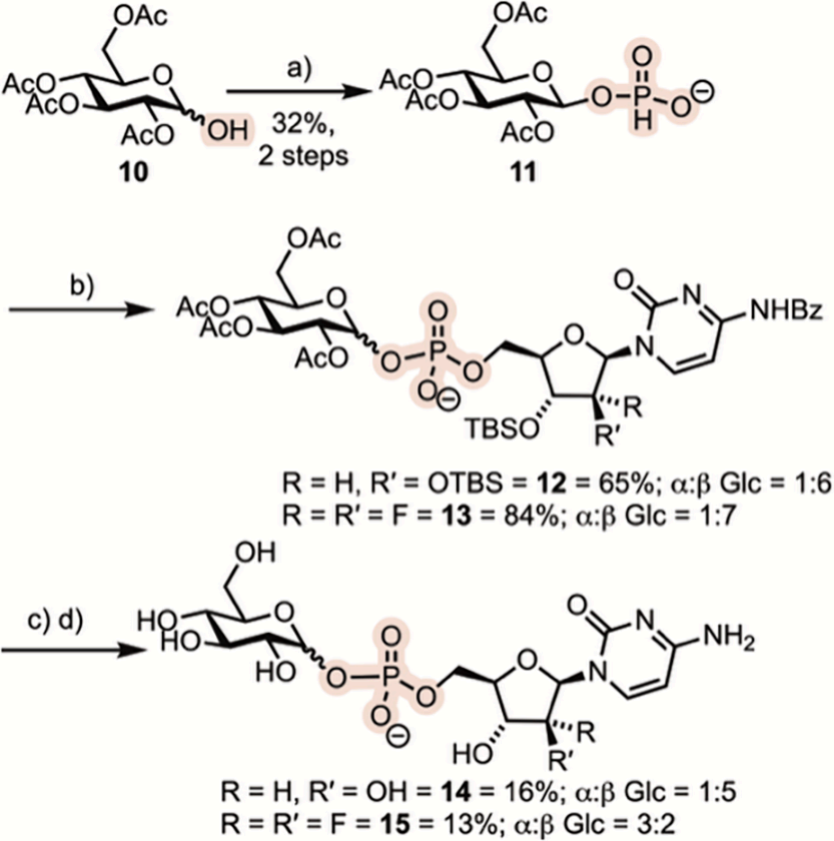
Reagents and Conditions^a^

a (a) (i) Trichloroacetonitrile, DBU, 2 h, 0 °C; (ii) H_3_PO_3_, THF, 5 min, rt; (b) (i) **1** or **2**, Piv-Cl, pyridine, MeCN, 2 h, rt; (ii) I_2_, pyridine, H_2_O, 30 min, rt; (c) HF/pyridine, pyridine, 20 h, rt; (d) NH_4_OH, MeOH, 20 h, rt.

Accordingly, we generated an α-trichloroacetimidate from hemiacetal **10** using trichloroacetonitrile and DBU at 0 °C (Scheme 2), followed by addition of phosphorous acid to form **11** (α/β, 1:10). The excess acid was converted to the triethylamine salt and precipitated from cold THF,^22^ allowing purification of *H*-phosphonate **11**. The structure of this material was confirmed through H1–P coupling (^3^ *J*_*H1–P*_ = 9.4 Hz) and a characteristic H1 chemical shift/coupling constant (δ = 5.08 ppm, ^3^ *J*_*H1–H2*_ = 8.0 Hz). The coupling of **11** to nucleoside **2** was then performed using the one-pot procedure described previously, affording phosphodiester **12** in 65% yield and retaining majority β-isomer at C1 of glucose (α:β Glc = 1:6). A similar result was obtained in forming gemcitabine conjugate **13** (84% yield, α:β Glc = 1:7). The protected glucoconjugates were then deprotected to afford **14** and **15** in 16% and 13% yields, respectively. As previously encountered, cleavage of the internal phosphate across the glucose/nucleoside framework proved problematic in the final deprotection and purification steps, impacting the observed yields. We also selected a series of galactose conjugates to expand the identity of the pyranose component. Galactose scavenging and metabolic remodelling is key in certain cancers and the transporters GLUT3 and GLUT14 are known to accept this sugar.^23^ Furthermore, galactose can be harnessed to improve the specificity and uptake of its conjugates.^24^ When attempting coupling of a comparative acetate-protected C6-OH galactose derivative **16** to **4** using the conditions established for the glucoside targets, only low yields of the corresponding phosphodiester were achieved (37%), and no coupling was observed with **3**. It is possible that the change of stereochemistry at C4 in galactose (to axial) imparted a steric hindrance in attaching to the nucleoside *H*-phosphonate through C6. Instead, and inspired by the C1-*H*-phosphonate chemistry developed for glucose (Scheme 2), we converted acetate **16** to C6-*H*-phopshonate **17** in 68% yield (Scheme 3). From here we coupled with nucleosides **1** or **2**, successfully affording phosphates **18** and **19** in 68% and 37% yields, respectively. Following the established procedure, gemcitabine conjugate **18** was successfully deprotected and purified affording target **20** in 15% yield. Unfortunately, efforts to produce the related cytarabine derivative from protected derivative **19** failed as the material could not be successfully isolated following final deprotections and purification, despite repeated attempts. Finally, we prepared a galactose 1-*O*-*H*-phosphonate **22** using the procedure established for **11,** affording compound **22** in 34% yield over two steps and noting a lack of β-selectivity (α/β, 3:7, Scheme 3). Using **22**, cytarabine and gemcitabine phosphates **23** and **24** were obtained in 66% and 65% yields, respectively. These two compounds were deprotected and purified to afford the final galactose-nucleotides **25** and **26** in 7% and 27% yields respectively.

**Scheme 3:**
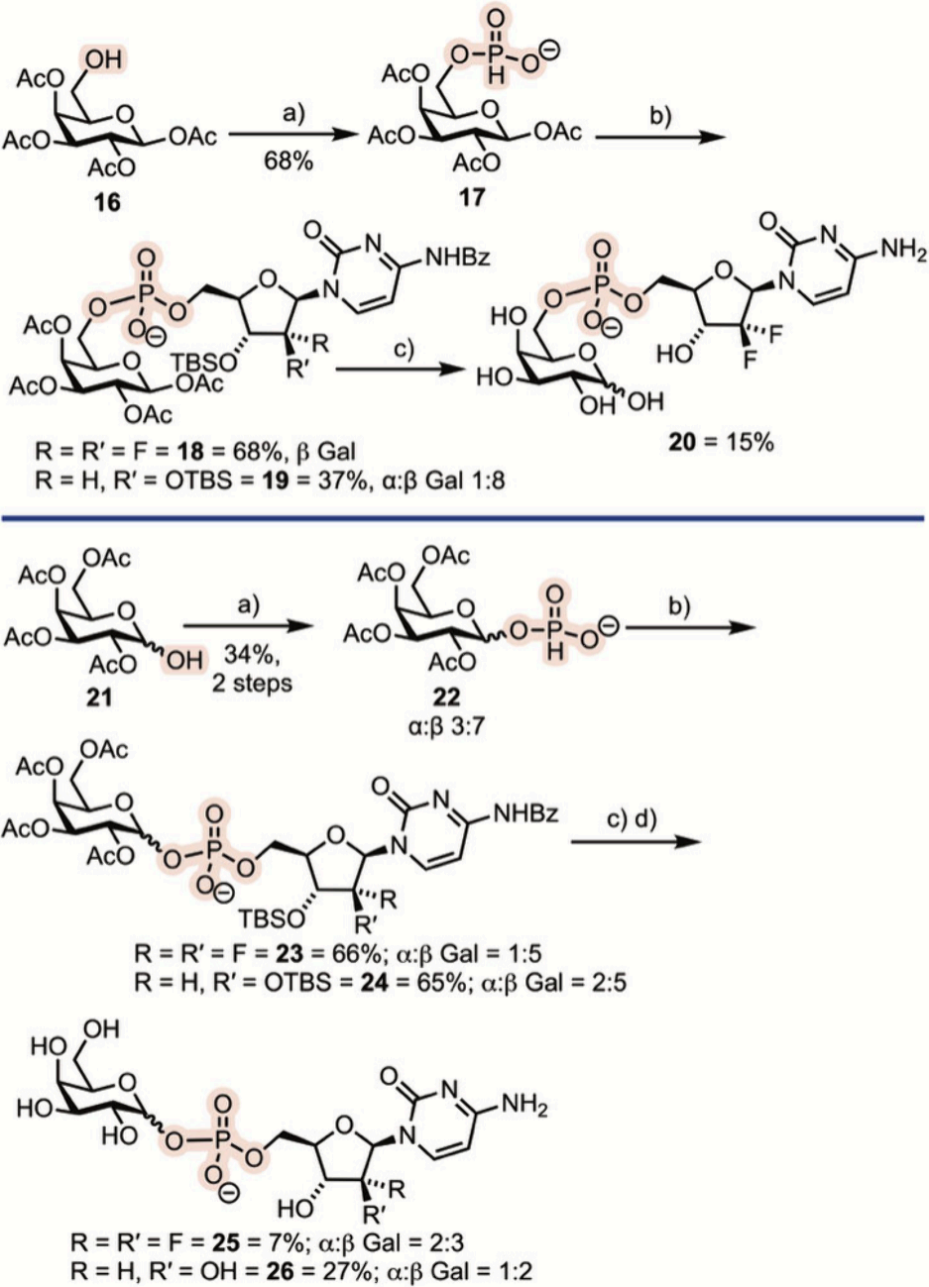
Reagents and Conditions^a^

a (a) (i) Trichloroacetonitrile, DBU, 2 h, 0 °C; (ii) H_3_PO_3_, THF, 5 min, rt; (b) (i) 1 or 2, Piv-Cl, pyridine, MeCN, 2 h, rt; (ii) I_2_, pyridine, H_2_O, 30 min, rt; (c) HF/pyridine, pyridine, 20 h, rt; (d) NH_4_OH, MeOH, 20 h, rt.

Overall, the implementation of an *H*-phosphonate-mediated coupling of pyranoses with nucleosides favored making the reactive phosphorus species within the pyranose component, affording glycoconjugates in good overall yields (65–84%) and enabling simple purification when attaching through both C1 and C6 positions. The reverse coupling (through the nucleoside *H*-phosphonate) was only successful with C6-OH of glucose, for the examples studied.

Nucleotide glycoconjugates **8**, **9**, **14**, **15**, **20**, **25**, & **26** were evaluated in cytotoxicity assays against human pancreatic cancer (PANC-1) and human primary glioblastoma (U87-MG) cells and their CC_50_ values compared to commercial arabinocytidine and gemcitabine (Table 1). Encouragingly, for all four tested gemcitabine conjugates (Table 1, entries 2–5), cytotoxicity was evident at low nM levels in both cell lines, comparable to gemcitabine. Of particular note was C1-glucose conjugate **15**, which exhibited CC_50_ values that were almost identical to the parent cytotoxic nucleoside (4.9 nM for **15** versus 1.8 nM in U87-MG and 33 nM for **15** versus 21 nM in PANC-1). Comparatively, the arabinocytidine conjugates were less cytotoxic (Table 1, entries 7–9), with 1-position glucose and galactose analogues **14** and **26** 2-fold less active versus the parent drug.

**Table 1:** Cytotoxicity Data for Nucleotide Glycoconjugates **8**, **9**, **14**, **15**, **20**, **25**, & **26**, Arabinocytidine and Gemcitabine in U87-MG and PANC-1 cells

Entry	Compound	CC_50_ (nM) U87-MG	CC_50_ (nM) PANC-1
1	Gemcitabine	1.8	21
2	**9**	6.4	89
3	**15**	4.9	33
4	**20**	14	79
5	**25**	8.2	92
6	Arabinocytidine	150	140
7	**8**	1580	7770
8	**14**	310	320
9	**26**	300	360

We have developed a chemical conjugation methodology to combine pyranoses with the cytotoxic nucleoside analogues gemcitabine and cytarabine, creating novel cytidine monophosphate-like sugar-nucleotides. Using protected nucleosides, we initially formed a 5′-*O*-*H*-phosphonate to couple with the 6-OH of glucose tetraactetate. The method was then reversed to create an anomeric glucose *H*-phosphonate for nucleoside conjugation through this position. We also introduce galactose conjugates, noting the pyranose *H*-phosphonate method is preferred for coupling through both C1-OH and C6-OH. Following protected sugar nucleotide formation, the materials were successfully deprotected, purified by HPLC and evaluated as cytotoxic agents, where the gemcitabine conjugates showed biological activity comparable to the parent drug. This supports further study of this motif as a potential prodrug form of established nucleoside analogue drugs and as a novel sugar nucleotide glycosyl donor framework for glycosyltransferases.

## Supplementary Material





## Data Availability

The data underlying this study are available in the published article and its .

## Notes

Experimental Procedures and compound characterization (PDF)
NMR spectra (PDF)
