# Supplementary material for: An H‑Phosphonate-Mediated Synthesis of Nucleotide-Pyranose Glycoconjugates
Source: Org Lett. 2026 Jun 10;28(24):7913–7. doi: 10.1021/acs.orglett.6c02135 (PMC13288873; doi:10.1021/acs.orglett.6c02135)
Supplement: Supplementary file 1 [file ol6c02135_si_001.pdf]

Supporting Information

An *H*-Phosphonate-mediated Synthesis of Nucleotide-Pyranose Glycoconjugates

Thibault Guillaume,[1] Ningwu Huang,[2] Mark Smith[2] and Gavin J. Miller[1,3*]

[1] School of Chemical and Physical Sciences, Keele University, Keele, Staffordshire, ST5 5BG, UK.

[2] Riboscience LLC, 428 Oakmead Pkwy, Sunnyvale, CA 94085, USA.

[3] Manchester Institute of Biotechnology & Department of Chemistry, University of Manchester, Manchester, M1 7DN, UK.

*gavin.miller@manchester.ac.uk

Table of Contents

S1. General Experimental S2

S2. General Procedures S4

S3. Synthesis of Building Blocks S6

S4. Preparation of Glycoconjugates S15

S5. Cytotoxicity Assays S35

S6. References S37

S1. General Experimental

All chemicals were purchased from Acros Organics, Alfa Aesar, Apollo Scientific, Biosynth Carbosynth, Fisher Scientific, Fluorochem, Sigma Aldrich or TCI Chemicals and were used without further purification unless otherwise stated. Anhydrous DMF, pyridine and Et_3N were obtained from Sure/Seal™ bottles *via* chemical suppliers. Anhydrous THF, DCM and toluene were obtained by passing solvent through activated alumina columns and dispensed from a PureSolv MD ASNA solvent purification system and stored in Young's flask over 4 Å molecular sieves under nitrogen. Unless otherwise stated, all reactions were conducted using anhydrous solvents, under an atmosphere of N_2 which was passed through a Drierite™ drying column. For reactions that required heating, DrySyn® heating blocks were used as the heat source. Thin layer chromatography (TLC) was performed using pre-coated 0.25 mm 60 F_{254} silica gel plates (Merck) and the eluents outlined in the respective experiments. Visualisation was achieved using UV light (λ = 254 nm), and $KMnO_4$ staining followed by heating, or 10% H_2SO_4/EtOH staining followed by heating. Flash column chromatography was performed using silica gel (high purity grade, pore size 60 Å, 230-400 mesh particle size, 40-63 µm particle size from Sigma Aldrich) or automated flash chromatography cartridge (FlashPure EcoFlex, 50 µm particle size from Büchi) on a Reveleris C-815 Flash. All final compounds were purified on an Agilent 1260 Infinity II preparative HPLC system equipped with a variable wavelength detector and a fraction collector, on a reverse phase column (Polaris 180 Å C18-A. 21.2 × 250 mm, 5 µm), a HILIC column (HILIC-B, Polaris 5, 250 × 21.2 mm), or a SAX column (PL-SAX 4000 Å, 5 µm) to achieve a purity level >95%. Visualisation was achieved using UV detection at 254 nm and 300 nm. Optical rotations were recorded on a Bellingham + Stanley ADP430 (specific rotation, tube length: 50 mm, concentrations in g/100 mL). High-resolution mass spectra were measured on a ThermoScientific LTQ Orbitrap XL at the ESPRC National Mass Spectrometry Facility at Swansea University, or at Keele University using a UPLC 1290 Infinity II equipped with a QToF 6530B. NMR spectra were recorded on a Bruker Avance 400 spectrometer. The chemical shift data for each signal are given as δ in units of parts per million (ppm) relative to tetramethylsilane, where δ = 0.00 ppm, or the internal solvent peak when overlapping with silyl peak, and to the ^{13}C signal of deuterated chloroform ($CDCl_3$) for ^{13}C NMR spectra. For compounds not reported in the literature, NMR assignments have been made using 2D NMR COSY, HSQC and HMBC.

The number of protons (n) for a given resonance is indicated by nH. The multiplicity of each signal is indicated by: s (singlet), d (doublet), t (triplet), q (quartet), m (multiplet), ov. (overlaping), app. (apparent), br. (broad) or combinations thereof. Coupling constants (*J*) are quoted in Hz and calculated to the nearest 0.1 Hz. Assignment of proton and carbon signals follows the numbering system illustrated below:

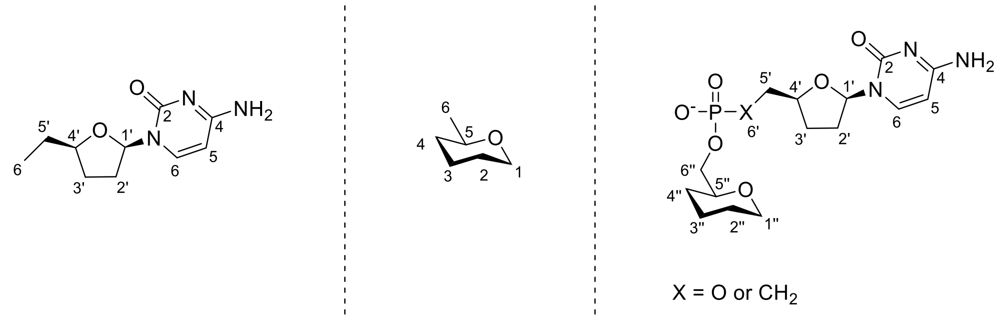

S2. General Procedures

General procedure A: *N*-benzoyl protection of nucleoside analogues

The nucleoside (1.00 mmol, 1.0 equiv.) was dissolved in DCM (10 mL, 0.10 M) and treated with Et_3N (2.00 mmol, 2.0 equiv.), DMAP (0.20 mmol, 0.2 equiv.) and benzoyl chloride (1.50 mmol, 1.5 equiv.). The solution was stirred at rt for 16 h. The reaction was monitored by TLC analysis (DCM/MeOH, 95:5) showing conversion of the starting material to a higher R_f. The reaction was quenched with methanol (1.0 mL) and the volatiles were removed *in vacuo* to provide the crude. The crude residue was purified on a silica gel column to afford the target compound.

General procedure B: selective deprotection of C5'-*O*-TBS of nucleoside analogues

The nucleoside (1.00 mmol, 1.0 equiv.) was dissolved in THF (10 mL, 0.10 M) and was added dropwise to a solution of trifluoroacetic acid (TFA) in H_2O (5 mL, 1:1, *v/v*) at 0 °C (THF/TFA/H_2O 4:1:1). The solution was stirred at 0 °C for 5 h. The reaction was monitored by TLC analysis (hexane/EtOAc, 3:2) showing appearance of the mono deprotected product at a lower R_f. When a second product at lower R_f appeared, indicating secondary deprotection occurring, the reaction was neutralised with sat. aq. $NaHCO_3$ (until pH < 8), then was extracted with EtOAc (3 × 40 mL). The combined organic phases were washed with brine (50 mL), dried over $MgSO_4$, and the volatiles were removed *in vacuo*. The crude residue was purified on a silica gel column to afford the target compound.

General procedure C: addition of *H*-phosphonate moiety

The nucleoside (1.00 mmol, 1.0 equiv.) and was dissolved in pyridine (5.5 mL, 0.18 M) and treated with diphenyl phosphite (2.00 mmol, 2.0 equiv.). The solution was stirred at rt for 2 h. The reaction was monitored by TLC analysis (DCM/MeOH, 98:2, +1% Et_3N) showing appearance of the product at a lower R_f. The solution was treated with Et_3N (5.00 mmol, 5.0 equiv.) and H_2O (10.0 mmol, 10 equiv.) was stirred for 30 min. The volatiles were removed *in vacuo* and co-evaporated with toluene (3 × 10 mL). The crude residue was purified on a silica gel column to afford the target compound.

General procedure D: one pot functionalization and oxidation of *H*-phosphonate derivative

The nucleoside (1.00 mmol, 1.0 equiv.) and pyranoside (1.50 mmol, 1.5 equiv.) were dissolved in pyridine/MeCN (50 mL, 1:1, *v/v*, 0.02 M). The solution was cooled to 0 °C, treated dropwise with PivCl (3.00 mmol, 3.0 equiv.). The solution was stirred at 0 °C for 1.5 h before a second dropwise addition of PivCl was made (1.50 mmol, 1.5 equiv.) and the solution stirred further at rt for 30 min. TLC analysis (DCM/MeOH, 98:2, +1% Et_3N) showed conversion of the starting material to a higher R_f. The reaction solution was treated with a solution of I_2 (1.50 mmol, 1.5 equiv.) in pyridine/H_2O solution (42 mL, 10:1, *v/v*) and stirred at rt for 40 min, at which point the reaction was diluted with DCM (100 mL) and quenched with 1 M $Na_2S_2O_3$ (75 mL). The phases were separated and the aqueous layer was extracted with DCM (3 × 100 mL). The organic phases were combined, dried over $MgSO_4$, volatiles were removed *in vacuo* and co-evaporated with toluene (3 × 100 mL). The crude residue was purified on a silica gel column to afford the target compound.

General procedure E: deprotection of silyl group using HF/pyridine

The nucleoside (1.00 mmol, 1.0 equiv.) was dissolved in pyridine (5 mL, 0.2 M) in a Teflon flask and treated with HF/pyridine (1.0 mL, 40.0 mmol, 40.0 equiv.). The reaction was stirred for 16 h at rt, and the excess HF was scavenged by the dropwise addition of EtOTMS (80.0 mmol, 80.0 equiv.). The solution was stirred at rt for 30 min, and the volatiles were removed *in vacuo*. The crude residue was purified on a silica gel column to afford the target compound.

General procedure F: deprotection of nucleoside acetyl and benzyl groups

The nucleoside (1.00 mmol, 1.0 equiv.) was dissolved in a MeOH/35% NH_4OH solution (20 mL, 1:1, *v/v*, 0.05 M). The reaction was stirred at rt for 16 h, and the volatiles were removed *in vacuo*. The crude material was dissolved in H_2O (5 mL), eluted through a Amberlite™ IR-120 sodium exchange resin column with H_2O and reduced *in vacuo*. The crude product was purified *via* the appropriate HPLC column, passed through Amberlite™ IR-120 sodium exchange resin and lyophilised to afford the target compound.

S3. Synthesis of Building Blocks

3.1 Preparation of Gemcitabine Derivatives

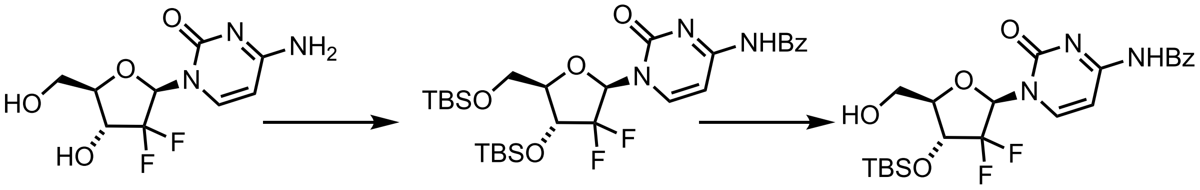

S0 S1 1

3',5'-Di-*O*-*tert*butyldimethylsilyl-*N*-4-benzoyl-2'-deoxy-2',2'-difluorocytidine S1

To a stirred solution of gemcitabine **S0** (5.00 g, 19.0 mmol, 1.0 equiv.) and imidazole (7.76 g, 114 mmol, 6.0 equiv.) in DMF (50 mL), TBSCl (14.3 g, 95.0 mmol, 5.0 equiv.) was added slowly at 0 °C. The reaction solution was allowed to gradually warm to rt and stirred for 16 h. The reaction was monitored by TLC analysis (DCM/MeOH, 95:5) showing conversion of the starting material to a higher R_f. Subsequently, H_2O (300 mL) was added and the solution was stirred for a further 15 min. The aqueous layer was extracted using EtOAc (3 × 200 mL). The combined organic phases were dried over $MgSO_4$ and volatiles were removed *in vacuo* to give the crude product. Purification *via* flash column chromatography on silica gel (DCM/MeOH, 97:3) afforded protected nucleoside 3',5'-Di-*O*-*tert*butyldimethylsilyl-2'-deoxy-2',2'-difluorocytidine as a white powder (9.34 g, 19.0 mmol, quant.). R_f = 0.24 (DCM/MeOH, 95:5); ^{1}H NMR (400 MHz, $CDCl_3$) δ 7.66 (d, $^3J_{H6\text{-}H5}$ = 7.5 Hz, 1H, H6), 6.32 (dd, $^3J_{H1'\text{-}F}$ = 10.7 Hz, $^3J_{H1'\text{-}F}$ = 4.6 Hz, 1H, H1'), 5.70 (d, $^3J_{H5\text{-}H6}$ = 7.5 Hz, 1H, H5), 4.29 (ddd, $^3J_{H3'\text{-}F}$ = 12.2 Hz, $^3J_{H3'\text{-}F}$ = 10.7 Hz, $^3J_{H3'\text{-}H4'}$ = 8.0 Hz, 1H, H3'), 3.98 (app. dt, $^2J_{H5'b\text{-}H5'a}$ = 11.7 Hz, $^3J_{H5'b\text{-}H4'}$ = 2.4 Hz, 1H, H5'b), 3.88 (app. dt, $^3J_{H4'\text{-}H3'}$ = 8.0 Hz, $^3J_{H4'\text{-}H5'b}$ = 2.4 Hz, $^3J_{H4'\text{-}H5'a}$ = 2.0 Hz, 1H, H4'), 3.78 (dd, $^2J_{H5'a\text{-}H5'b}$ = 11.7 Hz, $^3J_{H5'a\text{-}H4'}$ = 2.0 Hz, 1H, H5'a), 0.93 (s, 9H, ^{t}Bu$_{TBS}$), 0.90 (s, 9H, ^{t}Bu$_{TBS}$), 0.12 (s, 3H, Me$_{TBS}$), 0.11 (s, 3H, Me$_{TBS}$), 0.10 (s, 3H, Me$_{TBS}$), 0.09 (s, 3H, Me$_{TBS}$); ^{13}C NMR (101 MHz, $CDCl_3$) δ 166.0 (C2), 155.7 (C4), 140.7 (C6), 122.2 (dd, $^1J_{C2'\text{-}F}$ = 124.6 Hz, $^1J_{C2'\text{-}F}$ = 122.0 Hz, C2'), 95.3 (C5), 84.4 (dd, $^2J_{C1'\text{-}F}$ = 40.0 Hz, $^2J_{C1'\text{-}F}$ = 23.5 Hz, C1'), 81.0 (d, $^3J_{C4'\text{-}F}$ = 8.8 Hz, C4'), 69.9 (dd, $^2J_{C3'\text{-}F}$ = 27.5 Hz, $^2J_{C3'\text{-}F}$ = 18.0 Hz, C3'), 60.2 (C5'), 26.0(^{t}Bu$_{TBS}$), 25.7(^{t}Bu$_{TBS}$), 18.4(C$_{TBS}$), 18.1(C$_{TBS}$), –4.6(Me$_{TBS}$), –5.4(Me$_{TBS}$), –5.4(Me$_{TBS}$); ^{19}F NMR (377 MHz, $CDCl_3$) δ –115.9 (app. dd, $^2J_{F\text{-}F}$ = 238.4 Hz, $^3J_{F\text{-}H3'}$ = 11.8 Hz, F), –117.5 (dt, $^2J_{F\text{-}F}$ = 238.8 Hz, $^3J_{F\text{-}H3'}$ = $^3J_{F\text{-}H1'}$ = 10.7 Hz, F); HRMS (ESI$^+$) *m/z*: [M + H]$^+$ Calcd for

$C_{21}H_{40}F_2N_3O_4Si_2$ 492.2520; Found 492.2538. These data were in agreement with literature.[1]

Gemcitabine derivative **S1** (2.22 g, 3.73 mmol, 98%), a white powder, was obtained following general **A** from 3',5'-di-*O*-*tert*butyldimethylsilyl-2'-deoxy-2',2'-difluorocytidine (1.87 g, 3.80 mmol, 1.0 equiv.). The crude material was purified *via* flash column chromatography on silica gel (hexane/EtOAc, 4:1→1:1). R_f = 0.34 (hexane/EtOAc, 7:3); $[\alpha]^{19.5}_D$ + 67.8 (c = 1.0, $CHCl_3$); mp 165-168 °C; ^{1}H NMR (400 MHz, $CDCl_3$) δ 8.86 (s, 1H, NH) 8.14 (d, $^3J_{H6\text{-}H5}$ = 7.5 Hz, 1H, H6), 7.91 (d, $^3J_{HAr\text{-}HAr}$ = 7.5 Hz, 2H, 2 × HAr), 7.66 – 7.60 (m, 1H, HAr), 7.57 – 7.42 (m, 3H, HAr, H5), 6.37 (dd, $^3J_{H1'\text{-}F}$ = 10.4 Hz, $^3J_{H1'\text{-}F}$ = 3.8 Hz, 1H, H1'), 4.37 (ddd, $^3J_{H3'\text{-}F}$ = 11.6 Hz, $^3J_{H3'\text{-}F}$ = 11.5 Hz, $^3J_{H3'\text{-}H4'}$ = 8.0 Hz, 1H, H3'), 4.04 (dt, $^2J_{H5'a\text{-}H5'b}$ = 11.8 Hz, $^3J_{H5'a\text{-}H4'}$ = 2.1 Hz, 1H, H5'a), 3.98 (br. d, $^3J_{H4'\text{-}H3'}$ = 8.1 Hz, 1H, H4'), 3.83 (dd, $^2J_{H5'b\text{-}H5'a}$ = 11.8 Hz, $^3J_{H5'b\text{-}H4'}$ = 1.9 Hz, 1H, H5'b), 0.97 (s, 9H, ^{t}Bu$_{TBS}$), 0.91 (s, 9H, ^{t}Bu$_{TBS}$), 0.15 (s, 3H, Me$_{TBS}$), 0.15 (s, 3H, Me$_{TBS}$), 0.14 (s, 3H, Me$_{TBS}$), 0.11 (s, 3H, Me$_{TBS}$); ^{13}C NMR (101 MHz, $CDCl_3$) δ 162.6 (C4/C2), 144.2 (C6), 133.3 (C$_{Ar}$), 129.1 (C$_{Ar}$), 127.7 (C$_{Ar}$), 122.0 (t, $^1J_{C2'\text{-}F}$ = 261.0 Hz, C2'), 84.7 (dd, $^2J_{C1'\text{-}F}$ = 40.7 Hz, $^2J_{C1'\text{-}F}$ = 23.9 Hz, C1'), 81.5 (d, $^3J_{C4'\text{-}F}$ = 8.7 Hz, C4'), 69.5 (dd, $^2J_{C3'\text{-}F}$ = 27.3 Hz, $^2J_{C3'\text{-}F}$ = 18.1 Hz, C3'), 60.0 (C5'), 25.9 (^{t}Bu$_{TBS}$), 25.5 (^{t}Bu$_{TBS}$), 18.3 (C$_{TBS}$), 18.0 (C$_{TBS}$), –4.75 (Me$_{TBS}$), –5.3 (Me$_{TBS}$), –5.4 (Me$_{TBS}$), –5.4 (Me$_{TBS}$); ^{19}F NMR (377 MHz, $CDCl_3$) δ –115.9 (app. dd, $^2J_{F\text{-}F}$ = 239.5 Hz, $^3J_{F\text{-}H3'}$ = 12.2 Hz, F), –117.4 (dt, $^2J_{F\text{-}F}$ = 239.5 Hz, $^3J_{F\text{-}H1'}$ = 10.8 Hz, $^3J_{F\text{-}H3'}$ = 10.8 Hz, F). HRMS (ESI$^+$) *m/z*: [M + H]$^+$ Calcd for $C_{28}H_{44}F_2N_3O_5Si_2$ 596.2788; Found 596.2786.

3'-*O*-*tert*Butyldimethylsilyl-*N*-4-benzoyl-2'-deoxy-2',2'-difluorocytidine 1

Gemcitabine derivative **1** (656 mg, 1.36 mmol, 81%), a white powder, was obtained following general procedure **B** from nucleoside derivative **S1** (1.00 g, 1.68 mmol, 1.0 equiv.). The crude material was purified *via* flash column chromatography on silica gel (hexane/EtOAc, 7:3→2:3). R_f = 0.58 (hexane/EtOAc, 2:3); $[\alpha]^{19.5}_D$ + 55.2 (c = 1.0, $CHCl_3$); mp 202-205 °C; ^{1}H NMR (400 MHz, $CDCl_3$) 8.13 (d, $^3J_{H6\text{-}H5}$ = 7.7 Hz, 1H, H6), 7.85– 7.78 (m, 2H, 2 × HAr), 7.60 – 7.50 (m, 2H, HAr, H5), 7.43 (app. t, $^3J_{HAr\text{-}HAr}$ = 7.8 Hz, 2H, 2 × HAr), 6.28 (app. t, $^3J_{H1'\text{-}F}$ = 7.4 Hz, $^3J_{H1'\text{-}F}$ = 7.4 Hz, 1H, H1'), 4.42 (app. td, $^3J_{H3'\text{-}F}$ = 11.5 Hz, $^3J_{H3'\text{-}F}$ = 11.5 Hz, $^3J_{H3'\text{-}H4'}$ = 8.1 Hz, 1H, H3'), 4.09 (dd, $^2J_{H5'a\text{-}H5'b}$ = 12.7 Hz, $^3J_{H5'a\text{-}H4'}$ = 2.6 Hz, 1H, H5'a), 3.97 (dt, $^3J_{H4'\text{-}H3'}$ = 8.1 Hz, $^3J_{H4'\text{-}H5'a}$ = 2.6 Hz, $^3J_{H4'\text{-}H5'b}$ = 2.6 Hz, 1H, H4'), 3.81 (dd, $^2J_{H5'b\text{-}H5'a}$ = 12.7 Hz, $^3J_{H5'b\text{-}H4'}$ = 2.6 Hz, 1H, H5'b), 0.90 (s, 9H, ^{t}Bu$_{TBS}$), 0.12 (s, 3H, Me$_{TBS}$), 0.12 (s, 3H, Me$_{TBS}$); ^{1}H

NMR (400 MHz, DMSO-d$_6$) δ 12.22 (br. s, 1H, NH), 9.12 (d, $^3J_{H6\text{-}H5}$ = 7.6 Hz, 1H, H6), 8.89 – 8.80 (m, 2H, HAr), 8.47 (t, ^3J = 7.4 Hz, 1H, HAr), 8.35 (t, ^3J = 7.7 Hz, 2H, HAr), 8.26 (d, $^3J_{H5\text{-}H6}$ = 7.6 Hz, 1H, H5), 7.12 – 7.01 (m, 1H, H1'), 6.19 (t, $^3J_{OH\text{-}H5'a}$ = 5.3 Hz, $^3J_{OH\text{-}H5'b}$ = 5.3 Hz, 1H, OH), 5.24 (td, $^3J_{H3'\text{-}F}$ = 12.1 Hz, $^3J_{H3'\text{-}H4'}$ = 8.4 Hz, 1H, H3'), 4.77 (dt, $^3J_{H4'\text{-}H3'}$ = 8.2 Hz, $^3J_{H4'\text{-}H5'b}$ = 2.8 Hz, 1H, H4'), 4.67 (app. d, $^2J_{H5'a\text{-}H5'b}$ = 12.7 Hz, 1H, H5'a), 4.46 (ddd, $^2J_{H5'b\text{-}H5'a}$ = 12.7 Hz, $^3J_{5'b\text{-}OH}$ = 5.9 Hz, $^3J_{H5'b\text{-}H4'}$ = 3.5 Hz, 1H, H5'b), 1.72 (s, 9H, ^{t}Bu$_{TBS}$), 0.96 (s, 3H, Me$_{TBS}$), 0.94 (s, 3H, Me$_{TBS}$); ^{13}C NMR (101 MHz, CDCl3) δ 163.1 (C2), 155.1 (C4), 145.3 (C6), 133.4(C$_{Ar}$), 132.9 (C$_{Ar}$), 129.1 (C$_{Ar}$), 127.8 (C$_{Ar}$), 122.1 (t, $^1J_{C2'\text{-}F}$ = 261.3 Hz, C2'), 97.6 (C5), 86.5 – 84.3 (m, C1'), 81.9 (d, $^3J_{C4'\text{-}F}$ = 8.2 Hz, C4'), 70.1 (t, $^2J_{C3'\text{-}F}$ = 21.8 Hz, C3'), 59.5 (C5'), 25.6 (^{t}Bu$_{TBS}$), 18.1 (C$_{TBS}$), –4.8 (Me$_{TBS}$), –5.1 (Me$_{TBS}$); ^{19}F NMR (377 MHz, CDCl$_3$) δ –115.4 (dd, $^2J_{F\text{-}F}$ = 238.9 Hz, $^3J_{F\text{-}H3'}$ = 9.0 Hz, F), –116.5 (app. d, $^2J_{F'\text{-}F}$ = 246.1 Hz, F); HRMS (ESI$^+$) *m/z*: [M + Na]$^+$ Calcd for $C_{22}H_{29}F_2N_3NaO_5Si$ 504.1742; Found 504.1727.

3.2 Preparation of Cytarabine Derivatives

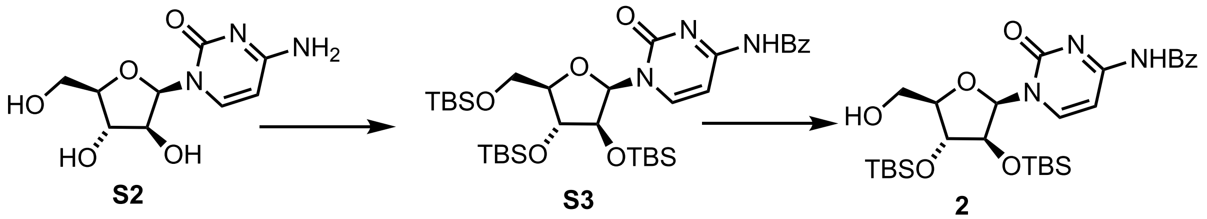

S2 **S3** **2**

2',3',5'-Tri-*O*-*tert*butyldimethylsilyl-*N*-4-benzoyl-arabinocytidine S3

To a suspension of cytarabine **S2** (500 mg, 2.06 mmol, 1.0 equiv.) in THF (21 mL) was added pyridine (778 µL, 9.76 mmol, 4.7 equiv.) and $AgNO_3$ (1.15 g, 6.78 mmol, 3.3 equiv.). The mixture was stirred for 5 min before addition of TBSCl (1.02 g, 6.79 mmol, 3.3 equiv.). After stirring at rt for 2 h, Et_3N (1.1 mL, 7.71 mmol, 3.7 equiv.) and TBSCl (1.02 g, 6.79 mmol, 3.3 equiv.) were added. After stirring for a further 18 h, the reaction was monitored by TLC analysis (DCM/MeOH, 95:5) showing conversion of the starting material to a higher R_f. The reaction was filtered over celite to remove Ag salts. The filter pad was washed with DCM (10 mL) and sat. aq. $NaHCO_3$ (10 mL) was added to the filtrate. The phases were separated and the aqueous phase was extracted with DCM (3 × 20 mL). The organic phases were combined, dried over $MgSO_4$ and the volatiles were removed *in vacuo* to give the crude material. Purification *via* flash column chromatography on silica gel (DCM/MeOH, 100:0→95:5) afforded protected nucleoside 2',3',5'-Tri-*O*-

*tert*butyldimethylsilyl-arabinocytidine (1.11 g, 1.89 mmol, 92%) as a white powder. R_f = 0.37 (DCM/MeOH, 95:5); ^{1}H NMR (400 MHz, $CDCl_3$) δ 7.55 (d, $^3J_{H6-H5}$ = 7.4 Hz, 1H, H6), 6.23 (d, $^3J_{H1'-H2'}$ = 2.9 Hz, 1H, H1'), 5.67 (d, $^3J_{H5-H6}$ = 7.4 Hz, 1H, H5), 4.20 (d, $^3J_{H2'-H1'}$ = 3.0 Hz, 1H, H2'), 4.15 (s, 1H, H3'), 3.96 (dd, $^3J_{H4'-H5'b}$ = 8.7 Hz, $^3J_{H4'-H5'a}$ = 5.9 Hz, 1H, H4'), 3.80 (dd, $^2J_{H5'a-H5'b}$ = 9.5 Hz, $^3J_{H5'a-H4'}$ = 5.9 Hz, 1H, H5'a), 3.73 (dd, $^2J_{H5'b-H5'a}$ = 9.6 Hz, $^3J_{H5'b-H4'}$ = 8.7 Hz, 1H, H5'b), 0.90 (s, 9H, ^{t}Bu$_{TBS}$), 0.89 (s, 9H, ^{t}Bu$_{TBS}$), 0.79 (s, 9H, ^{t}Bu$_{TBS}$), 0.10 (s, 3H, Me$_{TBS}$), 0.10 (s, 3H, Me$_{TBS}$), 0.07 (s, 3H, Me$_{TBS}$), 0.06 (s, 3H, Me$_{TBS}$), 0.03 (s, 3H, Me$_{TBS}$), –0.15 (s, 3H, Me$_{TBS}$); ^{13}C NMR (101 MHz, $CDCl_3$) δ 165.6 (C2), 155.6 (C4), 143.5 (C6), 92.7 (C5), 88.0 (C1'), 87.1 (C4'), 78.7 (C3'), 76.6 (C2'), 63.2 (C5'), 26.0 (^{t}Bu$_{TBS}$), 25.9 (^{t}Bu$_{TBS}$), 25.8 (^{t}Bu$_{TBS}$), 18.4 (C$_{TBS}$), 18.0 (C$_{TBS}$), 17.9 (C$_{TBS}$), –4.5 (2 × Me$_{TBS}$), –5.0 (Me$_{TBS}$), –5.2 (Me$_{TBS}$), –5.2 (Me$_{TBS}$), –5.3 (Me$_{TBS}$); HRMS (ESI$^+$) *m/z*: [M + H]$^+$ Calcd for $C_{27}H_{56}N_3O_5Si_3$ 586.3528; Found 586.3521. These data were in agreement with literature.[2]

Arabinocytidine derivative **S3** (1.25 g, 1.81 mmol, 95%), a white powder, was obtained following general procedure **A** from nucleoside derivative 2',3',5'-Tri-*O*-*tert*butyldimethylsilyl-arabinocytidine (1.11 g, 1.89 mmol, 1.0 equiv). The crude material was purified *via* flash column chromatography on silica gel (hexane/EtOAc, 4:1→1:1). R_f = 0.25 (hexane/EtOAc, 7:3); ^{1}H NMR (400 MHz, $CDCl_3$) δ 8.63 (br. s, 1H, NH), 7.96 (d, $^3J_{H6-H5}$ = 7.5 Hz, 1H, H6), 7.90 (d, $^3J_{HAr-HAr}$ = 7.3 Hz, 2H, 2 × HAr), 7.60 (td, $^3J_{HAr-HAr}$ = 7.4 Hz, $^4J_{HAr-HAr}$ = 1.2 Hz, 1H, HAr), 7.55 – 7.47 (m, 3H, 2 × HAr, H5), 6.27 (d, $^3J_{H1'-H2'}$ = 3.0 Hz, 1H, H1'), 4.33 (d, $^3J_{H2'-H1'}$ = 3.0 Hz, 1H, H2'), 4.19 (app. s, 1H, H3'), 4.04 (dd, $^3J_{H4'-H5'a}$ = 8.6 Hz, $^3J_{H4'-H5'b}$ = 6.2 Hz, 1H, H4'), 3.86 (dd, $^2J_{H5'b-H5'a}$ = 9.6 Hz, $^3J_{H5'b-H4'}$ = 6.2 Hz, 1H, H5'b), 3.77 (dd, $^2J_{H5'a-H5'b}$ = 9.6 Hz, $^3J_{H5'a-H4'}$ = 8.6 Hz, 1H, H5'a), 0.92 (s, 9H, ^{t}Bu$_{TBS}$), 0.91 (s, 9H, ^{t}Bu$_{TBS}$), 0.78 (s, 9H, ^{t}Bu$_{TBS}$), 0.13 (s, 3H, Me$_{TBS}$), 0.12 (s, 3H, Me$_{TBS}$), 0.10 (s, 3H, Me$_{TBS}$), 0.09 (s, 3H, Me$_{TBS}$), 0.05 (s, 3H, Me$_{TBS}$), –0.16 (s, 3H, Me$_{TBS}$); ^{13}C NMR (101 MHz, $CDCl_3$) δ 162.0 (C4/C2), 146.8 (C6), 133.3 (2 × C$_{Ar}$), 129.2 (2 × C$_{Ar}$), 127.7 (2 × C$_{Ar}$), 88.9 (C1'), 87.8 (C4'), 78.6 (C2'), 76.5 (C3'), 63.1 (C5'), 26.0 (s, ^{t}Bu$_{TBS}$), 25.9 (^{t}Bu$_{TBS}$), 25.8 (^{t}Bu$_{TBS}$), 18.5 (C$_{TBS}$), 18.1 (C$_{TBS}$), 17.9 (C$_{TBS}$), –4.5 (Me$_{TBS}$), –4.5 (Me$_{TBS}$), –5.0 (Me$_{TBS}$), –5.2 (Me$_{TBS}$), –5.2 (Me$_{TBS}$), –5.3 (Me$_{TBS}$); HRMS (ESI$^+$) *m/z*: [M + H]$^+$ Calcd for $C_{34}H_{60}N_3O_6Si_3$ 690.3790; Found 690.3792. These data were in agreement with literature.[2]

2',3'-Di-*O*-*tert*butyldimethylsilyl-*N*-4-benzoyl-arabinocytidine 2

Cytarabine derivative **2** (3.73 g, 6.48 mmol, 76%), a white powder, was obtained following general procedure **B** from nucleoside derivative **S3** (5.84 g, 8.46 mmol, 1.0 equiv.). The crude material was purified *via* flash column chromatography on silica gel (hexane/EtOAc, 7:3→2:3). R_f = 0.63 (hexane/EtOAc, 3:2); ^{1}H NMR (400 MHz, $CDCl_3$) δ 8.71 (br. s, 1H, NH), 8.10 (d, $^3J_{H6-H5}$ = 7.5 Hz, 1H, H6), 7.88 (d, $^3J_{HAr-HAr}$ = 7.5 Hz, 2H, 2 × HAr), 7.64 – 7.56 (m, 1H, HAr), 7.49 (m, 3H, 2 × HAr, H5), 6.26 (d, $^3J_{H1'-H2'}$ = 2.4 Hz, 1H, H1'), 4.34 (d, $^3J_{H2'-H1'}$ = 2.4 Hz, 1H, H2'), 4.14 (app. t, $^3J_{H4'-H5'}$ = 4.8 Hz, 1H, H4'), 4.11 (app. s, 1H, H3'), 3.87 (app. d, $^3J_{H5'-H4'}$ = 4.8 Hz, 2H, H5'a, H5'b), 0.91 (s, 9H, ^{t}Bu$_{TBS}$), 0.79 (s, 9H, ^{t}Bu$_{TBS}$), 0.12 (s, 3H, Me$_{TBS}$), 0.10 (s, 3H, Me$_{TBS}$), 0.05 (s, 3H, Me$_{TBS}$), –0.16 (s, 3H, Me$_{TBS}$); ^{13}C NMR (101 MHz, $CDCl_3$) 171.3 (C=O), 162.1 (C=O), 146.7 (C6), 133.3 (C$_{Ar}$), 129.1 (C$_{Ar}$), 127.6 (C$_{Ar}$), 95.6 (C5), 88.6 (C1'), 88.2 (C4'), 79.4 (C3'), 76.3 (C2'), 62.9 (C5'), 25.8 (*t*-Bu$_{TBS}$), 25.7 (*t*-Bu$_{TBS}$), 18.0 (C$_{TBS}$), 17.9 (C$_{TBS}$), –4.4 (Me$_{TBS}$), –4.6 (Me$_{TBS}$), –5.1 (Me$_{TBS}$), –5.2 (Me$_{TBS}$); HRMS (ESI$^+$) *m/z*: [M + H]$^+$ Calcd for $C_{28}H_{46}N_3O_6Si_2$ 576.2925; Found 576.2923. These data were in agreement with literature.[2]

3.3 Preparation of 6-OH Pyranoses

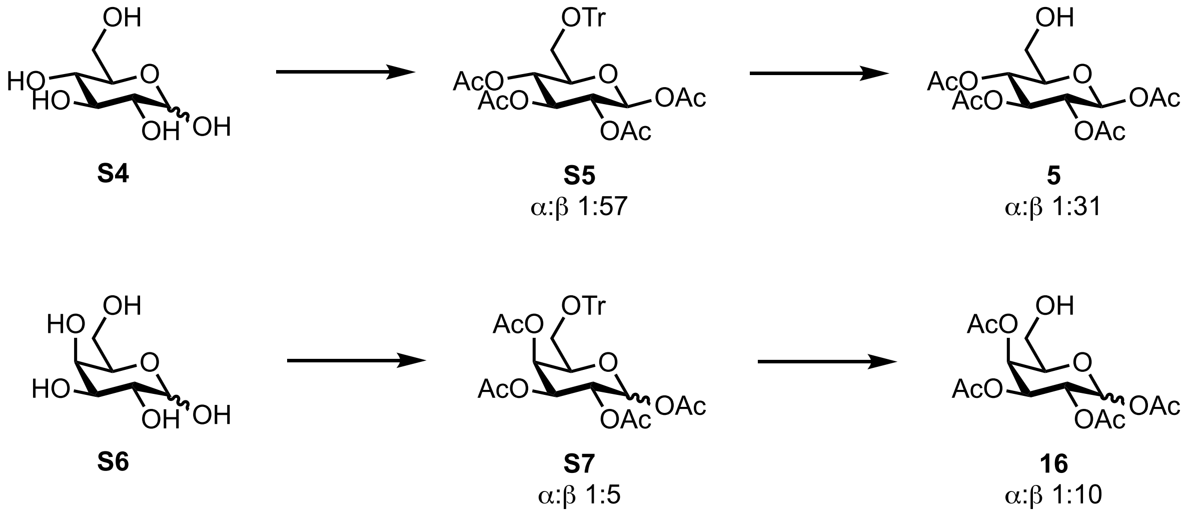

1,2,3,4-Tetra-*O*-acetyl-6-*O*-trityl-β-D-glucopyranoside S5

D-glucose **S4** (2.00 g, 11.1 mmol, 1.0 equiv.) was dissolved in pyridine (10.0 mL) and treated with trityl chloride (3.40 g, 12.2 mmol, 1.1 equiv.). The solution was stirred at 40 °C for 20 h. The solution was treated with Ac_2O (6.3 mL, 66.6 mmol, 6.0 equiv.) and was stirred for 2 h. The reaction solution was cooled to rt, was poured over ice and

extracted with DCM (100 mL). The organic layer was washed with H_2O (3 x 100 mL), brine (50 mL), dried over $MgSO_4$ and the volatiles were removed *in vacuo* to give an anomeric mixture (5.61 g, 9.84 mmol, α:β 45:55, 84%) as a white powder. The crude was washed with cold Et_2O affording the β-anomer **S5** (2.58 g, 4.37 mmol, α:β 1:57, 39%) as a white powder. R_f = 0.80 (hexane/EtOAc, 1:1). ^{1}H NMR (400 MHz, $CDCl_3$) δ 7.46 – 7.40 (m, 6H, HAr), 7.31 – 7.25 (m, 6H, HAr), 7.25 – 7.19 (m, 3H, HAr), 5.74 – 5.70 (m, 1H, H1), 5.30 – 5.23 (m, 1H, H4), 5.23 – 5.13 (m, 2H, H2, H3), 3.69 (ddd, $^3J_{H5-H4}$ = 9.8 Hz, $^3J_{H5-H6b}$ = 4.2 Hz, $^3J_{H5-H6a}$ = 2.4 Hz, 1H, H5), 3.33 (dd, $^2J_{H6a-H6b}$ = 10.6 Hz, $^3J_{H6a-H5}$ = 2.4 Hz, 1H, H6a), 3.07 (dd, $^2J_{H6b-H6a}$ = 10.6 Hz, $^3J_{H6ab-H6a}$ = 4.2 Hz, 1H, H6b), 2.15 (s, 3H, CH_{3Ac}), 2.04 (s, 3H, CH_{3Ac}), 2.00 (s, 3H, CH_{3Ac}), 1.73 (s, 3H, CH_{3Ac}); ^{13}C NMR (101 MHz, $CDCl_3$) δ 170.4 (C=O_{Ac}), 169.5 (C=O_{Ac}), 169.1 (C=O_{Ac}), 169.1 (C=O_{Ac}), 143.6 (C_{Ar}),128.9 (C_{Ar}), 127.9 (C_{Ar}), 127.2 (C_{Ar}), 92.1 (C1), 86.8 (C_{Tr}), 74.2 (C5), 73.3 (C2), 70.7 (C3), 68.5 (C4), 61.8 (C6), 21.0 (CH_{3Ac}), 20.8 (CH_{3Ac}), 20.7 (CH_{3Ac}), 20.6 (CH_{3Ac}); HRMS (ESI$^+$) *m/z*: [M + Na]$^+$ Calcd for $C_{33}H_{34}NaO_{10}$ 613.2050; Found 613.2044. These data were in agreement with literature.[3]

1,2,3,4-Tetra-*O*-acetyl-β-D-glucopyranoside 5

Glucopyranoside **S5** (2.58 g, 4.37 mmol, 1.0 equiv.) was dissolved in acetic acid (11 mL), cooled to 10 °C and treated with HBr (30% in acetic acid, 1.0 mL, 4.81 mmol, 1.1 equiv.) The reaction solution was stirred at 10 °C for 30 seconds, filtered and washed with H_2O (10 mL). The filtrate was diluted with H_2O (10 mL) and extracted with DCM (200 mL). The organic phase was washed with small amount of cold H_2O (2 × 25 mL) to remove acetic acid, dried over $MgSO_4$ and the volatiles were removed in vacuo to give the crude product. Purification *via* flash silica column chromatography (hexane/EtOAc, 4:1→2:1) afforded protected pyranoside **5** (1.27 g, 3.70 mmol, 83%) as a white solid. R_f = 0.36 (hexane/EtOAc, 1:1). ^{1}H NMR (400 MHz, $CDCl_3$) δ 5.70 (d, $^3J_{H1-H2}$ = 8.3 Hz, 1H, H1), 5.28 (t, $^3J_{H3-H2}$ = $^3J_{H3-H4}$ = 9.5 Hz, 1H, H3), 5.13 – 5.01 (m, 2H, H2, H4), 3.74 (ddd, $^2J_{H6a-H6b}$ = 12.6 Hz, $^3J_{H6a-OH}$ = 8.0 Hz, $^3J_{H6a-H5}$ = 2.2 Hz, 1H, H6a), 3.63 (ddd, $^3J_{H5-H4}$ = 9.9 Hz, $^3J_{H5-H6b}$ = 4.2 Hz, $^3J_{H5-H6a}$ = 2.2 Hz, 1H, H5), 3.56 (ddd, $^2J_{H6b-H6a}$ = 12.6 Hz, $^3J_{H6b-OH}$ = 5.6 Hz, $^3J_{H6b-H65}$ = 4.2 Hz, 1H, H6b), 2.41 – 2.33 (m, 1H, OH), 2.08 (s, 3H, CH_{3Ac}), 2.04 (s, 3H, CH_{3Ac}), 2.01 (s, 3H, CH_{3Ac}), 2.00 (s, 3H, CH_{3Ac}). ^{13}C NMR (101 MHz, $CDCl_3$) δ 170.3 (C=O_{Ac}), 170.2 (C=O_{Ac}), 169.4 (C=O_{Ac}), 169.2 (C=O_{Ac}), 91.8 (C1), 75.0 (C5), 72.7 (C3), 70.5 (C2), 68.3 (C4), 60.9 (C6), 20.9

(CH_{3Ac}), 20.7 (CH_{3Ac}), 20.7 (CH_{3Ac}), 20.6 (CH_{3Ac}); HRMS (ESI$^+$) *m/z*: $[M + NH_4]^+$ Calcd for $C_{14}H_{24}NO_{10}$ 366.1400; Found 366.1407. These data were in agreement with literature.[3]

1,2,3,4-Tetra-*O*-acetyl-6-*O*-trityl-α/β-D-galactopyranoside S7

D-galactose **S6** (2.50 g, 13.9 mmol, 1.0 equiv.) was dissolved in pyridine (12.5 mL) and treated with trityl chloride (4.26 g, 15.3 mmol, 1.1 equiv.). The solution was stirred at 40 °C for 20 h. The solution was treated with Ac_2O (7.9 mL, 83.2 mmol, 6.0 equiv.) and was stirred for 2 h. The reaction solution was cooled to rt, poured over ice and extracted with DCM (100 mL). The organic layer was washed with H_2O (3 × 100 mL), brine (50 mL), dried over $MgSO_4$ and the volatiles were removed *in vacuo* to give the crude product. Purification *via* flash silica column chromatography (hexane/Et_2O, 1:1→1:9) afforded protected pyranoside **S7** (3.77 g, 6.38 mmol, 46%, α:β 1:5) as a white solid. R_f = 0.48 (hexane/Et_2O, 3:7); β anomer: ^{1}H NMR (400 MHz, $CDCl_3$) δ 7.47 – 7.32 (m, 6H, 6 × HAr), 7.32 – 7.16 (m, 9H, 6 × HAr), 5.67 (d, $^3J_{H1-H2}$ = 8.2 Hz, 1H, H1), 5.65 (dd, $^3J_{H4-H3}$ = 3.4 Hz, $^3J_{H4-H5}$ = 1.2 Hz, 1H, H4), 5.26 (dd, $^3J_{H2-H3}$ = 10.5 Hz, $^3J_{H2-H1}$ = 8.2 Hz, 1H, H2), 5.12 (dd, $^3J_{H3-H2}$ = 10.5, $^3J_{H3-H4}$ = 3.4 Hz, 1H, H3), 3.95 (ddd, $^3J_{H5-H6a}$ = 8.7 Hz, $^3J_{H5-H6b}$ = 5.3 Hz, $^3J_{H5-H4}$ = 1.2 Hz, 1H, H4) 3.40 (dd, $^2J_{H6b-H6a}$ = 8.7 Hz, $^2J_{H6b-H5}$ = 5.3 Hz, 1H, H6b), 3.07 (t, $^2J_{H6a-H6b}$ = 8.7 Hz, $^3J_{H6a-H5}$ = 8.7 Hz, 1H, H6a), 2.07 (s, 3H, CH_{3Ac}), 2.03 (s, 3H, CH_{3Ac}), 2.00 (s, 3H, CH_{3Ac}), 1.88 (s, 3H, CH_{3Ac}); ^{13}C NMR (101 MHz, $CDCl_3$) δ 170.2 ($C=O_{Ac}$), 169.9 ($C=O_{Ac}$), 169.5 ($C=O_{Ac}$), 169.1 ($C=O_{Ac}$), 143.3 (C_{Ar}), 128.8 (C_{Ar}), 128.1 (C_{Ar}), 127.3 (C_{Ar}), 92.4 (C1), 87.1 (C_{Tr}), 73.0 (C5), 71.2 (C3), 68.3 (C2), 67.0 (C4), 60.4 (C6), 20.9 (CH_{3Ac}), 20.8 (CH_{3Ac}), 20.8 (CH_{3Ac}), 20.7 (CH_{3Ac}); HRMS (ESI$^+$) *m/z*: $[M + Na]^+$ Calcd for $C_{33}H_{34}NaO_{10}$ 613.2050; Found 613.2044. These data were in agreement with literature.[4]

1,2,3,4-Tetra-*O*-acetyl-α/β-D-galactopyranoside 16

Galactopyranoside **S7** (3.77 g, 6.38 mmol, 1.0 equiv.) was dissolved in acetic acid (11 mL), cooled to 10 °C and treated with HBr (30% in acetic acid, 1.4 mL, 7.02 mmol, 1.1 equiv.). The reaction solution was stirred at 10 °C for 30 seconds, filtered and washed with H_2O (10 mL). The filtrate was diluted with H_2O (10 mL) and extracted with DCM (100 mL). The organic phase was washed with small amount of cold H_2O (2 × 25 mL) to remove acetic acid, dried over $MgSO_4$ and the volatiles were removed in vacuo. The crude material was purified via flash column chromatography on silica gel (hexane/EtOAc,

4:1→2:1) affording product **16** (1.77 g, 5.08 mmol, 80%, α:β 1:5) as a white solid. R_f = 0.35 (hexane/EtOAc, 1:1); β anomer : ^{1}H NMR (400 MHz, $CDCl_3$) δ 5.72 (d, $^3J_{H1-H2}$ = 8.3 Hz, 1H, H1), 5.44 (dd, $^3J_{H4-H3}$ = 3.4 Hz, $^3J_{H4-H5}$ = 1.1 Hz, 1H, H4), 5.36 (dd, $^3J_{H2-H3}$ = 10.4 Hz, $^3J_{H2-H1}$ = 8.3 Hz, 1H, H2), 5.12 (dd, $^3J_{H3-H2}$ = 10.4 Hz, $^3J_{H3-H4}$ = 3.4 Hz, 1H, H3), 3.91 (td, $^3J_{H5-H6a}$ = 6.7 Hz, $^3J_{H5-H6b}$ = 6.4 Hz, $^3J_{H5-H4}$ = 1.1 Hz, 1H, H5), 3.74 (dd, $^2J_{H6a-H6b}$ = 11.6, $^3J_{H6a-H5}$ = 6.7 Hz, 1H, H6a), 3.54 (dd, $^2J_{H6b-H6a}$ = 11.6, $^3J_{H6b-H5}$ = 6.4 Hz, 1H, H6b), 2.19 (s, 3H, CH_{3Ac}), 2.12 (s, 3H, CH_{3Ac}), 2.05 (s, 3H, CH_{3Ac}), 2.01 (s, 3H, CH_{3Ac}); ^{13}C NMR (101 MHz, $CDCl_3$) δ 171.1 ($C{=}O_{Ac}$), 170.0 ($C{=}O_{Ac}$), 169.6 ($C{=}O_{Ac}$), 169.2 ($C{=}O_{Ac}$), 92.5 (C1), 74.6 (C4), 71.0 (C2), 68.3 (C3), 67.7 (C5), 60.5 (C6), 20.9 (CH_{3Ac}), 20.8 (CH_{3Ac}), 20.8 (CH_{3Ac}), 20.7 (CH_{3Ac} HRMS (ESI$^+$) *m/z*: [M + Na]$^+$ Calcd for $C_{14}H_{20}NaO_{10}$ 371.0949; Found 371.0946. These data were in agreement with literature.[4]

3.4 Preparation of 1-OH Pyranoses

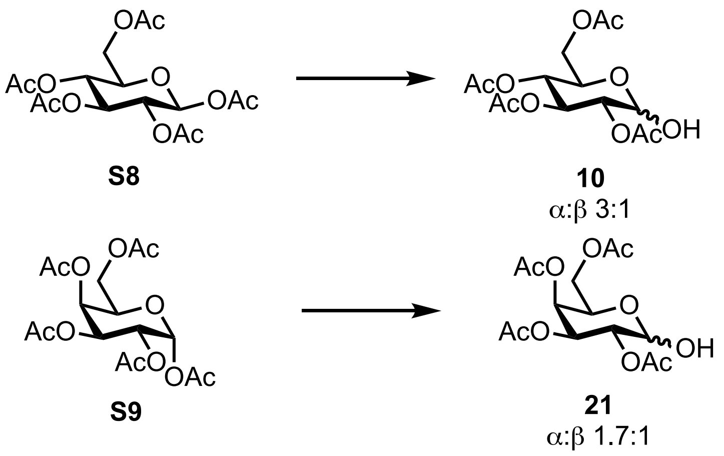

2,3,4,6-Tetra-*O*-acetyl-α/β-D-glucopyranose 10

1,2,3,4,6-Penta-*O*-acetyl-β-D-glucopyranoside **S8** (3.00 g, 7.69 mmol, 1.0 equiv.) was dissolved in THF (190 mL) and treated with ethylenediamine (616 µL, 9.22 mmol, 1.2 equiv.) and acetic acid (615 µL, 10.7 mmol, 1.4 equiv.). The solution was stirred at rt for 16 h and TLC analysis (hexane/EtOAc, 7:3) showed conversion of the starting material to a lower R_f. The reaction was quenched with H_2O (100 mL) and the product extracted with DCM (3 × 100 mL). The organic phases were combined and washed with 1.0 M HCl (100 mL), sat $NaHCO_3$ (100 mL), H_2O (100 mL), dried over $MgSO_4$ and the volatiles were removed *in vacuo*. Purification *via* flash column chromatography on silica gel (hexane/EtOAc, 8:2→1:1) afforded protected pyranose **10** (2.34 g, 6.72 mmol, 87%, α:β

3:1) as a colourless residue. ^{1}H NMR (400 MHz, $CDCl_3$) δ5.54 (dd, $^3J_{H3-H2}$ = 10.3 Hz, $^3J_{H3-H4}$ = 9.5 Hz, 1H, H3), 5.47 (t, $^3J_{H1-H2}$ = 3.7 Hz, 1H, H1), 5.15 – 5.04 (m, 2H, $H4_α$, $H4_β$), 4.93 – 4.86 (ov. dd, $^3J_{H2α-H1α}$ = 3.7 Hz, $^3J_{H2α-H3α}$ = 10.3 Hz, $^3J_{H2β-H1β}$ = 8.4, $^3J_{H2β-H3β}$ = 9.5, 2H, $H2_α$, $H2_β$), 4.34 – 4.21 (m, 3H, $H5_α$, $H6a_α$, $H6a_β$), 4.19 – 4.10 (m, 2H, $H6b_α$, $H6b_β$), 3.33 (br. s, 1H, HO), 2.13 – 1.98 (m, 24H, 3 × $CH_{3Acα}$, 3 × $CH_{3Acβ}$); ^{13}C NMR (101 MHz, $CDCl_3$) δ 171.0 ($C{=}O_{Ac}$), 170.9 ($C{=}O_{Ac}$), 170.34 ($C{=}O_{Ac}$), 170.32 ($C{=}O_{Ac}$), 169.8 ($C{=}O_{Ac}$), 169.7 ($C{=}O_{Ac}$), 90.2 (C1), 71.2 (C2), 70.0 (C3), 68.6 (C4), 67.3 (C5), 62.1 ($C6_α$, $C6_β$), 20.9 ($CH_{3Acα/β}$), 20.8 ($CH_{3Acα/β}$), 20.8 ($CH_{3Acα/β}$), 20.7 ($CH_{3Acα/β}$), 20.7 ($CH_{3Acα/β}$); HRMS (ESI$^+$) *m/z*: $[M + NH_4]^+$ Calcd for $C_{14}H_{24}NO_{10}$ 366.1395; Found 366.1389. These data were in agreement with literature.[5]

2,3,4,6-Tetra-*O*-acetyl-α/β-D-galactopyranose 21

1,2,3,4,6-Penta-*O*-acetyl-β-D-galactopyranoside **S9** (2.00 g, 5.12 mmol, 1.0 equiv.) was dissolved in THF (130 mL) and treated with ethylenediamine (410 µL, 6.15 mmol, 1.2 equiv.) and acetic acid (410 µL, 7.17 mmol, 1.4 equiv.). The solution was stirred at rt for 16 h and TLC analysis (hexane/EtOAc, 7:3) showed conversion of the starting material to a lower R_f. The reaction was quenched with H_2O (70 mL) and the product extracted with DCM (3 × 70 mL). The organic phases were combined and washed with 1.0 M HCl (70 mL), sat $NaHCO_3$ (70 mL), H_2O (70 mL), dried over $MgSO_4$ and the volatiles were removed *in vacuo*. Purification *via* flash column chromatography on silica gel (hexane/EtOAc, 8:2→1:1) afforded pyranose **21** (1.48 g, 4.25 mmol, 83%, α:β = 17:10) as a colourless residue. ^{1}H NMR (400 MHz, $CDCl_3$) δ 5.45 (br. d, $^3J_{H1-H2}$ = 3.6 Hz, 1H, H1), 5.41 (dd, $^3J_{H4-H3}$ = 3.4 Hz, $^3J_{H4-H5}$ = 1.5 Hz, 1H, H4), 5.36 (dd, $^3J_{H3-H2}$ = 10.8 Hz, $^3J_{H3-H4}$ = 3.4 Hz, 1H, $H3_α$, $H4_β$), 5.08 (dd, $^3J_{H2-H3}$ = 10.8 Hz, $^3J_{H2-H1}$ = 3.6 Hz, 1H, H2), 4.17 (s, 1H, OH), 4.42 (td, $^3J_{H5-H6a}$ = 6.7 Hz, $^3J_{H5-H6b}$ = 6.7 Hz, $^3J_{H5-H4}$ = 1.5 Hz, 1H, H5), 4.04 (ov. d, $^3J_{H6-H5}$ = 6.7 Hz, 2H, H6a, H6b), 2.10 (s, 3H, CH_{3Ac}), 2.05 (s, 6H, $CH_{3Acα}$, $CH_{3Acβ}$,), 2.00 (s, 3H, CH_{3Ac}), 1.94 (s, 3H, CH_{3Ac});; ^{13}C NMR (101 MHz, $CDCl_3$) δ 170.8 ($C{=}O_{Ac}$), 170.6 ($C{=}O_{Ac}$), 170.4 ($C{=}O_{Ac}$), 170.3 ($C{=}O_{Ac}$), 90.6 (C1), 61.8 (C6), 68.5 (C2), 68.3 (C4), 67.4 (C3), 66.1 (C5), 20.8 ($CH_{3Acα/β}$), 20.7 $CH_{3Acα/β}$), 20.7 ($CH_{3Acα/β}$), 20.7 ($CH_{3Acα/β}$), 20.6 ($CH_{3Acα/β}$), 20.6 (CH_{3Ac}); HRMS (ESI$^+$) *m/z*: $[M + Na]^+$ Calcd for $C_{14}H_{20}NaO_{10}$ 371.0949; Found 371.0944. These data were in agreement with literature.[6]

S4. Preparation of Glycoconjugates

3'-*O-tert*Butyldimethylsilyl-*N*-4-benzoyl-2'-deoxy-2',2'-difluorocytidine-5'-*O*-hydrogenphosphonate triethylammonium salt 3

Gemcitabine *H*-phosphonate **3** (543 mg, 0.82 mmol, 79%), a white foam, was synthesised following general procedure **C** for the synthesis of *H*-phosphonates from **1** (500 mg, 1.04 mmol, 1.0 equiv.) and purified *via* flash column chromatography on silica gel (Et_2O/DCM, 3:1→0:1, +1% Et_3N, then DCM/MeOH, 100:0→97:3, +1% Et_3N). R_f = 0.49 (DCM/MeOH, 95:5, +1% Et_3N). ^{1}H NMR (400 MHz, $CDCl_3$) δ 8.33 (d, $^3J_{H6-H5}$ = 7.7 Hz, 1H, H6), 7.94 (d, $^3J_{HAr-HAr}$ = 7.3 Hz, 2H, 2 × HAr), 7.58 (app.t, $^3J_{H5-H6}$ = 7.7Hz, $^3J_{HAr-HAr}$ = 7.7 Hz, 2H, HAr, H5), 7.49 (t, $^3J_{HAr-HAr}$ = 7.8 Hz, 2H, 2 × HAr), 6.92 (d, $^1J_{H-P}$ = 625.1 Hz, 1H, H–P), 6.35 (dd, $^3J_{H1'-F}$ = 9.8 Hz, $^3J_{H1'-F}$ = 4.7 Hz, 1H, H1'), 4.44 – 4.26 (m, 2H, H3', H5a'), 4.07 – 3.97 (m, 2H, H4', H5b'), 3.07 (q, $^3J_{CH2-CH3}$ = 7.3 Hz, 6H, 3 × CH_{2Et3N}), 1.34 (t, $^3J_{CH3-CH2}$ = 7.3 Hz, 9H, 3 × CH_{3Et3N}), 0.89 (s, 9H, ^{t}Bu$_{TBS}$), 0.12 (s, 6H, Me$_{TBS}$); ^{13}C NMR (101 MHz, $CDCl_3$) δ 163.1 (C2), 145.6 (C6), 133.3 (C_{Ar}), 129.0 (C_{Ar}), 128.0 (C_{Ar}), 121.9 (t, $^1J_{C2'-F}$ = 261.6 Hz, C2'), 97.3 (C5), 85.0 (dd, $^2J_{C1'-F}$ = 40.5 Hz, $^2J_{C1'-F}$ = 22.5 Hz, C1'), 80.9 (t, $^3J_{C4'-F}$ = 8.3 Hz, $^3J_{C4'-F}$ = 8.3 Hz, C4'), 70.5 (dd, $^2J_{C3'-F}$ = 28.2 Hz, $^2J_{C3'-F}$ = 17.8 Hz, C3'), 60.5 (d, $^2J_{C5'-P}$ = 3.4 Hz, C5'), 45.7 (3 × CH_{2Et3N}), 25.6 (^{t}Bu$_{TBS}$), 18.1 (C_{TBS}), 8.8 (3 × CH_{3Et3N}), -4.7 (Me$_{TBS}$), -5.0 (Me$_{TBS}$); ^{19}F {^{1}H}NMR (376 MHz, $CDCl_3$) δ -115.9 (d, $^2J_{F-F}$ = 239.4 Hz), -117.7 (d, $^2J_{F-F}$ = 239.4 Hz); ^{31}P {^{1}H}NMR (162 MHz, $CDCl_3$) δ 3.89; HRMS (ESI$^+$) *m/z*: $[M + H]^+$ Calcd for $C_{22}H_{31}F_2N_3O_7PSi$ 546.1631; Found 546.1641.

2',3'-Di-*O-tert*butyldimethylsilyl-*N*-4-benzoyl-arabinocytidine-5'-*O*-hydrogenphosphonate triethylammonium salt 4

Cytarabine *H*-phosphonate **4** (500 mg, 0.67 mmol, 97%), a colourless oil, was synthesised following general procedure **C** for the synthesis of *H*-phosphonates from **2** (400 mg, 0.69 mmol, 1.0 equiv.) and purified *via* flash column chromatography on silica gel (Et_2O/DCM, 3:1→0:1, +1% Et_3N, then DCM/MeOH, 100:0→97:3, +1% Et_3N). R_f = 0.55 (DCM/MeOH, 98:2, +1% Et_3N). ^{1}H NMR (400 MHz, $CDCl_3$) δ 8.23 (d, $^3J_{H6-H5}$ = 7.5 Hz, 1H, H6), 7.94 – 7.87 (m, 2H, 2 × HAr), 7.60 (app.tt, $^3J_{HAr-HAr}$ = 7.4 Hz, $^4J_{HAr-HAr}$ = 1.1 Hz, 1H, HAr), 7.55 – 7.43 (m, 3H, 2 × HAr, H5), 6.92 (d, $^1J_{H-P}$ = 617.1 Hz, 1H, H–P), 6.30 (d, $^3J_{H1'-H2'}$ = 3.0 Hz, 1H, H1'), 4.33 (dd, $^3J_{H3'-H1'}$ 3.1Hz, $^3J_{H2'-H3'}$ 1.0 Hz, 1H, H2'), 4.26 – 4.14 (m, 2H, H4', H5'a), 4.06 (d, $^3J_{H3'-H2'}$ 0.9 Hz, 1H, H3'), 4.04 – 3.95 (m, 1H, H5'b), 2.92 (q, $^3J_{CH2-CH3}$ = 7.3 Hz, 6H, 3

× CH_{2Et3N}), 1.25 (t, $^{3}J_{CH3-CH2}$ = 7.3 Hz, 9H, 3 × CH_{3Et3N}), 0.90 (s, 9H, $^{t}Bu_{TBS}$), 0.78 (s, 9H, $^{t}Bu_{TBS}$), 0.13 (s, 3H, Me_{TBS}), 0.12 (s, 3H, Me_{TBS}), 0.03 (s, 3H, Me_{TBS}), –0.17 (s, 3H, Me_{TBS}); 13C NMR (101 MHz, $CDCl_3$) δ 162.1 (C4/C2), 147.6 (C6), 133.2 (C_{Ar}), 129.1 (C_{Ar}), 127.7 (C_{Ar}), 89.3 (C1'), 87.2 (d, $^{3}J_{C4'-P}$ = 6.6 Hz, C4'), 79.0 (C3'), 76.3 (C2'), 63.8 (d, $^{2}J_{C5'-P}$ = 4.5 Hz, C5'), 45.8 (3 × CH_{2Et3N}), 25.9 ($^{t}Bu_{TBS}$), 25.8 ($^{t}Bu_{TBS}$), 18.0 (C_{TBS}), 17.9 (C_{TBS}), 9.5 (3 × CH_{3Et3N}), –4.5 (Me_{TBS}), –4.5 (Me_{TBS}), –5.0 (Me_{TBS}), –5.3 (Me_{TBS}); ^{31}P NMR (162 MHz, $CDCl_3$) δ 4.26 (dt, $^{1}J_{P-H}$ = 617.2, $^{3}J_{P-H5'a}$ = 8.2 Hz, $^{3}J_{P-H5'b}$ = 8.2 Hz); HRMS (ESI$^+$) *m/z*: $[M + H]^+$ Calcd for $C_{28}H_{47}N_3O_8PSi_2$ 640.2634; Found 640.2637.

2',3'-Di-*O*-*tert*butyldimethylsilyl-*N*-4-benzoyl-arabinocytidine-5'-*O*-[6''-*O*-(1'',2'',3'',4''-tetra-*O*-actetyl-β-D-glucopyranose)]-hydrogenphosphonate

Cytarabine derivative **4** (137 mg, 0.18 mmol, 1.0 equiv.) and glucopyranose **5** (97 mg, 0.28 mmol, 1.5 equiv.), were dissolved in pyridine/MeCN (12 mL). The solution was cooled to 0 °C, treated dropwise with PivCl (68 μL, 0.55 mmol, 3.0 equiv.) and stirred at rt for 1.5 h. A further dropwise addition of PivCl was added (34 μL, 0.28 mmol, 1.5 equiv.) and the solution was stirred for a further 30 min. The reaction was monitored *via* TLC analysis (DCM/MeOH, 98:2, +1% Et_3N). Upon complete consumption of the stating material to a higher R_f, the reaction was quenched with sat. aq. $NaHCO_3$ (12 mL). The aqueous phase was extracted with DCM (3 × 12 mL), and the combined organic phases were dried over $MgSO_4$. The volatiles were removed *in vacuo*, and co-evaporated twice toluene (3 × 10 mL). The crude residue was purified on a silica gel column (Et_2O/DCM, 3:1→0:1, +1% Et_3N, then DCM/MeOH, 100:0→97:3, +1% Et_3N) to afford the title compound (153 mg, 0.16 mmol, 84%, α:β 9:11) as a white foam. **Observed for both diastereoisomers**: ^{1}H NMR (400 MHz, $CDCl_3$): δ 8.72 (br. s, 2H, $NH_{(a)}$, $NH_{(b)}$), 7.90 (s, 4H, 2 × $HAr_{(a)}$, 2 × $HAr_{(b)}$), 7.64 – 7.55 (m, 2H, $HAr_{(a)}$, $HAr_{(b)}$), 7.54 – 7.48 (m, 6H, 2 × $HAr_{(a)}$, 2 × $HAr_{(b)}$, $H5_{(a)}$, $H5_{(b)}$), 5.26 (ov. t, $^{3}J_{H3''-H2''}$ = 9.4 Hz, $^{3}J_{H3''-H4''}$ = 9.4 Hz, 1H, $H3''_{(a)}$, $H3''_{(b)}$), 4.51 – 4.36 (m, 3H, $H2''a_{(a)}$, $H2''a_{(b)}$, $H4''_{(b)}$), 4.33 (s, 2H, $H3'_{(a)}$, $H3'_{(b)}$), 4.32 – 4.07 (m, 8H, $H5'/6''b_{(a)}$, $H5'/6''b_{(b)}$, 2 × $H5'/6''_{(a)}$, 2 × $H5'/6''_{(b)}$, $H4'_{(a)}$, $H4'_{(b)}$), 3.92 – 3.82 (m, 2H, $H5''_{(a)}$, $H5''_{(b)}$), 0.05 (s, 6H, $Me_{TBS(a)}$, $Me_{TBS(b)}$), –0.17 (s, 6H, $Me_{TBS(a)}$, $Me_{TBS(b)}$); **diastereoisomer α**: ^{1}H NMR (400 MHz, $CDCl_3$) δ 8.23 (d, $^{3}J_{H6-H5}$ = 7.5 Hz, 1H, H6), 6.95 (d, $^{1}J_{H-P}$ = 722.8 Hz, 1H, H–P), 6.34 (d, $^{3}J_{H1'-H2'}$ = 2.8 Hz, 1H, H1'), 5.73 (d, $^{3}J_{H1''-H2''}$ = 8.3 Hz, 1H, H1''), 5.05 (dd, $^{3}J_{H6-H5}$ = 10.1 Hz, $^{3}J_{H4''-H3''}$ = 9.4 Hz, 1H, H4''), 4.05 (s, 1H, H3'), 2.11 (s, 3H, CH_{3Ac}), 2.06 (s, 3H, CH_{3Ac}), 2.03 (s, 3H, CH_{3Ac}), 2.02 (s,

3H, CH_{3Ac}), 0.92 (s, 9H, $^{t}Bu_{TBS}$), 0.81 (s, 9H, $^{t}Bu_{TBS}$), 0.14 (s, 3H, Me_{TBS}), 0.13 (s, 3H, Me_{TBS}); **diastereoisomer β**: ^{1}H NMR (400 MHz, $CDCl_3$) δ 8.19 (d, $^{3}J_{H6\text{-}H5}$ = 7.5 Hz, 1H, H6), 6.88 (d, $^{1}J_{H\text{-}P}$ = 728.6 Hz, 1H, H–P), 6.32 (d, $^{3}J_{H1'\text{-}H2'}$ = 2.8 Hz, 1H, H1'), 5.69 (d, $^{3}J_{H1''\text{-}H2''}$ = 8.3 Hz, 1H, H1''), 4.07 (s, 1H, H3'), 2.11 (s, 3H, CH_{3Ac}), 2.07 (s, 3H, CH_{3Ac}), 2.04 (s, 3H, CH_{3Ac}), 2.01 (s, 3H, CH_{3Ac}), 0.92 (s, 9H, $^{t}Bu_{TBS}$), 0.80 (s, 9H, $^{t}Bu_{TBS}$), 0.14 (s, 3H, Me_{TBS}), 0.13 (s, 3H, Me_{TBS}); **Observed for both diastereoisomers**: ^{13}C NMR (101 MHz, $CDCl_3$) δ 170.1 (C=O_{Ac}), 170.1 (C=O_{Ac}), 169.5 (C=O_{Ac}), 169.3 (C=O_{Ac}), 169.2 (C=O_{Ac}), 169.2 (C=O_{Ac}), 169.0 (C=O_{Ac}), 168.9 (C=O_{Ac}), 162.1 (C4/C2), 133.1 (C_{Ar}), 129.0 (C_{Ar}), 127.6 (C_{Ar}), 91.7 (C1''$_{(a/b)}$), 91.6 (C1''$_{(a/b)}$), 89.1 (2 × C1'), 86.0 – 85.5 (m, 2 × C4'), 78.6 (C3'$_{(a/b)}$), 78.4 (C3'$_{(a/b)}$), 75.8 (C2'$_{(a/b)}$), 73.5 (d, $^{3}J_{C5''\text{-}P}$ = 5.2 Hz, C5''$_{(a/b)}$), 73.1 (d, $^{3}J_{C5''\text{-}P}$ = 6.3 Hz,, C5''$_{(a/b)}$), 72.7 (C3''$_{(a/b)}$), 72.6 (C3''$_{(a/b)}$), 70.1(C2''$_{(a/b)}$), 70.1 (C2''$_{(a/b)}$), 67.8 (C4''$_{(a)}$), 67.6 (C4''$_{(b)}$), 65.1 – 63.2 (ov. m, C5', C6''), 25.7 ($^{t}Bu_{TBS}$), 25.7 ($^{t}Bu_{TBS}$), 25.7 ($^{t}Bu_{TBS}$), 20.8 (CH_{3Ac}), 20.8, (CH_{3Ac}) 20.6 (CH_{3Ac}), 20.6 – 20.5 (5 × CH_{3Ac}), 17.9 (C_{TBS}), 17.7 (C_{TBS}), –4.6 (Me_{TBS}), –4.7 (Me_{TBS}), –5.2 (Me_{TBS}), –5.2 (Me_{TBS}), –5.4 (Me_{TBS}), –5.4 (Me_{TBS}); **diastereoisomer α**: ^{31}P CPD NMR (162 MHz, $CDCl_3$) δ 8.90; **diastereoisomer β**: ^{31}P CPD NMR (162 MHz, $CDCl_3$) δ 9.14; HRMS (ESI^{+}) *m/z*: $[M + H]^{+}$ Calcd for $C_{42}H_{65}N_3O_{17}PSi_2$ 970.3590; Found 970.3586.

2',3'-Di-*O*-*tert*butyldimethylsilyl-*N*-4-benzoyl-arabinocytidine-5'-*O*-[6''-*O*-(1'',2'',3'',4''-tetra-*O*-acetyl-β-D-glucopyranose)]-phosphate triethylammonium salt 6

2',3'-Di-*O*-*tert*butyldimethylsilyl-*N*-4-benzoyl-arabinocytidine-5'-*O*-[6''-*O*-(1'',2'',3'',4''-tetra-*O*-actetyl-β-D-glucopyranose)]-hydrogenphosphonate (153 mg, 0.16 mmol, 1.0 equiv.) was dissolved in pyridine (3.0 mL) and treated with a solution of I_2 (60 mg, 0.24 mmol, 1.5 equiv.) in pyridine and H_2O (3.0 mL, 2:1). The solution was and stirred at rt for 40 min. The solution was diluted with DCM, quenched with 1 M $Na_2S_2O_3$ (15 mL) and extracted with DCM (3 × 15 mL). The organic phases were combined, dried over $MgSO_4$, the volatiles were removed *in vacuo*, and co-evaporated toluene (2 × 10 mL). The residue was purified *via* flash column chromatography on silica gel (DCM/MeOH, 1:0→9:1, +1% Et_3N) afforded product **6** (163 mg, 0.15 mmol, 95%) as a white foam. R_f = 0.30 (DCM/MeOH, 98:2, +1% Et_3N). ^{1}H NMR (400 MHz, $CDCl_3$) δ 8.25 (d, $^{3}J_{H6\text{-}H5}$ = 7.5 Hz, 1H, H6), 7.91 – 7.84 (m, 2H, 2 × HAr), 7.60 – 7.51 (m, 1H, HAr), 7.50 – 7.41 (m, 3H, 2 × HAr, H5), 6.24 (d, $^{3}J_{H1'\text{-}H2'}$ = 3.1 Hz, 1H, H1'), 5.66 (d, $^{3}J_{H1''\text{-}H2''}$ = 8.2 Hz, 1H, H1''), 5.18 (t, $^{3}J_{H3''\text{-}H2''}$ =

9.4 Hz, $^{3}J_{H3''-H4''}$ = 9.4 Hz, 1H, H3''), 5.07 – 4.98 (m, 2H, H2'', H4''), 4.26 (d, $^{3}J_{H2'-H1'}$ = 3.1, 1.0 Hz, 1H, H2'), 4.20 – 4.08 (m, 2H, H4', H5'/6''a), 4.02 (s, 1H, H3'), 3.98 (ddd, $^{2}J_{Ha'-Hb'}$ = 11.6 Hz, 5.4 Hz, 2.5 Hz, 1H, H5'/6''), 3.97 – 3.88 (m, 1H, H5'/6''), 3.87 (dd, $^{2}J_{Ha'-Hb'}$ = 11.6 Hz, $^{3}J_{H5'/6''-P}$ = 6.7 Hz, 1H, H5'/6''b), 3.80 (ddd, $^{3}J_{H5''-4H''}$ = 10.0 Hz, $^{3}J_{H5-H6b}$ = 5.6 Hz, $^{3}J_{H5-H6a}$ = 2.4 Hz, 1H, H5''),3.04 (q, $^{3}J_{CH2-CH3}$ = 7.3 Hz, 6H, 3 × CH_{2Et3N}), 2.03 (s, 3H, CH_{3Ac}), 1.99 (s, 3H, CH_{3Ac}), 1.97 (s, 3H, CH_{3Ac}), 1.94 (s, 3H, CH_{3Ac}), 1.30 (t, $^{3}J_{CH3-CH2}$ = 7.3 Hz, 9H, 3 × CH_{3Et3N}), 0.86 (s, 9H, $^{t}Bu_{TBS}$), 0.73 (s, 9H, $^{t}Bu_{TBS}$), 0.09 (s, 6H, 2 × Me_{TBS}), –0.01 (s, 3H, Me_{TBS}), –0.21 (s, 3H, Me_{TBS}); ^{13}C NMR (101 MHz, $CDCl_3$) δ 170.2 (C=O_{Ac}), 169.6 (C=O_{Ac}), 169.3 (C=O_{Ac}), 168.9 (C=O_{Ac}), 162.1 (C4/C2), 147.8 (C6), 133.1 (C_{Ar}), 129.0 (C_{Ar}), 127.7 (C_{Ar}), 95.5 (C5), 91.7 (C1''), 89.1 (C1'), 87.0 (d, $^{3}J_{C4'-P}$ = 6.7 Hz, C4'), 78.8 (C3'), 76.2 (C2'), 74.2 (d, $^{3}J_{C5'-P}$ = 8.1 Hz, C5'), 73.1 (C3''), 70.4 (C2''), 68.3 (C4''), 65.2 (d, $^{2}J_{C5/6''-P}$ = 5.8 Hz, C5'/6''), 63.7 (d, $^{2}J_{C5/6''-P}$ = 5.1 Hz, C5'/6''), 45.7 (3 × CH_{2Et3N}), 25.8 ($^{t}Bu_{TBS}$), 25.7 ($^{t}Bu_{TBS}$), 20.8 (CH_{3Ac}), 20.7 (CH_{3Ac}), 20.6 (CH_{3Ac}), 20.6 (CH_{3Ac}), 17.9 (C_{TBS}), 17.8 (C_{TBS}), 8.6 (3 × CH_{3Et3N}), –4.6 (2 × Me_{TBS}), –5.1 (Me_{TBS}), –5.4 (Me_{TBS}); ^{31}P NMR (162 MHz, $CDCl_3$) δ –0.27 (app. p, $^{3}J_{P-H5'a/6''a}$ = 6.7 Hz, $^{3}J_{P-H5'b/6''b}$ = 6.7 Hz, $^{3}J_{P-H5'a/6''a}$ = 6.4 Hz, $^{3}J_{P-H5'b/6''b}$ = 6.4 Hz); HRMS (ESI^{+}) *m/z*: [M + H]$^{+}$ Calcd for $C_{42}H_{65}N_3O_{18}PSi_2$ 986.3534; Found 986.3524.

3'-*O*-*tert*Butyldimethylsilyl-*N*-4-benzoyl-2'-deoxy-2',2'-difluorocytidine-5'-*O*-[6''-*O*-(1'',2'',3'',4''-tetra-*O*-acetyl-β-D-glucopyranose)]-phosphate triethylammonium salt 7

Phosphate **7** (190 mg, 0.19 mmol, 58%), a white foam, was synthesised following general procedure **D** from **3** (212 mg, 0.33 mmol, 1.0 equiv.) and glucopyranose **5** (171 mg, 0.49 mmol, 1.5 equiv.) and purified *via* flash column chromatography on silica gel (DCM/MeOH, 100:0→95:5, +1% Et_3N). R_f = 0.25 (DCM/MeOH, 98:2, +1% Et_3N). ^{1}H NMR (400 MHz, $CDCl_3$) δ 8.31 (d, $^{3}J_{H6-H5}$ = 7.7 Hz, 1H, H6), 8.00 – 7.85 (m, 2H, 2 × HAr), 7.64 – 7.56 (m, 1H, HAr), 7.49 (app. tt, $^{3}J_{HAr-HAr}$ = 7.7 Hz, $^{4}J_{HAr-HAr}$ = 1.5 Hz, 3H, 2 × Har, H5), 6.36 (dd, $^{3}J_{H1'-F}$ = 9.7 Hz, $^{3}J_{H1'-F}$ =5.4 Hz, 1H, H1'), 5.69 (d, $^{3}J_{H1''-H2''}$ = 8.2 Hz, 1H, H1''), 5.16 (t, $^{3}J_{H3''-H2''}$ = 9.4 Hz, $^{3}J_{H3''-H4''}$ = 9.4 Hz 1H, H3''), 5.08 – 5.01 (m, 2H, H2'', H4''), 4.53 – 4.34 (m, 1H, H3'), 4.34 – 4.27 (m, 1H, H5'a/6''a), 4.06 – 3.97 (m, 3H, H4', H5'b/H6''a, H5'a/H6''b), 3.92 (ddd, $^{2}J_{Ha'-Hb'}$ = 11.4, 7.8, 5.7 Hz, 1H, H5'b/6''b), 3.85 (ddd, $^{3}J_{H5''-H4''}$ = 10.0, $^{3}J_{H5''-H6''b}$ = 5.6, $^{3}J_{H5''-H6''a}$ = 2.4 Hz, 1H, H5''), 3.01 (q, $^{3}J_{CH2-CH3}$ = 7.3 Hz, 6H, 3 × CH_{2Et3N}), 2.04 (s, 3H, CH_{3Ac}), 2.03 (s, 2H, CH_{3Ac}), 1.99 (s, 3H, CH_{3Ac}), 1.94 (s, 3H, CH_{3Ac}), 1.30 (t, $^{3}J_{CH3-CH2}$ = 7.3 Hz, 9H, 3 ×

CH_{3Et3N}, 0.89 (s, 9H, $^{t}Bu_{TBS}$), 0.13 (s, 3H, 2 × Me_{TBS}), 0.12 (s, 3H, 2 × Me_{TBS}); ^{13}C NMR (101 MHz, $CDCl_3$) δ 170.1 ($C{=}O_{Ac}$), 169.7 ($C{=}O_{Ac}$), 169.4 ($C{=}O_{Ac}$), 169.0 ($C{=}O_{Ac}$), 162.7 (C4/C2), 145.5 (C6), 133.2 (C_{Ar}), 129.1 (C_{Ar}), 127.8 (C_{Ar}), 122.0 (C2'), 97.4 (C5), 91.7 (C1''), 85.0 (dd, $^{3}J_{C1'-F}$ = 41.0 Hz, $^{3}J_{C1'-F}$ = 23.3 Hz, C1'), 81.2 (C4'), 74.0 (d, $^{3}J_{C5''-P}$ = 7.7 Hz, C5''), 73.1 (C3''), 70.5 (C2''), 70.5 (dd, $^{3}J_{C3'-F}$ = 28.5 Hz, $^{3}J_{C3'-F}$ = 17.9 Hz, C3'), 68.3 (C4''), 63.9 (d, $^{2}J_{C5'/6''-P}$ = 4.8 Hz, C5'/6''), 62.2 (d, $^{2}J_{C5'/6''-P}$ = 5.1 Hz, C5'/6''), 45.8 (3 × CH_{2Et3N}), 25.6 ($^{t}Bu_{TBS}$), 20.9 (CH_{3Ac}), 20.8 (CH_{3Ac}), 20.68 (CH_{3Ac}), 20.66 (CH_{3Ac}), 18.1 (C_{TBS}), 9.0 (3 × CH_{3Et3N}), –4.8 (Me_{TBS}), –4.9 (Me_{TBS}); ^{19}F NMR (377 MHz, $CDCl_3$) δ –115.7 (ddd, $^{2}J_{F-F}$ = 239.3 Hz, $^{3}J_{F-H3'}$ = 12.6 Hz, $^{3}J_{F-H1'}$ = 5.6 Hz, F), –117.8 (dt, $^{2}J_{F-F}$ = 239.3, $^{3}J_{F-H1'}$ = 9.5 Hz, F); ^{31}P {^{1}H} NMR (162 MHz, $CDCl_3$) δ –0.78; HRMS (ESI^{+}) *m/z*: $[M + H]^{+}$ Calcd for $C_{36}H_{49}F_2N_3O_{17}PSi$ 892.2531; Found 892.2515.

Arabinocytidine-5'-*O*-(6''-*O*-α/β-D-glucopyranose)-phosphate sodium salt 8

Phosphate **8** (17 mg, 33 μmol, 26%, α:β 2:3), a white foam, was synthesised following general procedure **E** then **F** from **6** (191 mg, 0.18 mmol, 1.0 equiv.) and purified *via* HILIC-B HPLC, Polaris 5, 250 × 21.2 mm; flow rate: 20.0 mL/min; retention time: 20.81 min; following method:

**Time (min.)**	**%A (H_2O, 0.1% formic**	**%B (Acetonitrile)**
0.0	5	95
5.0	5	95
7.0	60	40
25.0	95	5
30.0	95	5
31.0	5	95
41.0	5	95

Observed for both diastereoisomers: ^{1}H NMR (400 MHz, D_2O) δ 4.27 – 4.09 (m, 11H, $H3'_{\alpha}$, $H3'_{\beta}$, $H4'_{\alpha}$, $H4'_{\beta}$, $H5'a_{\alpha}$, $H5a'_{\beta}$, $H5'b_{\alpha}$, $H5b'_{\beta}$, $H6''a_{\alpha}$, $H6''b_{\alpha}$, $H6''a_{\beta}$), 3.52 – 3.42 (m, 3H, $H3''_{\alpha}$ $H4''_{\alpha}$, $H4''_{\beta}$); **diastereoisomer α**: ^{1}H NMR (400 MHz, D_2O) δ 8.02 (d, $^{3}J_{H6-H5}$ = 7.7 Hz, 1H, H6), 6.24 (d, $^{3}J_{H1'-H2'}$ = 5.5 Hz, 1H, H1'), 6.19 (d, $^{3}J_{H6-H5}$ = 7.7 Hz, 1H, H5),5.22 (d, $^{3}J_{H1''-H2''}$ = 3.8 Hz, 1H, H1''), 4.49 (t, $^{3}J_{H2'-H1'}$ = 5.4 Hz, $^{3}J_{H2'-H3'}$ = 5.4 Hz, 1H, H2'), 3.94 (dtd, $^{3}J_{H5''-H4''}$ = 10.1 Hz, $^{3}J_{H5''-H6''a}$ = 3.3 Hz, $^{3}J_{H5''-P}$ = 3.3 Hz, $^{3}J_{H5''-H6''b}$ = 1.4 Hz, 1H, H5''), 3.72 (t, $^{3}J_{H3'-H2'}$ = 9.5 Hz, $^{3}J_{H3'-H4'}$ = 9.5 Hz, 1H, H3''), 3.54 (dd, $^{3}J_{H2''-H3''}$ = 9.8 Hz, $^{3}J_{H2''-H3''}$ = 3.8 Hz, 1H, H2''); **diastereoisomer β**: ^{1}H NMR (400 MHz, D_2O) δ 8.03 (d, $^{3}J_{H6-H5}$ = 7.7 Hz, 1H, H6), 6.25 (d, $^{3}J_{H1'-H2'}$ = 5.5 Hz, 1H, H1'), 6.21 (d, $^{3}J_{H6-H5}$ = 7.7 Hz, 1H, H5),4.66 (d, $^{3}J_{H1''-H2''}$ = 7.9 Hz, 1H, H1''), 4.49 (t, $^{3}J_{H2'-H1'}$ = 5.4 Hz, $^{3}J_{H2'-H3'}$ = 5.4 Hz, 1H, H2'), 4.06 (ddd, $^{2}J_{H6''b-H6''a}$ = 11.6 Hz, $^{3}J_{H6''b-H5''/P}$ =

6.6 Hz, $^{3}J_{H6''b-H5''/P}$ = 5.1 Hz, 1H, H6''b), 3.61 – 3.56 (m, 1H, H5''), 3.26 (app dd, $^{3}J_{H2''-H3''}$ = 9.5 Hz, $^{3}J_{H2''-H1''}$ = 7.9 Hz, 1H); **Observed for both diastereoisomers**: ^{13}C NMR (101 MHz, D_2O) δ 144.1 (C6), 81.3 (ov. d, $^{3}J_{C4'-P}$ = 8.4 Hz, C4'), 75.4 (C2'), 64.6 (ov. d, $^{2}J_{C6''-P}$ = 5.3 Hz), 63.7 (ov. d, $^{2}J_{C5'-P}$ = 5.3 Hz); **diastereoisomer α**: ^{13}C NMR (101 MHz, D_2O) δ 162.2 (C2), 152.3 (C4), 94.9 (C5), 92.1 (C1''), 85.6 (C1), 73.9 (C3'), 72.6 (C3''), 71.4 (C2''), 70.4 (d, $^{3}J_{C5''-P}$ = 7.7 Hz, C5''), 69.3 (C4); **diastereoisomer β**: ^{13}C NMR (101 MHz, D_2O) δ 162.1 (C2), 152.2 (C4), 96.0 (C1''), 95.0 (C5), 85.5 (C1), 75.6 (C3''), 74.6 (d, $^{3}J_{C5''-P}$ = 7.7 Hz, C5''), 74.1 (C2''), 73.8 (C3'), 69.2 (C4''), **observed for both diastereoisomers**: ^{31}P NMR (162 MHz, D_2O) δ 0.44 (ov. s, P_α, P_β); HRMS (ESI$^-$) *m/z*: [M − H]$^-$ Calcd for $C_{15}H_{23}N_3O_{13}P$ 484.0974; Found 484.0984.

2’-Deoxy-2’,2’-difluorocytidine-5'-*O*-(6''-*O*-β-D-glucopyranose)-phosphate sodium salt 9

Phosphate **9** (14 mg, 28.8 µmol, 17 %, α:β 2:3), a white foam, was synthesised following general procedure **E** then **F** from **7** (169 mg, 0.17 mmol, 1.0 equiv.) and purified *via* HILIC-B HPLC, Polaris 5, 250 × 21.2 mm; flow rate: 20.0 mL/min; retention time: 20.70 min; following method:

**Time (min.)**	**%A (H_2O, 0.1% formic**	**%B (Acetonitrile)**
0.0	5	95
5.0	5	95
7.0	80	20
25.0	95	5
30.0	95	5
31.0	5	95
41.0	5	95

Observed for both diastereoisomers: ^{1}H NMR (400 MHz, D_2O) δ 8.09 (d, $^{3}J_{H6-H5}$ = 8.1 Hz, 2H, $H6_\alpha$, $H6_\beta$), 6.30 – 6.22 (m, 2H, $H1'_\alpha$, $H1'_\beta$), 4.52 (app. tt, $^{3}J_{H3'-F}$ = 11.9, $^{3}J_{H3'-H4'}$ = 8.1 Hz, 2H, $H3'_\alpha$, $H3'_\beta$), 4.42 – 4.33 (m, 2H, $H5'a_\alpha$, $H5'a_\beta$), 4.29 – 4.19 (m, 4H $H4'_\alpha$, $H4'_\beta$, $H5'b_\alpha$, $H5'b_\beta$), 3.81 – 3.71 (m, $5H_\alpha$, H5'', $H6''a_\alpha$, $H6''a_\beta$, $H6''b_\alpha$, $H6''b_\beta$); **diastereoisomer α**: ^{1}H NMR (400 MHz, D_2O) δ 6.32 (d, $^{3}J_{H5-H6}$ = 8.1 Hz, 1H, H5), 5.56 (dd, $^{3}J_{H1''-P}$ = 7.2 Hz, $^{3}J_{H1''-H2''}$ = 3.4 Hz, 1H, H1''), 4.03 (dd, $^{3}J_{H4''-H3''}$ = 3.1 Hz, $^{3}J_{H4''-H5''}$ = 1.2 Hz, 1H), 3.91 (dd, $^{3}J_{H3''-H2''}$ = 10.3, $^{3}J_{H3''-H4''}$ = 3.1 Hz, 1H, H4''), 3.85 (dt, $^{3}J_{H2''-H3''}$ = 10.4 Hz, $^{3}J_{H2''-H1''}$ = 3.5 Hz, $^{3}J_{H2''-P}$ = 3.5 Hz, 1H, H2''); **diastereoisomer β**: ^{1}H NMR (400 MHz, D_2O) δ 6.33 (d, $^{3}J_{H5-H6}$ = 7.9 Hz, 1H, H5), 4.88 (t, $^{3}J_{H1''-P}$ = 7.5 Hz, $^{3}J_{H1''-H2''}$ = 7.5 Hz, 1H, H1''), 4.12 (t, $^{3}J_{H5''-H6''a}$ = 6.0 Hz, $^{3}J_{H5''-H6''a}$ = 6.0 Hz, 1H,

H5''), 3.93 (d, $^3J_{H4''-H3''}$ = 3.3 Hz, 1H, H4''), 3.69 (dd, $^3J_{H3''-H2''}$ = 10.0 Hz, $^3J_{H3''-H4''}$ = 3.3 Hz, 1H, H3''), 3.59 (dd, $^3J_{H2''-H3''}$ = 10.0 Hz, $^3J_{H2''-H1''}$ = 7.7 Hz, 1H, H2''); **Observed for both diastereoisomers**: ^{13}C NMR (101 MHz, D_2O) δ 159.5 (C2), 148.4 (C4), 143.6 (C6), 125.0 – 118.8 (m, C2'), 95.7 (C5), 85.2 – 83.9 (m, C1'), 80.1 – 79.3 (m, C4'), 68.4 – 68.1 (m, C3'), 62.7 (ov. d, $^2J_{C5'-P}$ = 5.1 Hz); **diastereoisomer α**: ^{13}C NMR (101 MHz, D_2O) δ 95.7 (d, $^2J_{C1''-P}$ = 3.3 Hz, C1''), 75.7 (C5''), 69.1 (C4''), 69.0 (C3''), 68.1 (d, $^3J_{C2''-P}$ = 8.1 Hz, C2''), 61.0 (C6); **diastereoisomer β**: ^{13}C NMR (101 MHz, D_2O) δ 98.4 (d, $^2J_{C1''-P}$ = 6.2 Hz, C1''), 72.3 (d, $^4J_{C3''-P}$ = 1.7 Hz, C3''), 72.0 C5''), 71.2 (d, $^3J_{C2''-P}$ = 8.4 Hz, C2''), 68.5 (C4''), 61.0 (C6); **diastereoisomer α**:^{31}P {^{1}H} NMR (162 MHz, D_2O) δ –1.46; **diastereoisomer β**: ^{31}P {^{1}H} NMR (162 MHz, D_2O) δ –1.70; **diastereoisomer α**: ^{19}F {^{1}H} NMR (377 MHz, D_2O) δ –117.7 (d, $^2J_{F-F}$ = 241.0 Hz), –118.7 (d, $^2J_{F-F}$ = 242.7 Hz); **diastereoisomer β**: ^{19}F {^{1}H} NMR (377 MHz, D_2O) δ –117.8 (d, $^2J_{F-F}$ = 241.0 Hz), –118.7 (d, $^2J_{F-F}$ = 241.1 Hz); HRMS (ESI$^-$) *m/z*: [M + H]$^+$ Calcd for $C_{15}H_{21}F_2N_3O_{12}P$ 504.0836; Found 504.0841.

2,3,4,6-Tetra-*O*-acetyl-β-D-glucopyranosyl-1-*O*-hydrogenphosphonate triethylammonium salt 11

2,3,4,6-Tetra-*O*-acetyl-D-glucopyranose **10** (500 mg, 1.44 mol, 1.0 equiv.) was dissolved in DCM, cooled to 0 °C, treated dropwise with DBU (64 μL, 0.43 mol, 0.3 equiv.) and trichloroacetonitrile (432 μL, 4.31 mol, 3.0 equiv.). The reaction was stirred at 0 °C and monitored by TLC analysis (DCM, +1% Et_3N) showing conversion of the starting material to a higher R_f after 2h. The volatiles were removed *in vacuo* and the crude material was passed through a silica plug (Et_2O, +1% Et_3N) to give the trichloroacetamidate intermediate (570 mg, 1.16 mmol, 80%) as a brown foam. The intermediate was dissolved in THF (14 mL), and treated with a H_3PO_3 solution (570 mg, 6.94 mmol, 6 equiv.) in THF (14 mL). The reaction solution was stirred at RT for 5 min, cooled to 0˚C and treated with triethylamine (2.3 mL, 16.2 mmol, 14 equiv.). The solution was stirred at 0˚C for 2 h. The precipitate (triethylammonium hydrogenphosphonate) was filtered off, washed with cold THF, the filtrate was recovered and the volatiles were removed *in vacuo*. The crude product was purified via flash silica column chromatography (DCM: MeOH, 100:0→94:6, +1% Et_3N) to give the product **11** as a beige hygroscopic powder (241 mg, 0.47 mmol, 40%, 32% over two steps, α:β 1:10). R_f = 0.51 (DCM/MeOH, 95:5, +1% Et_3N). β anomer: ^{1}H NMR (400 MHz, $CDCl_3$) δ 6.71 (d, $^1J_{H-P}$ = 639.8 Hz, 1H, H–P), 5.08 (dd,

$^3J_{H1-P}$ = 9.4 Hz, $^3J_{H1-H2}$ = 8.0 Hz, 1H, H1), 4.98 (t, $^3J_{H3-H2}$ = 9.6 Hz, $^3J_{H3-H4}$ = 9.6 Hz, 1H, H3), 4.88 (t, $^3J_{H4-H3}$ = 9.6 Hz, $^3J_{H4-H5}$ = 9.7 Hz, 1H, H4), 4.78 (dd, $^3J_{H2-H3}$ = 9.6 Hz, $^3J_{H2-H1}$ = 8.0 Hz, 1H, H2), 4.04 (dd, $^2J_{H6a-H6b}$ = 12.4 Hz, $^3J_{H6a-H5}$ = 4.4 Hz, 1H, H6a), 3.89 (dd, $^2J_{H6b-H6a}$ = 12.4 Hz, $^3J_{H6b-H5}$ = 2.3 Hz, 1H, H6b), 3.58 (ddd, $^3J_{H5-H4}$ = 9.7 Hz, $^3J_{H5-H6b}$ = 4.4 Hz, $^3J_{H5-H6a}$ = 2.4 Hz, 1H, H5), 2.85 (q, $^3J_{CH2-CH3}$ = 7.3 Hz, 6H, 3 × CH_{2Et3N}), 1.85 (s, 3H, CH_{3Ac}), 1.82 (s, 3H, CH_{3Ac}), 1.81 (s, 3H, CH_{3Ac}), 1.78 (s, 3H, CH_{3Ac}), 1.11 (t, $^3J_{CH3-CH2}$ = 7.3 Hz, 9H, 3 × CH_{3Et3N}); ^{13}C NMR (101 MHz, $CDCl_3$) δ 170.2 (C=O_{Ac}), 169.7 (C=O_{Ac}), 169.19 (C=O_{Ac}), 169.17 (C=O_{Ac}), 94.7 (d, $^2J_{HC1-P}$ = 4.4 Hz, C1), 72.5 (C3), 71.6 (C2, C5), 67.8 (C4), 61.4 (C6), 45.3 (3 × CH_{2Et3N}), 20.4 (2 × CH_{3Ac}), 20.30 (CH_{3Ac}), 20.28 (CH_{3Ac}), 8.2 (3 × CH_{3Et3N}); ^{31}P NMR (162 MHz, $CDCl_3$) δ 0.54 (dd, $^1J_{P-H}$ = 640.2 Hz, $^3J_{P-H1}$ = 9.4 Hz; MS (ESI$^-$) *m/z*: [M − H]$^-$ Calcd for $C_{14}H_{20}O_{12}P$ 411.1; Found 411.2. These data were in agreement with literature.[7]

2',3'-Di-*O*-*tert*butyldimethylsilyl-*N*-4-benzoyl-arabinocytidine-5'-*O*-[1''-*O*-(2'',3'',4'',6''-tetra-*O*-acetyl-α/β-D-glucopyranose)]-phosphate triethylammonium salt 12

Phosphate **12** (273 mg, 0.25 mmol, 65 %, α:β 1:6), a white foam, was synthesised following general procedure **D** from **2** (222 mg, 0.39 mmol, 1.0 equiv.) and **11** (300 mg, 0.58 mmol, 1.5 equiv.) purified *via* flash column chromatography on silica gel (DCM/MeOH, 100:0→95:5, +1% Et_3N). R_f = 0.30 (DCM/MeOH, 98:2, +1% Et_3N). **diastereoisomer β**: ^{1}H NMR (400 MHz, $CDCl_3$) δ 8.27 (d, $^3J_{H6-H5}$ = 7.3 Hz, 1H, H6), 7.92 – 7.87 (m, 2H, 2 × HAr), 7.62 – 7.56 (m, 2H, Har, H5), 7.50 (app. t, $^3J_{HAr-Ar5}$ = 7.7 Hz, 2H, 2 × HAr), 6.29 (d, $^3J_{H1'-H2'}$ = 3.1 Hz, 1H, H1'), 5.30 (t, $^3J_{H1''-H2''}$ = 8.1 Hz, $^3J_{H1''-P}$ = 8.1 Hz, 1H, H1''), 5.20 (t, $^3J_{H3''-H2''}$ = 9.5 Hz, $^3J_{H3''-H4''}$ = 9.6 Hz, 1H, H3''), 5.10 (t, $^3J_{H4''-H3''}$ = 9.6 Hz, $^3J_{H4''-H5''}$ = 9.9 Hz, 1H, H4''), 5.02 (dd, $^3J_{H2''-H3''}$ = 9.5 Hz, $^3J_{H2''-H1''}$ = 8.1 Hz, 1H, H2''), 4.31 (d, $^3J_{H2'-H1'}$ = 3.1 Hz, 1H, H2'), 4.28 – 4.17 (m, 3H, H4', H5'a, H6''a), 4.14 (dd, $^2J_{H5'b-H5'a}$ = 12.3 Hz, $^3J_{H5'b-H4'}$ = 2.6 Hz, 1H, H5'b), 4.05 (s, 1H, H3'), 4.04 – 3.93 (m, 1H, H6''b), 3.78 (ddd, $^3J_{H5''-H4''}$ = 9.9 Hz, $^3J_{H5''-H6''a}$ = 4.0 Hz, $^3J_{H5''-H6''b}$ = 2.6 Hz, 1H, H5''), 2.97 (q, $^3J_{CH2-CH3}$ = 7.3 Hz, 6H, 3 × CH_{2Et3N}), 2.05 (s, 3H, CH_{3Ac}), 2.04 (s, 3H, CH_{3Ac}), 2.01 (s, 3H, CH_{3Ac}), 1.98 (s, 3H, CH_{3Ac}), 1.27 (t, $^3J_{CH3-CH2}$ = 7.3 Hz, 9H, 3 × CH_{3Et3N}), 0.90 (s, 9H, ^{t}Bu$_{TBS}$), 0.77 (s, 9H, ^{t}Bu$_{TBS}$), 0.12 (s, 6H, 2 × Me_{TBS}), 0.03 (s, 3H, Me_{TBS}), –0.17 (s, 3H, Me_{TBS}); ^{13}C NMR (101 MHz, $CDCl_3$) δ 170.7 (C=O_{Ac}), 170.2 (C=O_{Ac}), 169.74 (C=O_{Ac}), 169.70 (C=O_{Ac}), 162.0 (C4/C2), 147.9 (C6), 133.2 (C_{Ar}), 129.1 (C_{Ar}), 127.6 (C_{Ar}), 96.1 (d, $^3J_{C1''-P}$ = 4.9 Hz, C1''), 95.4 (C5), 89.2 (C1'), 87.0 (d, $^3J_{C4'-P}$ = 6.9 Hz, C4'), 78.9

(C3'), 76.3 (C2'), 73.2 (C3''), 72.1 (ov. s, C2'', C5''), 68.4 (C4''), 65.5 (d, $^{2}J_{C6''-P}$ = 5.9 Hz, C6''), 62.0 (C5''), 45.8 (3 × CH_{2Et3N}), 25.9 ($^{t}Bu_{TBS}$), 25.8 ($^{t}Bu_{TBS}$), 21.0 (CH_{3Ac}), 20.9 (CH_{3Ac}), 20.8 (2 × CH_{3Ac}), 18.1 (C_{TBS}), 17.9 (C_{TBS}), 9.1 (3 × CH_{3Et3N}), –4.5 (Me_{TBS}), –4.5 (Me_{TBS}), –5.0 (Me_{TBS}), –5.3 (Me_{TBS}); ^{31}P NMR (162 MHz, $CDCl_3$) δ –2.33; HRMS (ESI$^+$) *m/z*: [M + H]$^+$ Calcd for $C_{42}H_{65}N_3O_{18}PSi_2$ 986.3534; Found 986.3532.

3'-*O*-*tert*Butyldimethylsilyl-*N*-4-benzoyl-2’-deoxy-2’,2’-difluorocytidine-5'-*O*-[1''-*O*-(2'',3'',4'',6''-tetra-*O*-acetyl-α/β-D-glucopyranose)]-phosphate triethylammonium salt 13

Phosphate **13** (373 mg, 0.38 mmol, 84 %, α:β 1:7), a white foam, was synthesised following general procedure **D** from **1** (214 mg, 0.44 mmol, 1.0 equiv.) and **11** (342 mg, 0.67 mmol, 1.5 equiv.) purified *via* flash column chromatography on silica gel (DCM/MeOH, 100:0→95:5, +1% Et_3N). R_f = 0.30 (DCM/MeOH, 98:2, +1% Et_3N). **diastereoisomer β**: ^{1}H NMR (400 MHz, $CDCl_3$) δ 8.32 (d, $^{3}J_{H6-H5}$ = 7.8 Hz, 1H, H6), 7.95 – 7.91 (m, 2H, 2 × HAr), 7.63 – 7.53 (m, 2H, Har, H5), 7.49 (app. t, $^{3}J_{HAr-HAr}$ = 8.1 Hz, 2H, 2 × HAr), 6.40 – 6.32 (m, 1H, H1'), 5.32 (t, $^{3}J_{H1''-H2''}$ = 8.1 Hz, $^{3}J_{H1''-P'}$ = 8.1 Hz, 1H, H1''), 5.20 (t, $^{3}J_{H3''-H2''}$ = 9.4 Hz, $^{3}J_{H3''-H4''}$ = 9.4 Hz, 1H, H3''), 5.10 (t, $^{3}J_{H4''-H3''}$ = 9.4 Hz, $^{3}J_{H4''-H5''}$ = 9.4 Hz, 1H, H4''), 5.01 (dd, $^{3}J_{H2''-H3''}$ = 9.4 Hz, $^{3}J_{H2''-H1''}$ = 8.1 Hz, 1H, H2''), 4.44 – 4.25 (m, 2H, H3', H5'a), 4.25 – 4.10 (m, 2H, H6''a, H6''b), 4.10 – 4.01 (m, 2H, H4', H5'b), 3.81 (ddd, $^{3}J_{H5''-H4''}$ = 10.0 Hz, $^{3}J_{H5''-H6''a}$ = 4.2 Hz, $^{3}J_{H5''-H6''b}$ = 2.4 Hz, 1H, H5''), 3.05 (, $^{3}J_{CH2-CH3}$ = 7.3 Hz, 6H, 3 × CH_{2Et3N}), 2.03 (s, 3H, CH_{3Ac}), 2.01 (s, 3H, CH_{3Ac}), 2.00 (s, 3H, CH_{3Ac}), 1.97 (s, 3H, CH_{3Ac}), 1.32 (t, $^{3}J_{CH3-CH2}$ = 7.3 Hz, 9H, 3 × CH_{3Et3N}), 0.89 (s, 9H, $^{t}Bu_{TBS}$), 0.13 (s, 3H, Me_{TBS}), 0.12 (s, 3H, Me_{TBS}); ^{13}C NMR (101 MHz, $CDCl_3$) δ 170.6 (C=O_{Ac}), 170.2 (C=O_{Ac}), 169.7 (2 × C=O_{Ac}), 133.3 (C_{Ar}), 129.0 (C_{Ar}), 128.0 (C_{Ar}), 73.1 (C3''), 72.12 (C2''), 72.10 (d, $^{3}J_{C5''-P}$ = 8.9 Hz, C5''), 68.3 (C4''), 63.03 – 62.75 (m, C5'), 62.0 – 61.8 (m, C6''), 45.7 (3 × CH_{2Et3N}),, 25.7 ($^{t}Bu_{TBS}$), 20.9 (CH_{3Ac}), 20.8 (CH_{3Ac}), 20.7 (2 × CH_{3Ac}), 18.1 (C_{TBS}), 8.8 (3 × CH_{3Et3N}), –4.8 (Me_{TBS}), –4.9 (Me_{TBS}); ^{19}F {^{1}H} NMR (377 MHz, $CDCl_3$) δ –116.2 (d, $^{2}J_{F-F}$ = 239.3 Hz, F), –118.6 (d, $^{2}J_{F-F}$ = 241.0 Hz, F);^{31}P NMR (162 MHz, $CDCl_3$) δ –3.42; HRMS (ESI$^+$) *m/z*: [M + H]$^+$ Calcd for $C_{36}H_{49}F_2N_3O_{17}PSi$ 892.2531; Found 892.2515.

Arabinocytidine-5'-*O*-(1''-*O*-α/β-D-glucopyranose)-phosphate sodium salt 14

Phosphate **14** (21.8 mg, 43.0 μmol, 16 %, α:β 1:5), a white foam, was synthesised following general procedure **E** then **F** from **12** (273 mg, 0.27 mmol, 1.0 equiv.) and purified *via* HILIC-B HPLC, Polaris 5, 250 × 21.2 mm; flow rate: 20.0 mL/min; retention time: 20.48 min; following method:

Time (min.)	%A (H_2O, 0.1% formic	%B (Acetonitrile)
0.0	5	95
5.0	5	95
7.0	60	40
25.0	95	5
30.0	95	5
31.0	5	95
41.0	5	95

Observed for both diastereoisomers: ^{1}H NMR (400 MHz, D_2O) δ 8.10 (d, $^3J_{H6\text{-}H5}$ = 7.8 Hz, 2H, H6$_α$, H6$_β$), 6.24 (ov. d, 4H, H5$_α$, H5$_β$, H1'$_α$, H1'$_β$), 4.49 (t, $^3J_{H2'\text{-}H1'}$ = 5.4 Hz, $^3J_{H2'\text{-}H3'}$ = 5.4 Hz. 2H, H2'$_α$, H2'$_β$), 4.28 (ddd, $^3J_{H5'a\text{-}H5'b}$ = 11.6 Hz, $^3J_{H5'a\text{-}H4'}$ = 5.2 Hz, $^3J_{H5'a\text{-}P}$ = 2.9 Hz, 2H, H5'a$_α$, H5'a$_β$), 4.24 – 4.15 (m, 4H, H4'$_α$, H4'$_β$ H5'b$_α$, H5'b$_β$), 4.15 – 4.11 (m, 2H, H4'$_α$, H4'$_β$), 3.76 – 3.69 (m, 2H, H3''$_α$, H6''b$_β$), 3.56 – 3.47 (m, 2H, H3''$_β$, H4''$_α$/H5''$_α$, H5''$_β$); **diastereoisomer α**: ^{1}H NMR (400 MHz, D_2O) δ 5.52 (dd, $^3J_{H1''\text{-}P}$ = 7.2 Hz, $^3J_{H1''\text{-}H2''}$ = 3.5 Hz, 1H, H1''), 3.87 – 3.76 (m, 3H, H4''/H5'', H6''a, H6''b), 3.58 (d, $^3J_{H2''\text{-}H3''}$ = 10.6 Hz, 1H, H2''); **diastereoisomer β**: ^{1}H NMR (400 MHz, D_2O) δ 4.93 (t, $^3J_{H1''\text{-}P}$ = 7.6 Hz, $^3J_{H1''\text{-}H2''}$ = 7.6 Hz, 1H, H1''), 3.91 (dd, $^3J_{H6''a\text{-}H6''b}$ = 12.4 Hz, $^3J_{H6''a\text{-}H5''}$ = 2.1 Hz, 1H, H6''a), 3.41 (dd, $^3J_{H4''\text{-}H3''/H5''}$ = 9.9 Hz, $^3J_{H4''\text{-}H3''/H5''}$ 8.9 Hz, 1H, H4''), 3.35 (dd, $^3J_{H2''\text{-}3H''}$ = 9.6 Hz, $^3J_{H2''\text{-}H1''}$ = 7.8 Hz, 1H, H2''); **Observed for both diastereoisomers**: ^{13}C NMR (101 MHz, D_2O) δ 160.3 (C2), 149.7 (C4), 94.6 (C5), 85.6 (C1'), 75.3 (C2'), 63.8 (d, $^2J_{C5'\text{-}P}$ = 5.1 Hz; **diastereoisomer α**: ^{13}C NMR (101 MHz, D_2O) δ 144.8 (C6), 95.4 (d, $^2J_{C1''\text{-}P}$ = 6.2 Hz, C1''), 81.5 (d, $^3J_{C4'\text{-}P}$ = 8.1 Hz, C4'), 73.7 (C3'), 72.9 (C4''/C5''), 72.5 (C3''), 71.3 (d, $^3J_{C2''\text{-}P}$ = 8.1 Hz, C2''), 69.1 (C4''/C5''), 60.3 (C6''); **diastereoisomer β**: ^{13}C NMR (101 MHz, D_2O) δ 144.8 (C6), 97.8 (d, $^2J_{C1''\text{-}P}$ = 6.2 Hz, C1), 81.5 (d, $^3J_{C4'\text{-}P}$ = 8.4 Hz, C4'), 76.5 (C5), 75.3 (d, $^4J_{C3''\text{-}P}$ = 1.8 Hz, C3''), 73.6 (C3'), 73.5 (d, $^3J_{C2''\text{-}P}$ = 8.4 Hz, C2''), 69.4 (C4''), 60.7 (C6''); **diastereoisomer α**: ^{31}P NMR (162 MHz, D_2O) δ –1.53; **diastereoisomer β**: ^{31}P NMR (162 MHz, D_2O) δ –-1.70; HRMS (ESI$^-$) *m/z*: $[M - H]^-$ Calcd for $C_{15}H_{23}N_3O_{13}P$ 484.0974; Found 484.0978.

2'-Deoxy-2',2'-difluorocytidine-5'-*O*-(1''-*O*-α/β-D-glucopyranose)-phosphate sodium salt 15

Phosphate **15** (25.7 mg, 48.7 μmol, 13 %, α:β 3:2), a white foam, was synthesised following general procedure **E** then **F** from **13** (370 mg, 0.37 mmol, 1.0 equiv.) and purified *via* HILIC-B HPLC, Polaris 5, 250 × 21.2 mm; flow rate: 20.0 mL/min; retention time: 20.42 min; following method:

Time (min.)	%A (H_2O, 0.1% formic	%B (Acetonitrile)
0.0	5	95
5.0	5	95
7.0	80	20
25.0	95	5
30.0	95	5
31.0	5	95
41.0	5	95

Observed for both diastereoisomers: ^{1}H NMR (400 MHz, D_2O) δ 6.30 (ov. d, $^3J_{H5\text{-}H6}$ =7.9 Hz, 1H), 6.27 (ov. t, $^3J_{H1'\text{-}F}$ = 7.2 Hz, $^3J_{H1'\text{-}F}$ = 7.2 Hz, 1H, H1'$_\alpha$, H1'$_\beta$), 4.52 (ov. td, $^3J_{H3'\text{-}F}$ = 12.0 Hz, $^3J_{H3'\text{-}H4'}$ = 8.2 Hz, 2H, H3'$_\alpha$, H3'$_\beta$), 4.42 – 4.32 (m, 2H, H5'a$_\alpha$, H5'a$_\beta$), 4.28 – 4.19 (m, 4H, H4'$_\alpha$, H4'$_\beta$, H5'b$_\alpha$, H5'b$_\beta$), 3.77 – 3.69 (m, 2H, H3''$_\alpha$, H6''b$_\beta$), 3.57 – 3.48 (m, 2H, H3''$_\beta$, H4''$_\alpha$/H5''$_\alpha$, H5''$_\beta$); **diastereoisomer α**: ^{1}H NMR (400 MHz, D_2O) δ 8.06 (d, $^3J_{H6\text{-}H5}$ = 7.9 Hz, 1H, H6), 5.53 (dd, $^3J_{H1''\text{-}P}$ = 7.2 Hz, $^3J_{H1''\text{-}H2''}$ = 3.5 Hz, 1H, H1''), 3.87 – 3.77 (m, 3H, H4''/H5'', H6''a, H6''b), 3.59 (d, $^3J_{H2''\text{-}H3''}$ = 10.6 Hz, 1H, H2''), **diastereoisomer β**: ^{1}H NMR (400 MHz, D_2O) δ 8.05 (d, $^3J_{H6\text{-}H5}$ = 7.9 Hz, 1H, H6), 4.94 (t, $^3J_{H1''\text{-}P}$ = 7.7 Hz, $^3J_{H1''\text{-}H2''}$ = 7.7 Hz, 1H, H1''), 3.91 (dd, $^3J_{H6''a\text{-}H6''b}$ = 12.4 Hz, $^3J_{H6''a\text{-}H5''}$ = 2.3 Hz, 1H, H6''a$_\beta$), 3.42 (dd, $^3J_{H4''\text{-}H3''/H5''}$ = 9.8 Hz, $^3J_{H4''\text{-}H3''/H5''}$ 9.0 Hz, 1H, H4''), 3.36 (dd, $^3J_{H2''\text{-}3H''}$ = 9.5 Hz, $^3J_{H2''\text{-}H1''}$ = 7.8 Hz, 1H, H2''); **diastereoisomer α**: ^{31}P {^{1}H} NMR (162 MHz, D_2O) δ –1.53; **diastereoisomer β**: ^{31}P {^{1}H} NMR (162 MHz, D_2O) δ –1.78; **Observed for both diastereoisomers**: ^{19}F {^{1}H} NMR (377 MHz, D_2O) δ -117.7 (ddd, $^2J_{F\text{–}F}$ = 241.0), -118.6 (br. d, $^2J_{F\text{–}F}$ = 241.0 Hz); HRMS (ESI$^-$) *m/z*: [M – H]$^-$ Calcd for $C_{15}H_{21}F_2N_3O_{12}P$ 504.0836; Found 504.0841.

1,2,3,4-Tetra-*O*-acetyl-β-D-galctopyranosyl-6-*O*-hydrogenphosphonate triethylammonium salt 17

2,3,4,6-*O*-Acetyl-β-D-galactopyranoside **16** (500 mg, 1.44 mmol, 1.0 equiv.) was dissolved in pyridine (8.0 mL) and treated with H_3PO_3 (589 mg, 7.18 mmol, 5.0 equiv.). The solution was cooled to 0 °C, treated dropwise with PivCl (439 μL, 3.58 mmol, 2.5 equiv.) and stirred at rt for 1.5 h. The reaction was monitored by TLC analysis (DCM/MeOH, 98:2, +1% Et_3N) showing appearance of the product at a lower R_f. The

solution was treated with Et_3N (4.0 mL) and H_2O (2.0 mL) and was stirred for 30 min. The volatiles were removed *in vacuo* and the crude material was suspended in THF. The precipitate (triethylammonium hydrogenphosphonate) was filtered off and washed with THF. The filtrate was recovered and the volatiles were removed *in vacuo*. The crude residue was purified *via* flash column chromatography on silica gel (DCM/MeOH, 100:0→90:10, +1% Et_3N) to afford the *H*-phosphonate **17** (503 mg, 0.98 mmol, 68%, α:β 1:11) as a colourless oil. R_f = 0.26 (DCM/MeOH, 95:5, +1% Et_3N); ^{1}H NMR (400 MHz, $CDCl_3$) δ 6.74 (d, $^1J_{H\text{-}P}$ = 630.6 Hz, 1H, H–P), 5.67 (d, $^3J_{H1\text{-}H2}$ = 8.3 Hz, 1H, H1), 5.45 (dd, $^3J_{H4\text{-}H3}$ = 3.4 Hz, $^3J_{H4\text{-}H5}$ = 1.0 Hz, 1H, H5), 5.26 (dd, $^3J_{H2\text{-}H3}$ = 10.4 Hz, $^3J_{H2\text{-}H1}$ = 8.3 Hz, 1H, H1), 5.03 (dd, $^3J_{H3\text{-}H2}$ = 10.5 Hz, $^3J_{H3\text{-}H4}$ = 3.4 Hz, 1H, H3), 4.08 (ddd, $^3J_{H5\text{-}H6a}$ = 7.5 Hz, $^3J_{H5\text{-}H6b}$ = 6.0 Hz, $^3J_{H5\text{-}H4}$ = 1.0 Hz, 1H, H5), 3.93 (ddd, $^3J_{H6b\text{-}H6a}$ = 10.6 Hz, $^3J_{H6b\text{-}P}$ = 8.5 Hz, $^3J_{H6b\text{-}H5}$ = 6.0 Hz, 1H, H6b), 3.83 (td, $^3J_{H6a\text{-}H6b}$ = 10.6 Hz, $^3J_{H6a\text{-}P}$ = 10.5 Hz, 7.5 Hz, 1H, H6a), 3.02 (q, $^3J_{CH2\text{-}CH3}$ = 7.3 Hz, 6H, 3 × CH_{2Et3N}), 2.10 (s, 3H, CH_{3Ac}), 2.05 (s, 3H, CH_{3Ac}), 1.98 (s, 3H, CH_{3Ac}), 1.92 (s, 3H, CH_{3Ac}), 1.24 (t, $^3J_{CH3\text{-}CH2}$ = 7.3 Hz, 9H, 3 × CH_{3Et3N}); ^{13}C NMR (101 MHz, $CDCl_3$) δ 170.3 ($C=O_{Ac}$), 170.0 ($C=O_{Ac}$), 169.5 ($C=O_{Ac}$), 168.9 ($C=O_{Ac}$), 92.3 (C1), 73.2 (d, $^3J_{HC5\text{-}P}$ = 7.0 Hz), 71.3 (C3), 68.3 (C2), 67.0 (C4), 60.6 (d, $^2J_{HC6\text{-}P}$ = 3.7 Hz), 45.4 (3 × CH_{2Et3N}), 20.9 (CH_{3Ac}), 20.8 (CH_{3Ac}), 20.72 (CH_{3Ac}), 20.65 (CH_{3Ac}), 8.5 (3 × CH_{3Et3N}); ^{31}P NMR (162 MHz, $CDCl_3$) δ 4.07 (ddd, $^1J_{P\text{-}H}$ = 630.6 Hz, $^3J_{P\text{-}H6a}$ = 10.5 Hz, $^3J_{P\text{-}H6b}$ = 8.5 Hz). MS (ESI$^-$) *m/z*: $[M - H]^-$ Calcd for $C_{14}H_{20}O_{12}P$ 411.1; Found 411.2.

2',3'-*O*-*tert*Butyldimethylsilyl-*N*-4-benzoyl-2'-deoxy-2',2'-difluorocytidine-5'-*O*-[6''-*O*-(1'',2'',3'',4''-tetra-*O*-acetyl-β-D-galactopyranose)]-phosphate triethylammonium salt 18

Phosphate **18** (177 mg, 0.18 mmol, 68%), a colourless oil, was synthesised following general procedure **D** from **1** (125 mg, 0.26 mmol, 1.0 equiv.) and **17** (200 mg, 0.39 mmol, 1.5 equiv.) and purified *via* flash column chromatography on silica gel (DCM/MeOH, 100:0→95:5, +1% Et_3N). R_f = 0.37 (DCM/MeOH, 95:5, +1% Et_3N). ^{1}H NMR (400 MHz, $CDCl_3$) δ 8.31 (d, $^3J_{H6\text{-}H5}$ = 7.6 Hz, 1H, H6), 7.96 – 7.92 (m, 2H, 2 × HAr), 7.63 – 7.51 (m, 2H, Har, H5), 7.51 – 7.44 (m, 2H, 2 × HAr), 6.35 (dd, $^3J_{H1'\text{-}F}$ = 9.8 Hz, $^3J_{H1'\text{-}F}$ = 4.9 Hz, 1H, H1'), 5.70 (d, $^3J_{H1''\text{-}H2''}$ = 8.2 Hz, 1H, H1''), 5.48 (d, $^3J_{H4''\text{-}H3''}$ = 3.4 Hz, 1H, H4''), 5.29 (dd, $^3J_{H2''\text{-}H3''}$ = 10.5 Hz, $^3J_{H2''\text{-}H1''}$ = 8.2 Hz, 1H, H2''), 5.03 (dd, $^3J_{H3''\text{-}H2''}$ = 10.5 Hz, $^3J_{H3''\text{-}H4''}$ = 3.4 Hz, 1H, H3''), 4.41 – 4.21 (m, 2H, H3', H5'a/6''a), 4.13 (t, $^3J_{H5''\text{-}H6''a}$ = 6.7 Hz, $^3J_{H5''\text{-}H6''a}$ = 6.7 Hz, 1H, H5'') 4.09 – 3.75 (m,

4H, H4', H5'a/6''a, H5'b, H6''b), 3.05 (q, ${}^{3}J_{CH2\text{-}CH3}$ = 7.3 Hz, 6H, 3 × CH_{2Et3N}), 2.12 (s, 3H, CH_{3Ac}), 2.05 (s, 3H, CH_{3Ac}), 2.00 (s, 3H, CH_{3Ac}), 1.94 (s, 3H, CH_{3Ac}), 1.29 (t, ${}^{3}J_{CH3\text{-}CH2}$ = 7.3 Hz, 9H, 3 × CH_{3Et3N}), 0.89 (s, 9H, ${}^{t}Bu_{TBS}$), 0.11 (ov. s, 6H, 2 × Me_{TBS}); ^{13}C NMR (101 MHz, $CDCl_3$) δ 170.3 (C=O_{Ac}), 170.0 (C=O_{Ac}), 169.5 (C=O_{Ac}), 168.9 (C=O_{Ac}), 163.2 (C4/C2), 145.7 (C6), 133.2 (C_{Ar}), 128.9 (C_{Ar}), 128.1 (C_{Ar}), 121.9 (C2'), 97.4 (C5), 92.3 (C1''), 84.9 (dd, ${}^{3}J_{C1'\text{-}F}$ = 40.7 Hz, ${}^{3}J_{C1'\text{-}F}$ = 23.1 Hz, C1'), 81.1 (t, ${}^{3}J_{C4'\text{-}P}$ = 8.3 Hz, C4'), 73.1 (d, ${}^{3}J_{C5''\text{-}P}$ = 7.7 Hz, C5''), 71.3 (C3''), 70.6 (dd, ${}^{3}J_{C3'\text{-}F}$ = 28.7 Hz, ${}^{3}J_{C3'\text{-}F}$ = 17.5 Hz, C3'), 68.2 (C2''), 67.0 (C4''), 62.6 (ov. d, ${}^{2}J_{C5'/6''\text{-}P}$ = 4.7 Hz, C5', C6''), 45.7 (3 × CH_{2Et3N}), 25.6 (${}^{t}Bu_{TBS}$), 20.9 (CH_{3Ac}), 20.82 (CH_{3Ac}), 20.75 (CH_{3Ac}), 20.7 (CH_{3Ac}), 18.1 (C_{TBS}), 8.6 (3 × CH_{3Et3N}), –4.8 (Me_{TBS}), –5.0 (Me_{TBS}); ^{19}F NMR (377 MHz, $CDCl_3$) δ –116.0 (ddd, ${}^{2}J_{F\text{-}F}$ = 239.7 Hz, ${}^{3}J_{F\text{-}H3'}$ = 12.6 Hz, ${}^{3}J_{F\text{-}H1'}$ = 4.9 Hz, F), –118.2 (dt, ${}^{2}J_{F\text{-}F}$ = 239.3 Hz, ${}^{3}J_{F\text{-}H1'}$ = 9.3 Hz, F); ^{31}P {^{1}H} NMR (162 MHz, $CDCl_3$) δ –1.15; HRMS (ESI$^+$) *m/z*: [M + H]$^+$ Calcd for $C_{36}H_{49}F_2N_3O_{17}PSi$ 892.2531; Found 892.2535.

2',3'-Di-*O*-*tert*butyldimethylsilyl-*N*-4-benzoyl-arabinocytidine-5'-*O*-[6''-*O*-(1'',2'',3'',4''-tetra-*O*-acetyl-α/β-D-galactopyranose)]-phosphate triethylammonium salt 19

Phosphate **19** (200 mg, 0.18 mmol, 37 %, α:β 1:8), a white foam, was synthesised following general procedure **D** from **2** (368 mg, 0.50 mmol, 1.0 equiv.) and **17** (259 mg, 0.74 mmol, 1.5 equiv.) and purified *via* flash column chromatography on silica gel (DCM/MeOH, 1:0→9:1, +1% Et_3N). R_f = 0.30 (DCM/MeOH, 98:2, +1% Et_3N). **diastereoisomer β**: ^{1}H NMR (400 MHz, $CDCl_3$) δ 8.24 (d, ${}^{3}J_{H6\text{-}H5}$ = 7.5 Hz, 1H, H6), 8.00 (app. dt, ${}^{3}J_{HAr\text{-}HAr}$ = 7.0 Hz, ${}^{4}J_{HAr\text{-}HAr}$ = 1.3 Hz, 2H, 2 × HAr), 7.60 – 7.53 (m, 2H, Har, H5), 7.47 (br. t, ${}^{3}J_{HAr\text{-}HAr}$ = 7.9 Hz, 2H, 2 × HAr), 6.26 (d, ${}^{3}J_{H1'\text{-}H2'}$ = 3.0 Hz, 1H, H1'), 5.70 (d, ${}^{3}J_{H1''\text{-}H2''}$ = 8.2 Hz, 1H, H1''), 5.48 (d, ${}^{3}J_{H4''\text{-}H3''}$ = 3.5 Hz, 1H, H4''), 5.29 (dd, ${}^{3}J_{H2''\text{-}H3''}$ = 10.5 Hz, ${}^{3}J_{H2''\text{-}H1''}$ = 8.2 Hz, 1H, H2''), 5.06 (dd, ${}^{3}J_{H3''\text{-}H2''}$ = 10.5 Hz, ${}^{3}J_{H3''\text{-}H4''}$ = 3.4 Hz, 1H, H3''), 4.27 (d, ${}^{3}J_{H2'\text{-}H1'}$ = 3.0 Hz, 1H, H2'), 4.24 – 4.12 (m, 3H, 4', 5'', 5'a/6''a), 4.05 (s, 1H, H3'), 4.02 – 3.79 (m, 3H, 5'a/6''a, H5'b, H6''b), 3.04 (q, ${}^{3}J_{CH2\text{-}CH3}$ = 7.3 Hz, 6H, 3 × CH_{2Et3N}), 2.13 (s, 3H, CH_{3Ac}), 2.06 (s, 3H, CH_{3Ac}), 2.00 s, 3H, CH_{3Ac}), 1.94 (s, 3H, CH_{3Ac}), 1.28 (t, ${}^{3}J_{CH3\text{-}CH2}$ = 7.3 Hz, 9H, 3 × CH_{3Et3N}), 0.87 (s, 9H, ${}^{t}Bu_{TBS}$), 0.75 (s, 9H, ${}^{t}Bu_{TBS}$), 0.10 (s, 3H, 2 × Me_{TBS}), 0.01 (s, 3H, Me_{TBS}), –0.19 (s, 3H, Me_{TBS}); ^{13}C NMR (101 MHz, $CDCl_3$) δ 170.3 (C=O_{Ac}), 170.0 (C=O_{Ac}), 169.6 (C=O_{Ac}), 168.9 (C=O_{Ac}), 162.8 (C2), 154.5 (C4), 148.0 (C6), 133.0 (C_{Ar}), 128.7 (C_{Ar}), 128.3 (C_{Ar}), 95.8 (C5), 92.3 (C1''), 89.2 (C1'), 86.9 (d, ${}^{3}J_{C4'\text{-}P}$ = 7.0 Hz, C4'), 78.7 (C3'), 76.2 (C2'), 73.1 (d, ${}^{3}J_{C5'\text{-}}$

$_P$ = 7.3 Hz, C5'), 71.3 (C3''), 68.4 (C2''), 67.1 (C4''), 65.4 (d, $^2J_{C5/6''-P}$ = 5.5 Hz, C5'/6''), 62.5 (d, $^2J_{C5/6''-P}$ = 4.6 Hz, C5'/6''), 45.6 (3 × CH_{2Et3N}), 25.8 (^{t}Bu$_{TBS}$), 25.8 (^{t}Bu$_{TBS}$), 20.9 (CH_{3Ac}), 20.8 (CH_{3Ac}), 20.7 (CH_{3Ac}), 20.7 (CH_{3Ac}), 17.9 (C_{TBS}), 17.8 (C_{TBS}), 8.6(3 × CH_{3Et3N}), –4.6 (Me_{TBS}), –4.6 (Me_{TBS}), –5.0 (Me_{TBS}), –5.3 (Me_{TBS}); ^{31}P {^{1}H} NMR (162 MHz, $CDCl_3$) δ –0.99; HRMS (ESI$^+$) *m/z*: [M + H]$^+$ Calcd for $C_{42}H_{65}N_3O_{18}PSi_2$ 986.3534; Found 986.3534.

2'-Deoxy-2',2'-difluorocytidine-5'-*O*-(6''-*O*-α/β-D-galactopyranose)-phosphate sodium salt 20

Phosphate **20** (13 mg, 25 µmol, 15%, α:β 2:3), a white foam, was synthesised following general procedure **E** then **F** from **18** (170 mg, 0.17 mmol, 1.0 equiv.) and purified *via* HILIC-B HPLC, Polaris 5, 250 × 21.2 mm; flow rate: 20.0 mL/min; retention time: 23.56 min; following method:

Time (min.)	%A (H_2O, 0.1% formic	%B (Acetonitrile)
0.0	5	95
5.0	5	95
7.0	80	20
25.0	95	5
30.0	95	5
31.0	5	95
41.0	5	95

Observed for both diastereoisomers: ^{1}H NMR (400 MHz, D_2O) δ 7.94 (d, $^3J_{H6-H5}$ = 7.8 Hz, 2H, H6$_α$, H6$_β$), 6.31 – 6.22 (m, 4H, H5$_α$, H5$_β$, H1'$_α$, H1'$_β$), 4.49 (td, $^3J_{H3'-F}$ = 12.1 Hz, $^3J_{H3'-H4'}$ = 8.5 Hz, 1H, H3'$_α$, H3'$_β$), 4.31 (br. dd, $^2J_{H5'a-H5'b}$ = 11.6 Hz, $^3J_{H5'a-H4'}$ = 4.6 Hz, 2H, H5'a$_α$, H5'a$_β$), 4.27 – 4.14 (m, 5H, H4'$_α$, H4'$_β$, H5'b$_α$, H5'b$_β$, H5''$_α$), 4.09 – 3.96 (m, 5H, H4''$_α$, H6''a$_α$, H6''b$_α$, H6''a$_β$, H6''b$_β$), 3.90 – 3.82 (m, 2H, H3''$_α$, H5''$_β$); **diasterisomer α**: ^{1}H NMR (400 MHz, D_2O) δ 5.26 (d, $^3J_{H1''-H2''}$ = 3.7 Hz, 1H, H1''), 3.80 (dd, $^3J_{H2''-H3''}$ = 10.3 Hz, $^3J_{H2''-H1''}$ = 3.7 Hz, 1H, H2''); **diasterisomer β**: ^{1}H NMR (400 MHz, D_2O) δ 4.59 (d, $^3J_{H1''-H2''}$ = 7.8 Hz, 1H, H1''), 3.95 (dd, $^3J_{H4''-H3''}$ = 3.5 Hz, $^3J_{H4''-H5''}$ = 1.2 Hz, 1H, H4''), 3.62 (dd, $^3J_{H3''-H2''}$ = 10.0 Hz, $^3J_{H3''-H4''}$ = 3.5 Hz, 1H, H3''), 3.49 (dd, $^3J_{H2''-H3''}$ = 10.0 Hz, $^3J_{H2''-H1''}$ = 7.9 Hz, 1H, H2''); **observed for both diastereoisomers**: ^{13}C NMR (101 MHz, D_2O) δ 163.0 (C4/C2), 153.0 (C4/C2), 142.3 (C6), 122.1 (t, $^1J_{C2'-F}$ = 259.0 Hz, C2'), 96.3 (C5), 84.6 (app. t, $^2J_{C1'-F}$ = 33.1 Hz, C1'), 79.2 (b. s, C4'), 68.6 (C4'), 62.7 (ov. d, $^2J_{C5'-P}$ = 4.7 Hz, C5'); **diasterisomer α**: ^{13}C NMR (101 MHz, D_2O) δ 92.3 (C1''), 68.3 (C2''), 69.2 (d, $^3J_{C5''-P}$ = 7.7 Hz, C5''), 69.0 (C4''), 68.9, (C3'') 64.9 (d, $^2J_{C6''-P}$ = 5.6 Hz, C6''); **diasterisomer β**: ^{13}C NMR (101 MHz, D_2O) δ 96.5, (C1''), 73.6 (d, $^3J_{C5''-P}$ = 7.7

Hz, C5''), 72.7 (C3''), 71.8 (C2''), 68.4 (C4''), 64.6 (d, $^{2}J_{C6''-P}$ = 5.5 Hz, C6''); **observed for both diastereoisomers**: ^{31}P {1H} NMR (162 MHz, D_2O) δ 0.15; **observed for both diastereoisomers**: ^{19}F NMR (377 MHz, D_2O) δ –117.8 – –118.0 (m); HRMS (ESI$^-$) *m/z*: [M – H]$^-$ Calcd for $C_{15}H_{21}F_2N_3O_{12}P$ 504.0836; Found 504.0847.

2,3,4,6-Tetra-*O*-acetyl-α/β-D-galactopyranosyl-1-*O*-hydrogenphosphonate triethylammonium salt 22

2,3,4,6-Tetra-*O*-acetyl-D-galactopyranose **21** (770 mg, 2.21 mol, 1.0 equiv.) was dissolved in DCM (2.2 mL), cooled to 0 °C, treated dropwise with DBU (99 µL, 0.66 mol, 0.3 equiv.) and trichloroacetonitrile (665 µL, 6.63 mol, 3.0 equiv.). The reaction was stirred at 0 °C and monitored by TLC analysis (DCM, +1% Et_3N) showing conversion of the starting material to a higher R_f after 2h. The volatiles were removed *in vacuo* and the crude material was passed through a silica plug (Et_2O, +1% Et_3N) to give the trichloroacetamidate intermediate (760 mg, 1.54 mmol, 70%) as a brown foam. The intermediate was dissolved in THF (18 mL), and treated with a H_3PO_3 solution (759 mg, 9.25 mmol, 6.0 equiv.) in THF (18 mL). The reaction solution was stirred at RT for 5 min, cooled to 0 ˚C and treated with triethylamine (3.0 mL, 22.6 mmol, 14 equiv.). The solution was stirred at 0 ˚C for 2 h. The precipitate (triethylammonium hydrogenphosphonate) was filtered off, washed with cold THF. The filtrate was recovered and the volatiles were removed *in vacuo*. The crude product was purified via flash silica column chromatography (DCM/MeOH, 100:0→94:6, +1% Et_3N) to give the product **22** as a beige hygroscopic powder (391 mg, 0.76 mmol, 49%, 34% over two steps, α:β 3:7). R_f = 0.51 (DCM/MeOH, 95:5, +1% Et_3N). ^{1}H NMR (400 MHz, $CDCl_3$) δ 6.77 (d, $^{1}J_{H-P}$ = 643.5 Hz, 1H, H–P), 5.26 (dd, $^{3}J_{H4-H3}$ = 3.5 Hz, $^{3}J_{H4-H5}$ = 1.2 Hz, 1H, H4), 5.12 (t, $^{3}J_{H1-P}$ = 8.4 Hz, $^{3}J_{H1-H2}$ = 8.4 Hz, 1H, H1), 5.06 (dd, $^{3}J_{H2-H3}$ = 10.0 Hz, $^{3}J_{H2-H}$ = 8.4 Hz, 1H, H2), 4.90 (dd, $^{3}J_{H3-H2}$ = 10.0 Hz, $^{3}J_{H3-H4}$ = 3.5 Hz, 1H, H3), 4.02 – 3.94 (m, 2H, H6a, H6b), 3.90 (ddd, $^{3}J_{H5-H6a}$ = 7.2 Hz, $^{3}J_{H5-H6b}$ = 5.9 Hz, $^{3}J_{H5-H4}$ = 1.2 Hz, 1H, H5), 2.96 (q, $^{3}J_{CH2-CH3}$ = 7.3 Hz, 6H, 3 × CH_{2Et3N}), 2.01 (s, 3H, CH_{3Ac}), 1.93 (s, 3H, CH_{3Ac}), 1.90 (s, 3H, CH_{3Ac}), 1.84 (s, 3H, CH_{3Ac}), 1.20 (t, $^{3}J_{CH3-CH2}$ = 7.3 Hz, 9H, 3 × CH_{3Et3N}); ^{31}P NMR (162 MHz, $CDCl_3$) δ 0.6; MS (ESI$^-$) *m/z*: [M − H]$^-$ Calcd for $C_{14}H_{20}O_{12}P$ 411.1; Found 411.2.These data were in agreement with literature.[8]

3'-*O*-*tert*Butyldimethylsilyl-*N*-4-benzoyl-2'-deoxy-2',2'-difluorocytidine-5'-*O*-[1''-*O*-(2'',3'',4'',6''-tetra-*O*-acetyl-α/β-D-galactopyranose)]-phosphate triethylammonium salt 23

Phosphate **23** (333 mg, 0.34 mmol, 66 %, α:β 1:5), a white foam, was synthesised following general procedure **D** from **1** (245 mg, 0.51 mmol, 1.0 equiv.) and **22** (391 mg, 0.76 mmol, 1.5 equiv.) and purified *via* flash column chromatography on silica gel (DCM/MeOH, 100:0 95:5, +1% Et_3N). R_f = 0.30 (DCM/MeOH, 98:2, +1% Et_3N). **Observed for both diastereoisomers**: ^{1}H NMR (400 MHz, $CDCl_3$) δ 8.34 (ov. d, $^3J_{H6-H5}$ = 7.9 Hz, 2H, $H6_α$, $H6_β$), 7.92 – 7.83 (m, 4H, 2 × $HAr_α$, 2 × $HAr_β$), 7.64 – 7.54 (m, 4H, 2 $HAr_α$, $HAr_β$, $H5_α$, $H5_β$), 7.54 – 7.44 (m, 4H, 4H, 2 × $HAr_α$, 2 × $HAr_β$), 6.38 (dd, $^3J_{H1'-F}$ = 10.6 Hz, $^3J_{H1'-F}$ = 5.0 Hz, 2H, $H1'_α$, $H1'_β$), 5.51 – 5.37 (m, 2H, $H4''_α$, $H4''_β$), 5.25 – 5.10 (m, 3H, $H2''_α$, $H2''_β$, $H3''_α$), 4.43 – 4.23 (m, 4H, $H3'_α$, $H3'_β$, $H5'a_α$, $H5'a_β$), 4.15 – 3.98 (m, 9H, $H4'_α$, $H4'_β$, $H5'b_α$, $H5'b_β$, $H5''_β$, $H6''a_α$, $H6''a_β$, $H6''b_α$, $H6''b_β$), 3.06 (q, $^3J_{CH2-CH3}$ = 7.3 Hz, 12H, 3 × $CH_{2Et3Nα}$, 3 × $CH_{2Et3Nβ}$), 1.33 (t, $^3J_{CH3-CH2}$ = 7.3 Hz, 18H, 3 × $CH_{3Et3Nα}$, 3 × $CH_{3Et3Nβ}$), 0.90 (s, 18H, $^t Bu_{TBSα}$, $^t Bu_{TBSβ}$), 0.16 – 0.12 (ov. s, 12H, 2 × $Me_{TBSα}$, 2 × $Me_{TBSβ}$); **diastereoisomer α**: ^{1}H NMR (400 MHz, $CDCl_3$) δ 5.82 (dd, $^3J_{H1''-P}$ = 7.7 Hz, $^3J_{H1''-H2''}$ = 3.4 Hz, 1H, H1''), 4.50 (t, $^3J_{H5''-H6''a}$ = 6.8 Hz, $^3J_{H5''-H6''b}$ = 6.8 Hz, 1H, H5''), 2.13 (s, 3H, CH_{3Ac}), 2.06 (s, 3H, CH_{3Ac}), 1.99 (s, 3H, CH_{3Ac}), 1.97 (s, 3H, CH_{3Ac}); **diastereoisomer β**: ^{1}H NMR (400 MHz, $CDCl_3$) δ 5.28 (t, $^3J_{H1''-P}$ = 7.8 Hz, $^3J_{H1''-H2''}$ = 7.8 Hz 1H, H1''), 5.03 (dd, $^3J_{H3''-H2''}$ = 10.1 Hz, $^3J_{H3''-H4''}$ = 3.5 Hz, 1H, H3''),2.12 (s, 3H, CH_{3Ac}), 2.04 (s, 3H, CH_{3Ac}), 2.01 (s, 3H, CH_{3Ac}), 1.96 (s, 3H, CH_{3Ac}); ^{13}C NMR (101 MHz, $CDCl_3$) δ 170.5 ($C{=}O_{Acα/β}$), 170.41 ($C{=}O_{Acα/β}$), 170.40 ($C{=}O_{Acα/β}$), 170.3 ($C{=}O_{Acα/β}$), 170.13 ($C{=}O_{Acα/β}$), 170.09 ($C{=}O_{Acα/β}$), 169.9 ($C{=}O_{Acα/β}$), 133.3 (C_{Ar}), 129.1 (C_{Ar}), 127.7 (C_{Ar}), 71.2 – 70.5 (m, 4C, $C3'_α$, $C3'_β$, $C3''_β$, $C5''_α$), 69.7 (d, $^3J_{C2''-P}$ = 8.1 Hz, $C2''_{α/β}$), 68.0 ($C2''_{α/β}$), 67.4 ($C4''_{α/β}$), 67.1 (ov. s, 2C, $C4''_{α/β}$, $C5''_α$), 63.0 – 62.5 (m, 2C, $C5'_α$, $C5'_β$), 61.1(ov. s, 2C, $C5'_α$, $C5'_β$) (ov. m, $C5'_α$, $C5'_β$), 45.7(3 × $CH_{2Et3Nα/β}$), 25.7 ($^t Bu_{TBSα/β}$), 25.6 ($^t Bu_{TBSα/β}$), 21.1 ($CH_{3Acα/β}$), 21.1 ($CH_{3Acα/β}$), 20.8 ($CH_{3Acα/β}$), 20.7 ($CH_{3Acα/β}$), 18.1 ($C_{TBSα}$, $C_{TBSβ}$), 8.7 (3 × $CH_{3Et3Nα/β}$), –4.7 ($Me_{TBSα/β}$), –4.8 ($Me_{TBSα/β}$), –4.9 ($Me_{TBSα/β}$), –5.0 ($Me_{TBSα/β}$); **diastereoisomer α**: ^{31}P {^{1}H} NMR (162 MHz, $CDCl_3$) δ –2.33; **diastereoisomer β**: ^{31}P {^{1}H} NMR (162 MHz, $CDCl_3$) δ –3.15; **diastereoisomer α**: ^{19}F {^{1}H} NMR (377 MHz, $CDCl_3$) δ –115.83 (d, $^2J_{F-F}$ = 240.0 Hz), –118.08 (d, $^2J_{F-F}$ = 240.0 Hz); **diastereoisomer β**: ^{19}F {^{1}H} NMR (377 MHz, $CDCl_3$) δ–116.08 (d, $^2J_{F-F}$

= 239.3 Hz), –118.57 (d, $^{2}J_{F-F}$ = 239.3 Hz); HRMS (ESI^{+}) *m/z*: [M + H]$^{+}$ Calcd for $C_{36}H_{49}F_{2}N_{3}O_{17}PSi$ 892.2531; Found 892.2513.

2',3'-Di-*O*-*tert*butyldimethylsilyl-*N*-4-benzoyl-arabinocytidine-5'-*O*-[1''-*O*-(2'',3'',4'',6''-tetra-*O*-acetyl-α/β-D-galactopyranose)]-phosphate triethylammonium salt 24

Phosphate **24** (273 mg, 0.25 mmol, 65%, α:β 2:5), a white foam, was synthesised following general procedure **D** from **3** (222 mg, 0.39 mmol, 1.0 equiv.) and **22** (300 mg, 0.59 mmol, 1.5 equiv.) and purified *via* flash column chromatography on silica gel (DCM/MeOH, 100:0→95:5, +1% Et_3N). R_f = 0.30 (DCM/MeOH, 98:2, +1% Et_3N). **Observed for both diastereoisomers**: ^{1}H NMR (400 MHz, $CDCl_3$) δ 7.85 – 7.79 (m, 4H, 2 × $HAr_{α}$, 2 × $HAr_{β}$), 7.50 – 7.44 (m, 4H, 2 $HAr_{α}$, $HAr_{β}$, $H5_{α}$, $H5_{β}$), 7.40 – 7.34 (m, 4H, 4H, 2 × $HAr_{α}$, 2 × $HAr_{β}$), 6.17 (t, $^{3}J_{H1'-H2'}$ = 3.5 Hz, 2H, $H1'_{α}$, $H1'_{β}$), 5.09 (ov. dd, $^{3}J_{H2''β-H3''β}$ = 10.5 Hz, $^{3}J_{H2''β-H1''β}$ = 7.9 Hz, 1H, $H2''_{α}$, $H2''_{β}$), 4.18 (d, $^{3}J_{H2'-H1'}$ = 3.5 Hz, 2H, $H2'_{α}$, $H2'_{β}$), 4.15 – 4.06 (m, 6H, $H4'_{α}$, $H4'_{β}$, $H5'a_{α}$, $H5'a_{β}$), 4.06 – 3.93 (m, 4H, $H6''a_{α}$, $H6''a_{β}$, $H6''b_{α}$, $H6''b_{β}$), 3.93 – 3.91 (m, 2H, $H3'_{α}$, $H3'_{β}$), 3.90 – 3.85 (m, 3H, $H5'b_{α}$, $H5'b_{β}$' $H5''_{β}$), 2.92 (q, $^{3}J_{CH2-CH3}$ = 7.3 Hz, 12H, 3 × $CH_{2Et3Nα}$, 3 × $CH_{2Et3Nβ}$), 1.94 (s, 6H, $CH_{3Acα}$, $CH_{3Acβ}$), 1.17 (t, $^{3}J_{CH3-CH2}$ = 7.3 Hz, 18H, 3 × $CH_{2Et3Nα}$, 3 × $CH_{3Et3Nβ}$), 0.77 (ov. s, 18H, $^{t}Bu_{TBSα}$, $^{t}Bu_{TBSβ}$), 0.65 (s, 18H, $^{t}Bu_{TBSα}$, $^{t}Bu_{TBSβ}$), 0.02 – –0.02 (s, 12H, 2 × $Me_{TBSα}$, 2 × $Me_{TBSβ}$), –0.10 (s, 6H, $Me_{TBSα}$, $Me_{TBSβ}$), –0.29 – –0.30 (s, 6H, $Me_{TBSα}$, $Me_{TBSβ}$); **diastereoisomer α**: ^{1}H NMR (400 MHz, $CDCl_3$) δ 8.26 (d, $^{3}J_{H6-H5}$ = 7.5 Hz, 1H, H6), 5.68 (dd, $^{3}J_{H1''-P}$ = 7.6 Hz, $^{3}J_{H1''-H2''}$ = 3.4 Hz, 1H, H1''), 5.34 (dd, $^{3}J_{H4''-H3''}$ = 3.4, $^{3}J_{H4''-H5''}$ = 1.4 Hz, 1H, H4''), 5.01 (dd, $^{3}J_{H3''-H2''}$ = 10.4, $^{3}J_{H3''-H4''}$ = 3.4 Hz, 1H, H3''), 4.38 (t, $^{3}J_{H5''-H6''a}$ = 7.3 Hz, $^{3}J_{H5''-H6''b}$ = 7.3 Hz, 1H, H5''), 2.01 (s, 3H, CH_{3Ac}), 1.88 (s, 3H, CH_{3Ac}), 1.84 (s, 3H, CH_{3Ac}); **diastereoisomer β**: ^{1}H NMR (400 MHz, $CDCl_3$) δ 8.14 (d, $^{3}J_{H6-H5}$ = 7.5 Hz, 1H, H6), 5.27 (dd, $^{3}J_{H4''-H3''}$ = 3.5 Hz, $^{3}J_{H4''-H5''}$ = 1.2 Hz, 1H, H4''), 5.17 (t, $^{3}J_{H1''-P}$ = 8.1 Hz, $^{3}J_{H1''-H2''}$ = 8.1 Hz 1H, H1''), 4.91 (dd, $^{3}J_{H3''-H2''}$ = 10.3 Hz, $^{3}J_{H3''-H4''}$ = 3.4 Hz, 1H, H3''), 2.00 (s, 3H, CH_{3Ac}), 1.89 (s, 3H, CH_{3Ac}), 1.84 (s, 3H, CH_{3Ac}); **observed for both diastereoisomers**: ^{13}C NMR (101 MHz, $CDCl_3$) δ 170.4 (C=$O_{Acα/β}$), 170.4 (C=$O_{Acα/β}$), 170.3 (C=$O_{Acα/β}$), 170.2 (C=$O_{Acα/β}$), 170.1 (C=$O_{Acα/β}$), 170.0 (C=$O_{Acα/β}$), 169.9 (C=$O_{Acα/β}$), 162.3 (C2/C4), 133.1 (C_{Ar}), 128.9 (C_{Ar}), 127.8 (C_{Ar}), 87.2 (d, $^{3}J_{C4'-P}$ = 7.0 Hz, $C4'_{α/β}$), 86.9 (d, $^{3}J_{C4'-P}$ = 6.9 Hz, $C4'_{α/β}$), 65.7 – 65.2 (m, C5'), 45.5 (3 × CH_{2Et3N}), 25.7 (ov. s, 2 × $^{t}Bu_{TBS}$), 21.0 ($CH_{3Acα/β}$), 21.0 ($CH_{3Acα/β}$), 20.7 ($CH_{3Acα/β}$), 20.7 ($CH_{3Acα/β}$), 20.6 ($CH_{3Acα/β}$), 17.9 (C_{TBS}), 8.6 (3 × CH_{3Et3N}), –4.6 ($Me_{TBS\,α/β}$), –4.7 ($Me_{TBS\,α/β}$), –5.10

($Me_{TBS\,\alpha/\beta}$), –5.14 ($Me_{TBS\,\alpha/\beta}$), –5.37 ($Me_{TBS\,\alpha/\beta}$), –5.39 ($Me_{TBS\,\alpha/\beta}$); **diastereoisomer α**: ^{13}C NMR (101 MHz, $CDCl_3$) δ 92.4 (d, $^2J_{C1''-P}$ = 5.9 Hz, C1''), 89.1 (C1'), 78.8 (C3'), 76.0 (C2'), 67.9 (d, $^3J_{C2''-P}$ = 6.8 Hz, C2''), 67.6 (C4''), 66.7 (C5''), 60.8 (C6''), 17.8 (C_{TBS}); **diastereoisomer β**: ^{13}C NMR (101 MHz, $CDCl_3$) δ 96.3 (d, $^2J_{C1''-P}$ = 4.9 Hz, C1''), 89.0 (C1'), 78.7 (C3'), 76.2 (C2'), 71.1 (C3''), 70.8 (C5''), 69.5 (d, $^3J_{C2''-P}$ = 6.8 Hz, C2''), 67.0 (C4''), 60.9 (C6''), 17.8 (C_{TBS}); **diastereoisomer α**: ^{31}P {^{1}H} NMR (162 MHz, $CDCl_3$) δ –2.20; **diastereoisomer β**: ^{31}P {^{1}H} NMR (162 MHz, $CDCl_3$) δ –2.60; HRMS (ESI$^+$) *m/z*: [M + H]$^+$ Calcd for $C_{42}H_{65}N_3O_{18}PSi_2$ 986.3534; Found 986.3525.

2’-Deoxy-2’,2’-difluorocytidine-5'-*O*-(1''-*O*-α/β-D-galactopyranose)-phosphate sodium salt 25

Phosphate **25** (6.9 mg, 13 µmol, 7%, α:β 2:3), a white foam, was synthesised following general procedure **E** then **F** from **23** (180 mg, 0.18 mmol, 1.0 equiv.) and purified *via* HILIC-B HPLC, Polaris 5, 250 × 21.2 mm; flow rate: 20.0 mL/min; retention time: 22.60 min; following method:

**Time (min.)**	**%A (H_2O, 0.1% formic**	**%B (Acetonitrile)**
0.0	5	95
5.0	5	95
7.0	80	20
25.0	95	5
30.0	95	5
31.0	5	95
41.0	5	95

Observed for both diastereoisomers: ^{1}H NMR (400 MHz, D_2O) δ 7.97 (ov. d, $^3J_{H6-H5}$ = 7.8, 1H, $H6_\alpha$, $H6_\beta$), 6.27 (t, $^3J_{H1'-F}$ = 7.3 Hz, $^3J_{H1'-F}$ = 7.3 Hz, 1H, $H1'_\alpha$, $H1'_\beta$), 6.23 (ov. d, $^3J_{H5-H6}$ = 7.8 Hz, 1H, $H5_\alpha$, $H5_\beta$), 4.51 (ov. td, $^3J_{H3'-F}$ = 12.2 Hz, $^3J_{H3'-H4'}$ = 8.9 Hz, 2H, $H3'_\alpha$, $H3'_\beta$), 4.42 – 4.31 (m, 2H, $H5'a_\alpha$, $H5'a_\beta$), 4.28 – 4.17 (m, 4H, $H4'_\alpha$, $H4'_\beta$, $H5'b_\alpha$, $H5'b_\beta$), 4.12 (app. t, $^3J_{H5''-H4''}$ = 6.1 Hz, $^3J_{H5''-H6''}$ = 6.1 Hz 1H, $H5''_\alpha$), 3.79 – 3.72 (m, 5H, $H5''_\beta$, $H6''a_\alpha$, $H6''a_\beta$, $H6''b_\alpha$, $H6''b_\beta$); **diasterisomer α:** ^{1}H NMR (400 MHz, D_2O) δ 5.56 (dd, $^3J_{H1''-P}$ = 7.2 Hz, $^3J_{H1''-H2''}$ = 3.5 Hz, 1H, H1''), 4.04 (dd, $^3J_{H4''-H3''}$ = 3.1 Hz, $^3J_{H4''-H5''}$ = 1.3 Hz, 1H, H4''), 3.91 (dd, $^3J_{H3''-H2''}$ = 10.3 Hz, $^3J_{H3''-H4''}$ = 3.1 Hz, 1H, H3''), 3.86 (ddd, $^3J_{H2''-H3''}$ = 10.3, $^3J_{H2''-H1''}$ = 3.5, $^4J_{H2''-P}$ = 2.5 Hz, 1H, H2''); **diasterisomer β:** ^{1}H NMR (400 MHz, D_2O) δ 3.95 (d, $^3J_{H4''-H3'}$ = 3.5 Hz, 1H, H4''), 3.69 (dd, $^3J_{H3''-H2'}$ = 10.0 Hz, $^3J_{H3''-H4'}$ = 3.4 Hz, 1H, H3''), 3.60 (dd, $^3J_{H2''-H3'}$ = 10.0 Hz, $^3J_{H2''-H1''}$ = 7.7 Hz, 1H, H2''); **Observed for both diastereoisomers**: ^{13}C NMR (101 MHz, D_2O) δ 159.5 (C2), 148.4

(C4), 143.6 (C6), 125.0 – 118.8 (m, C2'), 95.7 (C5), 85.2 – 83.9 (m, C1'), 80.1 – 79.3 (m, C4'), 68.4 – 68.1 (m, C3'), 62.7 (ov. d, $^{2}J_{C5'-P}$ = 5.1 Hz); **diastereoisomer α**: ^{13}C NMR (101 MHz, D_2O) δ 95.7 (d, $^{2}J_{C1''-P}$ = 3.3 Hz, C1''), 75.7 (C5''), 69.1 (C4''), 69.0 (C3''), 68.1 (d, $^{3}J_{C2''-P}$ = 8.1 Hz, C2''), 61.0 (C6); **diastereoisomer β**: ^{13}C NMR (101 MHz, D_2O) δ 98.4 (d, $^{2}J_{C1''-P}$ = 6.2 Hz, C1''), 72.3 (d, $^{4}J_{C3''-P}$ = 1.7 Hz, C3''), 72.0 C5''), 71.2 (d, $^{3}J_{C2''-P}$ = 8.4 Hz, C2''), 68.5 (C4''), 61.0 (C6); **diasterisomer α:** ^{31}P {^{1}H} NMR (162 MHz, D_2O) δ –1.42; **diasterisomer β:** ^{31}P {^{1}H} NMR (162 MHz, D_2O) δ –1.69; **diastereoisomer α**: ^{19}F {^{1}H} NMR (377 MHz, D_2O) δ –117.7 (d, $^{2}J_{F-F}$ = 241.0 Hz), –118.7 (d, $^{2}J_{F-F}$ = 242.7 Hz); **diastereoisomer β**: ^{19}F {^{1}H} NMR (377 MHz, D_2O) δ –117.8 (d, $^{2}J_{F-F}$ = 241.0 Hz), –118.7 (d, $^{2}J_{F-F}$ = 241.1 Hz); HRMS (ESI$^-$) *m/z*: [M – H]$^-$ Calcd for $C_{15}H_{21}F_2N_3O_{12}P$ 504.0836; Found 504.0846.

Arabinocytidine-5'-*O*-(1''-*O*-α/β-D-galactopyranose)-phosphate sodium salt 26

Phosphate **26** (27.8 mg, 54.8 μmol, 27%, α:β 1:2), a white foam, was synthesised following general procedure **E** then **F** from **24** (212 mg, 0.20 mmol, 1.0 equiv.) and purified *via* HILIC-B HPLC, Polaris 5, 250 × 21.2 mm; flow rate: 20.0 mL/min; retention time: 20.56 min; following method:

Time (min.)	%A (H_2O, 0.1% formic	%B (Acetonitrile)
0.0	5	95
5.0	5	95
7.0	60	40
25.0	95	5
30.0	95	5
31.0	5	95
41.0	5	95

Observed for both diastereoisomers: ^{1}H NMR (400 MHz, D_2O) δ 6.27 – 6.21 (m, 4H, H1'$_α$, H1'$_β$, H5$_α$, H5$_β$), 4.48 (ov. t, $^{3}J_{H2'-H1'}$ = 5.4 Hz, $^{3}J_{H2'-H1'}$ = 5.4 Hz, 1H, H2'$_α$, H2'$_β$), 4.37 – 4.24 (m, 1H, H5'$_α$, H5'$_β$), 4.24 – 4.15 (m, 4H, H3'$_α$, H3'$_β$, H5'$_α$, H5'$_β$), 4.15 – 4.08 (m, 3H, H4'$_α$, H4'$_β$, H5''$_α$), 3.79 – 3.71 (m, 5H, H5''$_β$, H6''a$_α$, H6''a$_β$, H6''b$_α$, H6''b$_β$); **diastereoisomer α**: ^{1}H NMR (400 MHz, D_2O) δ 8.10 (d, $^{3}J_{H6-H5}$ = 7.8 Hz, 1H, H6), 5.55 (dd, $^{3}J_{H1''-P}$ = 7.2 Hz, $^{3}J_{H1''-H2''}$ = 3.4 Hz, 1H, H1''), 4.03 (dd, $^{3}J_{H4''-H3''}$ = 3.1 Hz, $^{3}J_{H4''-H5''}$ = 1.3 Hz, 1H, H4''), 3.90 – 3.79 (m, 2H, H2'', H3''); **diastereoisomer β**: ^{1}H NMR (400 MHz, D_2O) δ 8.10 (d, $^{3}J_{H6-H5}$ = 7.8 Hz, 1H, H6), 4.88 (t, $^{3}J_{H1''-P}$ = 7.5 Hz, $^{3}J_{H1''-H2''}$ = 7.5 Hz, 1H, H1''), 3.94 (d, $^{3}J_{H4''-H3''}$ = 3.4 Hz, 1H, H4''), 3.69 (dd, $^{3}J_{H3''-H2''}$ = 10.0 Hz, $^{3}J_{H3''-H4''}$ = 3.4 Hz, 1H, H3''), 3.62 – 3.55 (m, 1H, H2''); **Observed for both diastereoisomers**: ^{13}C NMR (101 MHz, D_2O) δ 144.8 (C6), 75.3 (ov. s, C2'), 63.7 (d, $^{2}J_{C5'-P}$

= 5.5 Hz, C5'); **diastereoisomer α**: ^{13}C NMR (101 MHz, D_2O) δ 160.1 (C2/C4), 149.5 (C2/C4), 95.6 (d, $^{2}J_{C1''-P}$ = 6.6 Hz, C1''), 94.6 (C5), 85.7 (C1'), 81.6 (d, $^{3}J_{C4'-P}$ = 8.4 Hz, C4'), 73.7 (C3'), 72.0 (C5''),69.1 (C4''), 69.0 (C3''), 68.1 (d, $^{3}J_{C2''-P}$ = 8.1 Hz, C2''), 61.0 (C6''); **diastereoisomer β**: ^{13}C NMR (101 MHz, D_2O) δ 160.1 (C2/C4), 149.5 (C2/C4), 98.3 (d, $^{2}J_{C1''-P}$ = 6.2 Hz, C1''), 94.6 (C5), 85.6 (C1'), 81.5 (d, $^{3}J_{C4'-P}$ = 8.4 Hz, C4'), 75.8 (C5''), 73.5 (C3'), 72.3 (d, $^{4}J_{C3'-P}$ = 1.8 Hz, C3''), 71.2 (d, $^{3}J_{C2''-P}$ = 8.8 Hz, C2''), 68.5 (C4''), 61.0 (C6''); **diastereoisomer α**: ^{31}P {^{1}H} NMR (162 MHz, D_2O) δ –1.39; **diastereoisomer β**: ^{31}P {^{1}H} NMR (162 MHz, D_2O) δ –1.60; HRMS (ESI$^-$) *m/z*: $[M - H]^-$ Calcd for $C_{15}H_{23}N_3O_{13}P$ 484.0974; Found 484.0976.

S5. Cytotoxicity Assays

Testing compound stock solution, 20 mM in DMSO: 20 mM DMSO stock solutions were diluted for 20- fold in EMEM medium, followed by 1:10x dilution in EMEM with 5% DMSO, and addition of 11.1 µL of the 10- fold diluted compounds into the cells with 100 µL medium, final DMSO concentration in each well is 0.5%. The cells treated with medium with 0.5% DMSO served as DMSO control. The final compound concentrations were 100 µM, in duplicate. Gemcitabine top concentration for both PANC-1 and U87-MG was 100 µM, followed by 8 points of 5-fold serial dilutions in EMEM medium. The tenth point contained no compounds, only medium served as DMSO control. In addition, a single point of 50 µM Gemcitabine, in quadruplet, for both PANC-1 and U87-MG, was tested in the plate treated with compounds. Reference compound Cytarabine stock was 2 mM in DMSO. The top concentration for both PANC-1 and U87-MG was 100 µM, followed by 8 points of 5-fold serial dilutions in EMEM medium with 0.5% DMSO. The tenth point contained no compounds, only medium with 0.5% DMSO served as DMSO control.

The cells were seeded in a density of 3000 cells/well/100 µL for both PANC-1 cells and U87-MG cells on 96-well black plates with clear bottom and clear plates, respectively, and incubated at 37 °C with 5% CO2 for 24 h. On day two, 10 µL of the serial diluted compounds were added onto the plate with cells in duplicate. The top concentration of the testing compound was 100 µM, and 10 µM, respectively, in duplicate. The final DMSO concentration in the assay for all wells was 0.5%. The top concentrations of references were indicated above #8. The cells incubated with the compounds for three days at 37 °C with 5% CO_2. After the cells were treated with compounds for 72h, cell viabilities were determined using PrestoBlue™ HS Cell Viability Reagent (Invitrogen, catalog# P50200). Briefly, 10 µL of PrestoBlue™ HS Cell Viability Reagent was added to cells in the 96-well black plates with clear bottom and was incubated at 37 °C for 10 minutes. The fluorescence signal, which was positively correlated with viable cell counts, was measured with fluorescence excitation/emission maxima: 560/590 nm on an EnSpire plate reader. The fluorescence signals from 4 wells containing only medium were used as background which was subtracted from all other testing wells. The wells treated with only 0.5% DMSO or medium only (for Gemcitabine) were DMSO or medium control, were set as 100% of cell viability. All the wells with treated cells will be as % of the Control.

S5.1 Control viability assays

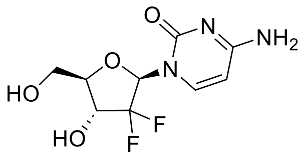

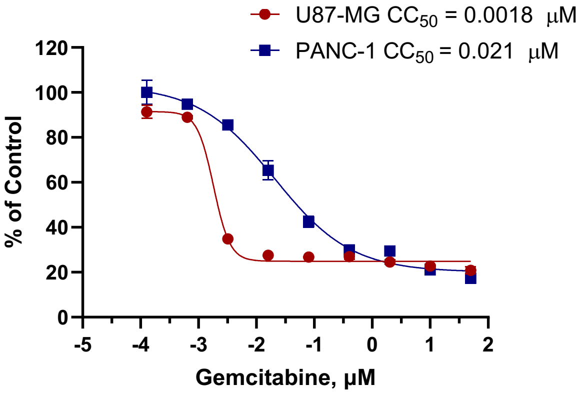

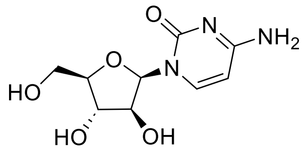

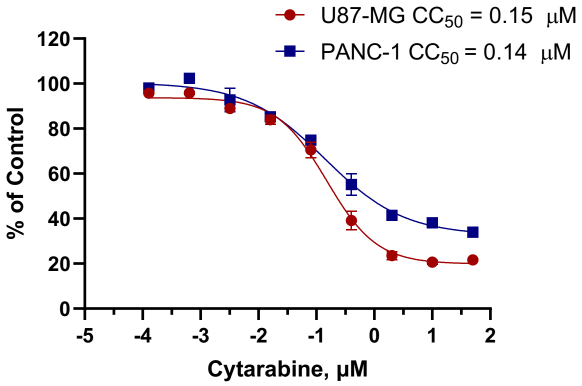

S6. References

1 M. Cong, G. Xu, S. Yang, J. Zhang, W. Zhang, D. Dhumal, E. Laurini, K. Zhang, Y. Xia, S. Pricl, L. Peng and W. Zhao, *Chinese Chemical Letters*, 2022, 33, 2481–2485.

2 C. F. Ainsworth, S. S. Danyluk and M. MacCoss, *Nucleosides Nucleotides*, 1983, 2, 435–452.

3 M. A. Arai, Y. Yamaguchi and M. Ishibashi, *Org Biomol Chem*, 2017, 15, 5025–5032.

4 E. Manzo, C. Gallo, R. Sartorius, G. Nuzzo, A. Sardo, P. De Berardinis, A. Fontana and A. Cutignano, *Marine Drugs 2019, Vol. 17, Page 103*, 2019, 17, 103.

5 B. Rønne Kristensen and C. M. Pedersen, *European J Org Chem*, 2023, 26, e202300213.

6 T. B. Cai, D. Lu, X. Tang, Y. Zhang, M. Landerholm and P. G. Wang, *Journal of Organic Chemistry*, 2005, 70, 3518–3524.

7 P. Greimel, M. Lapeyre, Y. Nagatsuka, Y. Hirabayashi and Y. Ito, *Bioorg Med Chem*, 2008, 16, 7210–7217.

8 P. Greimel, M. Lapeyre, Y. Nagatsuka, Y. Hirabayashi and Y. Ito, *Bioorg Med Chem*, 2008, 16, 7210–7217.
