# Supplementary material for: An H‑Phosphonate-Mediated Synthesis of Nucleotide-Pyranose Glycoconjugates
Source: Org Lett. 2026 Jun 10;28(24):7913–7. doi: 10.1021/acs.orglett.6c02135 (PMC13288873; doi:10.1021/acs.orglett.6c02135)
Supplement: Supplementary file 2 [file ol6c02135_si_002.pdf]

Supporting Information

An *H*-Phosphonate-mediated Synthesis of Nucleotide-Pyranose Glycoconjugates

Thibault Guillaume,[1] Ningwu Huang,[2] Mark Smith[2] and Gavin J. Miller[1,3]*

[1] School of Chemical and Physical Sciences, Keele University, Keele, Staffordshire, ST5 5BG, UK.

[2] Riboscience LLC, 428 Oakmead Pkwy, Sunnyvale, CA 94085, USA.

[3] Manchester Institute of Biotechnology & Department of Chemistry, University of Manchester, Manchester, M1 7DN, UK.

*gavin.miller@manchester.ac.uk

Table of Contents

Figure S1 ^{1}CH NMR (400 MHz, $CDCl_3$): 3',5'-O-tertButyldimethylsilyl-N-4-benzoyl-2',2'-difluorocytidine S1 8

Figure S2 ^{13}C NMR (101 MHz, $CDCl_3$): 3',5'-O-tertButyldimethylsilyl-N-4-benzoyl-2',2'-difluorocytidine S1 9

Figure S3 ^{19}F NMR (377 MHz, $CDCl_3$): 3',5'-O-tertButyldimethylsilyl-N-4-benzoyl-2',2'-difluorocytidine S1 10

Figure S4 ^{1}H NMR (400 MHz, $CDCl_3$): 3'-*O*-*tert*Butyldimethylsilyl-*N*-4-benzoyl-2',2'-difluorocytidine 1 11

Figure S5 ^{13}C NMR (101 MHz, $CDCl_3$): 3'-*O*-*tert*Butyldimethylsilyl-*N*-4-benzoyl-2',2'-difluorocytidine 1 12

Figure S6 ^{19}F NMR (377 MHz, $CDCl_3$): 3'-*O*-*tert*Butyldimethylsilyl-*N*-4-benzoyl-2',2'-difluorocytidine 1 13

Figure S7 ^{1}H NMR (400 MHz, $CDCl_3$): 2',3'-5'-Tri-*O*-*tert*butyldimethylsilyl -*N*-4-benzoyl-arabinocytidine S3 14

Figure S8 ^{13}C NMR (101 MHz, $CDCl_3$): 2',3'-5'-Tri-*O*-*tert*butyldimethylsilyl-*N*-4-benzoyl-arabinocytidine S3 15

Figure S9 ^{1}H NMR (400 MHz, $CDCl_3$): 2',3'-Di-*O*-*tert*butyldimethylsilyl-*N*-4-benzoyl-arabinocytidine 2 16

Figure S10 ^{13}C NMR (101 MHz, $CDCl_3$): 2',3'-Di-*O*-*tert*butyldimethylsilyl-*N*-4-benzoyl-arabinocytidine 2 17

Figure S11 ^{1}H NMR (400 MHz, $CDCl_3$): 1,2,3,4-Tetra-*O*-acetyl-6-*O*-trityl-β-D-glucopyranoside S5 18

Figure S12 ^{13}C NMR (101 MHz, $CDCl_3$): 1,2,3,4-Tetra-*O*-acetyl-6-*O*-trityl-β-D-glucopyranoside S5 19

Figure S13 ^{1}H NMR (400 MHz, $CDCl_3$): 1,2,3,4-Tetra-*O*-acetyl-β-D-glucopyranoside 5 20

Figure S14 ^{13}C NMR (101 MHz, $CDCl_3$): 1,2,3,4-Tetra-*O*-acetyl-β-D-glucopyranoside 5 21

Figure S15 ^{1}H NMR (400 MHz, $CDCl_3$): 1,2,3,4-Tetra-*O*-acetyl-6-*O*-trityl-α/β-D-galactopyranoside S7 22

Figure S16 ^{13}C NMR (101 MHz, $CDCl_3$): 1,2,3,4-Tetra-*O*-acetyl-6-*O*-trityl-α/β-D-galactopyranoside S7 23

Figure S17 ^{1}H NMR (400 MHz, $CDCl_3$): 1,2,3,4-Tetra-*O*-acetyl-α/β-D-galactopyranoside 16 24

Figure S18 ^{13}C NMR (101 MHz, $CDCl_3$): 1,2,3,4-Tetra-*O*-acetyl-α/β-D-galactopyranoside 16 25

Figure S19 ^{1}H NMR (400 MHz, $CDCl_3$): 2,3,4,6-Tetra-*O*-acetyl-α/β-D-glucopyranoside 10 ... 26

Figure S20 ^{13}C NMR (101 MHz, $CDCl_3$): 2,3,4,6-Tetra-*O*-acetyl-α/β-D-glucopyranoside 10 .. 27

Figure S21 ^{1}H NMR (400 MHz, $CDCl_3$): 2,3,4,6-Tetra-*O*-acetyl-α/β-D-galactopyranoside 21 28

Figure S22 ^{13}C NMR (101 MHz, $CDCl_3$): 2,3,4,6-Tetra-*O*-acetyl-α/β-D-galactopyranoside 21 29

Figure S23 ^{1}H NMR (400 MHz, $CDCl_3$): 3'-*O*-*tert*Butyldimethylsilyl-*N*-4-benzoyl-2',2'-difluorocytidine-5'-*O*-hydrogenphosphonate triethylammonium salt 3 30

Figure S24 ^{13}C NMR (101 MHz, $CDCl_3$): 3'-*O*-*tert*Butyldimethylsilyl-*N*-4-benzoyl-2',2'-difluorocytidine-5'-*O*-hydrogenphosphonate triethylammonium salt 3 31

Figure S25 ^{19}F {^{1}H} NMR (377 MHz, $CDCl_3$): 3'-*O*-*tert*Butyldimethylsilyl-*N*-4-benzoyl-2',2'-difluorocytidine-5'-*O*-hydrogenphosphonate triethylammonium salt 3 32

Figure S26 ^{31}P {^{1}H} NMR (162 MHz, $CDCl_3$): 3'-*O*-*tert*Butyldimethylsilyl-*N*-4-benzoyl-2',2'-difluorocytidine-5'-*O*-hydrogenphosphonate triethylammonium salt 3 33

Figure S27 ^{1}H NMR (400 MHz, $CDCl_3$): 2',3'-*O*-*tert*Butyldimethylsilyl-*N*-4-benzoyl-arabinocytidine-5'-*O*-hydrogenphosphonate triethylammonium salt 4 34

Figure S28 ^{13}C NMR (101 MHz, $CDCl_3$): 2',3'-*O*-*tert*Butyldimethylsilyl-*N*-4-benzoyl-arabinocytidine-5'-*O*-hydrogenphosphonate triethylammonium salt 4 35

Figure S29 ^{31}P NMR (162 MHz, $CDCl_3$): 2',3'-*O*-*tert*Butyldimethylsilyl-*N*-4-benzoyl-arabinocytidine-5'-*O*-hydrogenphosphonate triethylammonium salt 4 36

Figure S30 ^{1}H NMR (400 MHz, $CDCl_3$): 2',3'-*O*-*tert*Butyldimethylsilyl-*N*-4-benzoyl-arabinocytidine-5'-*O*-[6''-*O*-(1'',2'',3'',4''-tetra-*O*-actetyl-β-D-glucopyranose)]-hydrogenphosphonate S10 37

Figure S31 ^{13}C NMR (101 MHz, $CDCl_3$): 2',3'-*O*-*tert*Butyldimethylsilyl-*N*-4-benzoyl-arabinocytidine-5'-*O*-[6''-*O*-(1'',2'',3'',4''-tetra-*O*-actetyl-β-D-glucopyranose)]-hydrogenphosphonate S10 38

Figure S32 ^{31}P {^1H} NMR (162 MHz, $CDCl_3$): 2',3'-*O*-*tert*Butyldimethylsilyl-*N*-4-benzoyl-arabinocytidine-5'-*O*-[6''-*O*-(1'',2'',3'',4''-tetra-*O*-actetyl-β-D-glucopyranose)]-hydrogenphosphonate S10 39

Figure S33 ^{1}H NMR (400 MHz, $CDCl_3$): 2',3'-*O*-*tert*Butyldimethylsilyl-*N*-4-benzoyl-arabinocytidine-5'-*O*-[6''-*O*-(1'',2'',3'',4''-tetra-*O*-acetyl-β-D-glucopyranose)]-phosphate triethylammonium salt 6 40

Figure S34 ^{13}C NMR (101 MHz, $CDCl_3$): 2',3'-*O*-*tert*Butyldimethylsilyl-*N*-4-benzoyl-arabinocytidine-5'-*O*-[6''-*O*-(1'',2'',3'',4''-tetra-*O*-acetyl-β-D-glucopyranose)]-phosphate triethylammonium salt 6 41

Figure S35 ^{31}P NMR (162 MHz, $CDCl_3$): 2',3'-*O*-*tert*Butyldimethylsilyl-*N*-4-benzoyl-arabinocytidine-5'-*O*-[6''-*O*-(1'',2'',3'',4''-tetra-*O*-acetyl-β-D-glucopyranose)]-phosphate triethylammonium salt 6 42

Figure S36 ^{1}H NMR (400 MHz, $CDCl_3$): 3'-*O*-*tert*Butyldimethylsilyl-*N*-4-benzoyl-2'-deoxy-2',2'-difluorocytidine-5'-*O*-[6''-*O*-(1'',2'',3'',4''-tetra-*O*-acetyl-β-D-glucopyranose)]-phosphate triethylammonium salt 7 43

Figure S37 ^{13}C NMR (101 MHz, $CDCl_3$): 3'-*O*-*tert*Butyldimethylsilyl-*N*-4-benzoyl-2'-deoxy-2',2'-difluorocytidine-5'-*O*-[6''-*O*-(1'',2'',3'',4''-tetra-*O*-acetyl-β-D-glucopyranose)]-phosphate triethylammonium salt 7 44

Figure S38 ^{19}F {^1H} NMR (377 MHz, $CDCl_3$): 3'-*O*-*tert*Butyldimethylsilyl-*N*-4-benzoyl-2'-deoxy-2',2'-difluorocytidine-5'-*O*-[6''-*O*-(1'',2'',3'',4''-tetra-*O*-acetyl-β-D-glucopyranose)]-phosphate triethylammonium salt 7 45

Figure S39 ^{31}P {^1H} NMR (162 MHz, $CDCl_3$): 3'-*O*-*tert*Butyldimethylsilyl-*N*-4-benzoyl-2'-deoxy-2',2'-difluorocytidine-5'-*O*-[6''-*O*-(1'',2'',3'',4''-tetra-*O*-acetyl-β-D-glucopyranose)]-phosphate triethylammonium salt 7 46

Figure S40 ^{1}H NMR (400 MHz, D_2O): Arabinocytidine-5'-*O*-(6''-*O*-α/β-D-glucopyranose)-phosphate sodium salt 8 47

Figure S41 ^{13}C NMR (101 MHz, D_2O): Arabinocytidine-5'-*O*-(6''-*O*-α/β-D-glucopyranose)-phosphate sodium salt 8 48

Figure S42 ^{31}P NMR (162 MHz, D_2O): Arabinocytidine-5'-*O*-(6''-*O*-α/β-D-glucopyranose)-phosphate sodium salt 8 49

Figure S43 ^{1}H NMR (400 MHz, D_2O): 2'-Deoxy-2',2'-difluorocytidine-5'-*O*-(6''-*O*-β-D-glucopyranose)-phosphate sodium salt 9 50

Figure S44 ^{13}C NMR (101 MHz, D_2O): 2'-Deoxy-2',2'-difluorocytidine-5'-*O*-(6''-*O*-β-D-glucopyranose)-phosphate sodium salt 9 51

Figure S45 ^{19}F {^1H} NMR (377 MHz, D_2O): 2'-Deoxy-2',2'-difluorocytidine-5'-*O*-(6''-*O*-β-D-glucopyranose)-phosphate sodium salt 9 52

Figure S46 ^{31}P {^{1}H} NMR (162 MHz, D_2O): 2'-Deoxy-2',2'-difluorocytidine-5'-*O*-(6''-*O*-β-D-glucopyranose)-phosphate sodium salt 9 53

Figure S47 ^{1}H NMR (400 MHz, $CDCl_3$): 2,3,4,6-Tetra-*O*-acetyl-β-D-glucopyranosyl-1-*O*-hydrogenphosphonate triethylammonium salt 11 54

Figure S48 ^{13}C NMR (101 MHz, $CDCl_3$): 2,3,4,6-Tetra-*O*-acetyl-β-D-glucopyranosyl-1-*O*-hydrogenphosphonate triethylammonium salt 11 55

Figure S49 ^{31}P NMR (162 MHz, $CDCl_3$): 2,3,4,6-Tetra-*O*-acetyl-β-D-glucopyranosyl-1-*O*-hydrogenphosphonate triethylammonium salt 11 56

Figure S50 ^{1}H NMR (400 MHz, $CDCl_3$): 2',3'-*O*-*tert*Butyldimethylsilyl-*N*-4-benzoyl-arabinocytidine-5'-*O*-[1''-*O*-(2'',3'',4'',6''-tetra-*O*-acetyl-α/β-D-glucopyranose)]-phosphate triethylammonium salt 12 57

Figure S51 ^{13}C NMR (101 MHz, $CDCl_3$): 2',3'-*O*-*tert*Butyldimethylsilyl-*N*-4-benzoyl-arabinocytidine-5'-*O*-[1''-*O*-(2'',3'',4'',6''-tetra-*O*-acetyl-α/β-D-glucopyranose)]-phosphate triethylammonium salt 12 58

Figure S52 ^{31}P {H^{1}} NMR (162 MHz, $CDCl_3$): 2',3'-*O*-*tert*Butyldimethylsilyl-*N*-4-benzoyl-arabinocytidine-5'-*O*-[1''-*O*-(2'',3'',4'',6''-tetra-*O*-acetyl-α/β-D-glucopyranose)]-phosphate triethylammonium salt 12 59

Figure S53 ^{1}H NMR (400 MHz, $CDCl_3$): 3'-*O*-*tert*Butyldimethylsilyl-*N*-4-benzoyl-2'-deoxy-2',2'-difluorocytidine-5'-*O*-[1''-*O*-(2'',3'',4'',6''-tetra-*O*-acetyl-α/β-D-glucopyranose)]-phosphate triethylammonium salt 13 60

Figure S54 ^{13}C NMR (101 MHz, $CDCl_3$): 3'-*O*-*tert*Butyldimethylsilyl-*N*-4-benzoyl-2'-deoxy-2',2'-difluorocytidine-5'-*O*-[1''-*O*-(2'',3'',4'',6''-tetra-*O*-acetyl-α/β-D-glucopyranose)]-phosphate triethylammonium salt 13 61

Figure S55 ^{19}F {^{1}H} NMR (377 MHz, $CDCl_3$): 3'-*O*-*tert*Butyldimethylsilyl-*N*-4-benzoyl-2'-deoxy-2',2'-difluorocytidine-5'-*O*-[1''-*O*-(2'',3'',4'',6''-tetra-*O*-acetyl-α/β-D-glucopyranose)]-phosphate triethylammonium salt 13 62

Figure S56 ^{31}P NMR (162 MHz, $CDCl_3$): 3'-*O*-*tert*Butyldimethylsilyl-*N*-4-benzoyl-2'-deoxy-2',2'-difluorocytidine-5'-*O*-[1''-*O*-(2'',3'',4'',6''-tetra-*O*-acetyl-α/β-D-glucopyranose)]-phosphate triethylammonium salt 13 63

Figure S57 ^{1}H NMR (400 MHz, D_2O): Arabinocytidine-5'-*O*-(1''-*O*-α/β-D-glucopyranose)-phosphate sodium salt 14 64

Figure S58 ^{13}C NMR (101 MHz, D_2O): Arabinocytidine-5'-*O*-(1''-*O*-α/β-D-glucopyranose)-phosphate sodium salt 14 65

Figure S59 ^{31}P NMR (162 MHz, D_2O): Arabinocytidine-5'-*O*-(1''-*O*-α/β-D-glucopyranose)-phosphate sodium salt 14 66

Figure S60 ^{1}H NMR (400 MHz, D_2O): 2'-Deoxy-2',2'-difluorocytidine-5'-*O*-(1''-*O*-α/β-D-glucopyranose)-phosphate sodium salt 15 67

Figure S61 ^{19}F {^{1}H} NMR (377 MHz, D_2O): 2'-Deoxy-2',2'-difluorocytidine-5'-*O*-(1''-*O*-α/β-D-glucopyranose)-phosphate sodium salt 15 68

Figure S62 ^{31}P {^{1}H} NMR (162 MHz, D_2O): 2’-Deoxy-2’,2’-difluorocytidine-5'-*O*-(1''-*O*-α/β-D-glucopyranose)-phosphate sodium salt 15 69

Figure S63 ^{1}H NMR (400 MHz, $CDCl_3$): 1,2,3,4-Tetra-*O*-acetyl-β-D-galctopyranosyl-6-*O*-hydrogenphosphonate triethylammonium salt 17 70

Figure S64 ^{13}C NMR (101 MHz, $CDCl_3$): 1,2,3,4-Tetra-*O*-acetyl-β-D-galctopyranosyl-6-*O*-hydrogenphosphonate triethylammonium salt 17 71

Figure S65 ^{31}P NMR (162 MHz, $CDCl_3$): 1,2,3,4-Tetra-*O*-acetyl-β-D-galctopyranosyl-6-*O*-hydrogenphosphonate triethylammonium salt 17 72

Figure S66 ^{1}H NMR (400 MHz, $CDCl_3$): 2',3'-*O*-*tert*Butyldimethylsilyl-*N*-4-benzoyl-2’-deoxy-2’,2’-difluorocytidine-5'-*O*-[6''-*O*-(1'',2'',3'',4''-tetra-*O*-acetyl-β-D-galactopyranose)]-phosphate triethylammonium salt 18 73

Figure S67 ^{13}C NMR (101 MHz, $CDCl_3$): 2',3'-*O*-*tert*Butyldimethylsilyl-*N*-4-benzoyl-2’-deoxy-2’,2’-difluorocytidine-5'-*O*-[6''-*O*-(1'',2'',3'',4''-tetra-*O*-acetyl-β-D-galactopyranose)]-phosphate triethylammonium salt 18 74

Figure S68 ^{19}F NMR (377 MHz, $CDCl_3$): 2',3'-*O*-*tert*Butyldimethylsilyl-*N*-4-benzoyl-2’-deoxy-2’,2’-difluorocytidine-5'-*O*-[6''-*O*-(1'',2'',3'',4''-tetra-*O*-acetyl-β-D-galactopyranose)]-phosphate triethylammonium salt 18 75

Figure S69 ^{31}P {^{1}H} NMR (162 MHz, $CDCl_3$): 2',3'-*O*-*tert*Butyldimethylsilyl-*N*-4-benzoyl-2’-deoxy-2’,2’-difluorocytidine-5'-*O*-[6''-*O*-(1'',2'',3'',4''-tetra-*O*-acetyl-β-D-galactopyranose)]-phosphate triethylammonium salt 18 76

Figure S70 ^{1}H NMR (400 MHz, $CDCl_3$): 2',3'-*O*-*tert*Butyldimethylsilyl-*N*-4-benzoyl-arabinocytidine-5'-*O*-[6''-*O*-(1'',2'',3'',4''-tetra-*O*-acetyl-α/β-D-galactopyranose)]-phosphate triethylammonium salt 19 77

Figure S71 ^{13}C NMR (101 MHz, $CDCl_3$): 2',3'-*O*-*tert*Butyldimethylsilyl-*N*-4-benzoyl-arabinocytidine-5'-*O*-[6''-*O*-(1'',2'',3'',4''-tetra-*O*-acetyl-α/β-D-galactopyranose)]-phosphate triethylammonium salt 19 78

Figure S72 ^{31}P {^{1}H} NMR (162 MHz, $CDCl_3$): 2',3'-*O*-*tert*Butyldimethylsilyl-*N*-4-benzoyl-arabinocytidine-5'-*O*-[6''-*O*-(1'',2'',3'',4''-tetra-*O*-acetyl-α/β-D-galactopyranose)]-phosphate triethylammonium salt 19 79

Figure S73 ^{1}H NMR (400 MHz, D_2O): 2’-Deoxy-2’,2’-difluorocytidine-5'-*O*-(6''-*O*-α/β-D-galactopyranose)-phosphate sodium salt 20 80

Figure S74 ^{13}C NMR (101 MHz, D_2O): 2’-Deoxy-2’,2’-difluorocytidine-5'-*O*-(6''-*O*-α/β-D-galactopyranose)-phosphate sodium salt 20 81

Figure S75 ^{19}F NMR (377 MHz, D_2O): 2’-Deoxy-2’,2’-difluorocytidine-5'-*O*-(6''-*O*-α/β-D-galactopyranose)-phosphate sodium salt 20 82

Figure S76 ^{31}P {1H} NMR (162 MHz, D_2O): 2’-Deoxy-2’,2’-difluorocytidine-5'-*O*-(6''-*O*-α/β-D-galactopyranose)-phosphate sodium salt 20 83

Figure S77 ^{1}H NMR (400 MHz, $CDCl_3$): 2,3,4,6-Tetra-*O*-acetyl-α/β-D-galactopyranosyl-1-*O*-hydrogenphosphonate triethylammonium salt 22 84

Figure S78 ^{31}P NMR (162 MHz, $CDCl_3$): 2,3,4,6-Tetra-*O*-acetyl-α/β-D-galactopyranosyl-1-*O*-hydrogenphosphonate triethylammonium salt 22 85

Figure S79 ^{1}H NMR (400 MHz, $CDCl_3$): 3'-*O*-*tert*Butyldimethylsilyl-*N*-4-benzoyl-2'-deoxy-2',2'-difluorocytidine-5'-*O*-[1''-*O*-(2'',3'',4'',6''-tetra-*O*-acetyl-α/β-D-galactopyranose)]-phosphate triethylammonium salt 23 86

Figure S80 ^{13}C NMR (101 MHz, $CDCl_3$): 3'-*O*-*tert*Butyldimethylsilyl-*N*-4-benzoyl-2'-deoxy-2',2'-difluorocytidine-5'-*O*-[1''-*O*-(2'',3'',4'',6''-tetra-*O*-acetyl-α/β-D-galactopyranose)]-phosphate triethylammonium salt 23 87

Figure S81 ^{19}F {^{1}H} NMR (377 MHz, $CDCl_3$): 3'-*O*-*tert*Butyldimethylsilyl-*N*-4-benzoyl-2'-deoxy-2',2'-difluorocytidine-5'-*O*-[1''-*O*-(2'',3'',4'',6''-tetra-*O*-acetyl-α/β-D-galactopyranose)]-phosphate triethylammonium salt 23 88

Figure S82 ^{31}P {^{1}H} NMR (162 MHz, $CDCl_3$): 3'-*O*-*tert*Butyldimethylsilyl-*N*-4-benzoyl-2'-deoxy-2',2'-difluorocytidine-5'-*O*-[1''-*O*-(2'',3'',4'',6''-tetra-*O*-acetyl-α/β-D-galactopyranose)]-phosphate triethylammonium salt 23 89

Figure S83 ^{1}H NMR (400 MHz, $CDCl_3$): 2',3'-*O*-*tert*Butyldimethylsilyl-*N*-4-benzoyl-arabinocytidine-5'-*O*-[1''-*O*-(2'',3'',4'',6''-tetra-*O*-acetyl-α/β-D-galactopyranose)]-phosphate triethylammonium salt 24 90

Figure S84 ^{13}C NMR (101 MHz, $CDCl_3$): 2',3'-*O*-*tert*Butyldimethylsilyl-*N*-4-benzoyl-arabinocytidine-5'-*O*-[1''-*O*-(2'',3'',4'',6''-tetra-*O*-acetyl-α/β-D-galactopyranose)]-phosphate triethylammonium salt 24 91

Figure S85 ^{31}P {^{1}H} NMR (162 MHz, $CDCl_3$): 2',3'-*O*-*tert*Butyldimethylsilyl-*N*-4-benzoyl-arabinocytidine-5'-*O*-[1''-*O*-(2'',3'',4'',6''-tetra-*O*-acetyl-α/β-D-galactopyranose)]-phosphate triethylammonium salt 24 92

Figure S86 ^{1}H NMR (400 MHz, D_2O): 2'-Deoxy-2',2'-difluorocytidine-5'-*O*-(1''-*O*-α/β-D-galactopyranose)-phosphate sodium salt 25 93

Figure S87 ^{13}C NMR (101 MHz, D_2O): 2'-Deoxy-2',2'-difluorocytidine-5'-*O*-(1''-*O*-α/β-D-galactopyranose)-phosphate sodium salt 25 94

Figure S88 ^{19}F {^{1}H} NMR (377 MHz, D_2O): 2'-Deoxy-2',2'-difluorocytidine-5'-*O*-(1''-*O*-α/β-D-galactopyranose)-phosphate sodium salt 25 95

Figure S89 ^{31}P {^{1}H} NMR (162 MHz, D_2O): 2'-Deoxy-2',2'-difluorocytidine-5'-*O*-(1''-*O*-α/β-D-galactopyranose)-phosphate sodium salt 25 96

Figure S90 ^{1}H NMR (400 MHz, D_2O): Arabinocytidine-5'-*O*-(1''-*O*-α/β-D-galactopyranose)-phosphate sodium salt 26 97

Figure S91 ^{13}C NMR (101 MHz, D_2O): Arabinocytidine-5'-*O*-(1''-*O*-α/β-D-galactopyranose)-phosphate sodium salt 26 98

Figure S92 ^{31}P {^{1}H} NMR (400 MHz, D_2O): Arabinocytidine-5'-*O*-(1''-*O*-α/β-D-galactopyranose)-phosphate sodium salt 26 99

Figure S93 Analytical HPLC traces of Arabinocytidine-5'-*O*-(6''-*O*-α/β-D-glucopyranose)-phosphate sodium salt 8 100

Figure S94 Analytical HPLC traces of 2’-Deoxy-2’,2’-difluorocytidine-5'-*O*-(6''-*O*-β-D-glucopyranose)-phosphate sodium salt 9..101

Figure S95 Analytical HPLC traces of Arabinocytidine-5'-*O*-(1''-*O*-α/β-D-glucopyranose)-phosphate sodium salt 14 102

Figure S96 Analytical HPLC traces of 2’-Deoxy-2’,2’-difluorocytidine-5'-*O*-(1''-*O*-α/β-D-glucopyranose)-phosphate sodium salt 15..103

Figure S97 Analytical HPLC traces of 2’-Deoxy-2’,2’-difluorocytidine-5'-*O*-(6''-*O*-α/β-D-galactopyranose)-phosphate sodium salt 20..104

Figure S98 Analytical HPLC traces of 2’-Deoxy-2’,2’-difluorocytidine-5'-*O*-(1''-*O*-α/β-D-galactopyranose)-phosphate sodium salt 25..105

Figure S99 Analytical HPLC traces of Arabinocytidine-5'-*O*-(1''-*O*-α/β-D-galactopyranose)-phosphate sodium salt 26 107

Figure S1 [1]CH NMR (400 MHz, $CDCl_3$): 3',5'-O-tertButyldimethylsilyl-N-4-benzoyl-2',2'-difluorocytidine S1

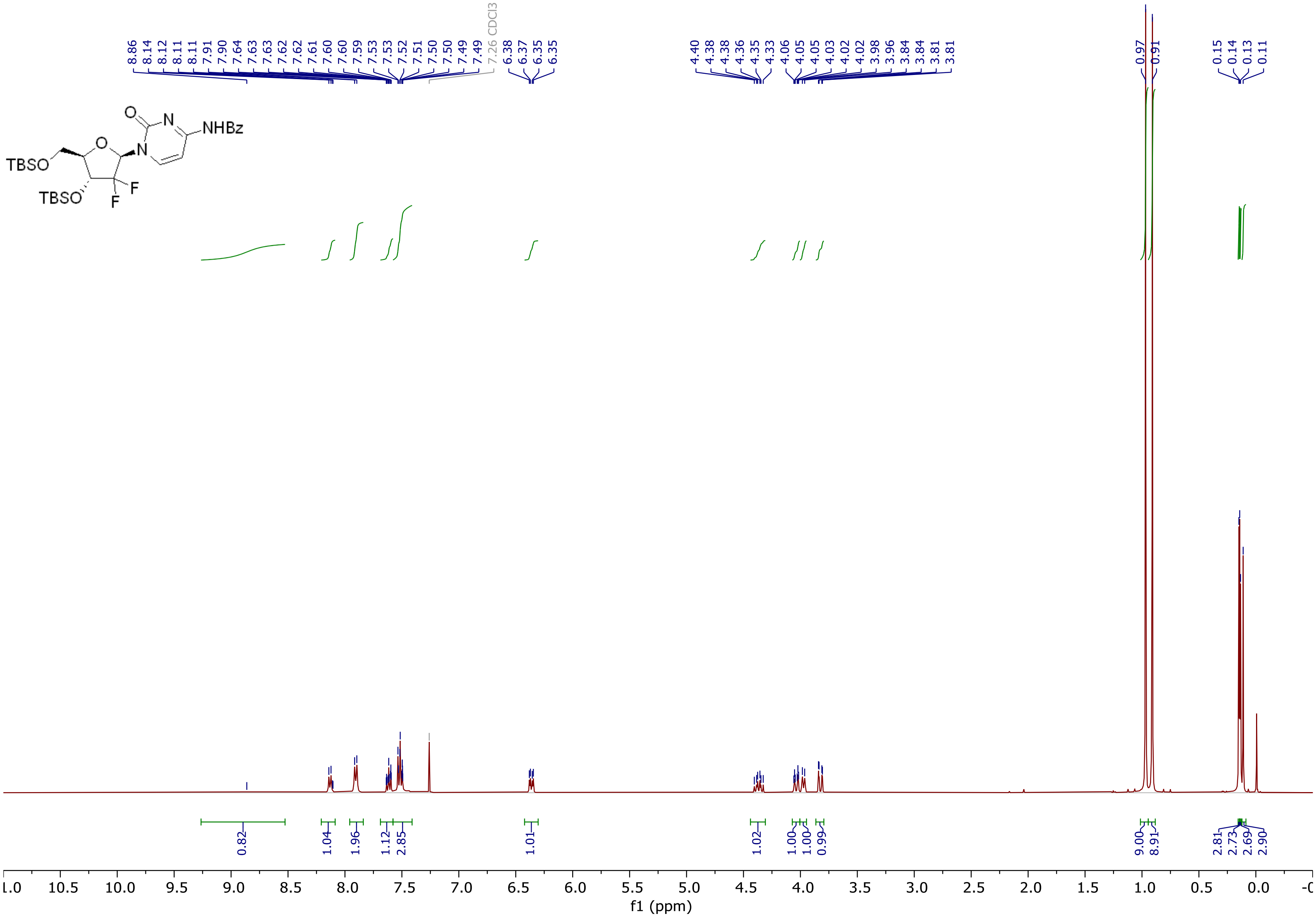

Figure S2 ^{13}C NMR (101 MHz, $CDCl_3$): 3',5'-O-tertButyldimethylsilyl-N-4-benzoyl-2',2'-difluorocytidine S1

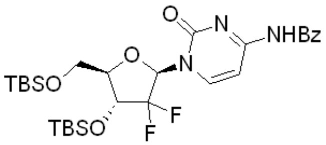

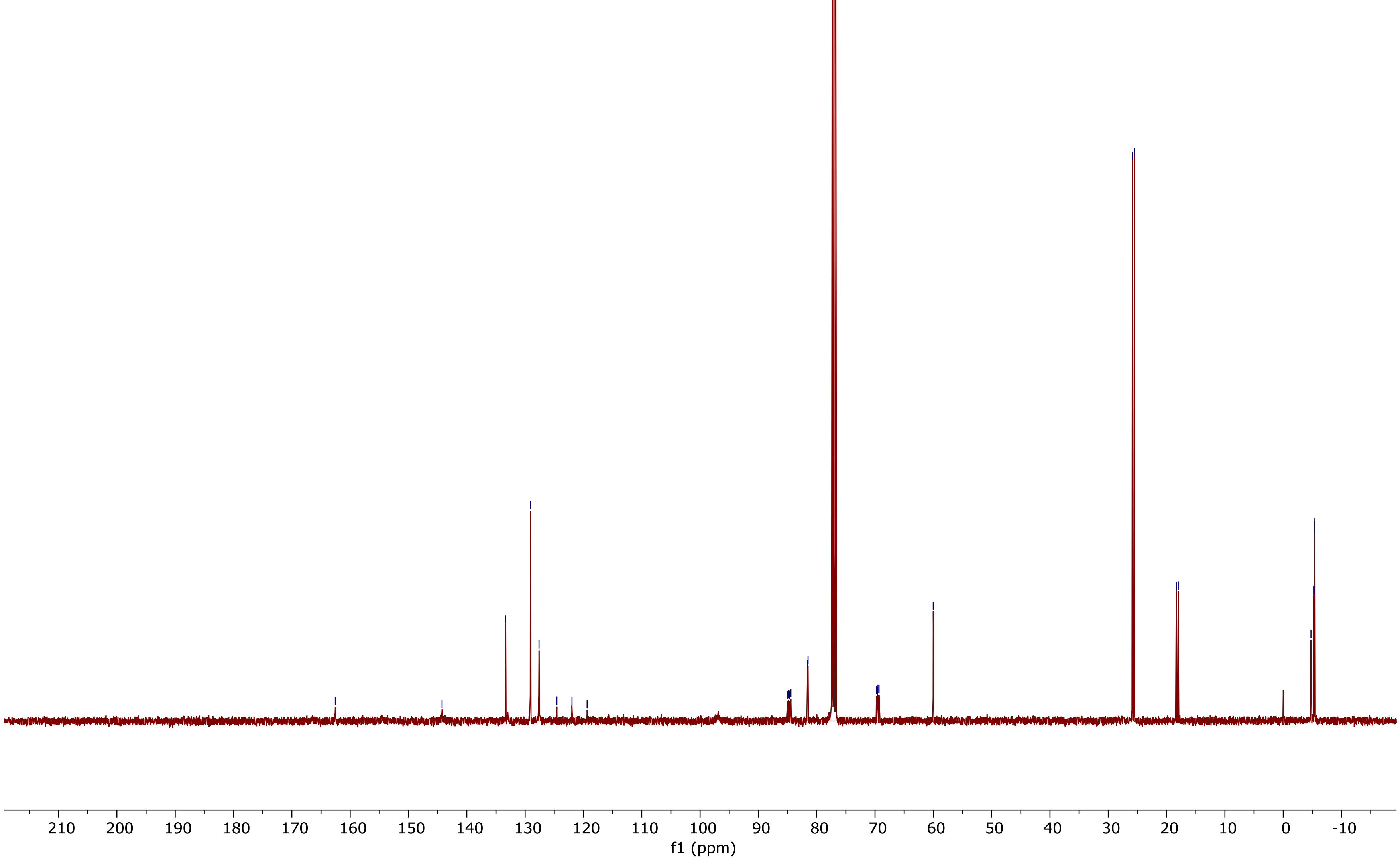

Figure S3 ^{19}F NMR (377 MHz, $CDCl_3$): 3',5'-O-tertButyldimethylsilyl-N-4-benzoyl-2',2'-difluorocytidine S1

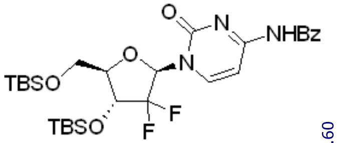

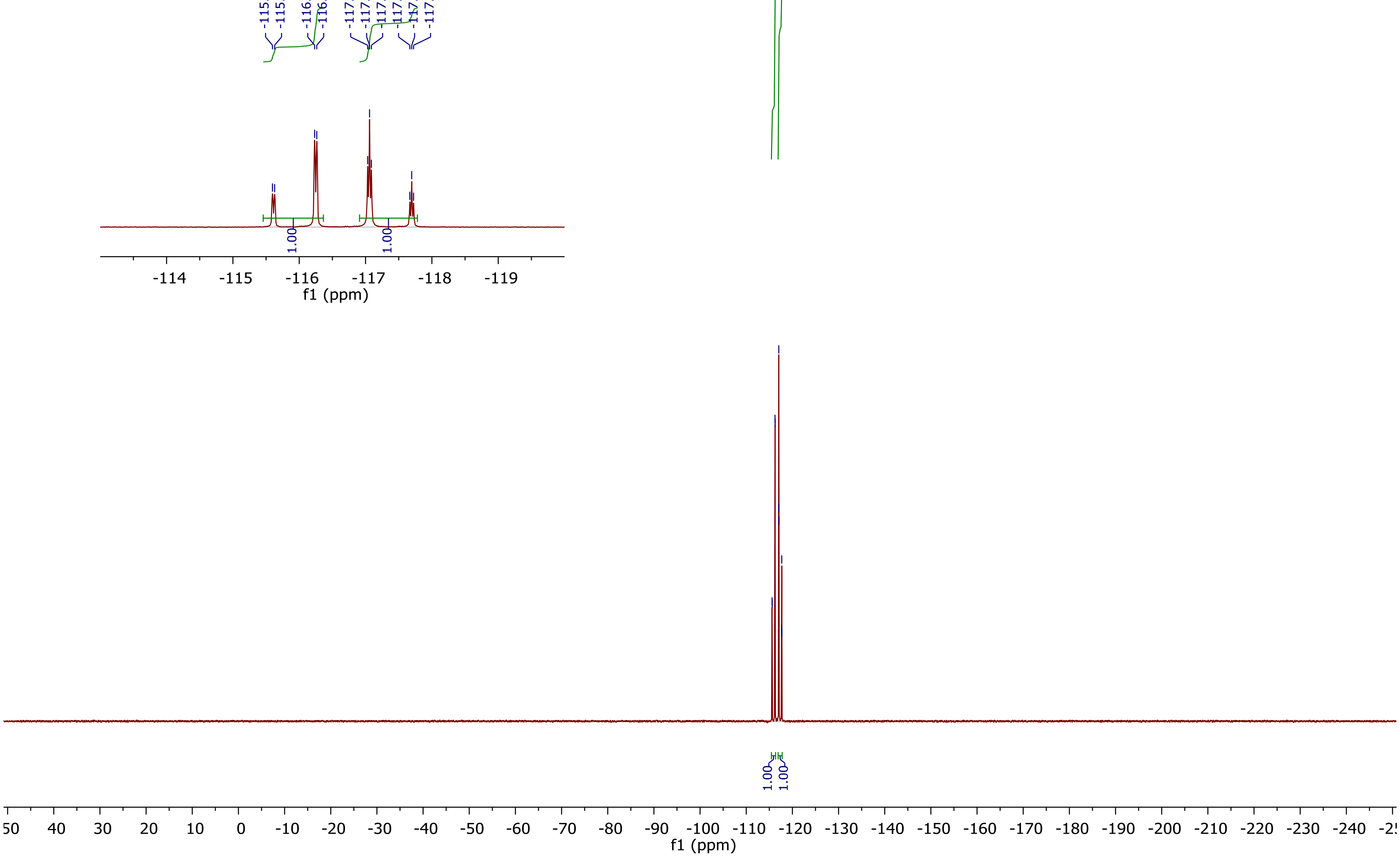

Figure S4 ^{1}H NMR (400 MHz, $CDCl_3$): 3'-*O*-*tert*Butyldimethylsilyl-*N*-4-benzoyl-2',2'-difluorocytidine 1

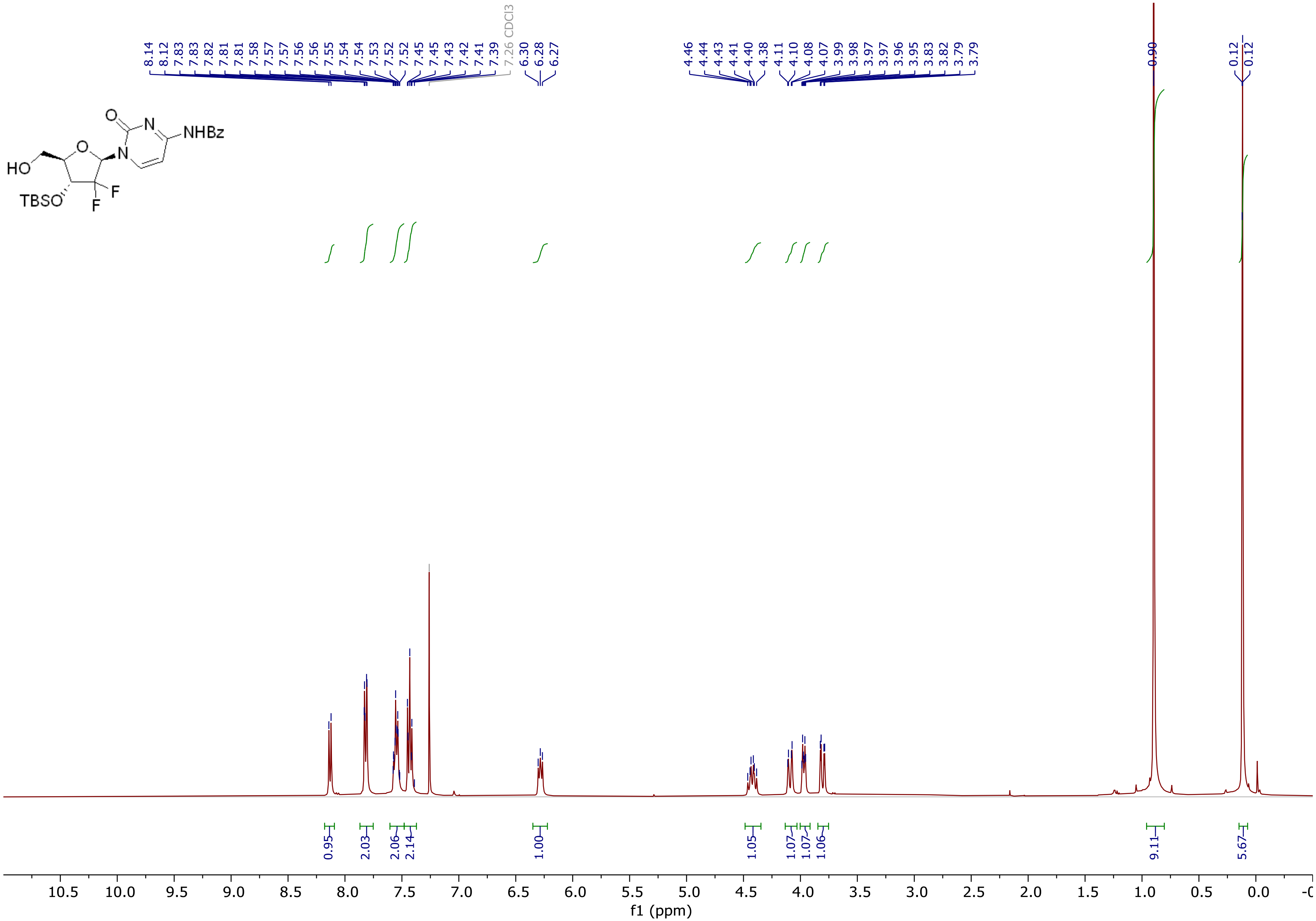

Figure S5 ^{13}C NMR (101 MHz, CDCl$_3$): 3'-*O*-*tert*Butyldimethylsilyl-*N*-4-benzoyl-2',2'-difluorocytidine 1

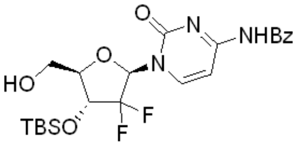

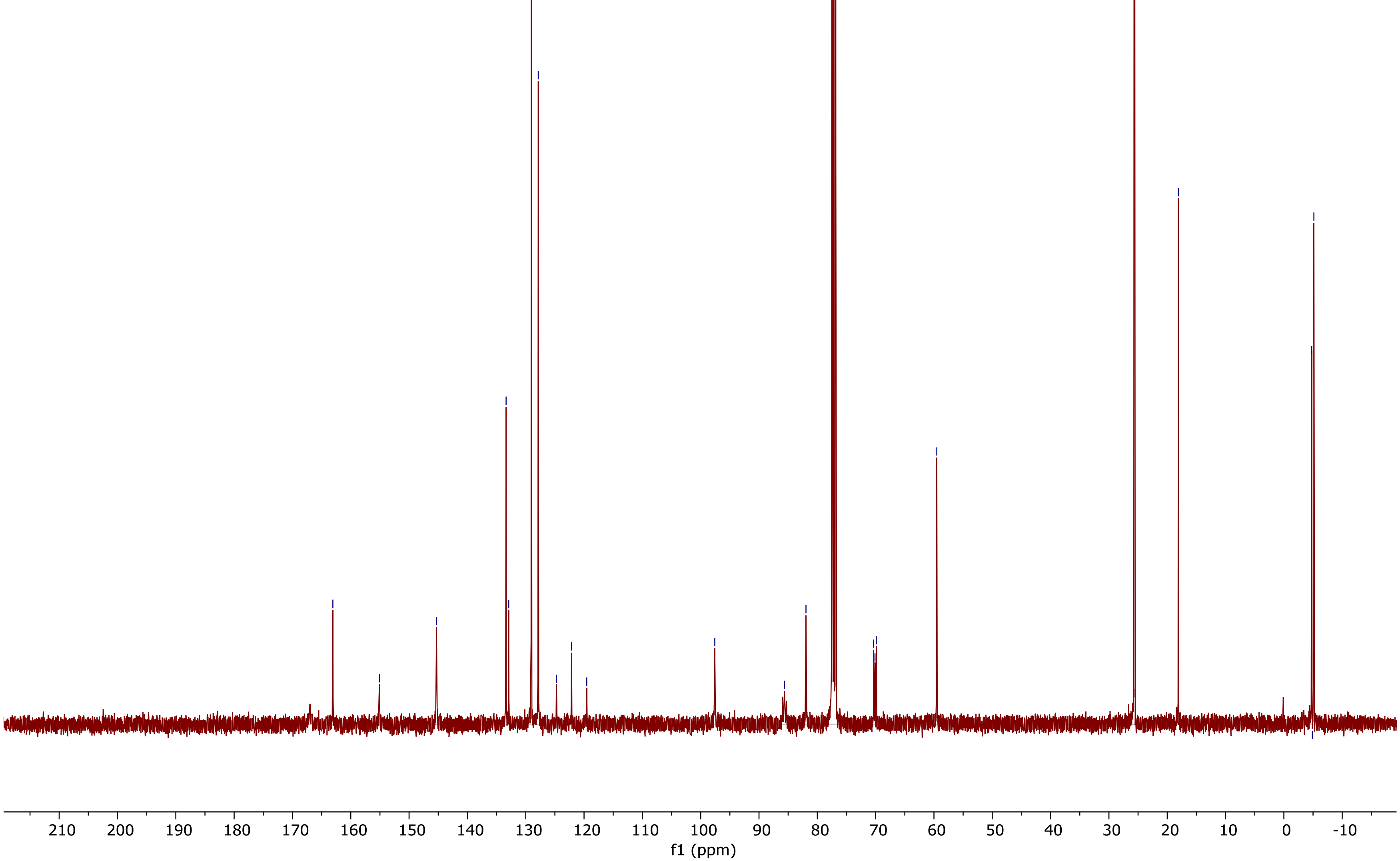

Figure S6 ^{19}F NMR (377 MHz, $CDCl_3$): 3'-*O*-*tert*Butyldimethylsilyl-*N*-4-benzoyl-2’,2’-difluorocytidine **1**

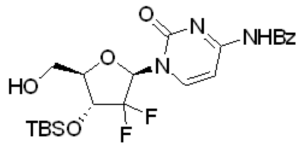

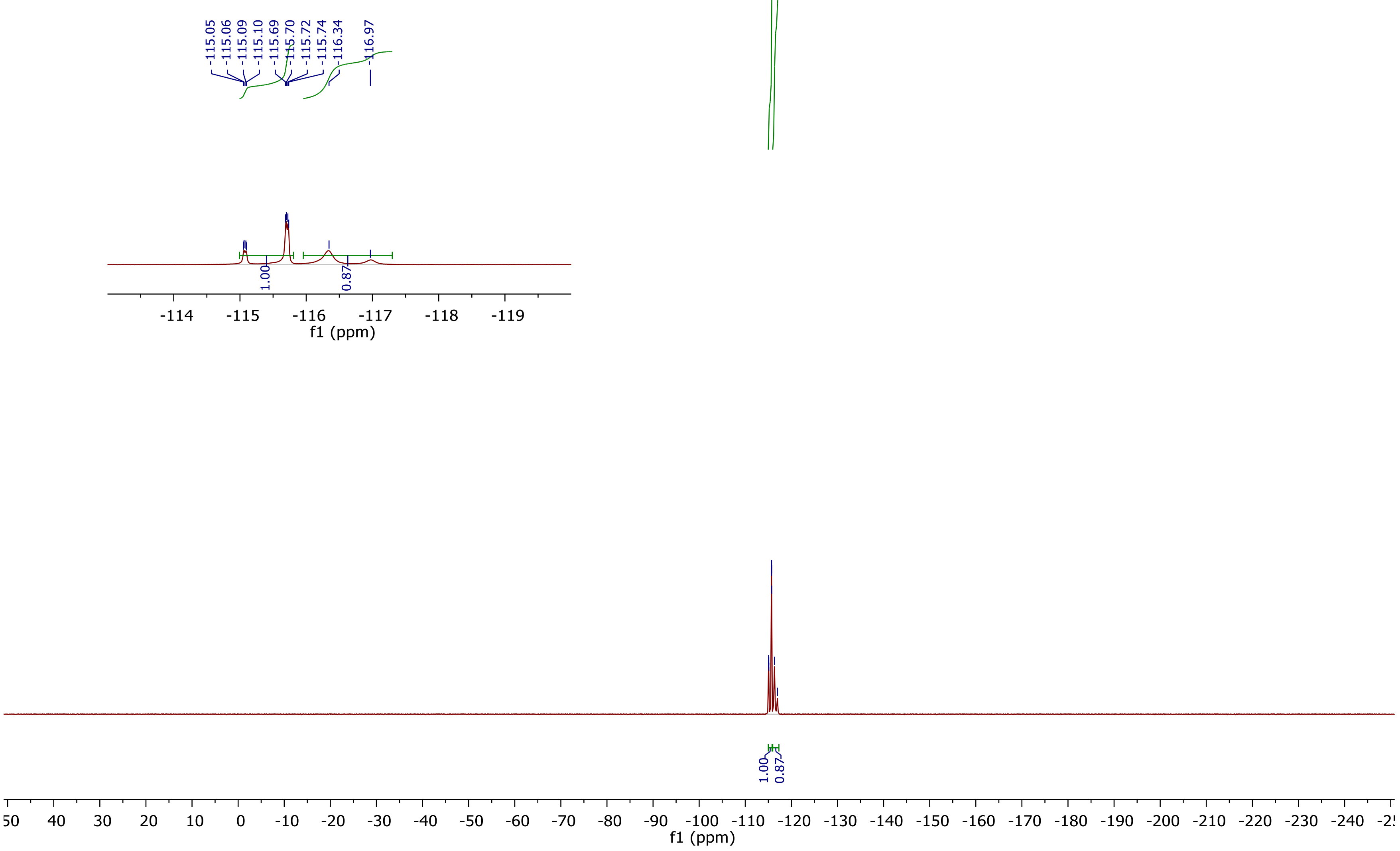

Figure S7 ^{1}H NMR (400 MHz, $CDCl_3$): 2',3'-5'-Tri-*O*-*tert*butyldimethylsilyl -*N*-4-benzoyl-arabinocytidine S3

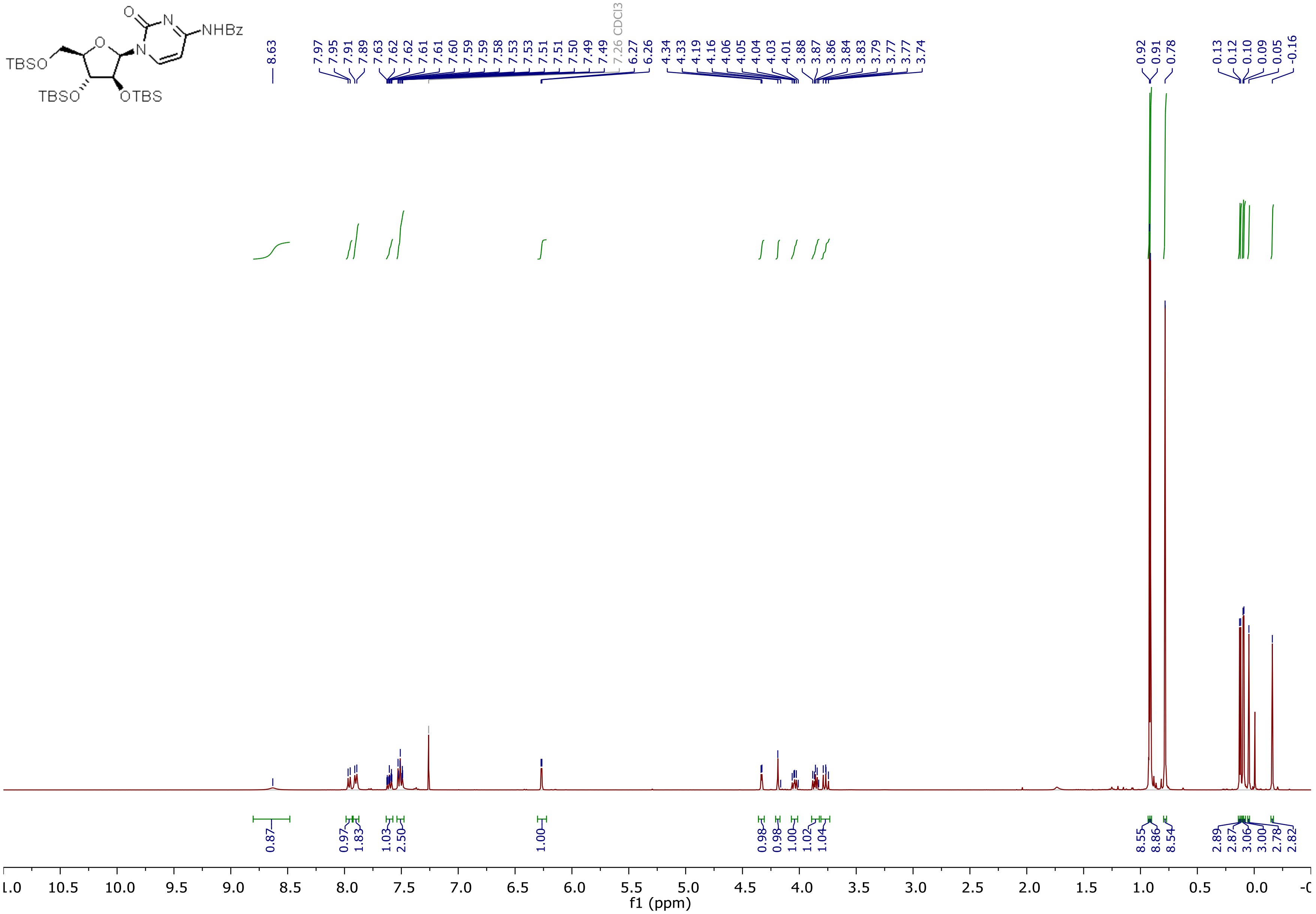

Figure S8 ^{13}C NMR (101 MHz, $CDCl_3$): 2',3'-5'-Tri-*O*-*tert*butyldimethylsilyl-*N*-4-benzoyl-arabinocytidine S3

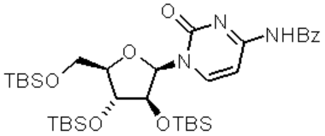

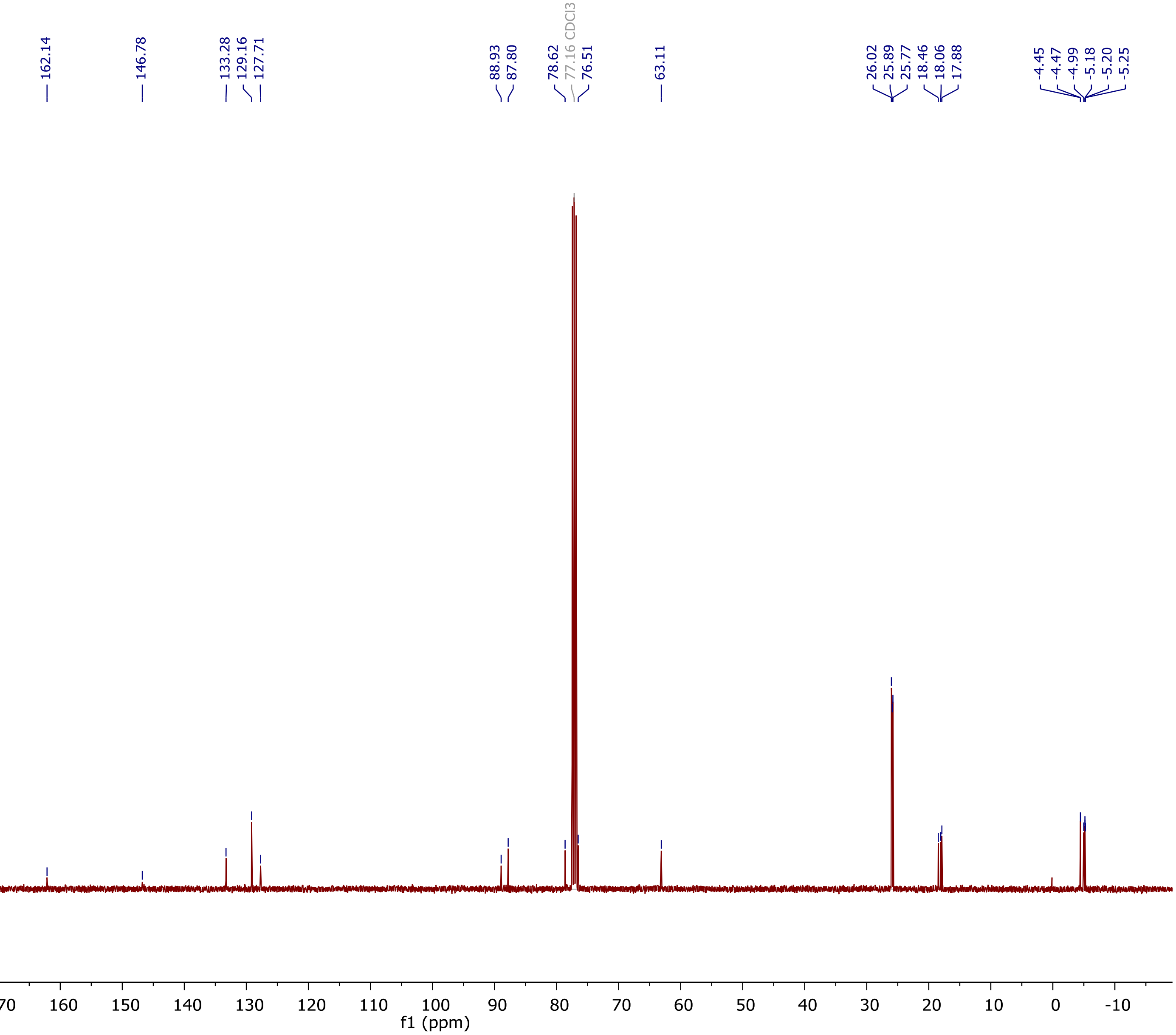

Figure S9 ^{1}H NMR (400 MHz, CDCl$_3$): 2',3'-Di-*O*-*tert*butyldimethylsilyl-*N*-4-benzoyl-arabinocytidine 2

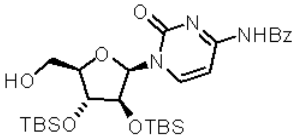

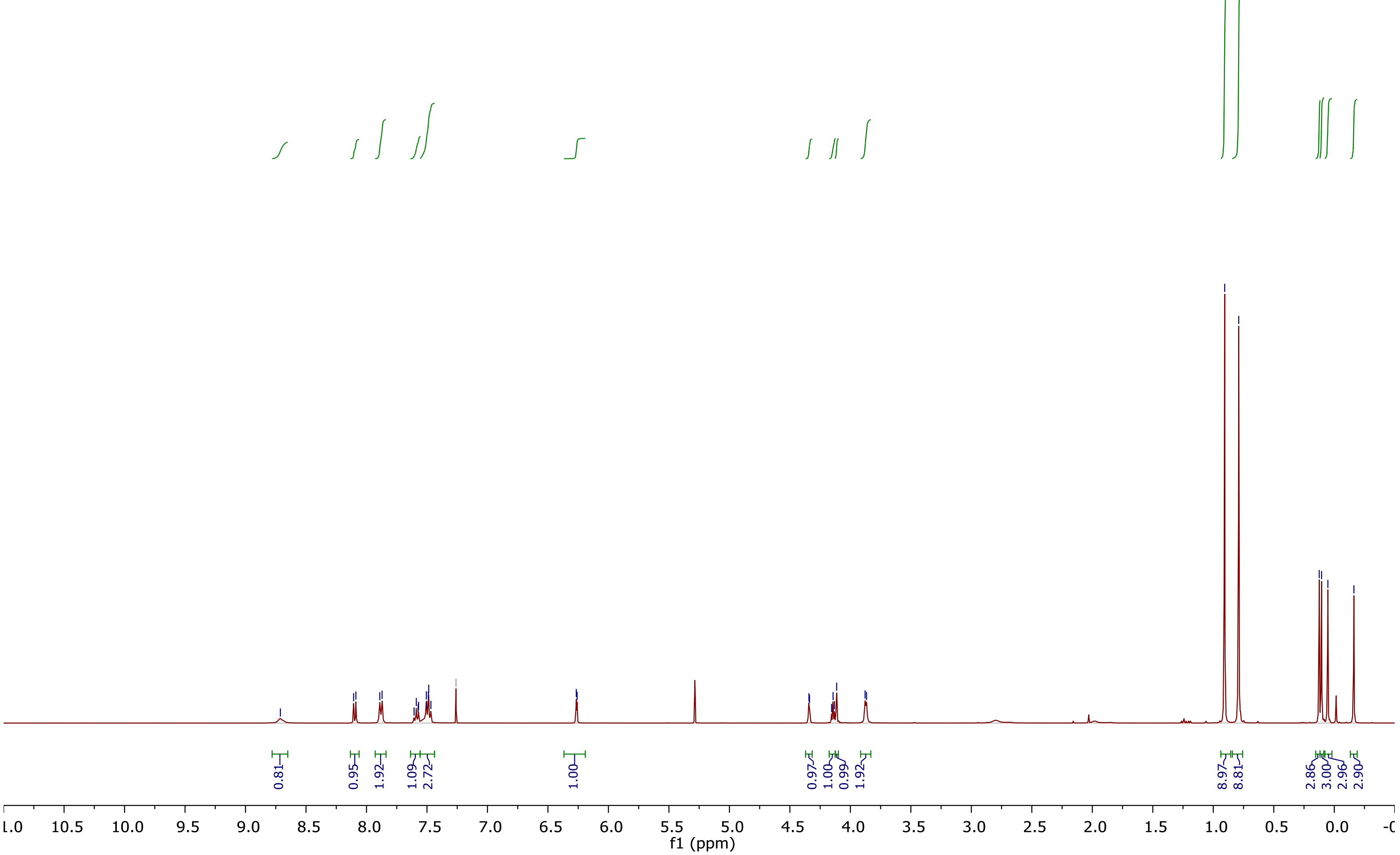

Figure S10 ^{13}C NMR (101 MHz, $CDCl_3$): 2',3'-Di-*O*-*tert*butyldimethylsilyl-*N*-4-benzoyl-arabinocytidine 2

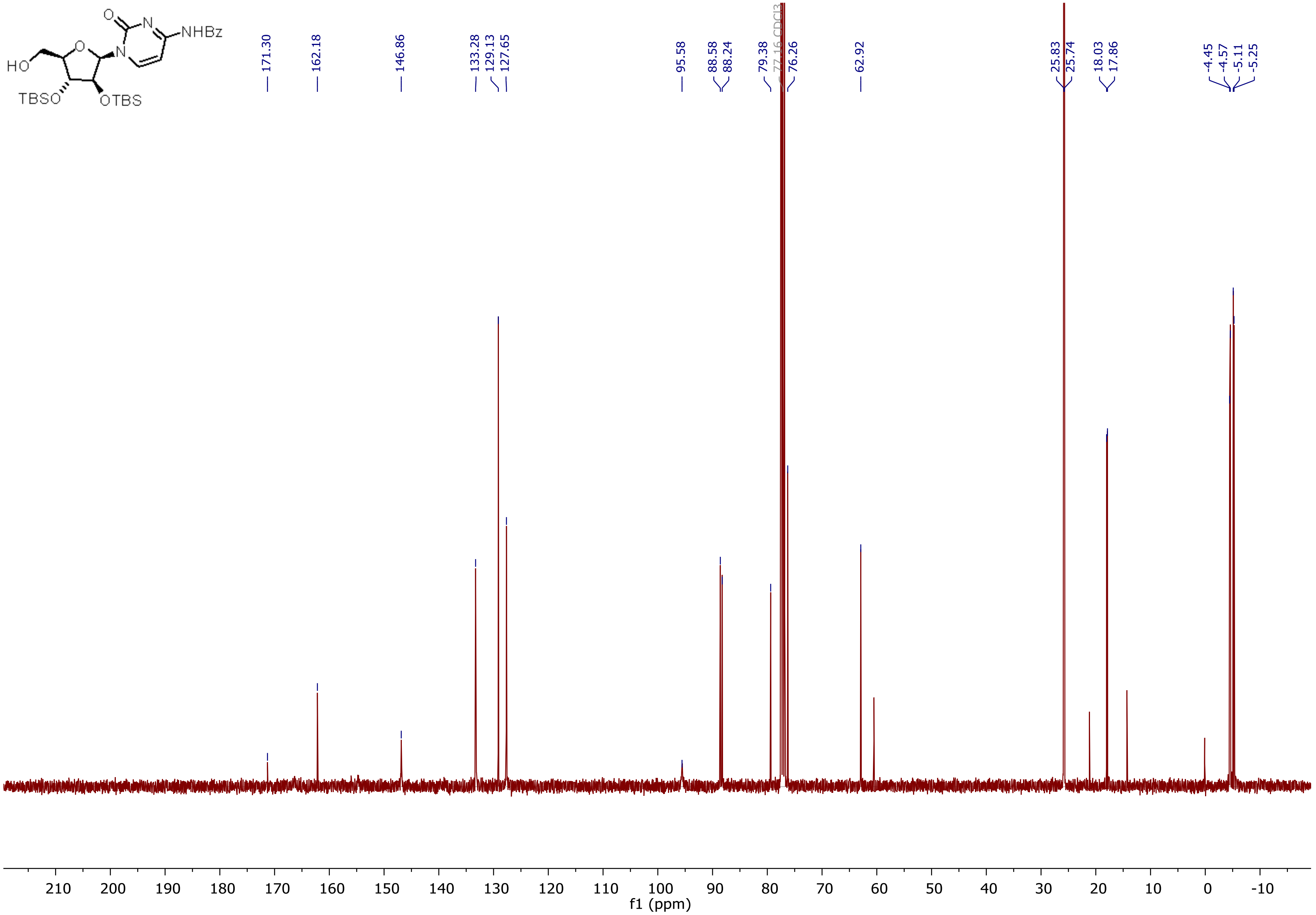

Figure S11 ^{1}H NMR (400 MHz, $CDCl_3$): 1,2,3,4-Tetra-*O*-acetyl-6-*O*-trityl-β-D-glucopyranoside S5

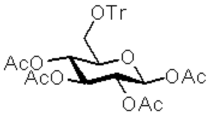

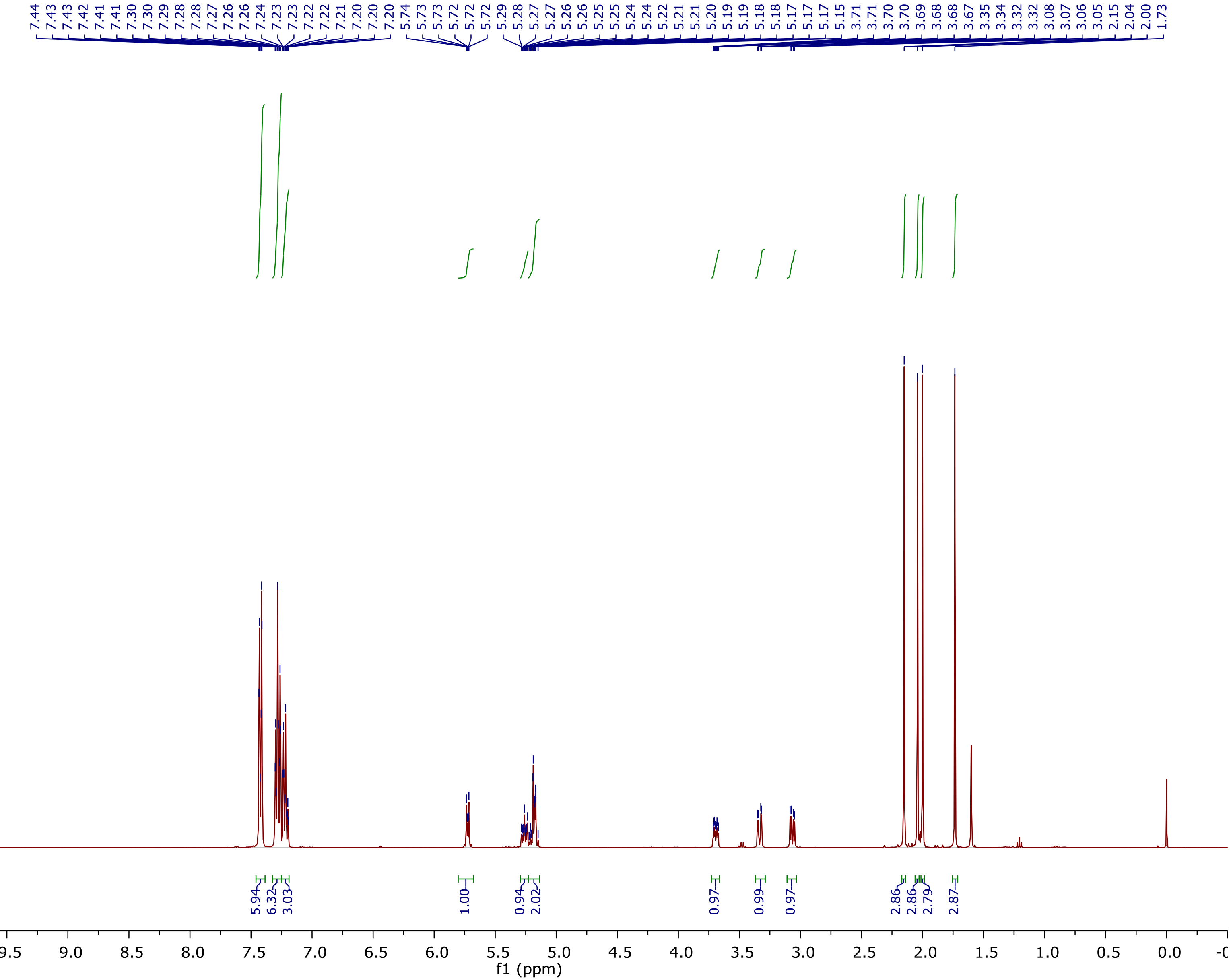

Figure S12 ^{13}C NMR (101 MHz, $CDCl_3$): 1,2,3,4-Tetra-*O*-acetyl-6-*O*-trityl-β-D-glucopyranoside S5

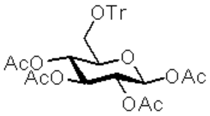

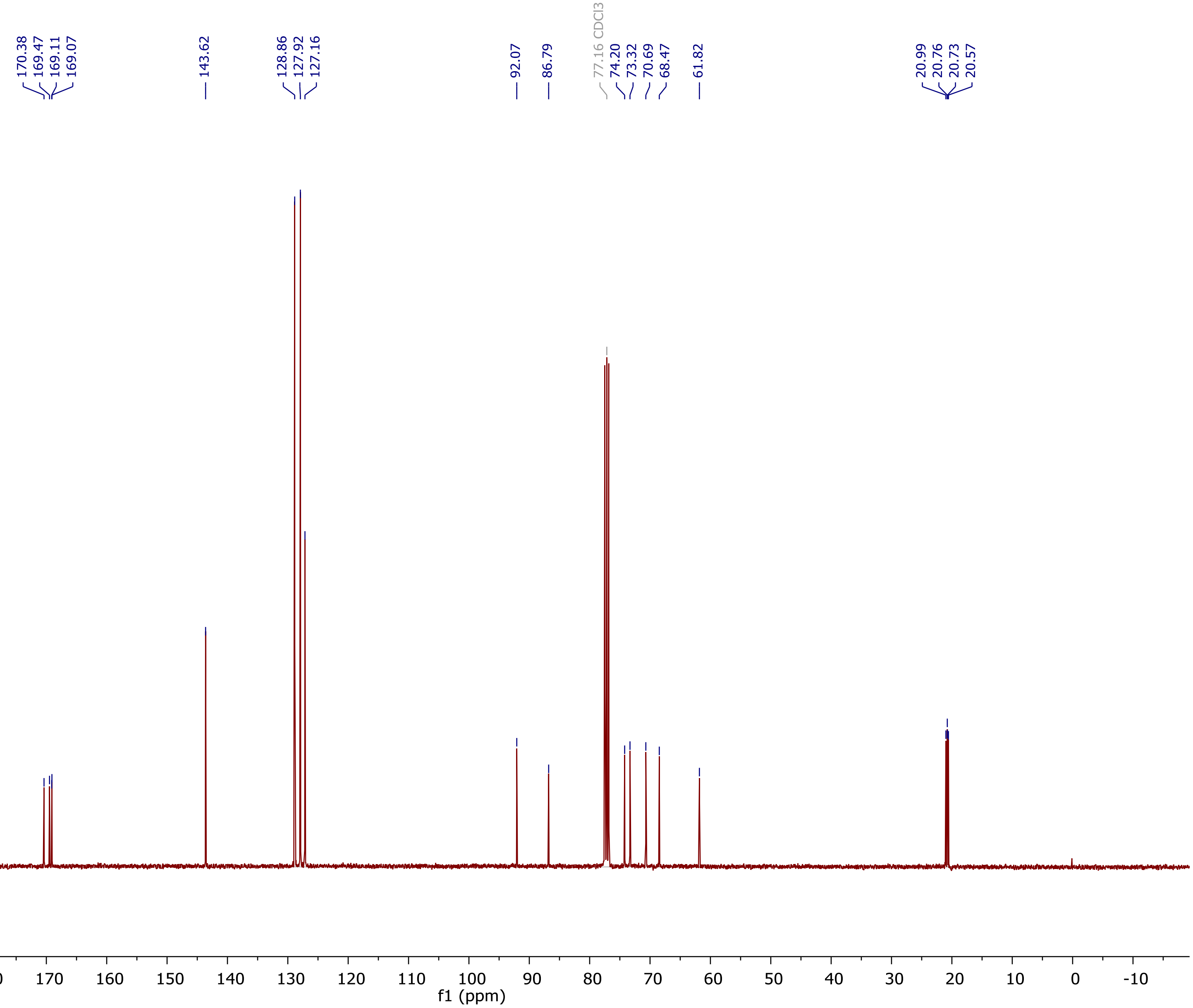

Figure S13 ^{1}H NMR (400 MHz, $CDCl_3$): 1,2,3,4-Tetra-*O*-acetyl-β-D-glucopyranoside 5

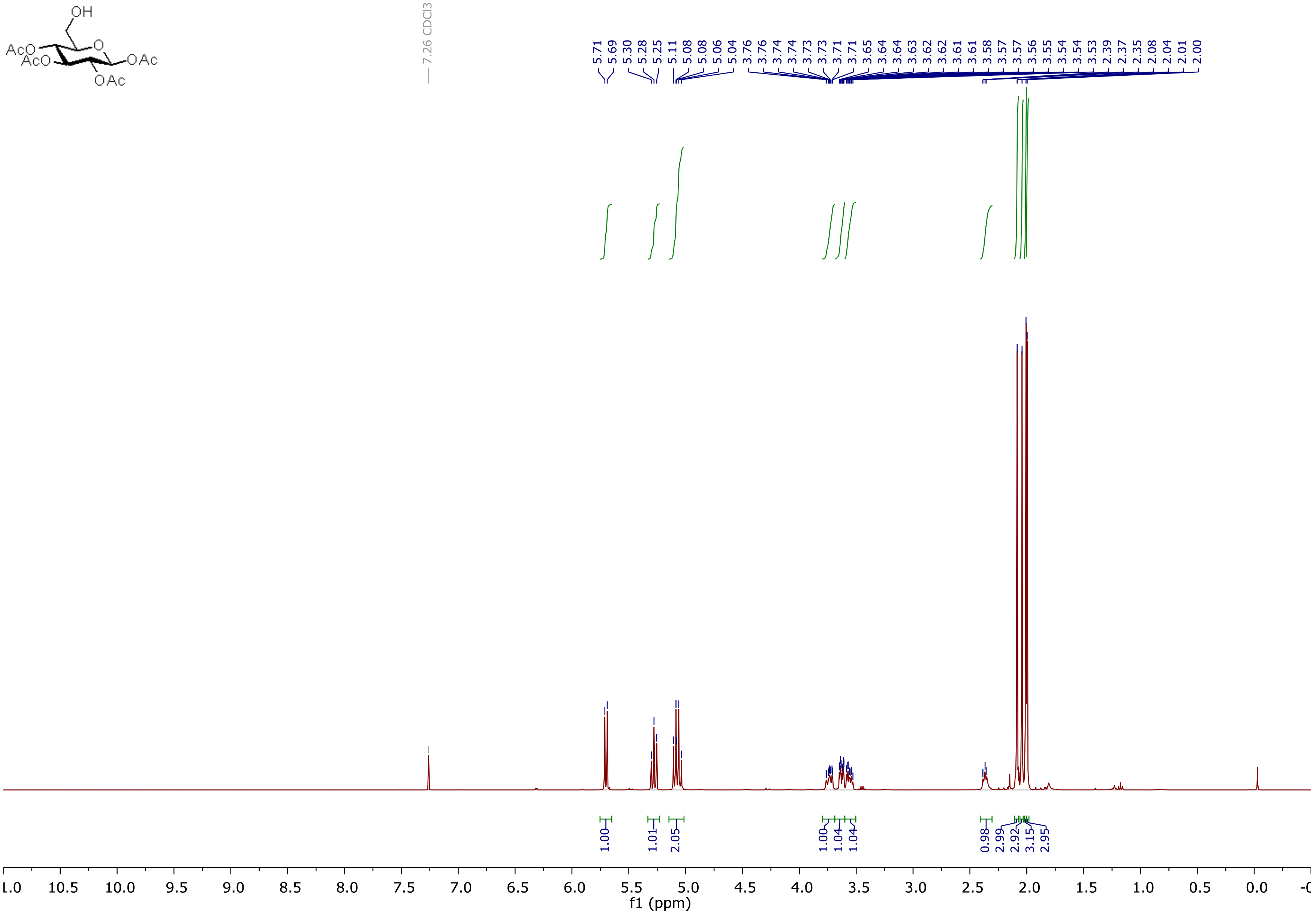

Figure S14 ^{13}C NMR (101 MHz, CDCl$_3$): 1,2,3,4-Tetra-*O*-acetyl-β-D-glucopyranoside 5

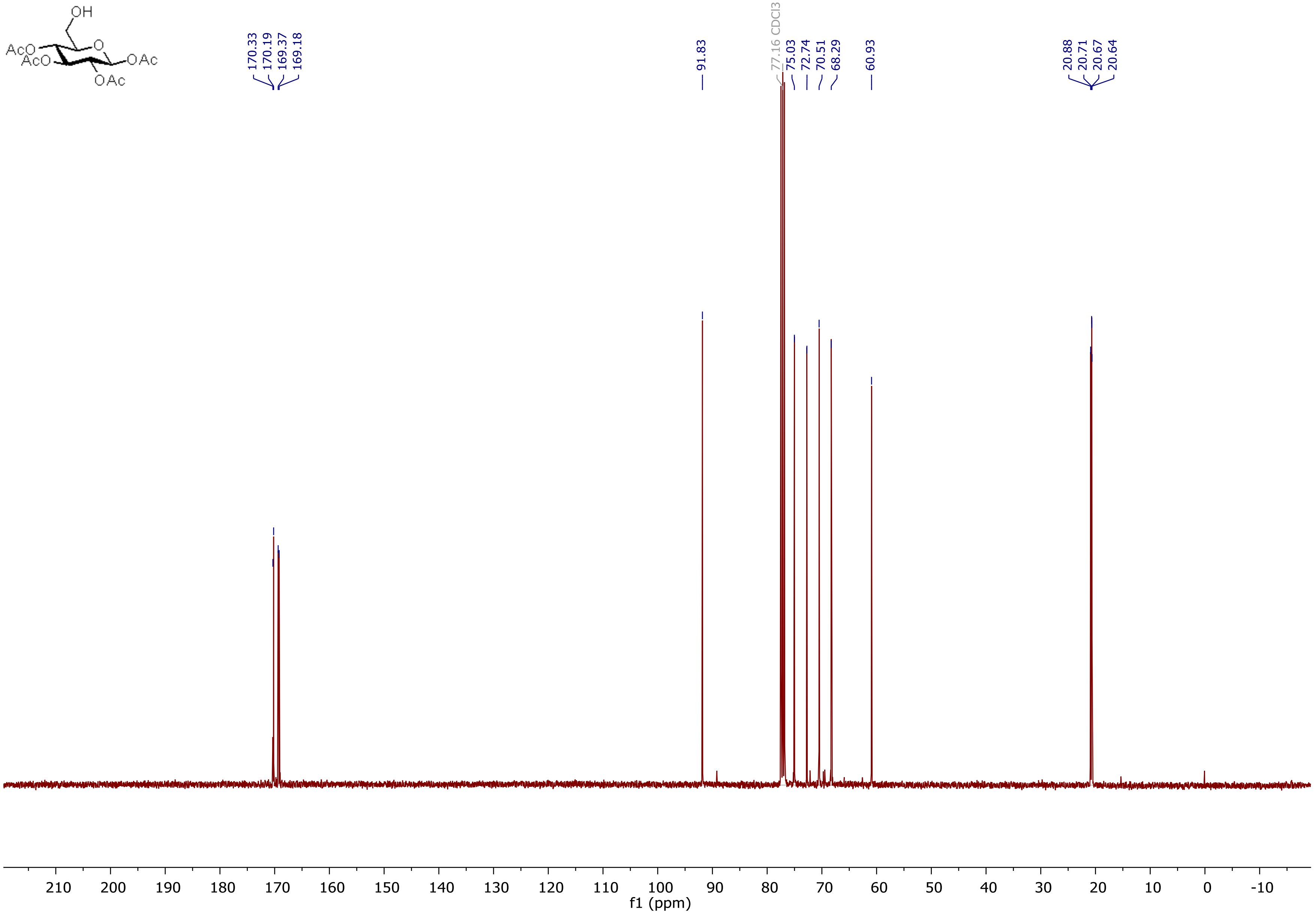

Figure S15 ^{1}H NMR (400 MHz, $CDCl_3$): 1,2,3,4-Tetra-*O*-acetyl-6-*O*-trityl-α/β-D-galactopyranoside S7

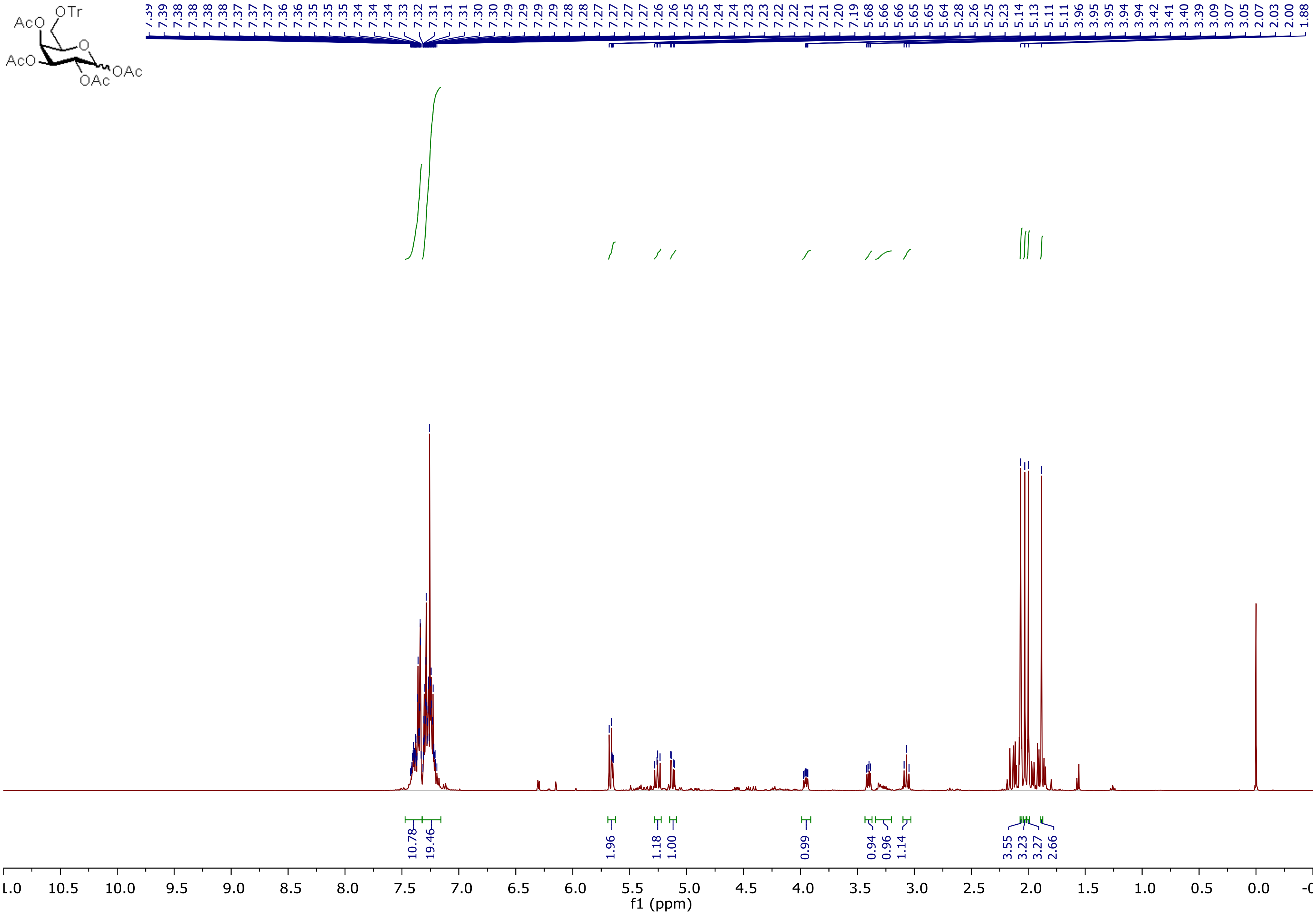

Figure S16 ^{13}C NMR (101 MHz, $CDCl_3$): 1,2,3,4-Tetra-*O*-acetyl-6-*O*-trityl-α/β-D-galactopyranoside S7

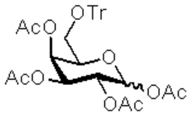

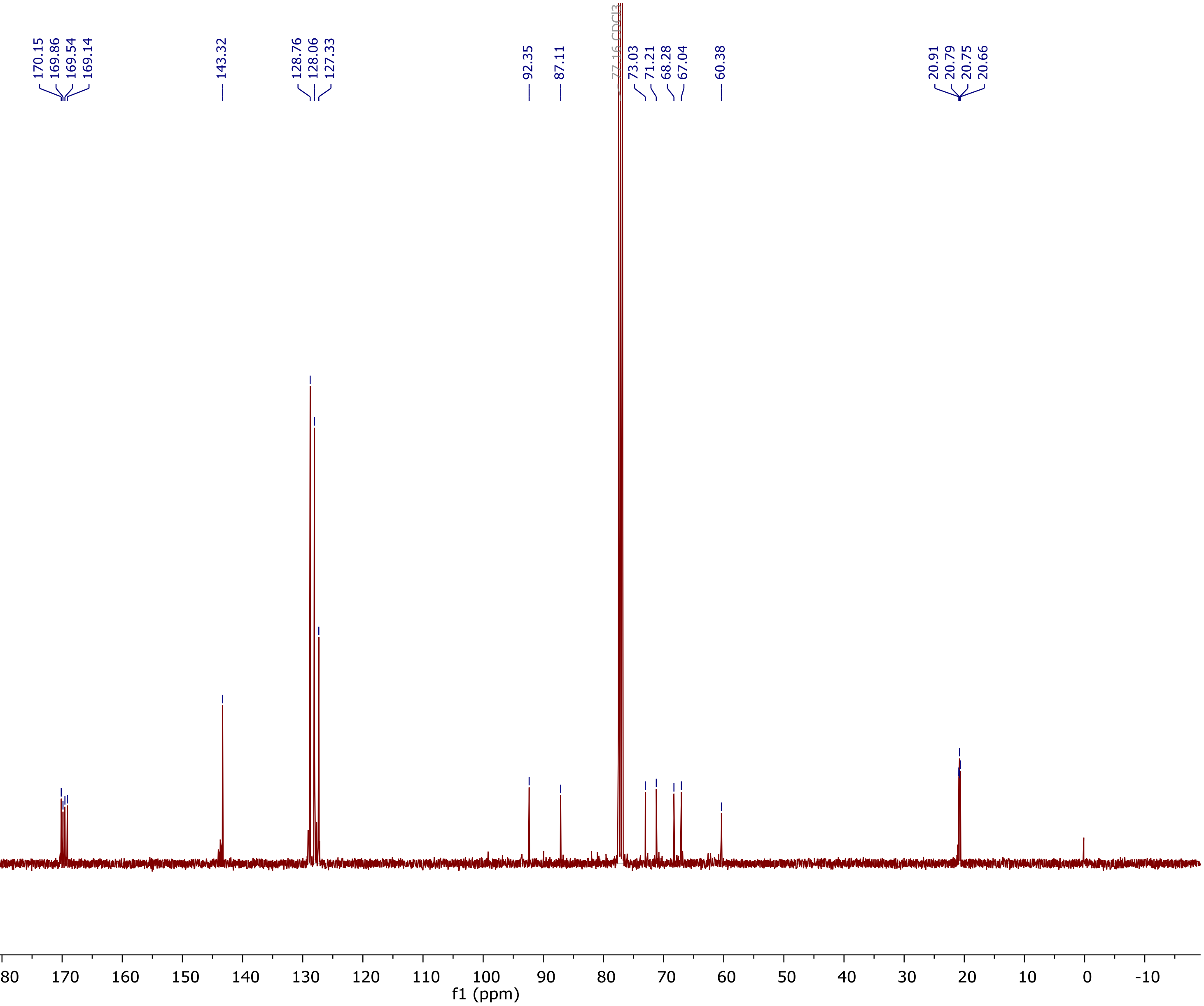

Figure S17 ^{1}H NMR (400 MHz, $CDCl_3$): 1,2,3,4-Tetra-*O*-acetyl-α/β-D-galactopyranoside 16

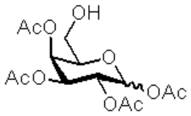

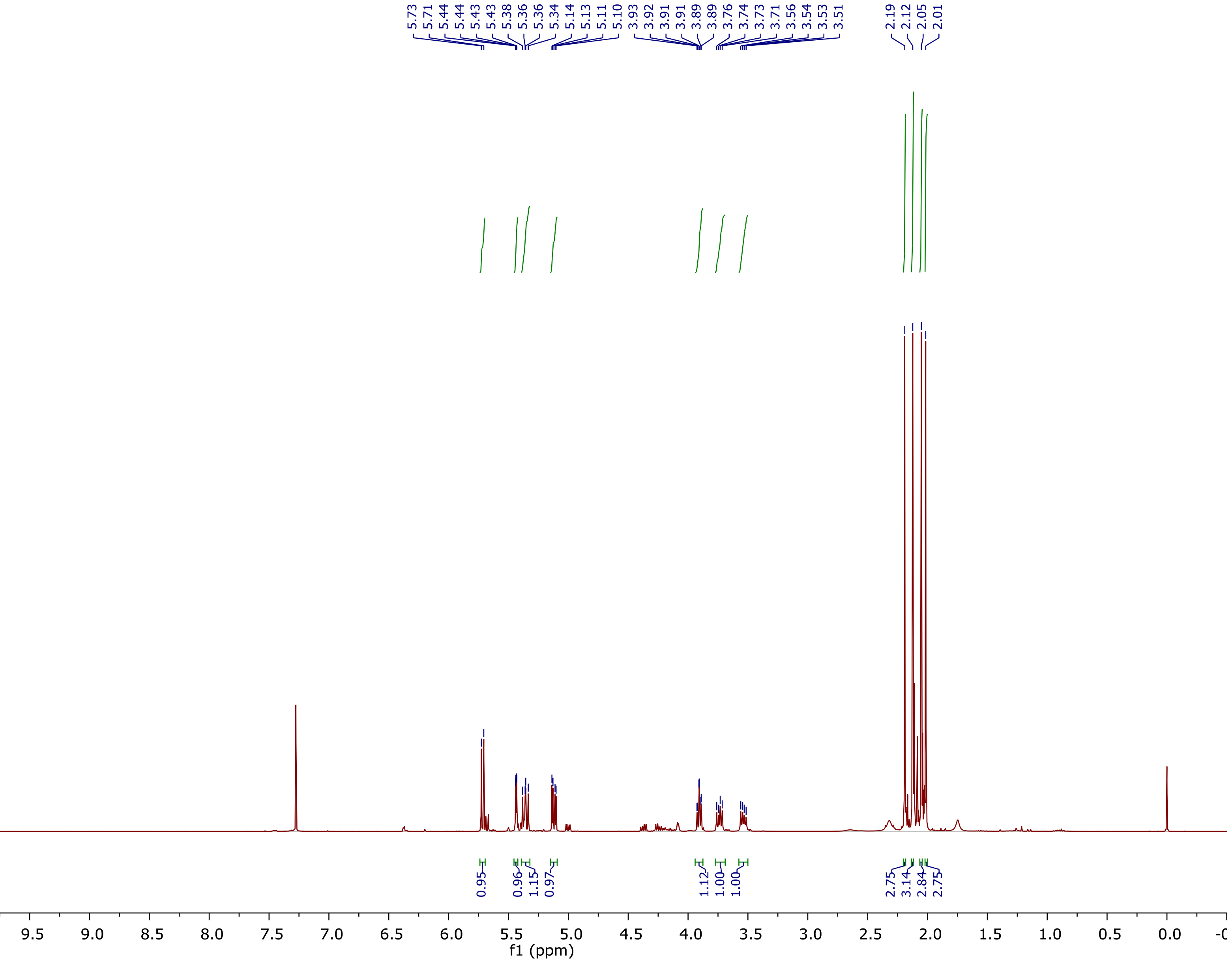

Figure S18 ^{13}C NMR (101 MHz, $CDCl_3$): 1,2,3,4-Tetra-*O*-acetyl-α/β-D-galactopyranoside 16

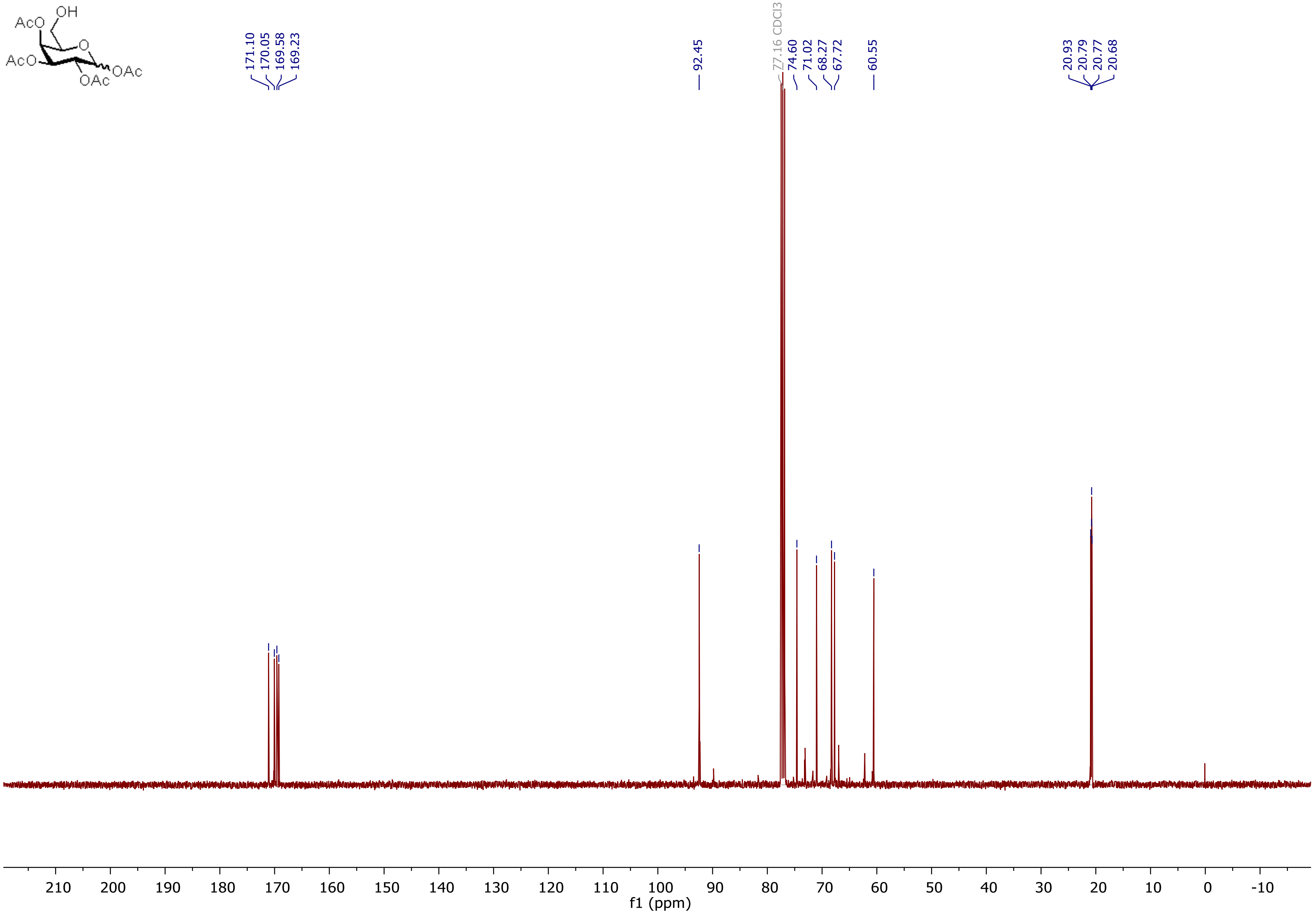

Figure S19 ^{1}H NMR (400 MHz, $CDCl_3$): 2,3,4,6-Tetra-*O*-acetyl-α/β-D-glucopyranoside 10

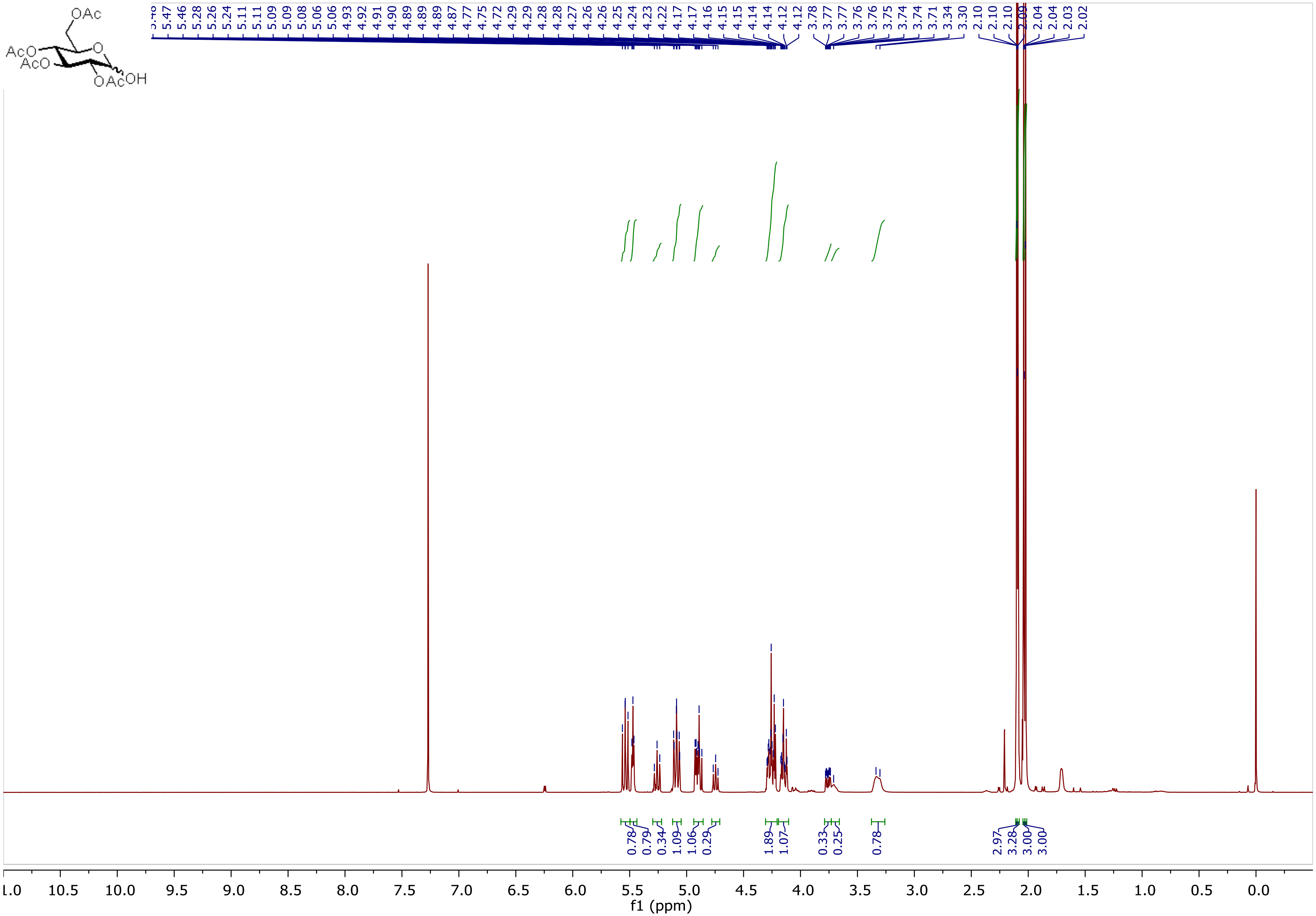

Figure S20 ^{13}C NMR (101 MHz, $CDCl_3$): 2,3,4,6-Tetra-*O*-acetyl-α/β-D-glucopyranoside 10

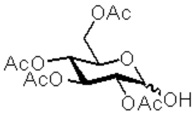

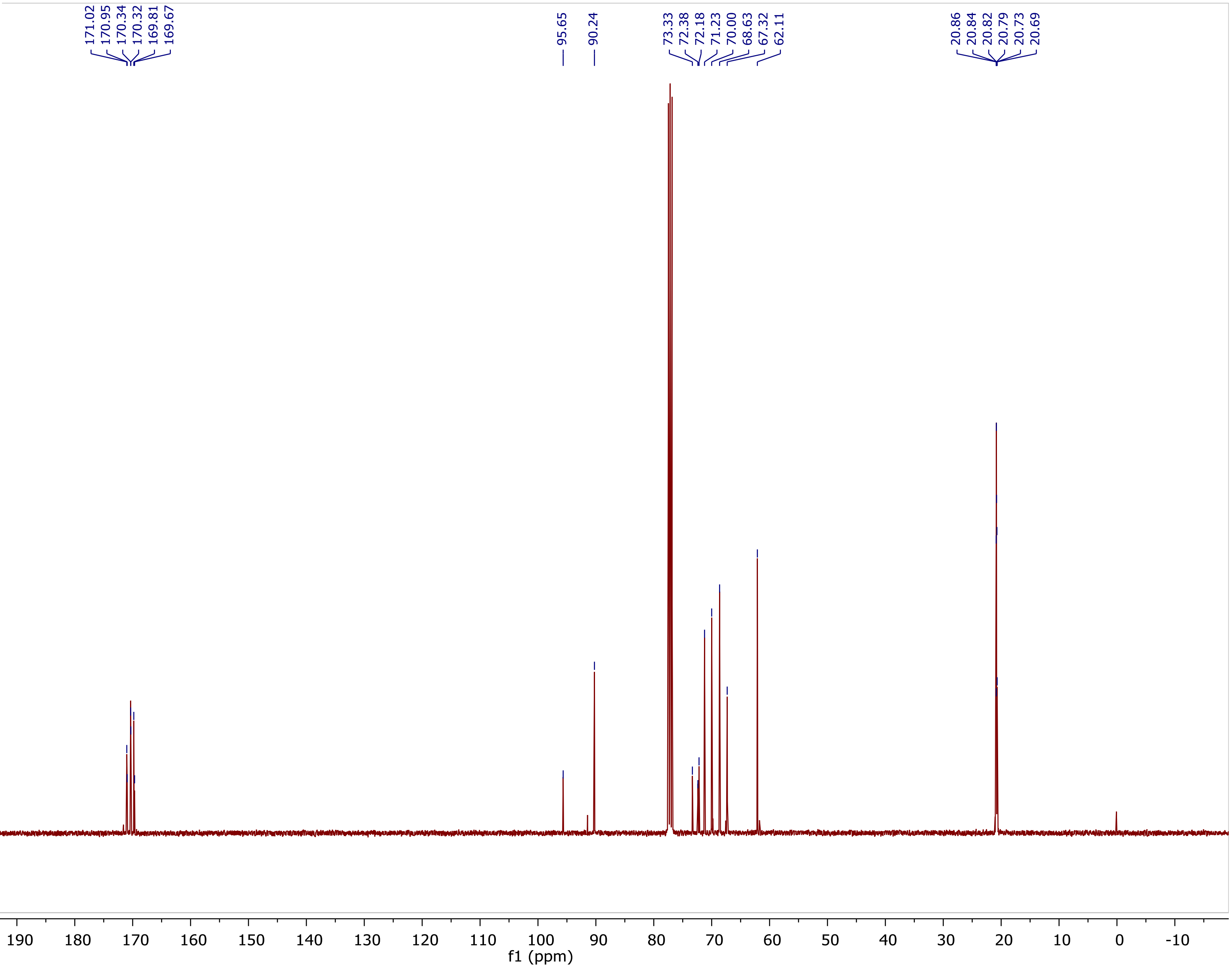

Figure S21 ^{1}H NMR (400 MHz, $CDCl_3$): 2,3,4,6-Tetra-*O*-acetyl-α/β-D-galactopyranoside 21

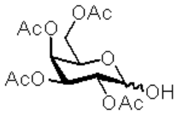

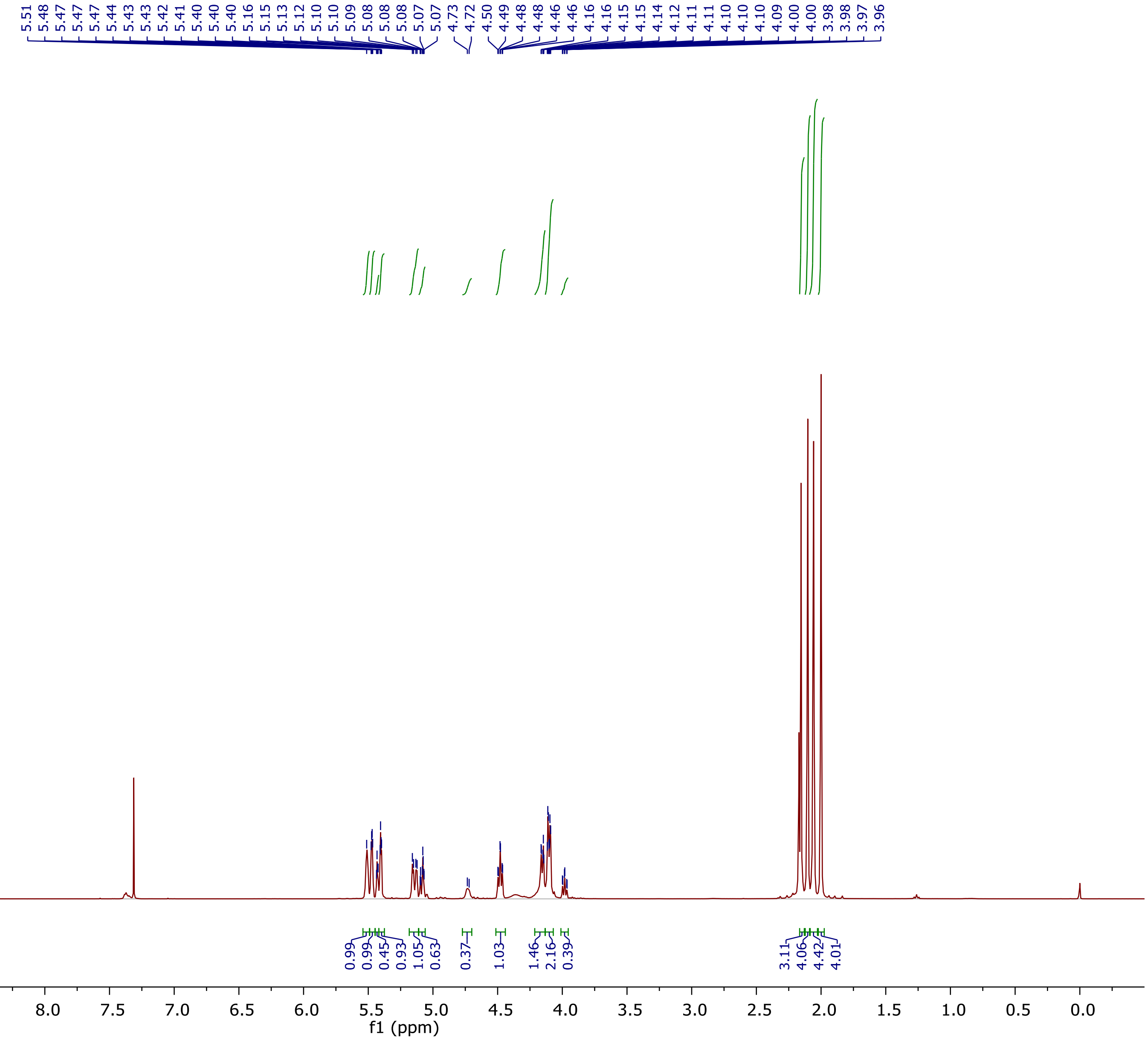

Figure S22 ^{13}C NMR (101 MHz, $CDCl_3$): 2,3,4,6-Tetra-*O*-acetyl-α/β-D-galactopyranoside 21

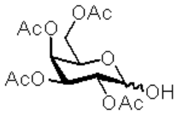

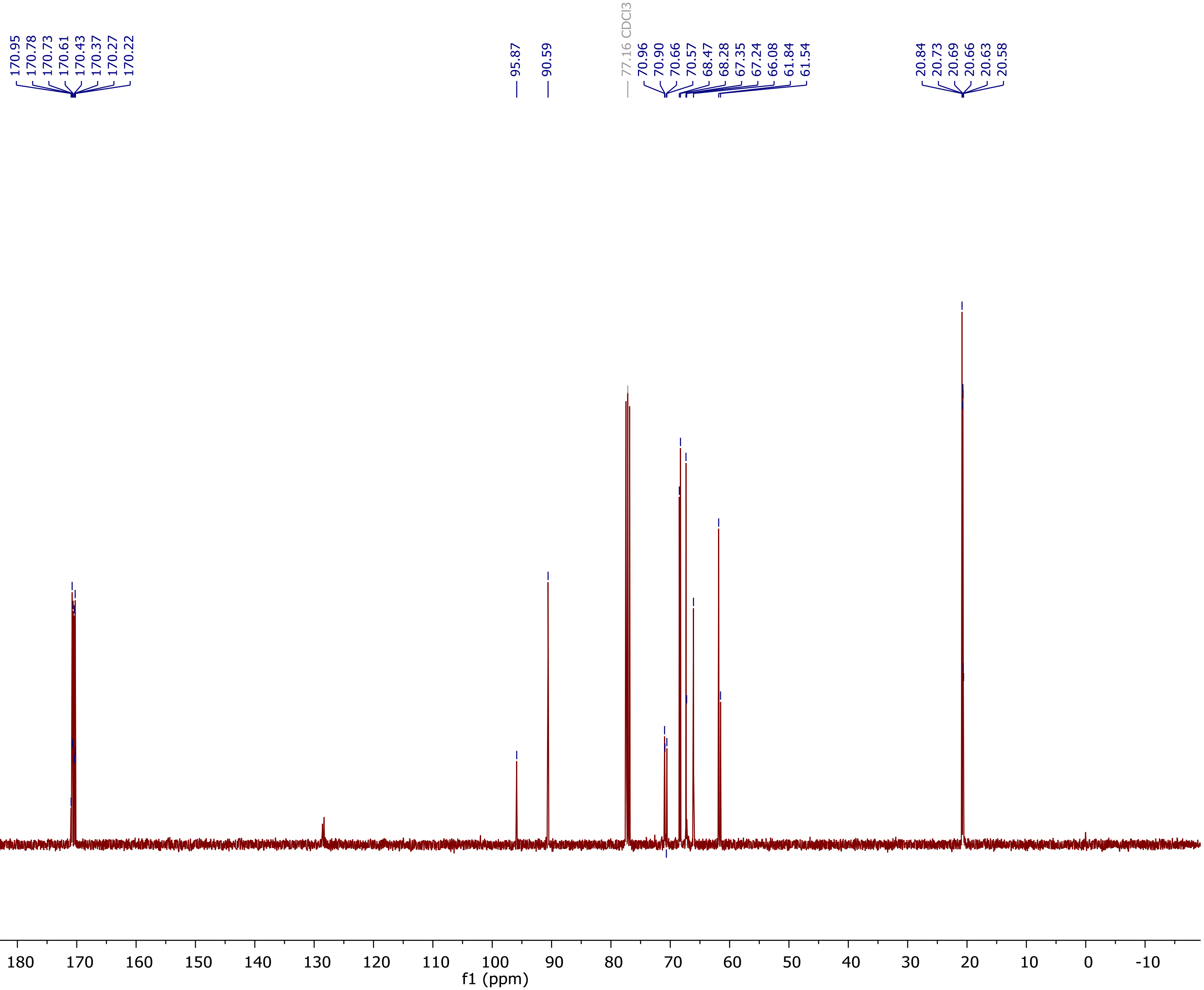

Figure S23 ^{1}H NMR (400 MHz, $CDCl_3$): 3'-*O*-*tert*Butyldimethylsilyl-*N*-4-benzoyl-2’,2’-difluorocytidine-5'-*O*-hydrogenphosphonate triethylammonium salt 3

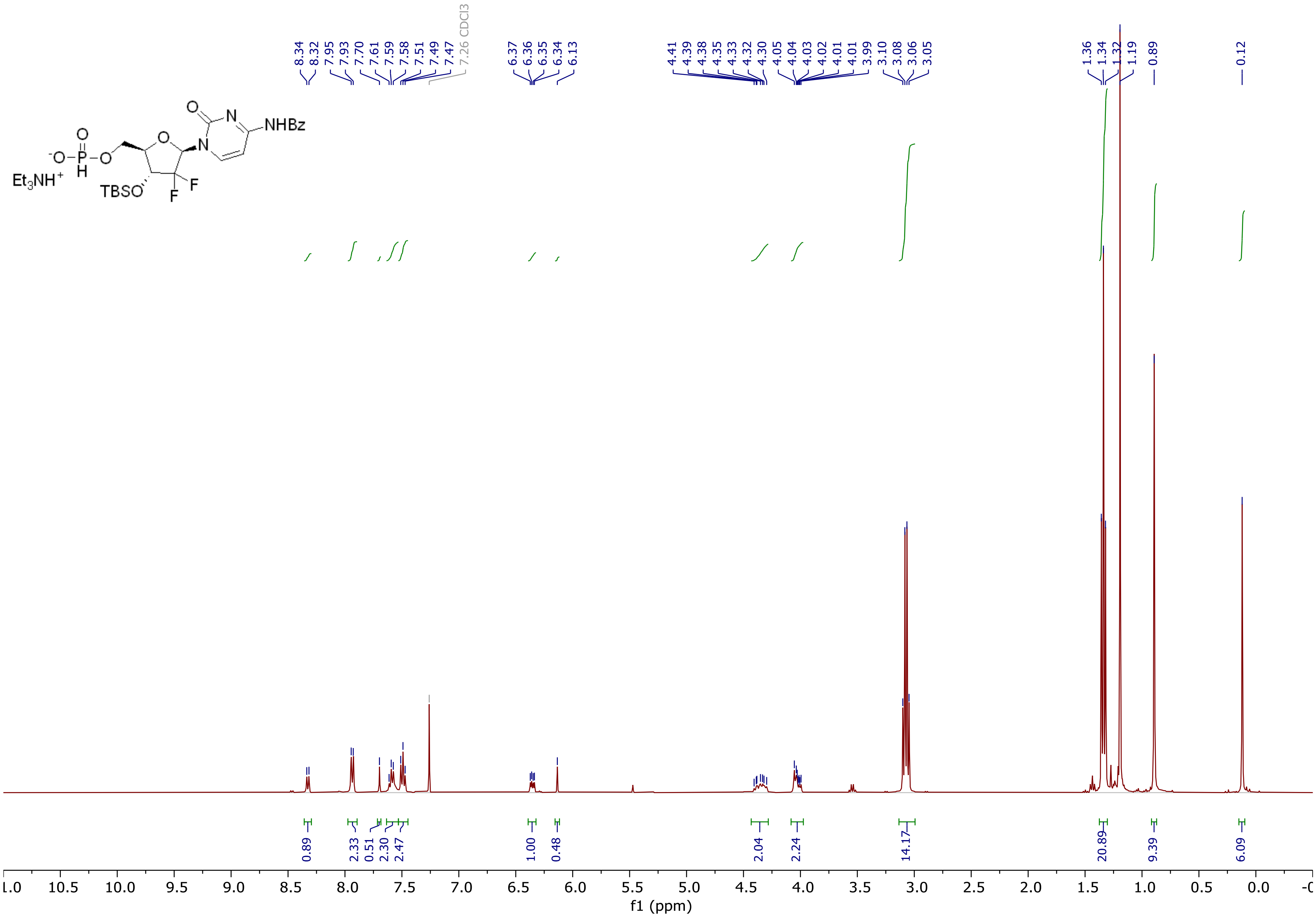

Figure S24 ^{13}C NMR (101 MHz, $CDCl_3$): 3'-*O*-*tert*Butyldimethylsilyl-*N*-4-benzoyl-2',2'-difluorocytidine-5'-*O*-hydrogenphosphonate triethylammonium salt 3

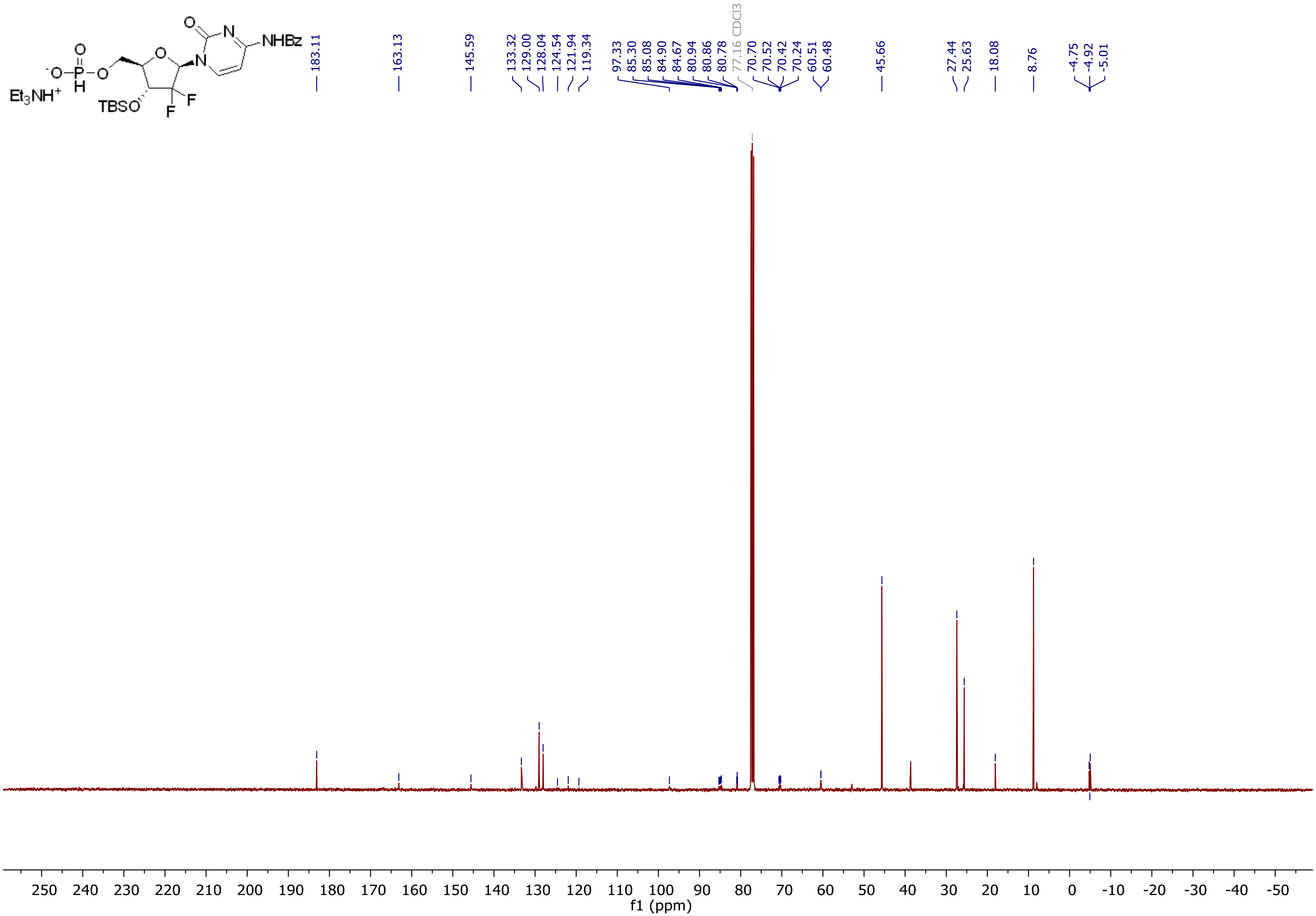

Figure S25 ^{19}F {^{1}H} NMR (377 MHz, $CDCl_3$): 3'-*O*-*tert*Butyldimethylsilyl-*N*-4-benzoyl-2',2'-difluorocytidine-5'-*O*-hydrogenphosphonate triethylammonium salt 3

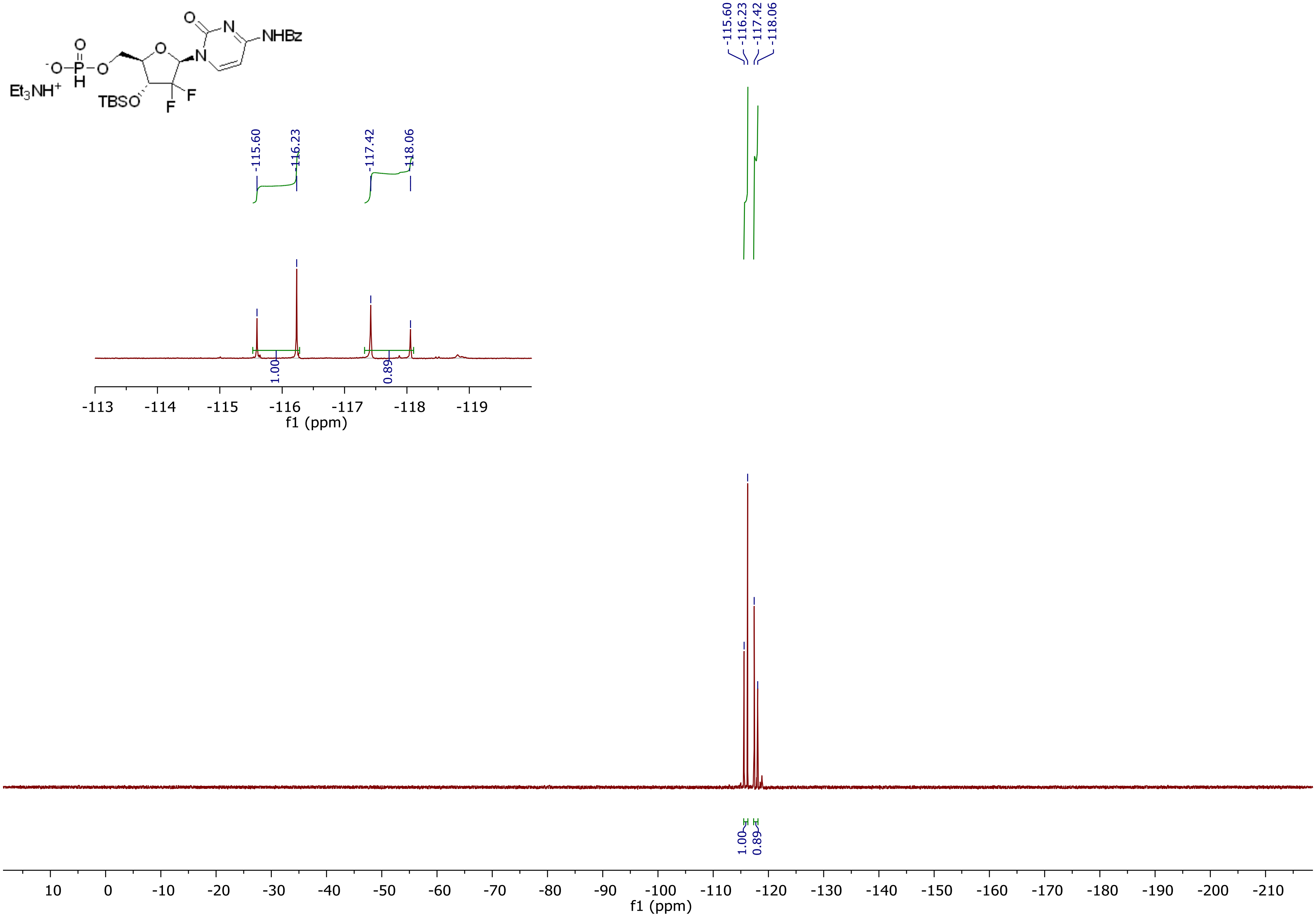

Figure S26 ^{31}P {^{1}H} NMR (162 MHz, $CDCl_3$): 3'-*O*-*tert*Butyldimethylsilyl-*N*-4-benzoyl-2',2'-difluorocytidine-5'-*O*-hydrogenphosphonate triethylammonium salt 3

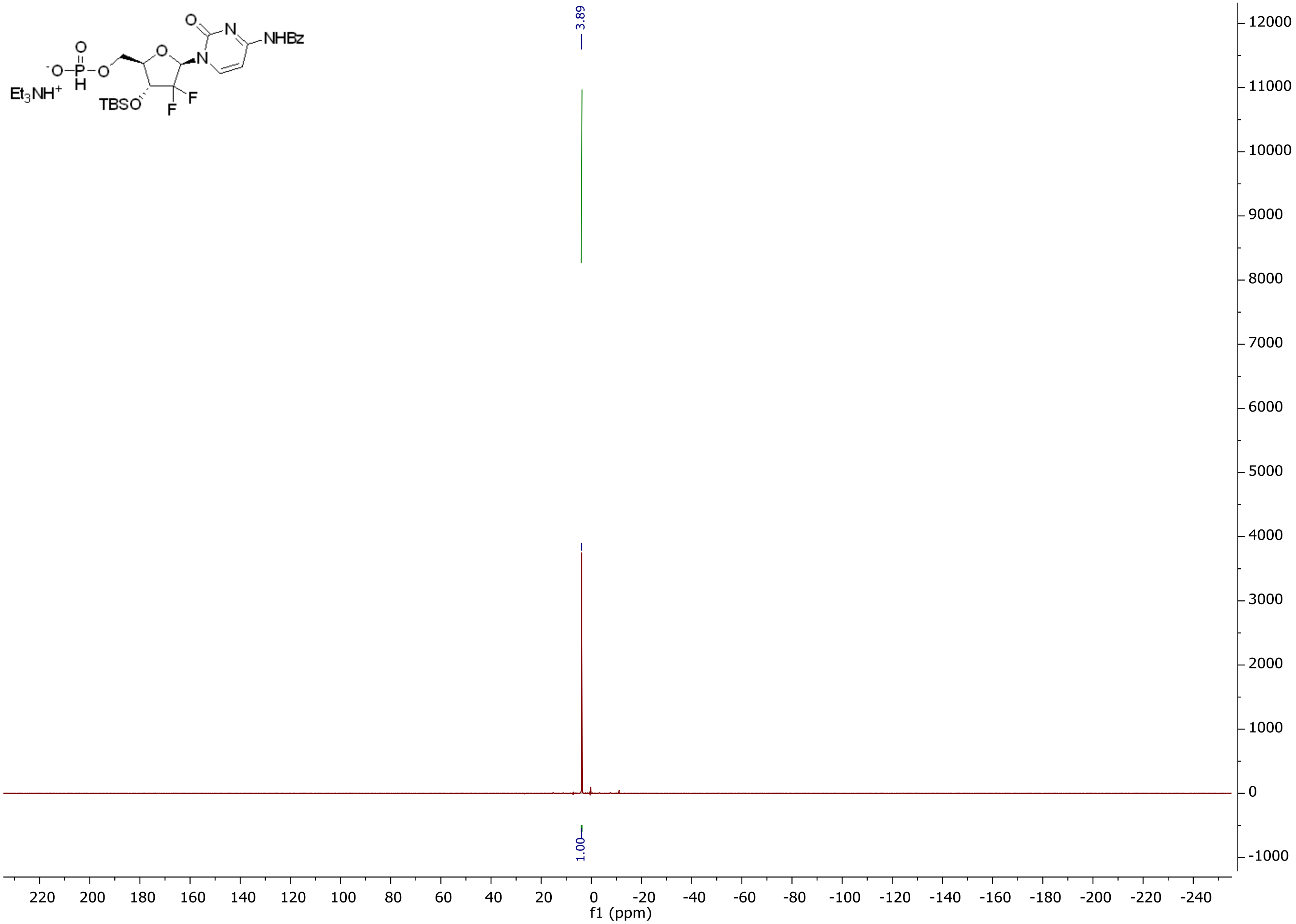

Figure S27 ^{1}H NMR (400 MHz, $CDCl_3$): 2',3'-*O*-*tert*Butyldimethylsilyl-*N*-4-benzoyl-arabinocytidine-5'-*O*-hydrogenphosphonate triethylammonium salt 4

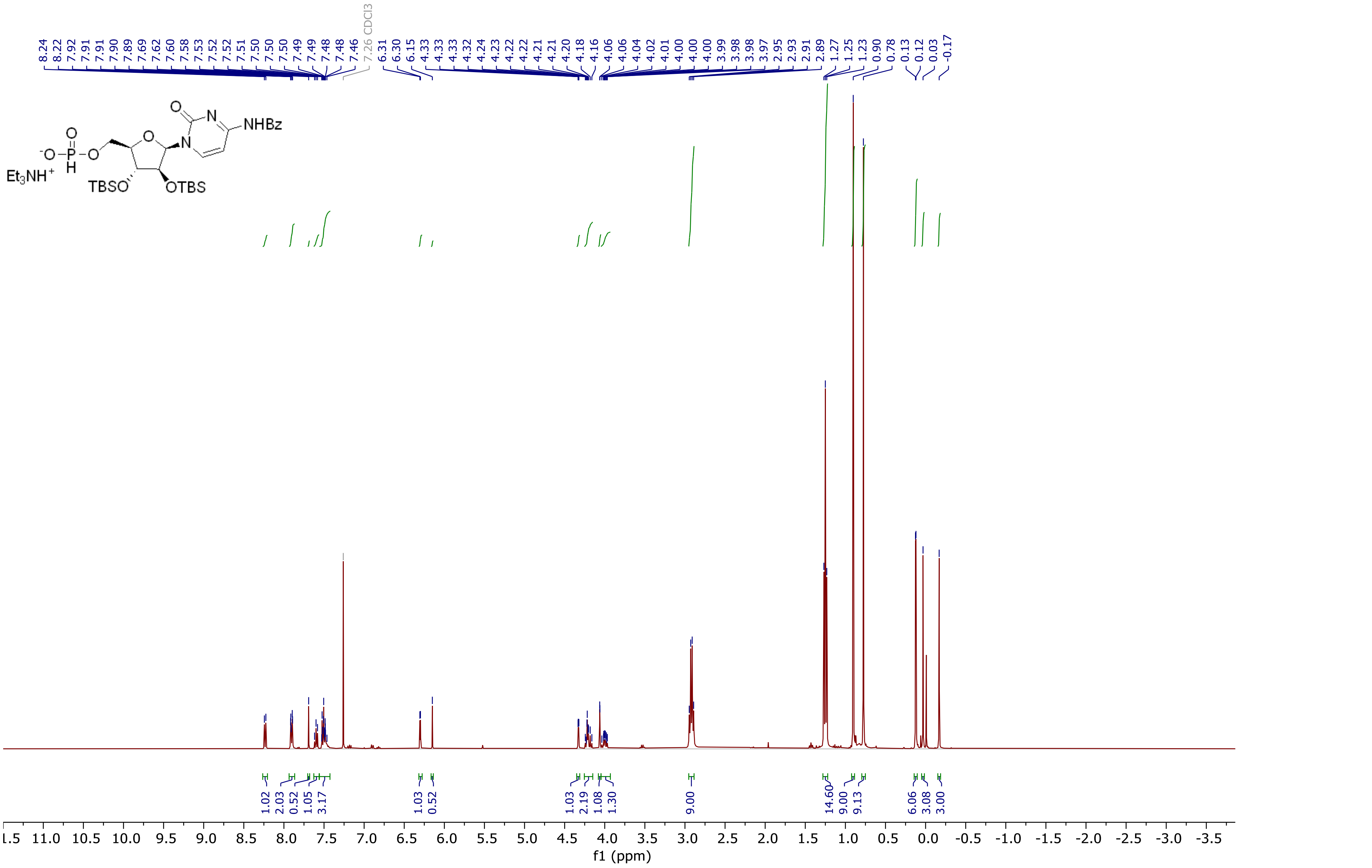

Figure S28 ^{13}C NMR (101 MHz, $CDCl_3$): 2',3'-*O*-*tert*Butyldimethylsilyl-*N*-4-benzoyl-arabinocytidine-5'-*O*-hydrogenphosphonate triethylammonium salt 4

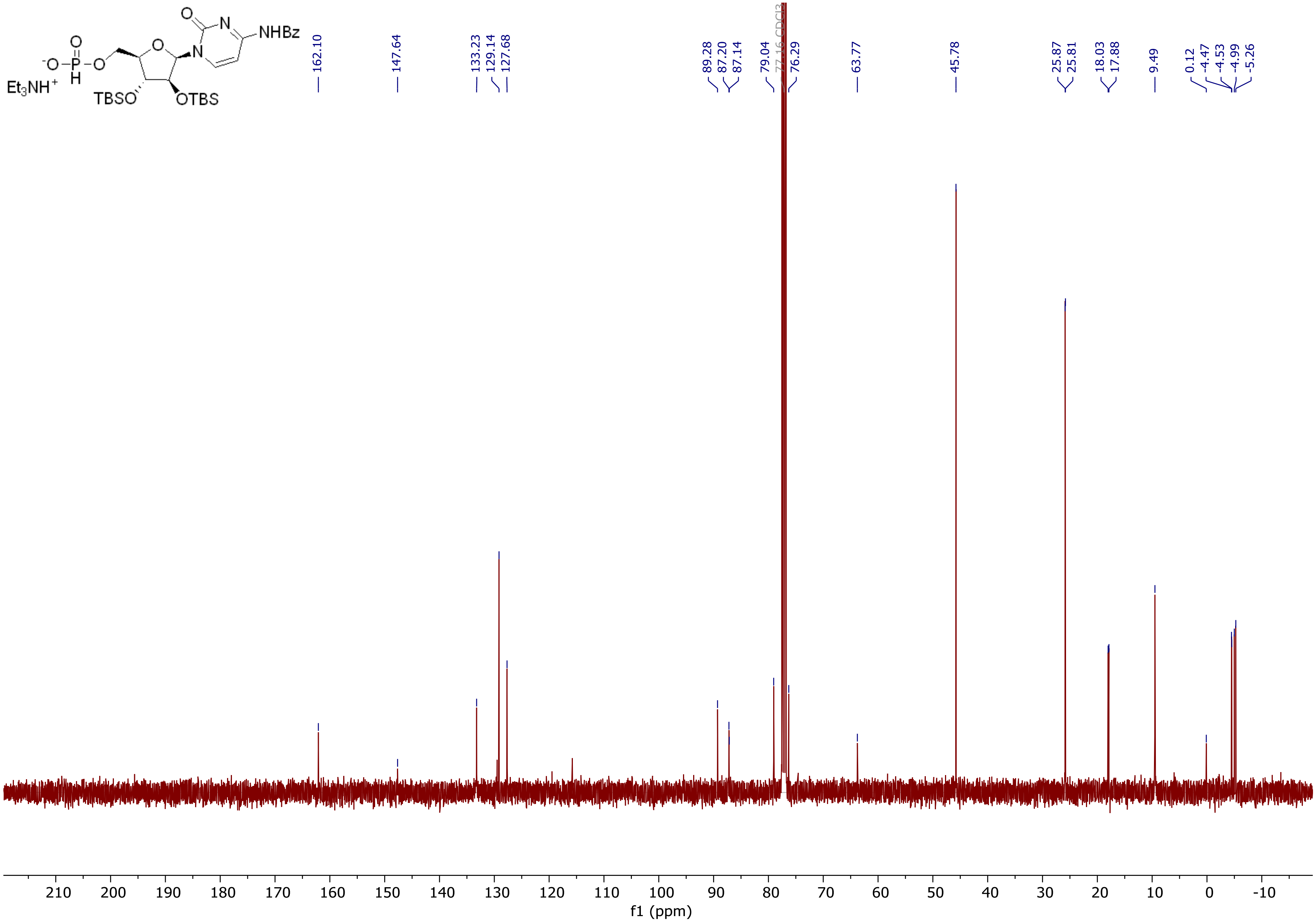

Figure S29 ^{31}P NMR (162 MHz, CDCl$_3$): 2',3'-*O*-*tert*Butyldimethylsilyl-*N*-4-benzoyl-arabinocytidine-5'-*O*-hydrogenphosphonate triethylammonium salt 4

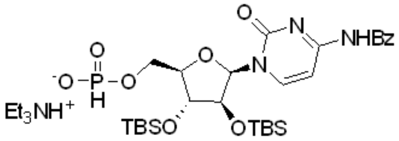

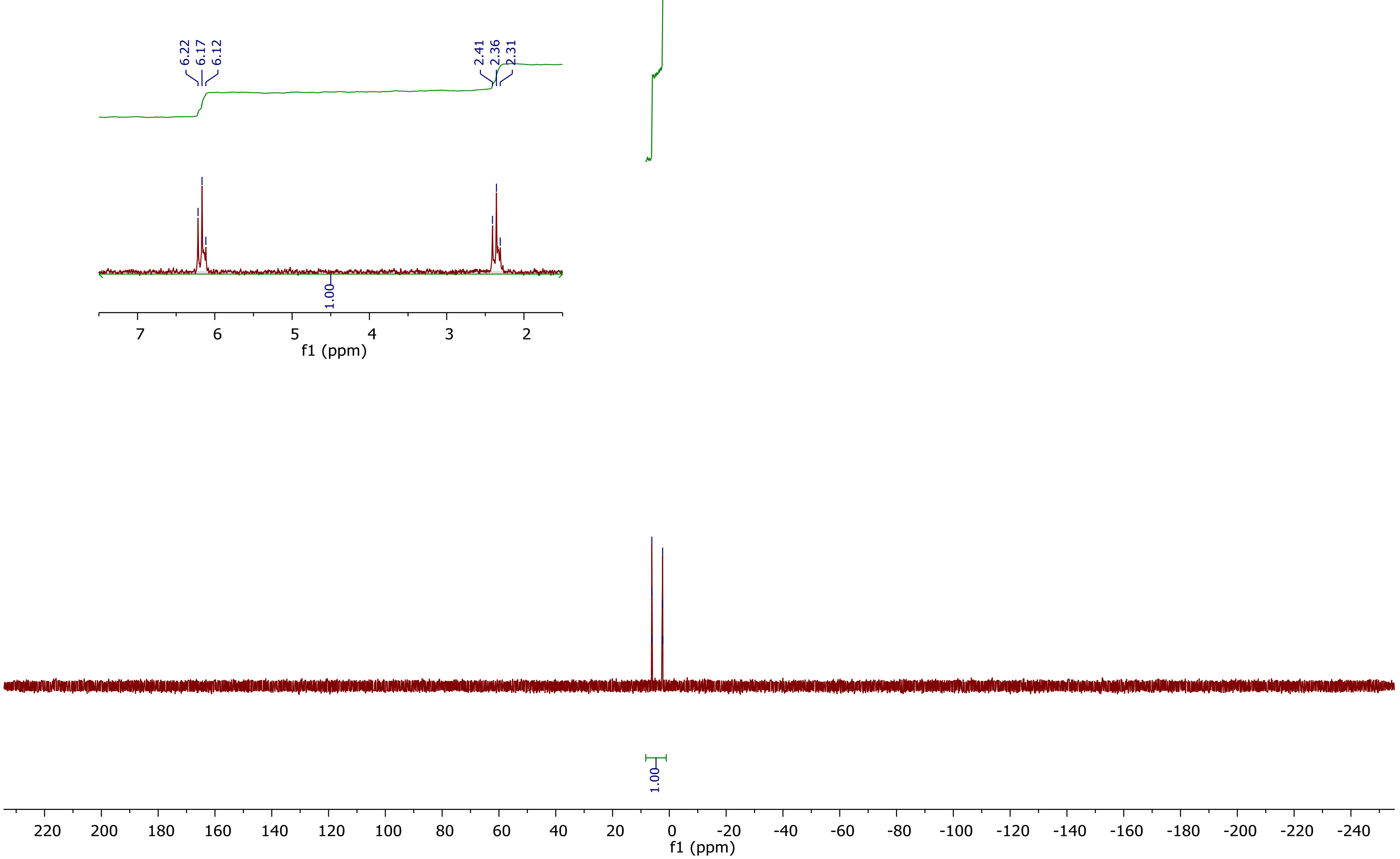

Figure S30 ^{1}H NMR (400 MHz, $CDCl_3$): 2',3'-*O*-*tert*Butyldimethylsilyl-*N*-4-benzoyl-arabinocytidine-5'-*O*-[6''-*O*-(1'',2'',3'',4''-tetra-*O*-actetyl-β-D-glucopyranose)]-hydrogenphosphonate S10

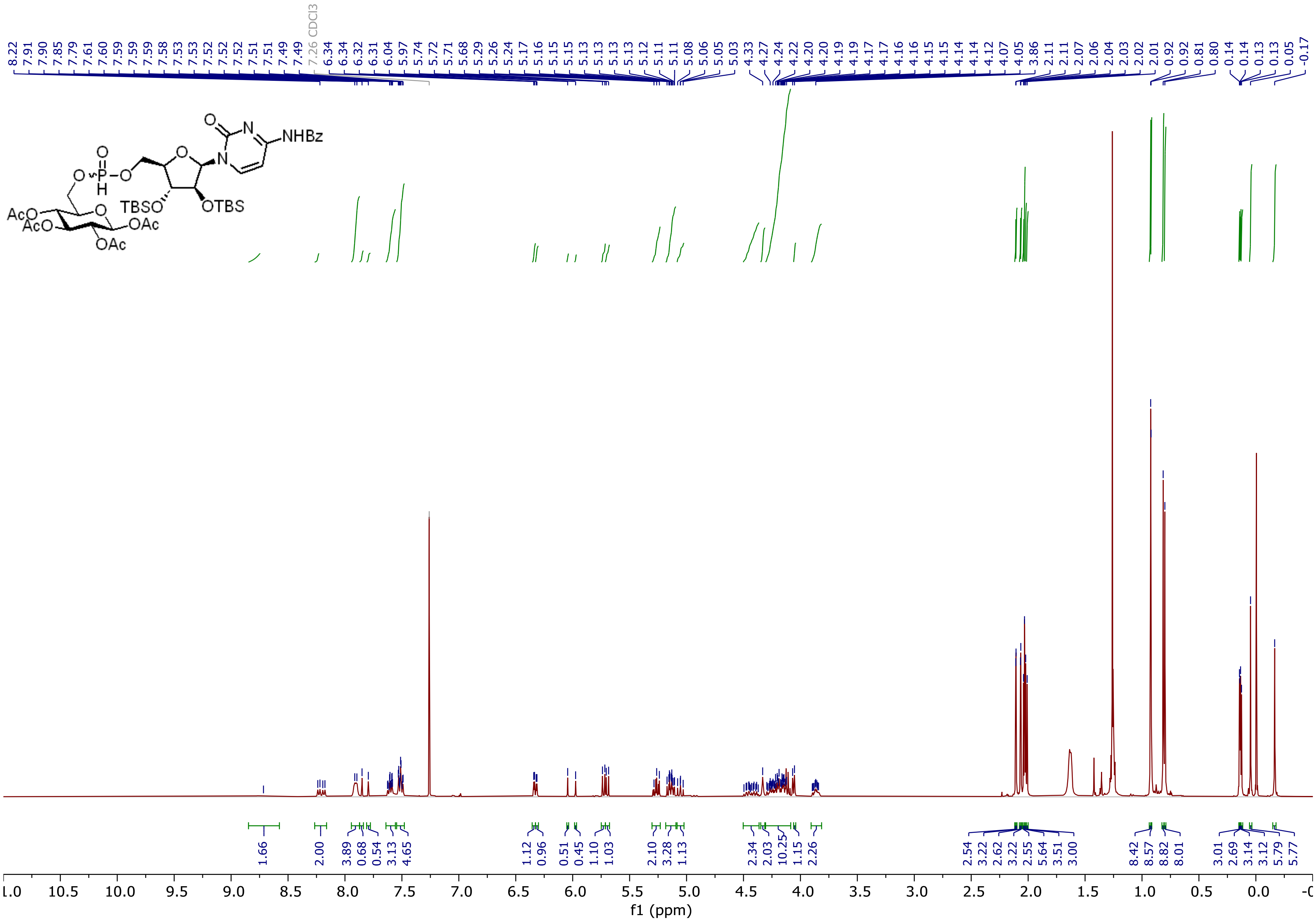

Figure S31 ^{13}C **NMR (101 MHz, $CDCl_3$): 2',3'-*O*-*tert*Butyldimethylsilyl-*N*-4-benzoyl-arabinocytidine-5'-*O*-[6''-*O*-(1'',2'',3'',4''-tetra-*O*-actetyl-β-D-glucopyranose)]-hydrogenphosphonate S10**

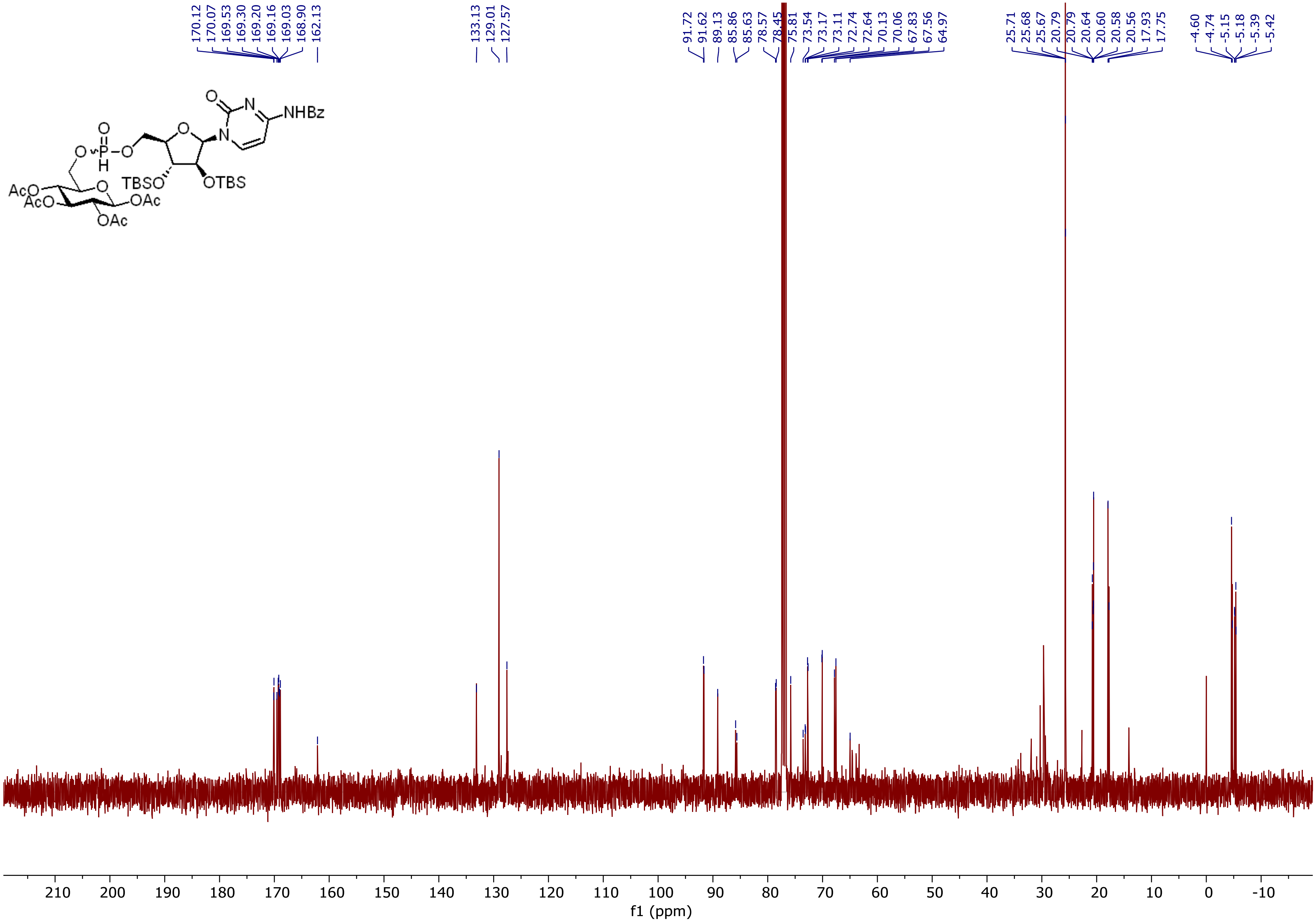

Figure S32 ^{31}P {^{1}H} NMR (162 MHz, $CDCl_3$): 2',3'-*O*-*tert*Butyldimethylsilyl-*N*-4-benzoyl-arabinocytidine-5'-*O*-[6''-*O*-(1'',2'',3'',4''-tetra-*O*-actetyl-β-D-glucopyranose)]-hydrogenphosphonate S10

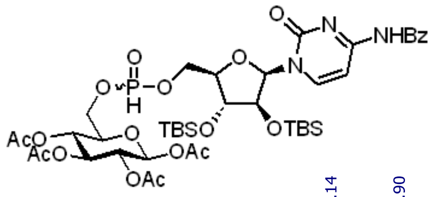

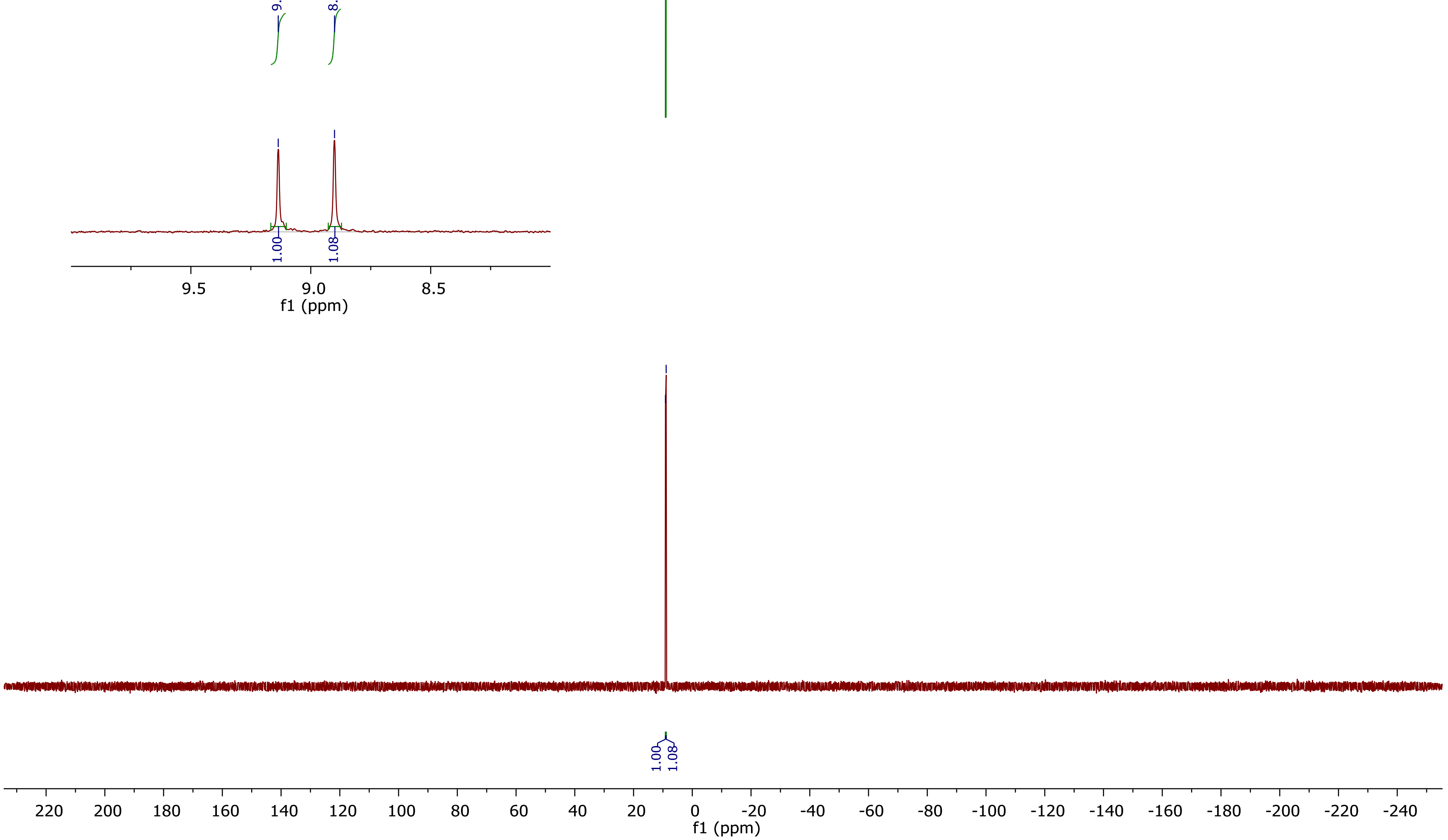

Figure S33 ^{1}H NMR (400 MHz, $CDCl_3$): 2',3'-*O*-*tert*Butyldimethylsilyl-*N*-4-benzoyl-arabinocytidine-5'-*O*-[6''-*O*-(1'',2'',3'',4''-tetra-*O*-acetyl-β-D-glucopyranose)]-phosphate triethylammonium salt 6

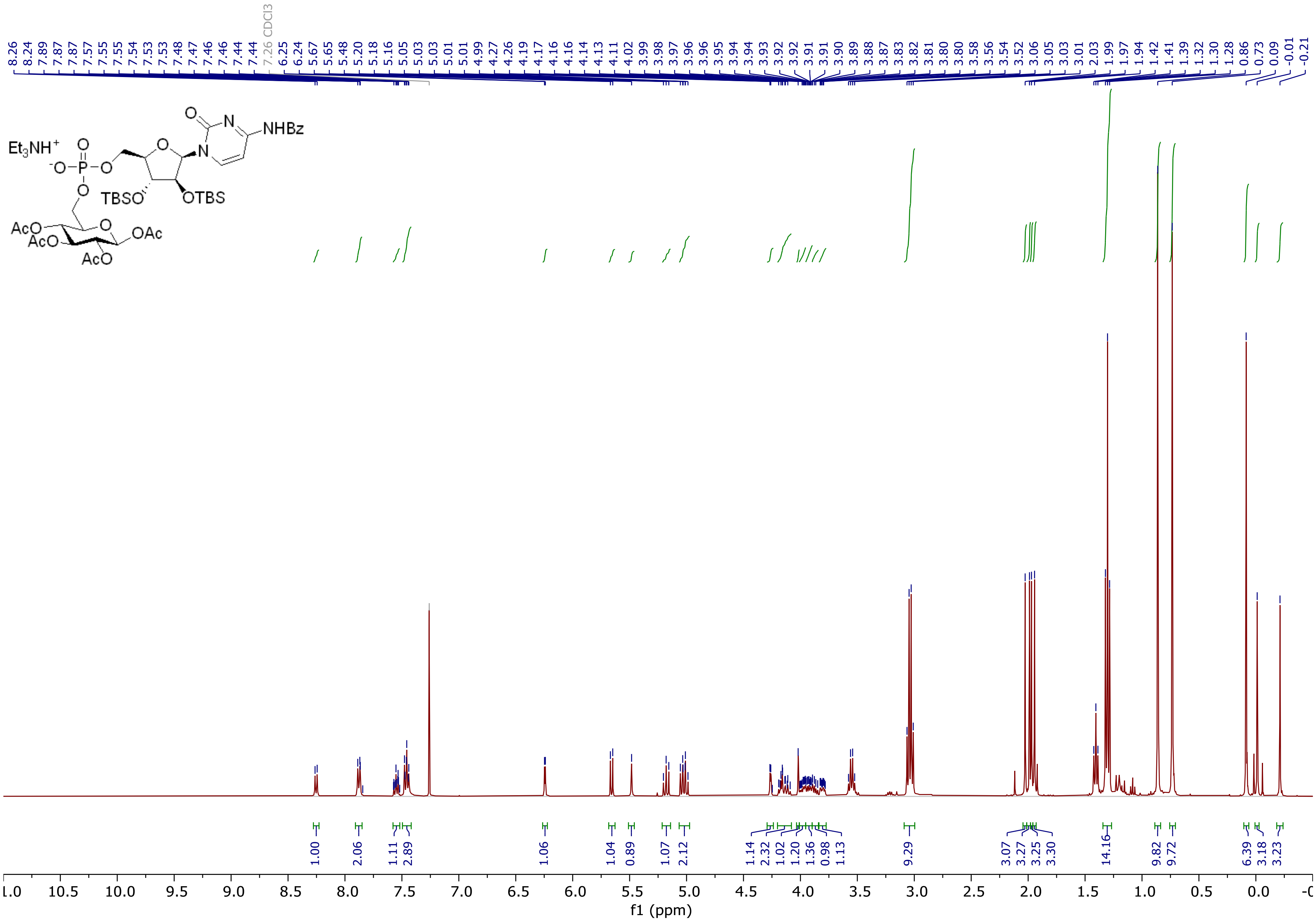

Figure S34 ^{13}C NMR (101 MHz, $CDCl_3$): 2',3'-*O*-*tert*Butyldimethylsilyl-*N*-4-benzoyl-arabinocytidine-5'-*O*-[6''-*O*-(1'',2'',3'',4''-tetra-*O*-acetyl-β-D-glucopyranose)]-phosphate triethylammonium salt 6

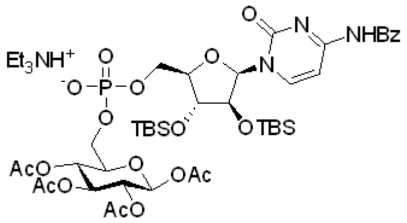

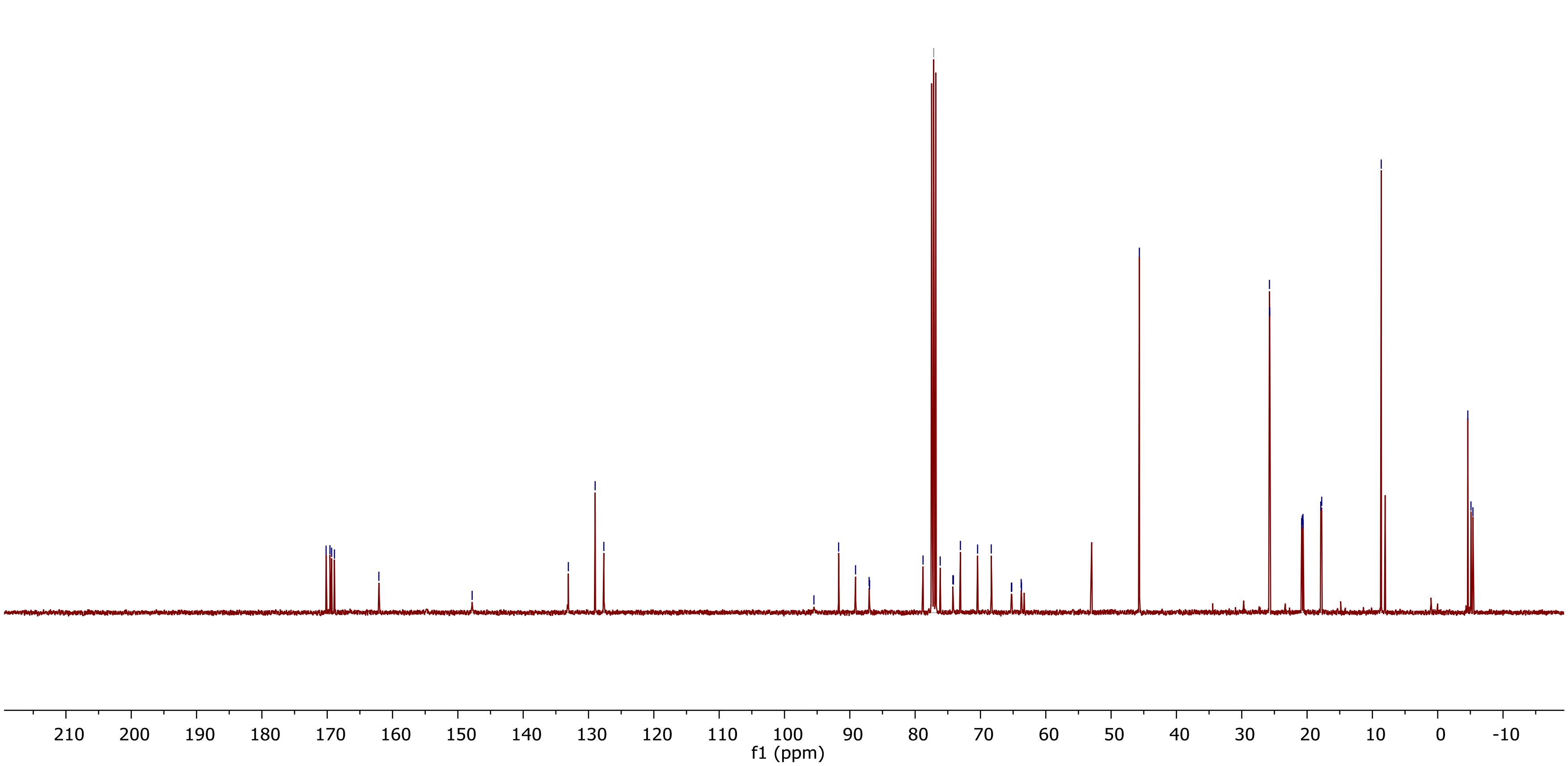

Figure S35 ^{31}P NMR (162 MHz, $CDCl_3$): 2',3'-*O*-*tert*Butyldimethylsilyl-*N*-4-benzoyl-arabinocytidine-5'-*O*-[6''-*O*-(1'',2'',3'',4''-tetra-*O*-acetyl-β-D-glucopyranose)]-phosphate triethylammonium salt 6

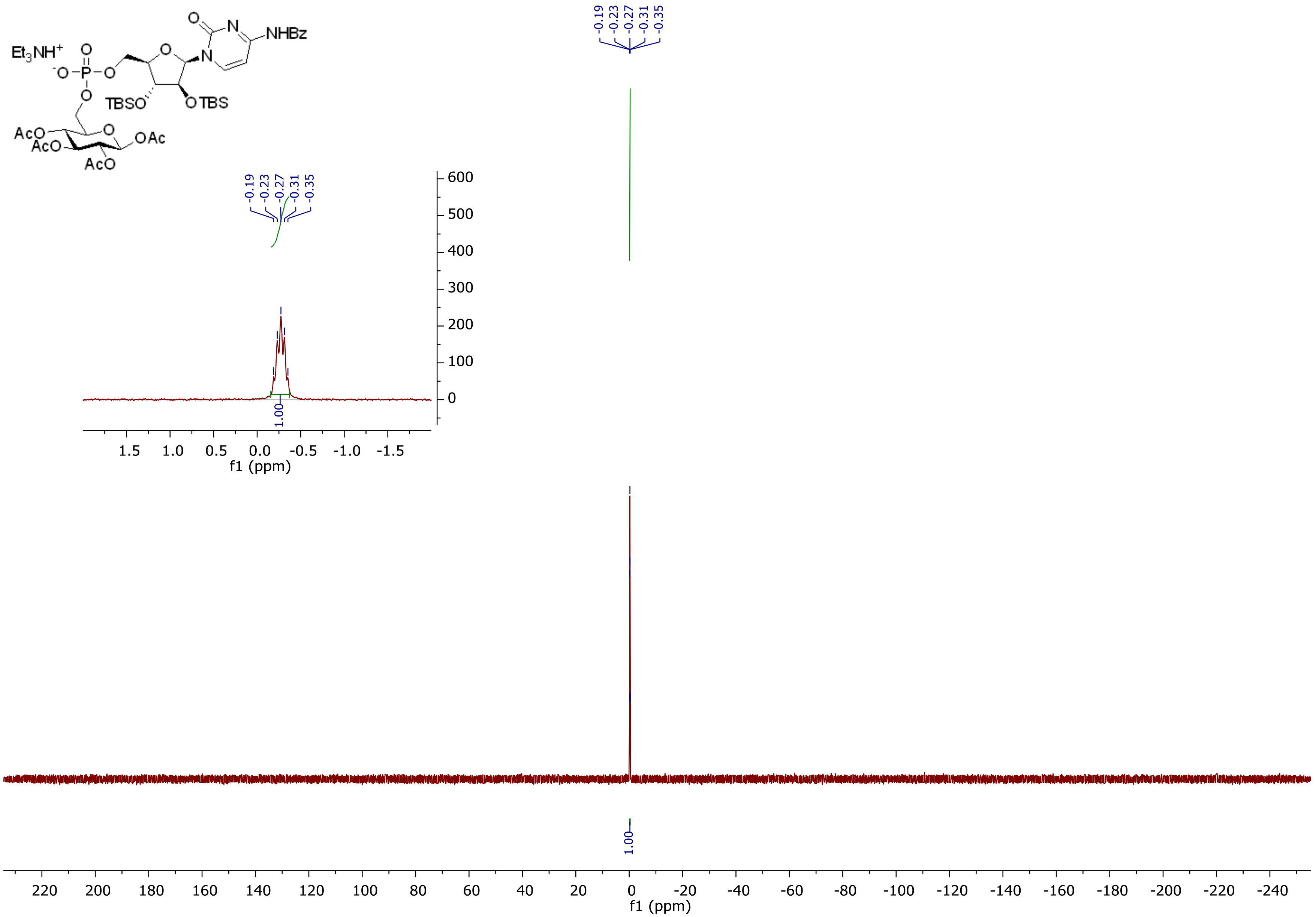

Figure S36 ^{1}H NMR (400 MHz, $CDCl_3$): 3'-*O*-*tert*Butyldimethylsilyl-*N*-4-benzoyl-2'-deoxy-2',2'-difluorocytidine-5'-*O*-[6''-*O*-(1'',2'',3'',4''-tetra-*O*-acetyl-β-D-glucopyranose)]-phosphate triethylammonium salt 7

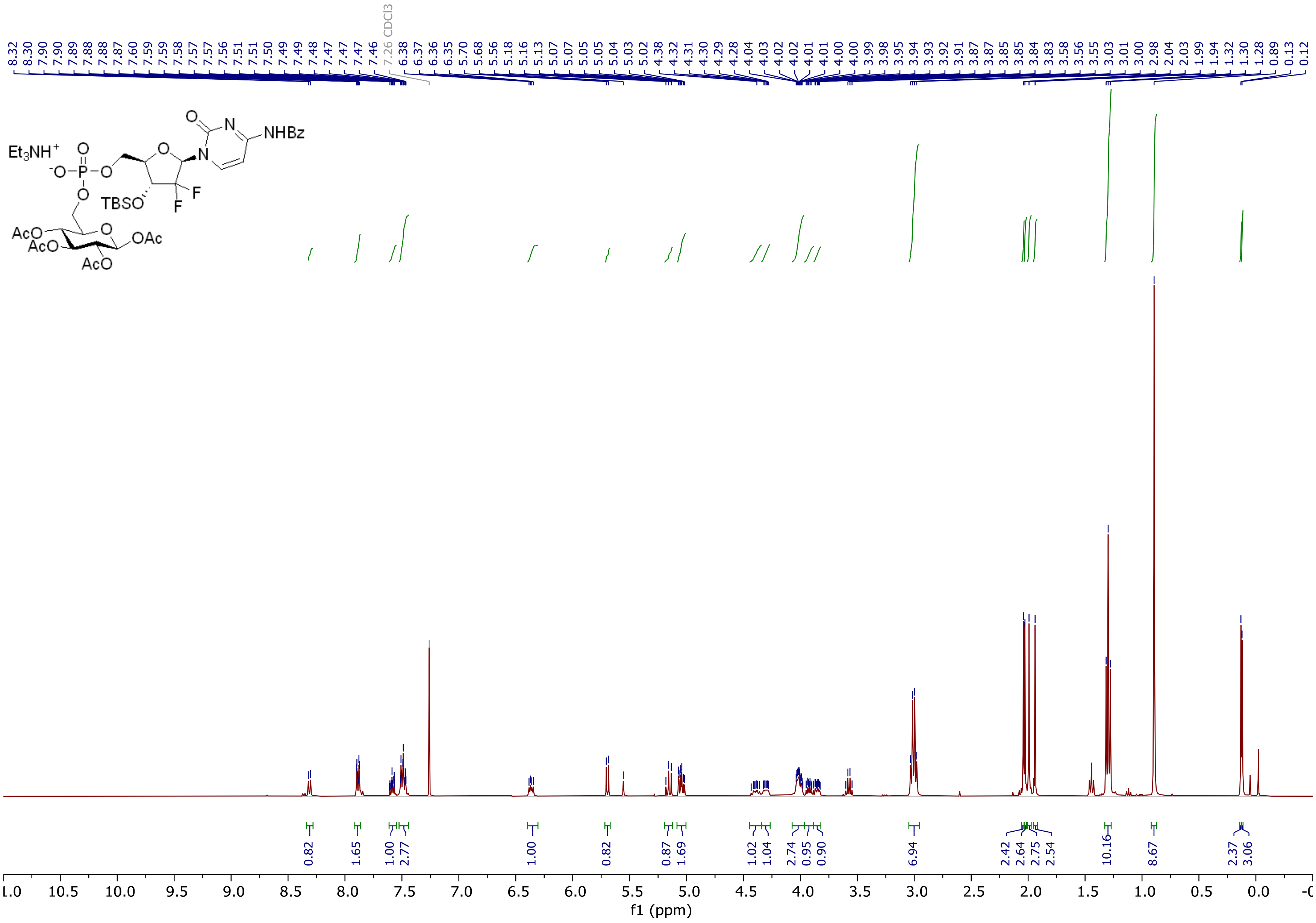

Figure S37 ^{13}C NMR (101 MHz, $CDCl_3$): 3'-*O*-*tert*Butyldimethylsilyl-*N*-4-benzoyl-2'-deoxy-2',2'-difluorocytidine-5'-*O*-[6''-*O*-(1'',2'',3'',4''-tetra-*O*-acetyl-β-D-glucopyranose)]-phosphate triethylammonium salt 7

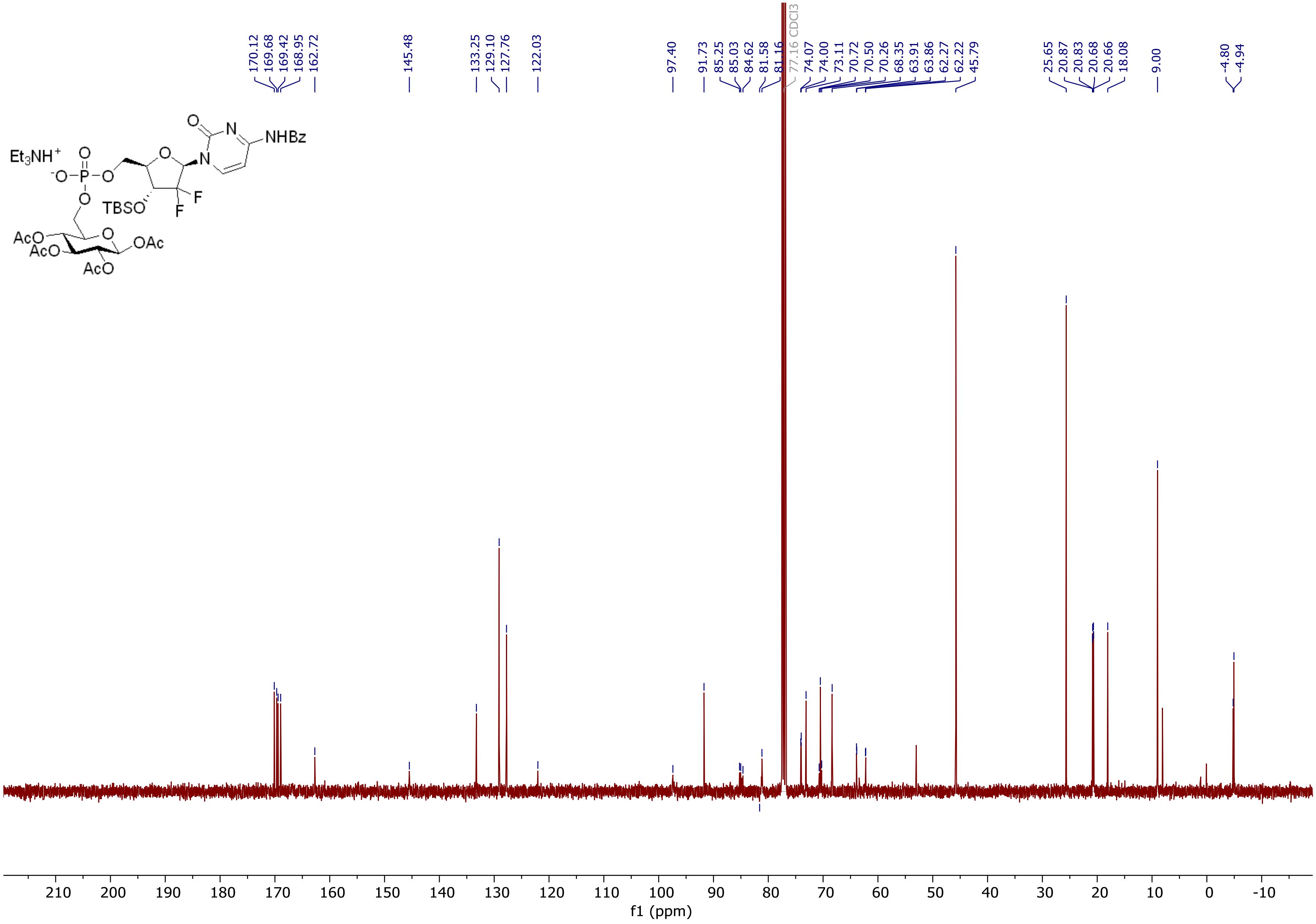

Figure S38 ^{19}F {^{1}H} NMR (377 MHz, $CDCl_3$): 3'-*O*-*tert*Butyldimethylsilyl-*N*-4-benzoyl-2'-deoxy-2',2'-difluorocytidine-5'-*O*-[6''-*O*-(1'',2'',3'',4''-tetra-*O*-acetyl-β-D-glucopyranose)]-phosphate triethylammonium salt 7

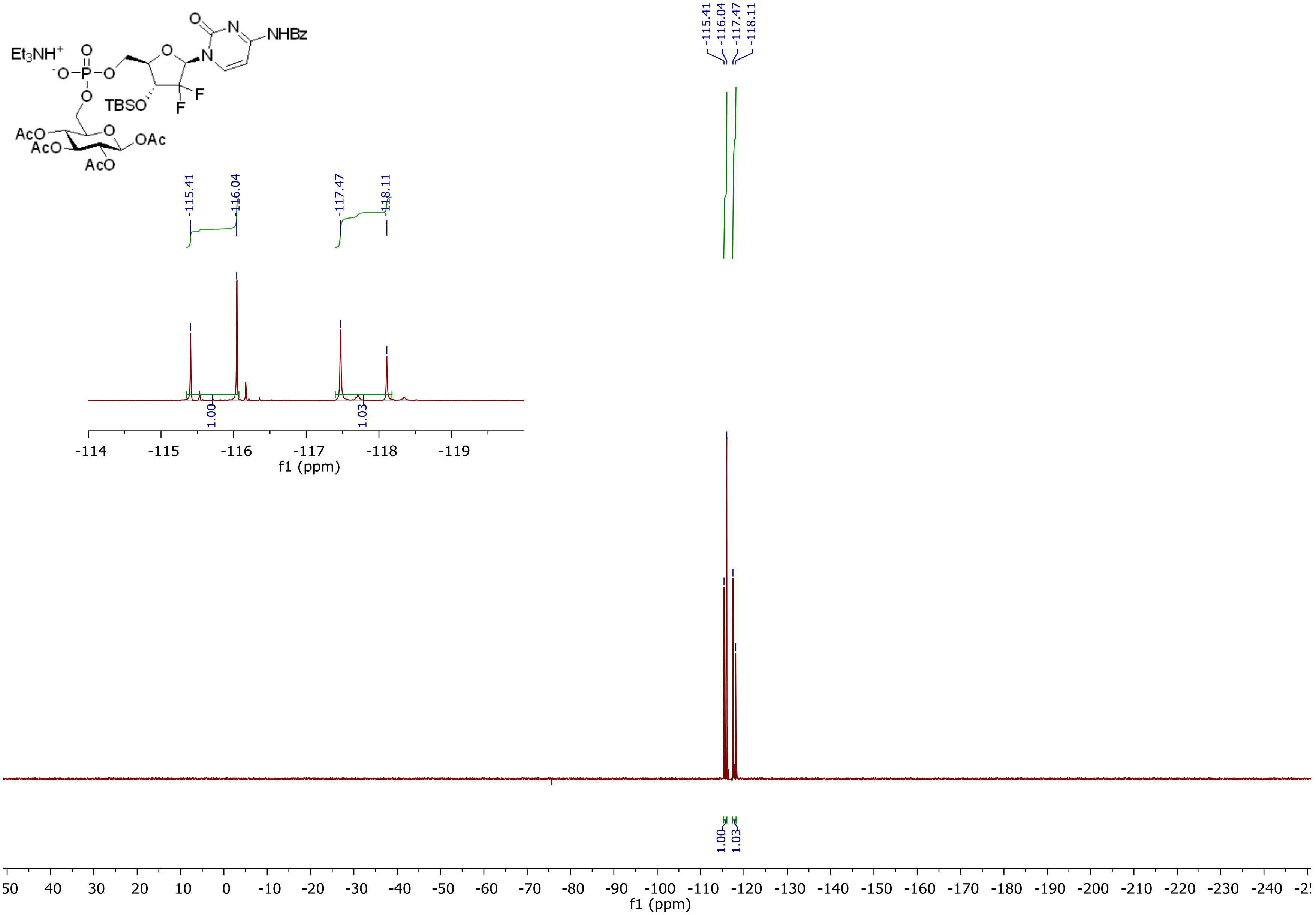

Figure S39 ^{31}P {^{1}H} NMR (162 MHz, CDCl$_3$): 3'-*O*-*tert*Butyldimethylsilyl-*N*-4-benzoyl-2'-deoxy-2',2'-difluorocytidine-5'-*O*-[6''-*O*-(1'',2'',3'',4''-tetra-*O*-acetyl-β-D-glucopyranose)]-phosphate triethylammonium salt 7

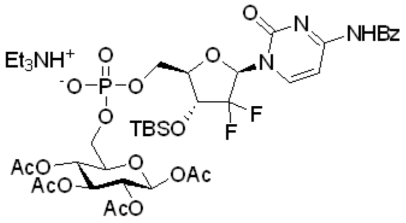

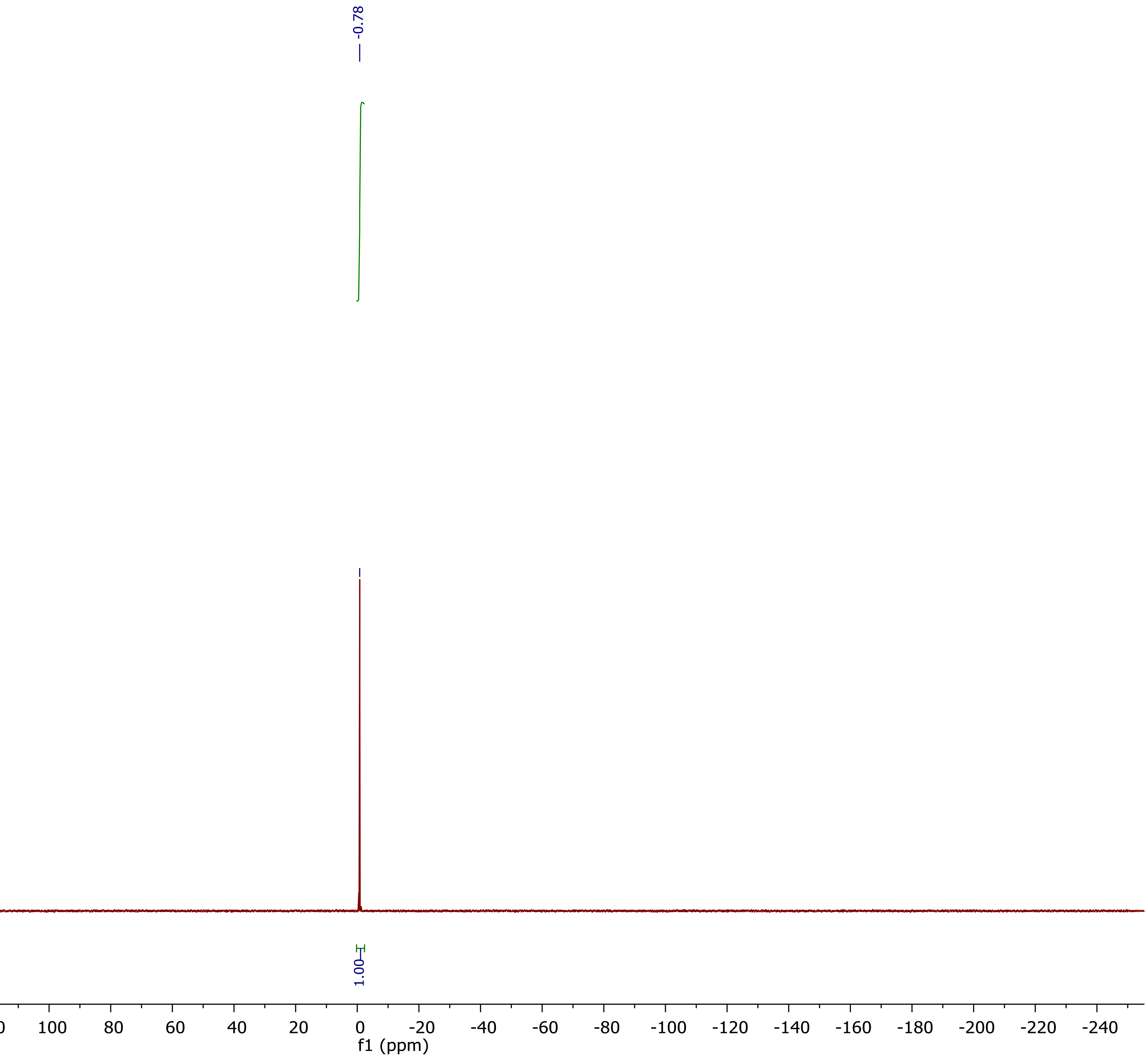

Figure S40 ^{1}H NMR (400 MHz, D_2O): Arabinocytidine-5'-*O*-(6''-*O*-α/β-D-glucopyranose)-phosphate sodium salt 8

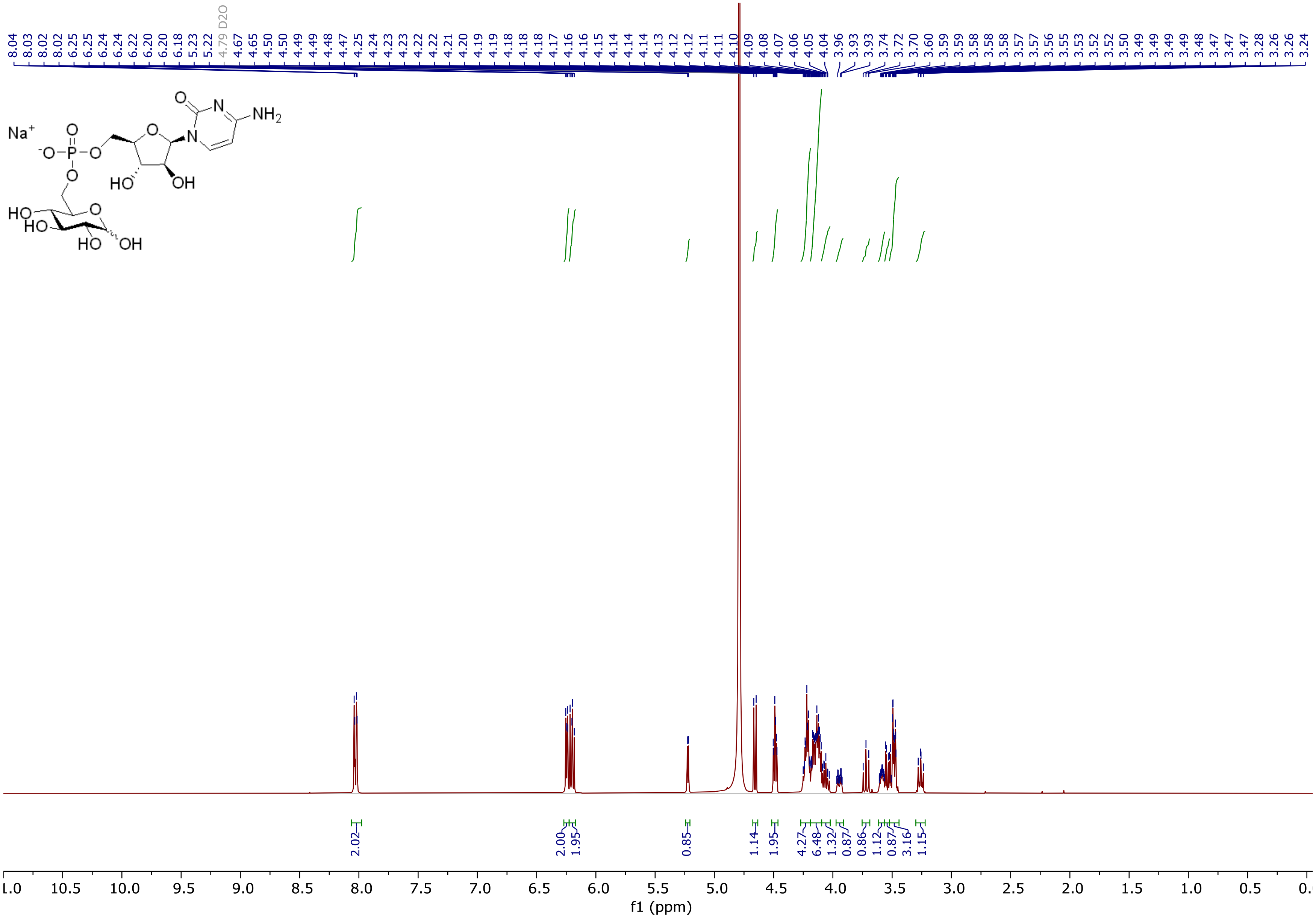

Figure S41 ^{13}C NMR (101 MHz, D_2O): Arabinocytidine-5'-*O*-(6''-*O*-α/β-D-glucopyranose)-phosphate sodium salt 8

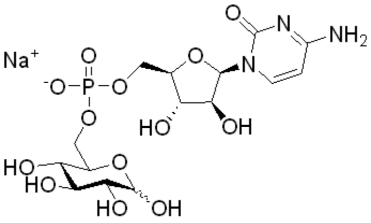

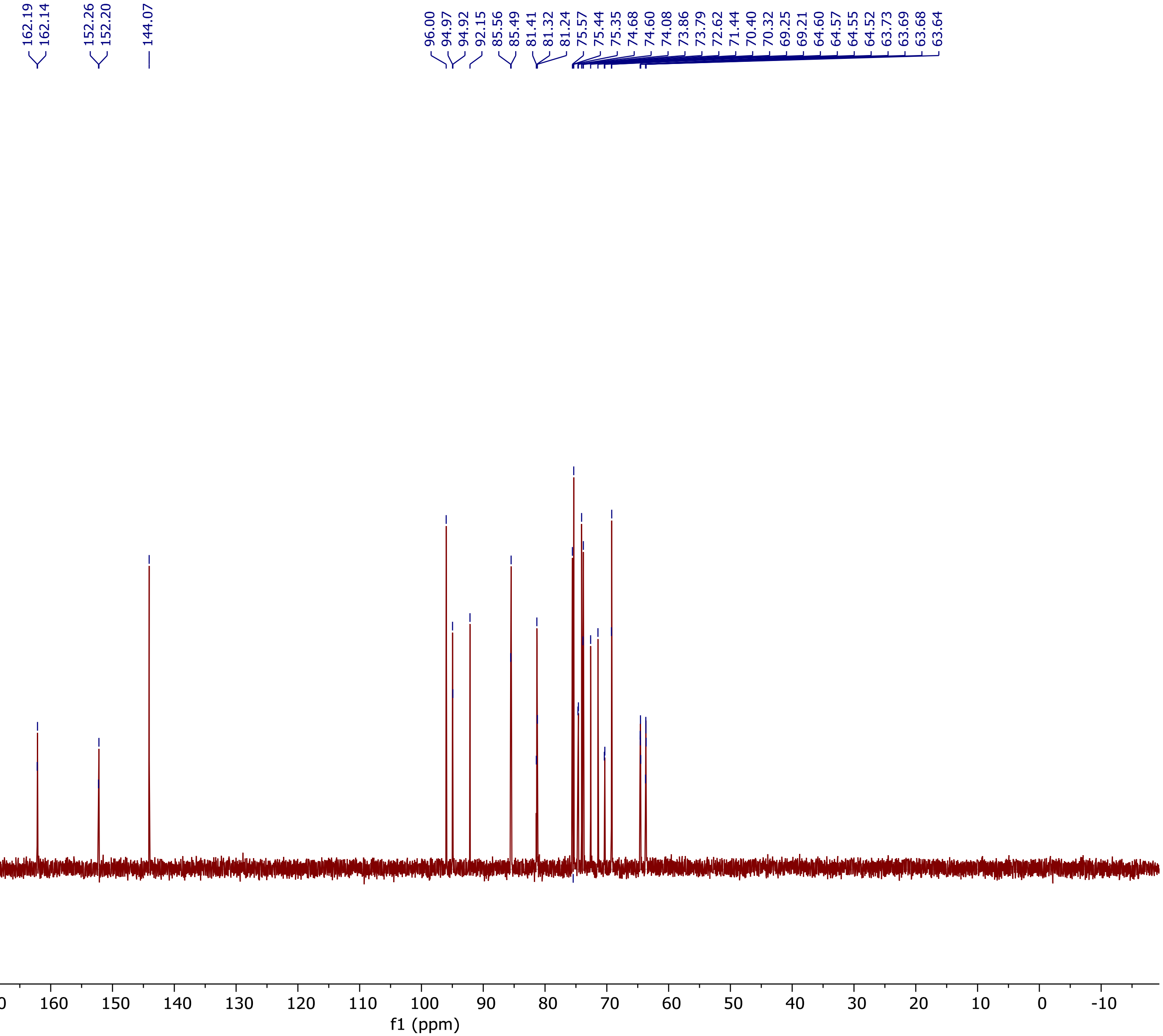

Figure S42 ^{31}P NMR (162 MHz, D_2O): Arabinocytidine-5'-*O*-(6''-*O*-α/β-D-glucopyranose)-phosphate sodium salt 8

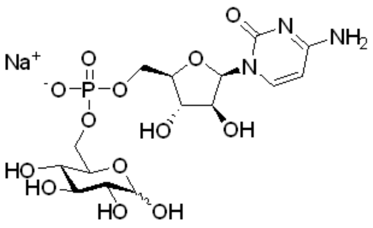

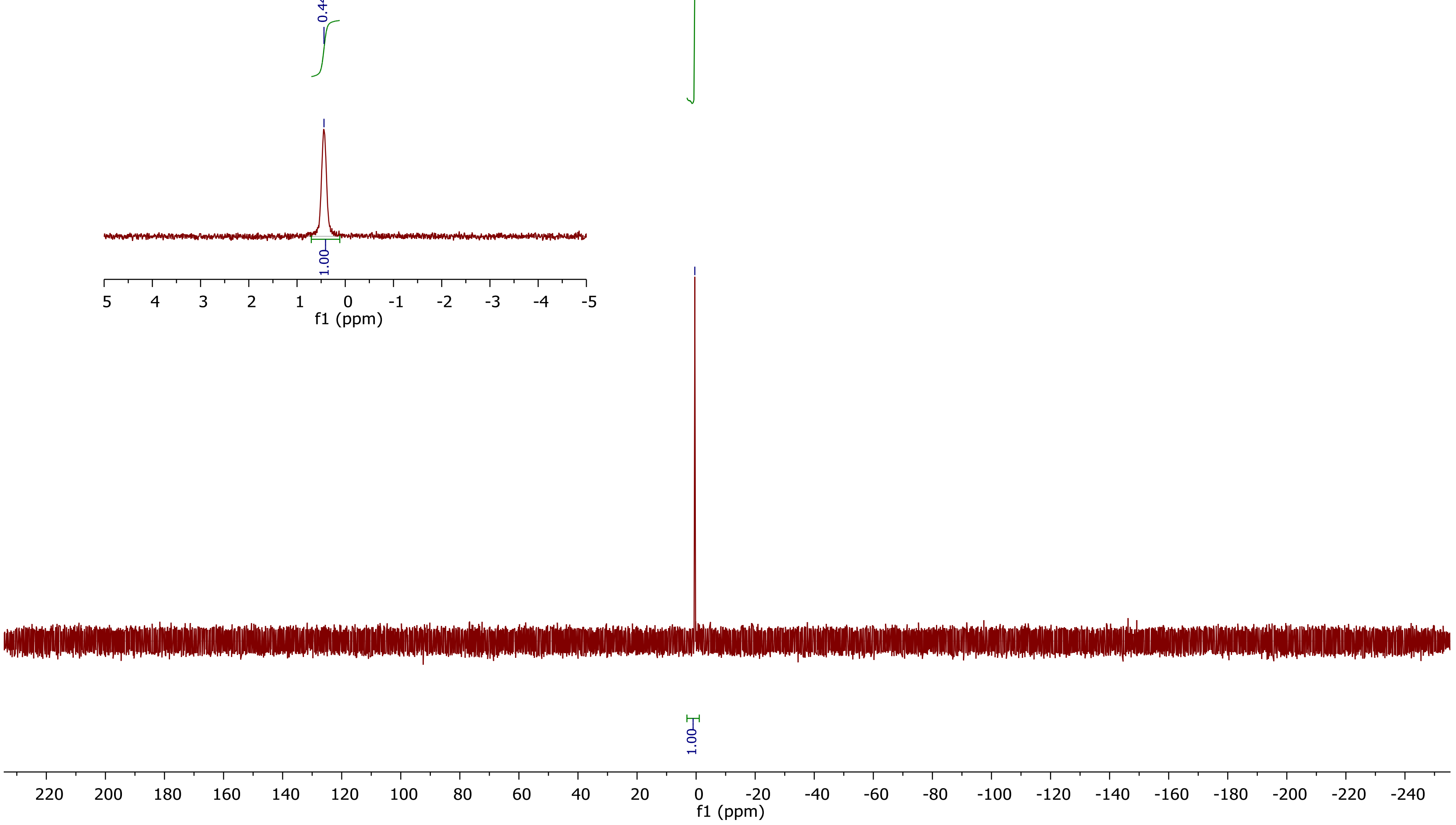

Figure S43 ^{1}H NMR (400 MHz, D_2O): 2’-Deoxy-2’,2’-difluorocytidine-5'-*O*-(6''-*O*-β-D-glucopyranose)-phosphate sodium salt 9

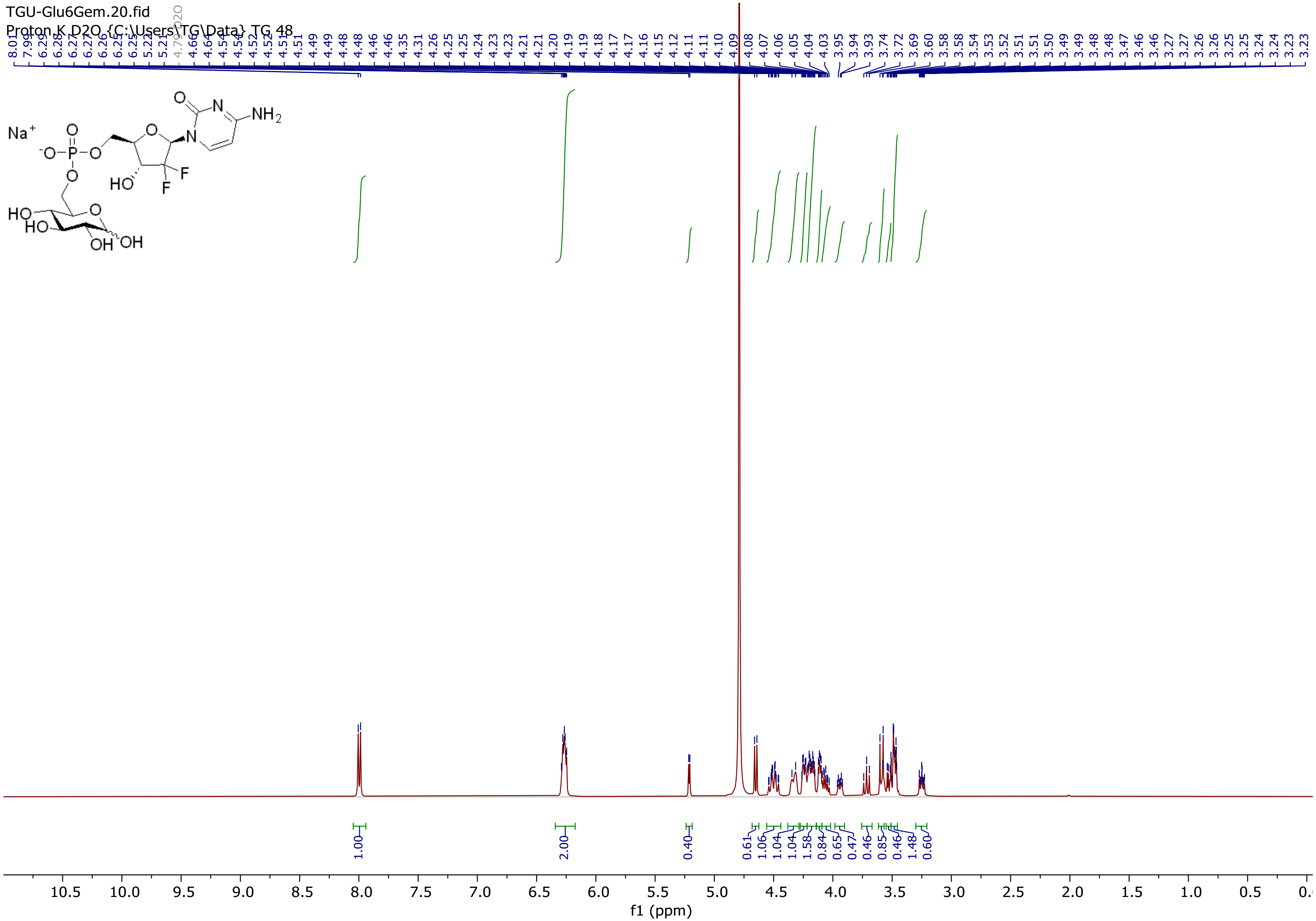

Figure S44 ^{13}C NMR (101 MHz, D_2O): 2'-Deoxy-2',2'-difluorocytidine-5'-*O*-(6''-*O*-β-D-glucopyranose)-phosphate sodium salt 9

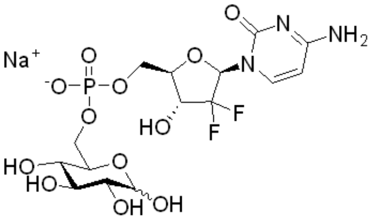

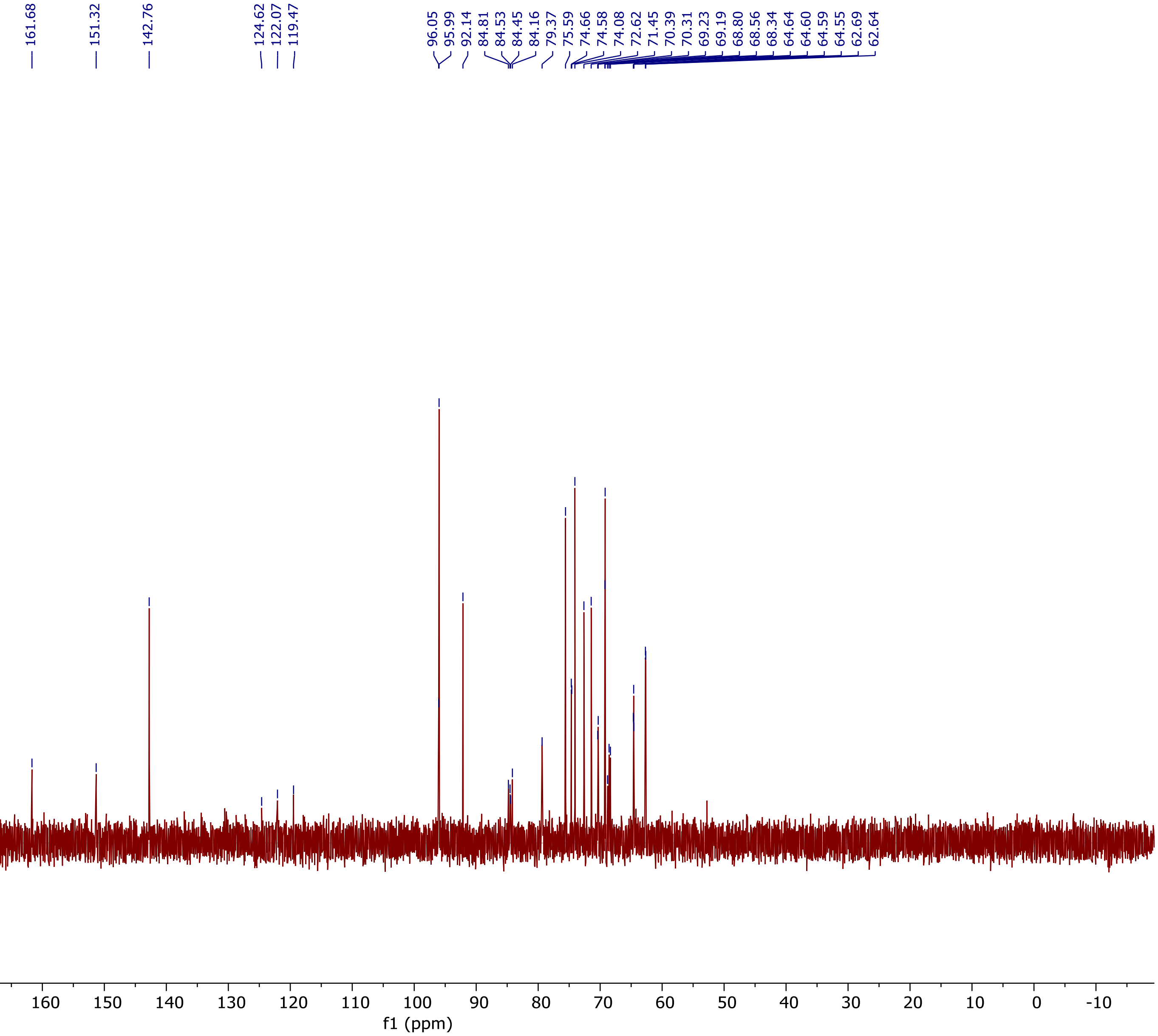

Figure S45 ^{19}F {^1H} NMR (377 MHz, D_2O): 2'-Deoxy-2',2'-difluorocytidine-5'-*O*-(6''-*O*-β-D-glucopyranose)-phosphate sodium salt 9

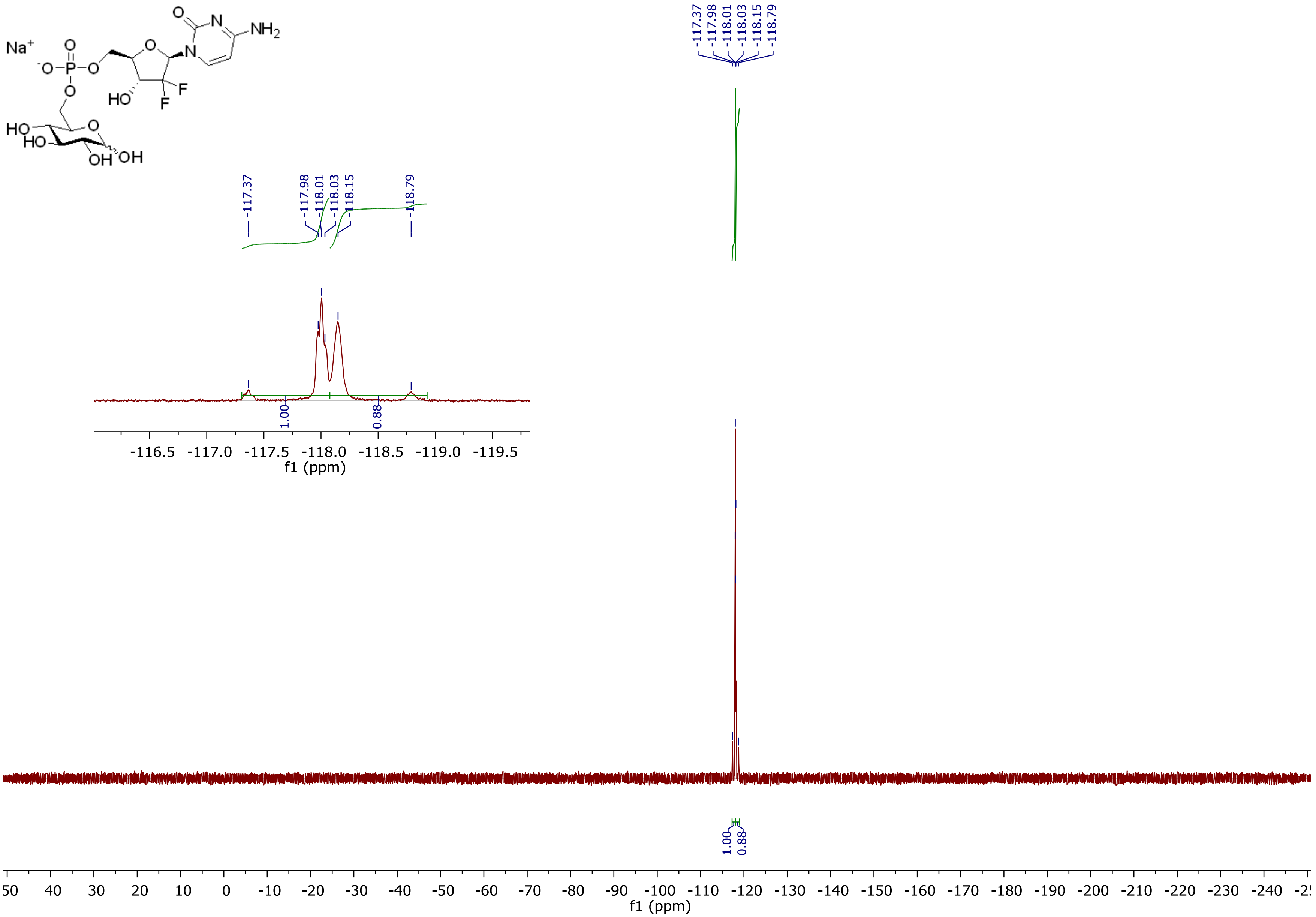

Figure S46 $^{31}P\ \{^{1}H\}$ NMR (162 MHz, D_2O): 2'-Deoxy-2',2'-difluorocytidine-5'-*O*-(6''-*O*-β-D-glucopyranose)-phosphate sodium salt 9

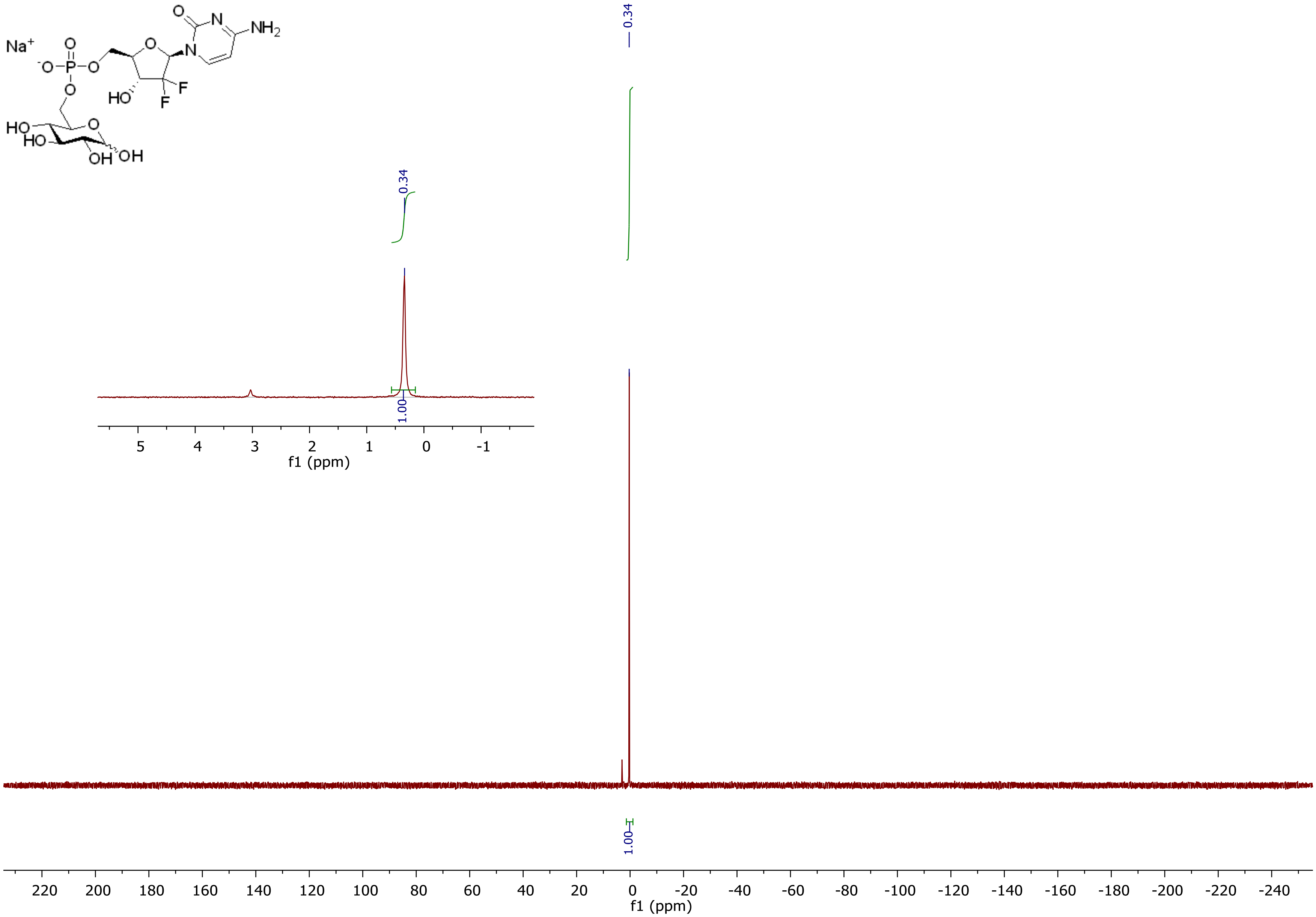

Figure S47 **^{1}H NMR (400 MHz, $CDCl_3$): 2,3,4,6-Tetra-*O*-acetyl-β-D-glucopyranosyl-1-*O*-hydrogenphosphonate triethylammonium salt 11**

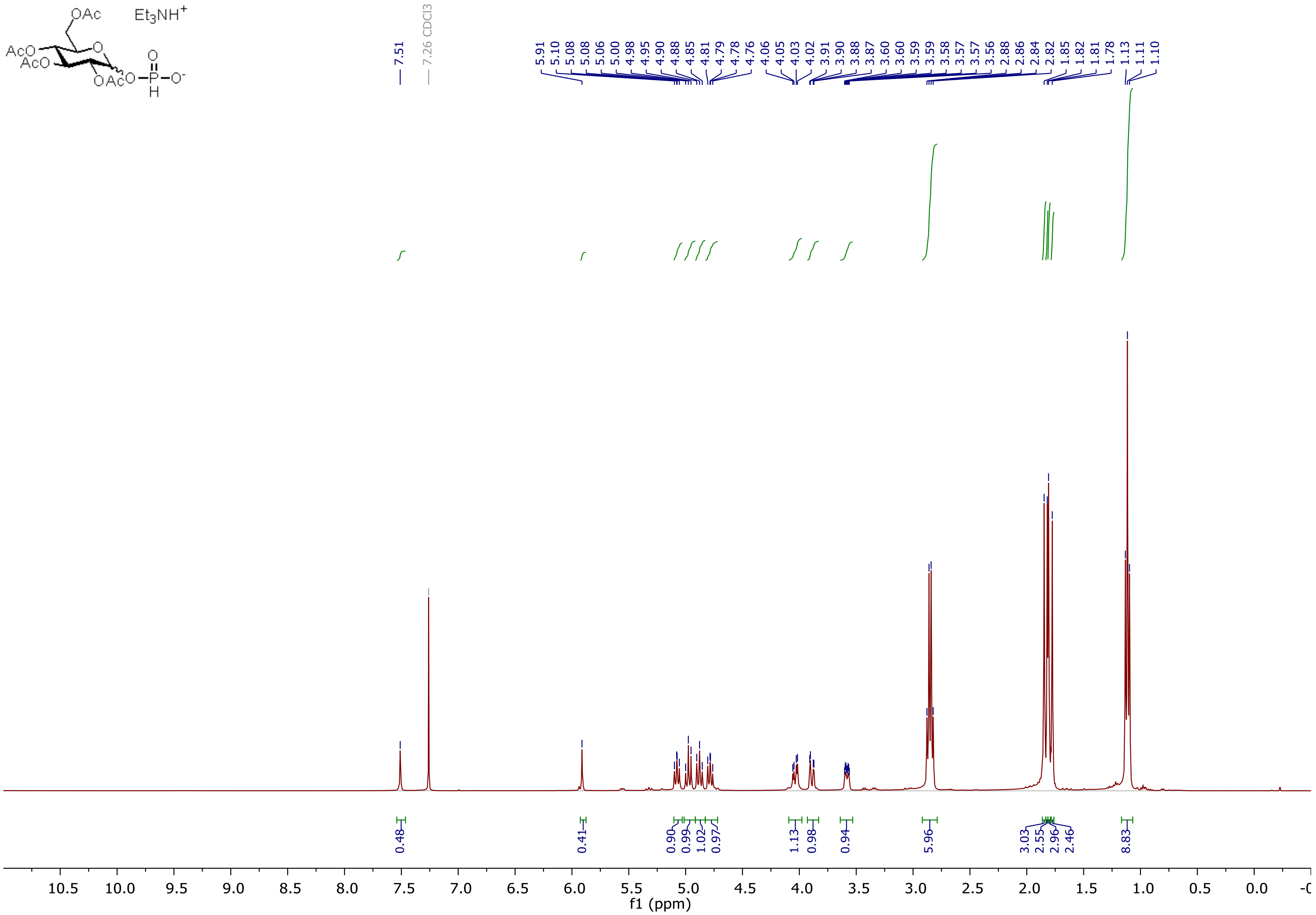

Figure S48 ^{13}C NMR (101 MHz, $CDCl_3$): 2,3,4,6-Tetra-*O*-acetyl-β-D-glucopyranosyl-1-*O*-hydrogenphosphonate triethylammonium salt 11

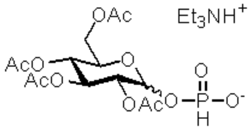

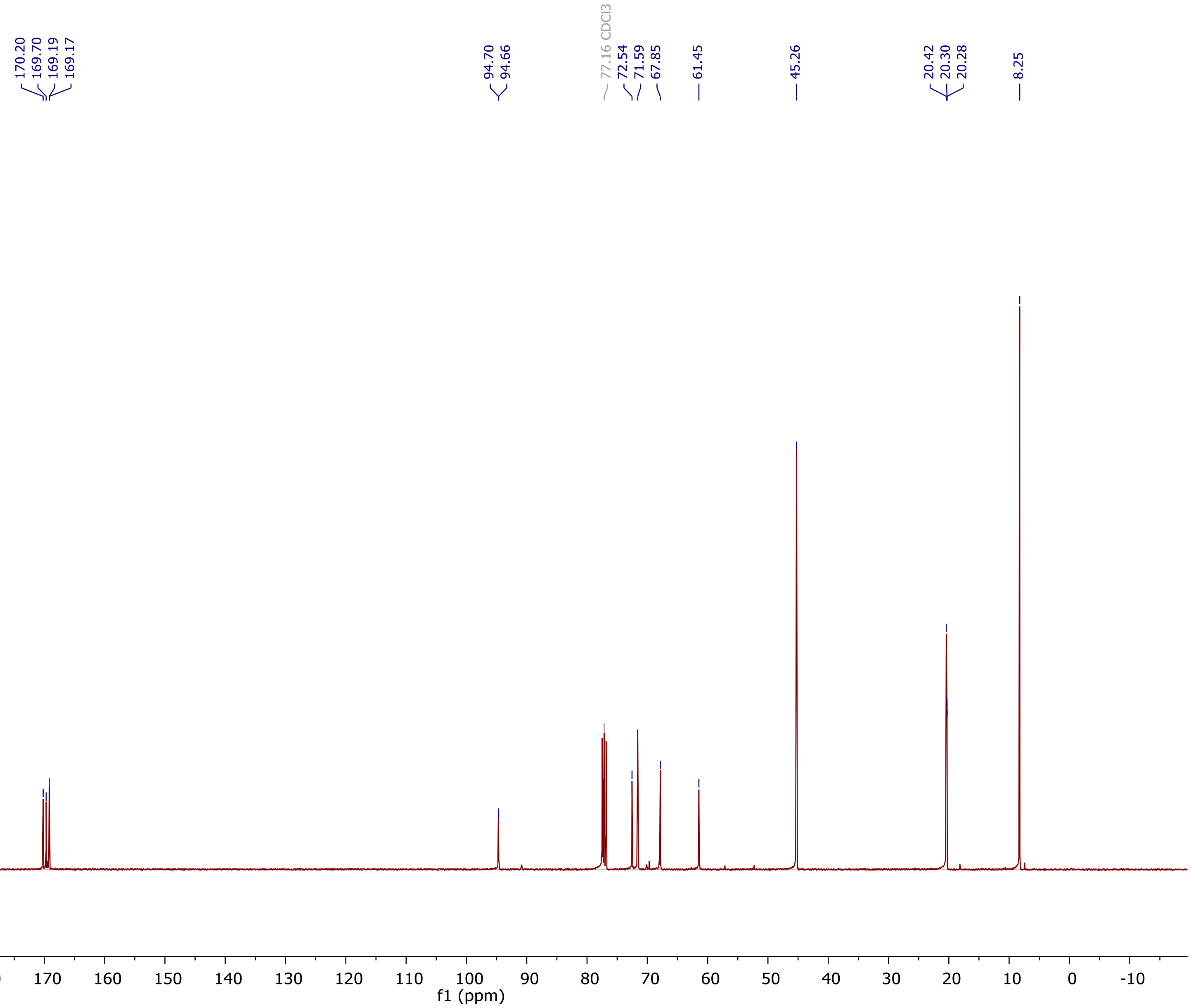

Figure S49 **^{31}P NMR (162 MHz, $CDCl_3$): 2,3,4,6-Tetra-*O*-acetyl-β-D-glucopyranosyl-1-*O*-hydrogenphosphonate triethylammonium salt 11**

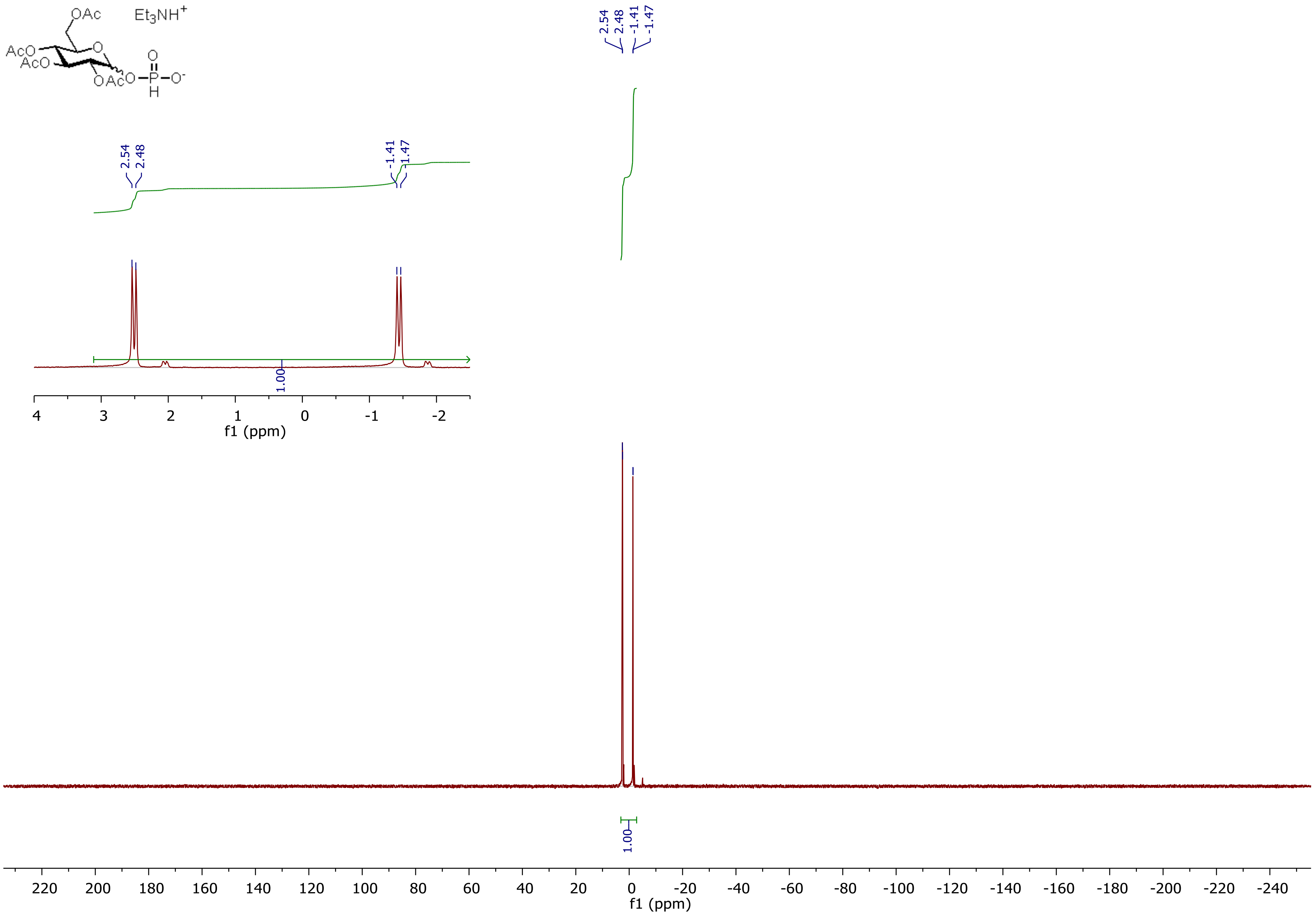

Figure S50 ^{1}H NMR (400 MHz, $CDCl_3$): 2',3'-*O*-*tert*Butyldimethylsilyl-*N*-4-benzoyl-arabinocytidine-5'-*O*-[1''-*O*-(2'',3'',4'',6''-tetra-*O*-acetyl-α/β-D-glucopyranose)]-phosphate triethylammonium salt 12

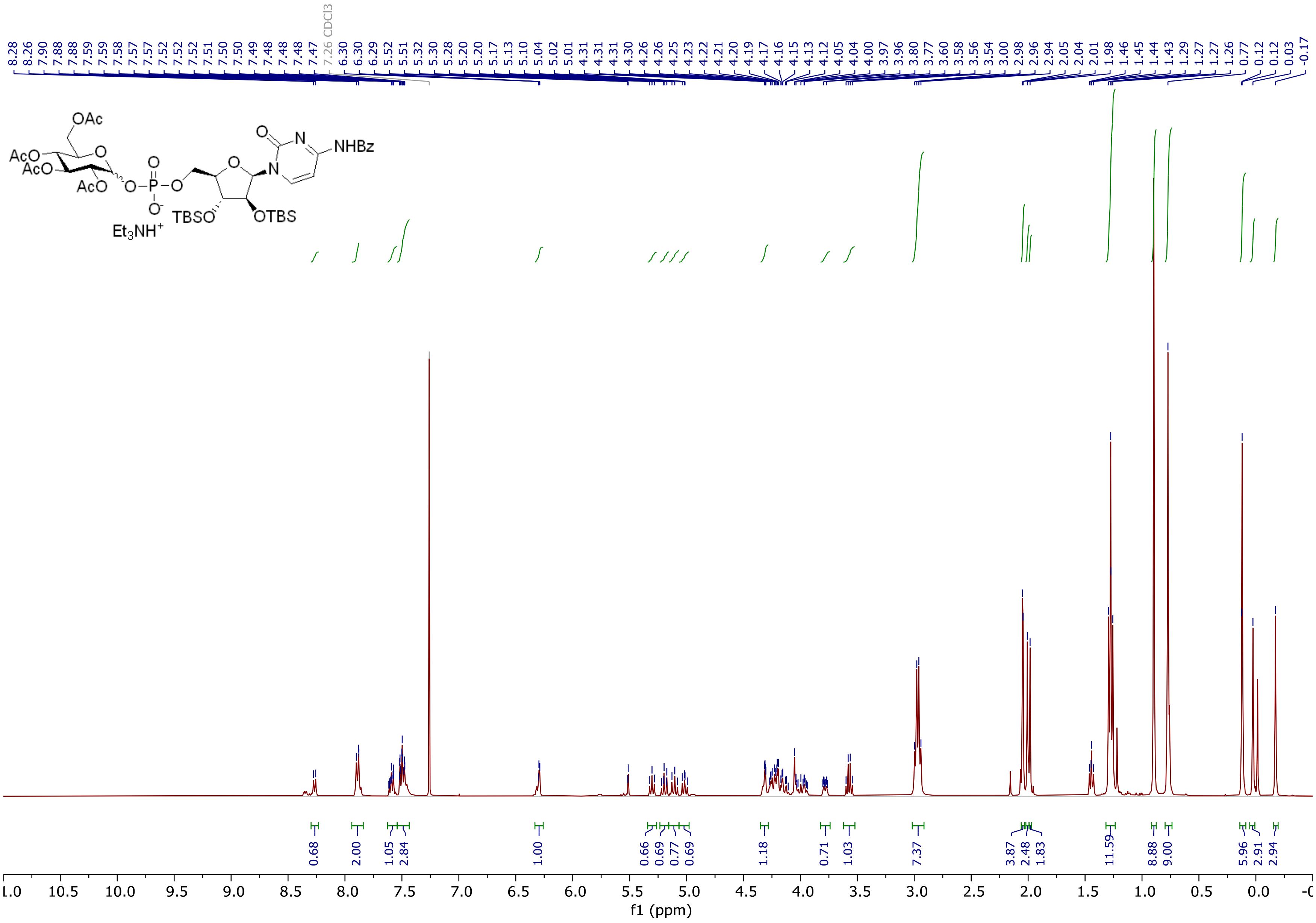

Figure S51 ^{13}C NMR (101 MHz, $CDCl_3$): 2',3'-*O*-*tert*Butyldimethylsilyl-*N*-4-benzoyl-arabinocytidine-5'-*O*-[1''-*O*-(2'',3'',4'',6''-tetra-*O*-acetyl-α/β-D-glucopyranose)]-phosphate triethylammonium salt 12

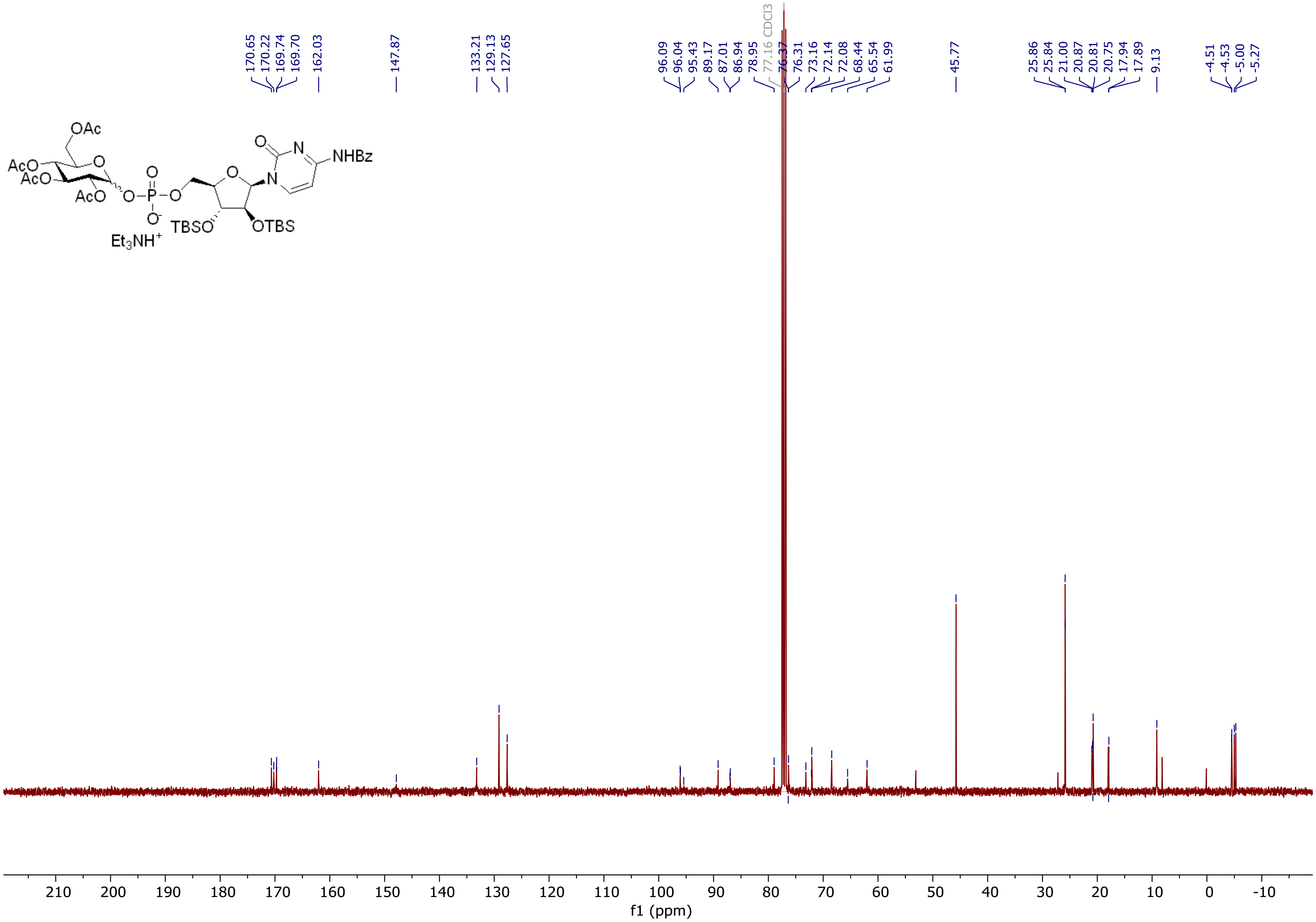

Figure S52 $^{31}P\{H^1\}$ NMR (162 MHz, $CDCl_3$): 2',3'-*O*-*tert*Butyldimethylsilyl-*N*-4-benzoyl-arabinocytidine-5'-*O*-[1''-*O*-(2'',3'',4'',6''-tetra-*O*-acetyl-α/β-D-glucopyranose)]-phosphate triethylammonium salt 12

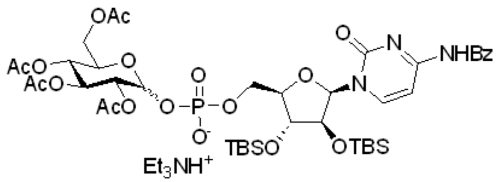

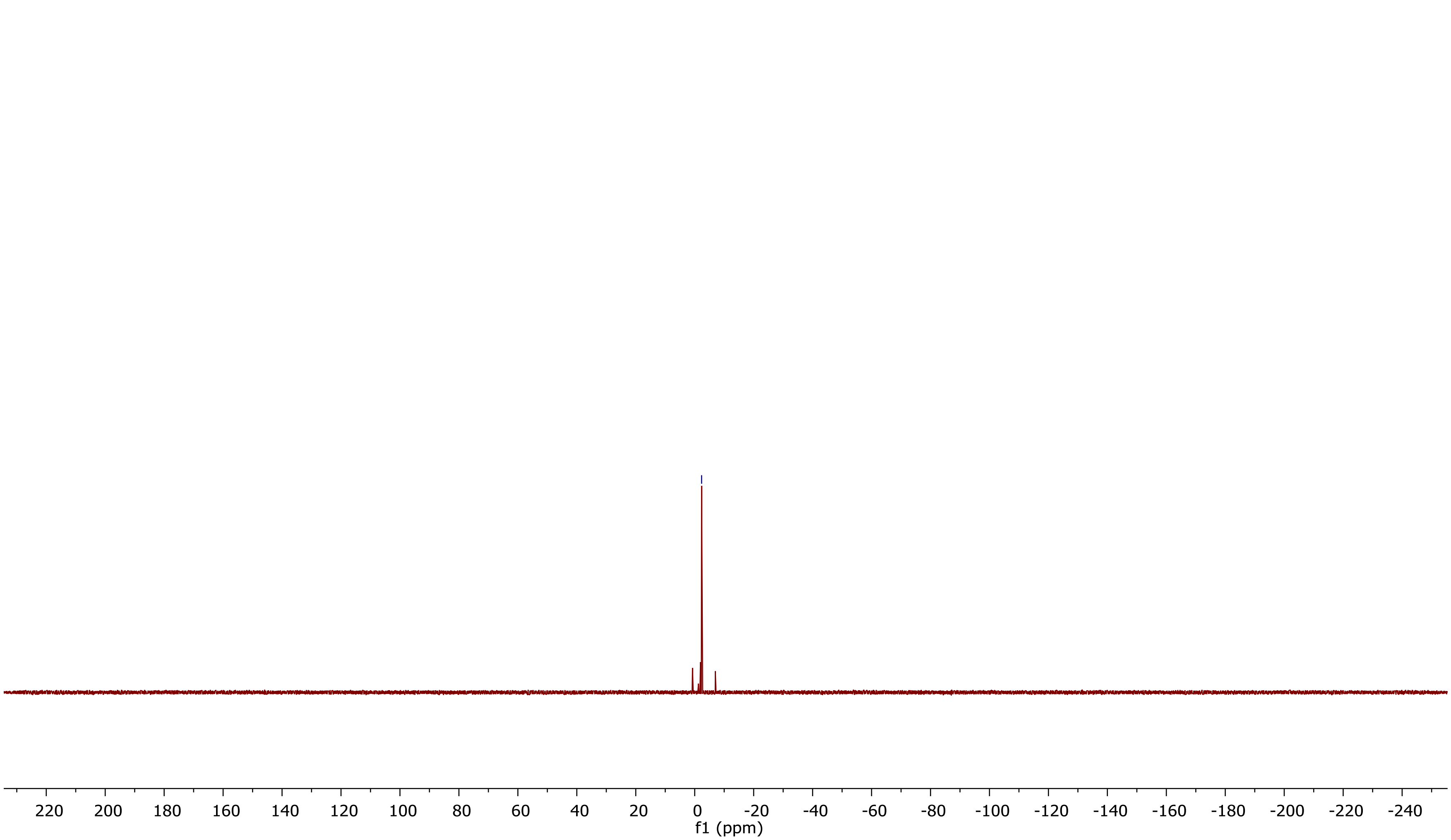

Figure S53 ^{1}H NMR (400 MHz, $CDCl_3$): 3'-*O*-*tert*Butyldimethylsilyl-*N*-4-benzoyl-2'-deoxy-2',2'-difluorocytidine-5'-*O*-[1''-*O*-(2'',3'',4'',6''-tetra-*O*-acetyl-α/β-D-glucopyranose)]-phosphate triethylammonium salt 13

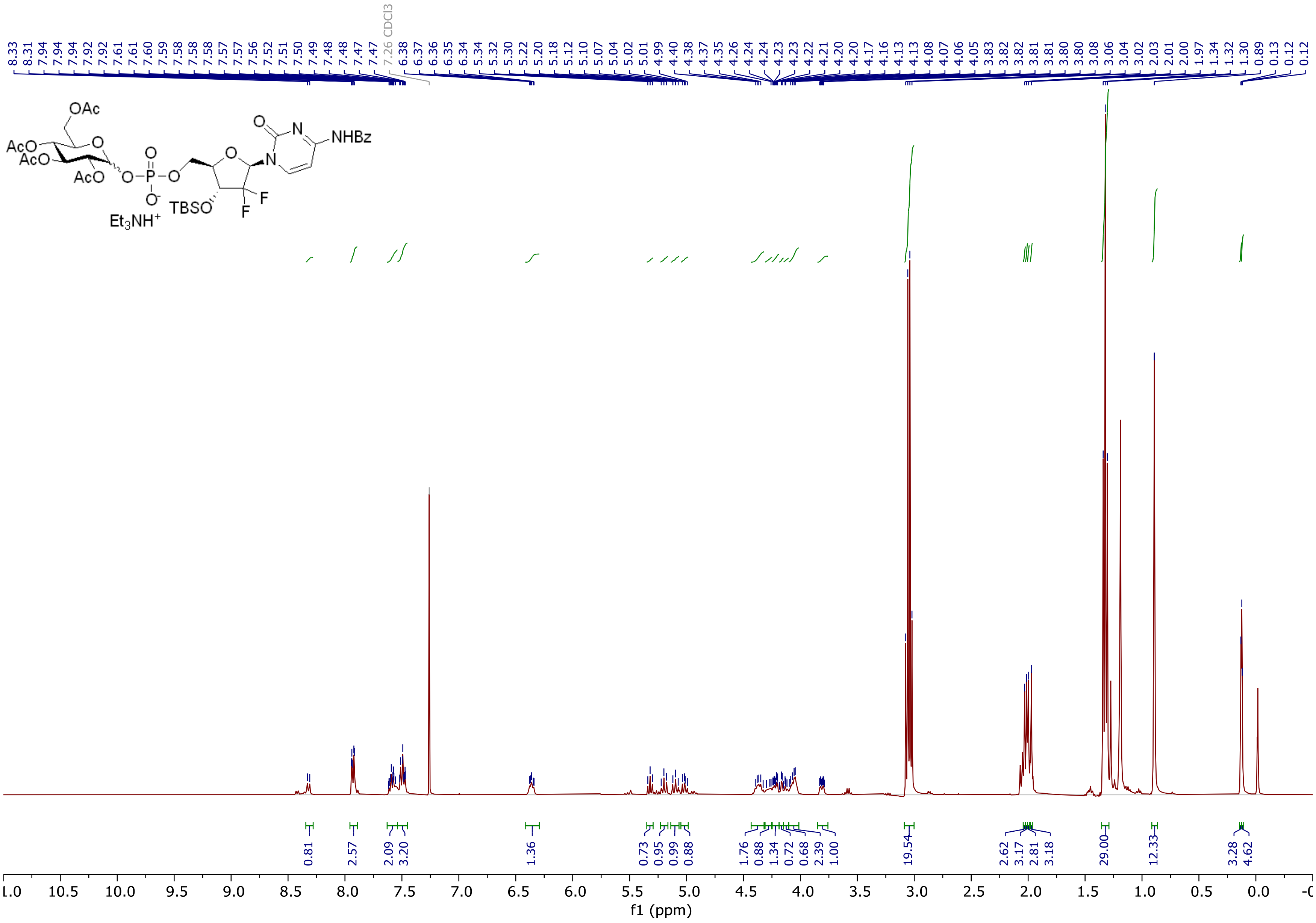

Figure S54 ^{13}C NMR (101 MHz, $CDCl_3$): 3'-*O*-*tert*Butyldimethylsilyl-*N*-4-benzoyl-2’-deoxy-2’,2’-difluorocytidine-5'-*O*-[1''-*O*-(2'',3'',4'',6''-tetra-*O*-acetyl-α/β-D-glucopyranose)]-phosphate triethylammonium salt 13

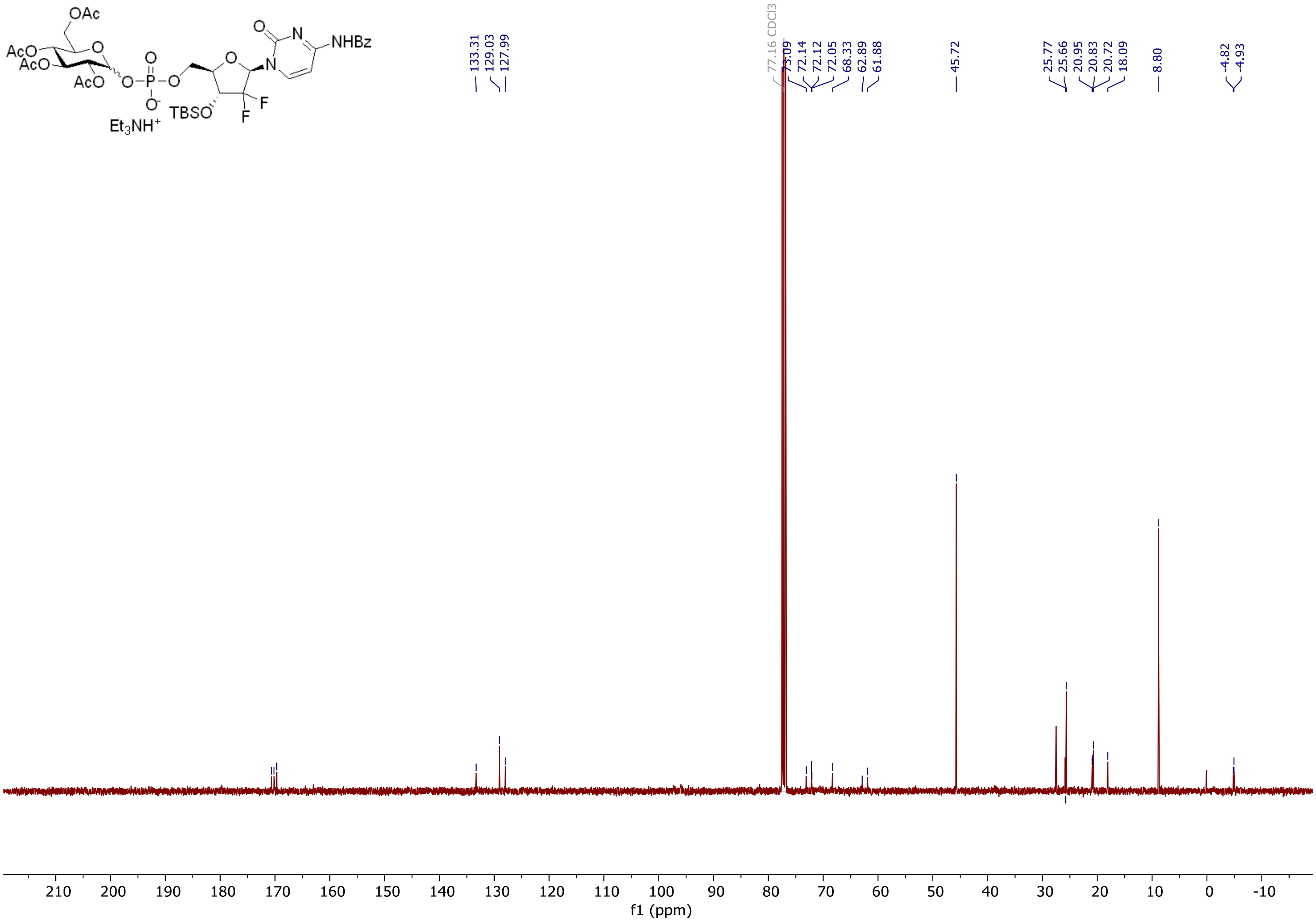

Figure S55 $^{19}F\{^{1}H\}$ NMR (377 MHz, $CDCl_3$): 3'-*O*-*tert*Butyldimethylsilyl-*N*-4-benzoyl-2'-deoxy-2',2'-difluorocytidine-5'-*O*-[1''-*O*-(2'',3'',4'',6''-tetra-*O*-acetyl-α/β-D-glucopyranose)]-phosphate triethylammonium salt 13

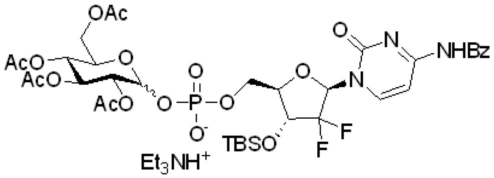

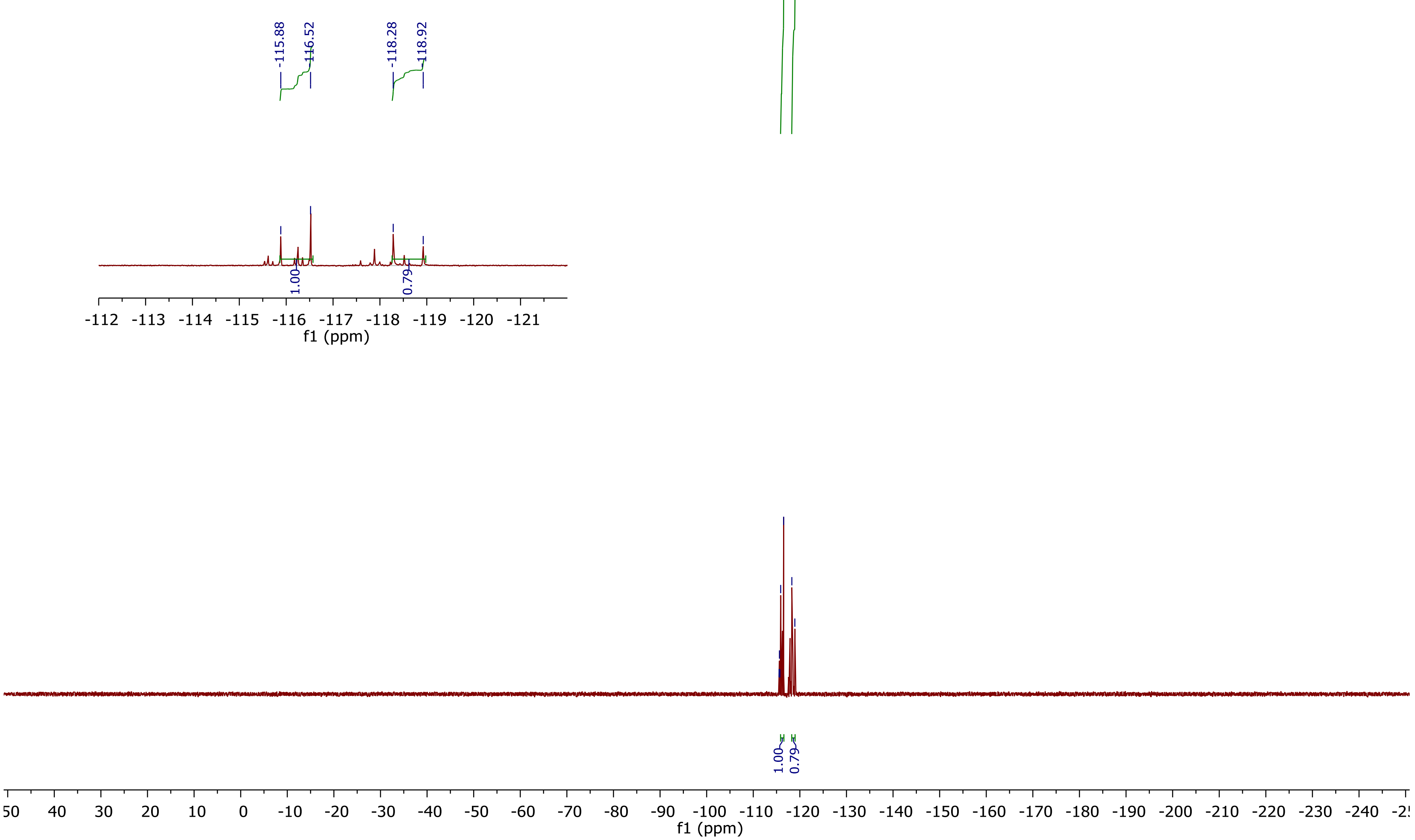

Figure S56 ^{31}P NMR (162 MHz, $CDCl_3$): 3'-*O*-*tert*Butyldimethylsilyl-*N*-4-benzoyl-2'-deoxy-2',2'-difluorocytidine-5'-*O*-[1''-*O*-(2'',3'',4'',6''-tetra-*O*-acetyl-α/β-D-glucopyranose)]-phosphate triethylammonium salt 13

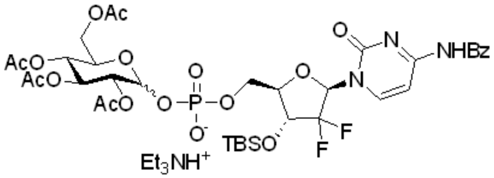

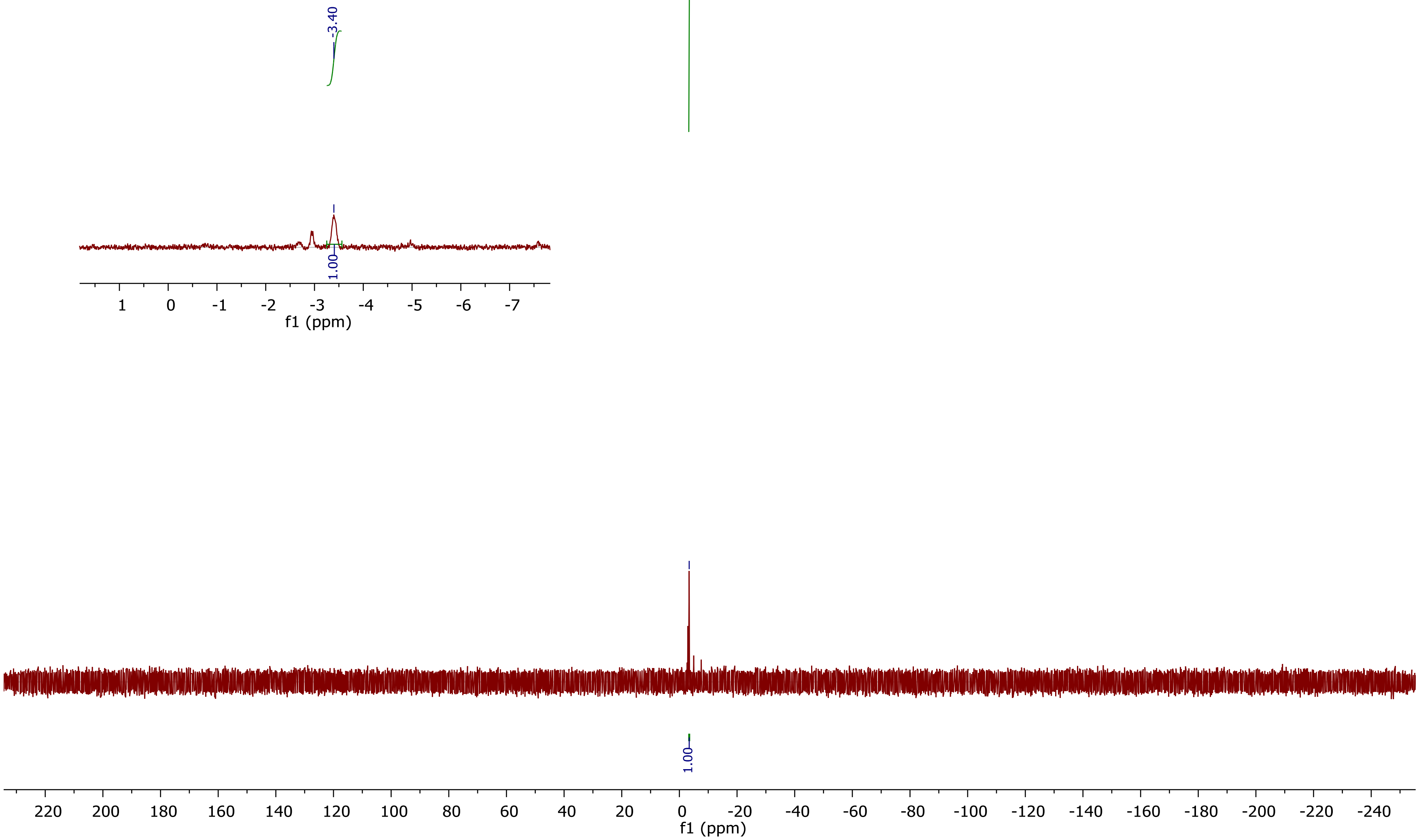

Figure S57 ^{1}H NMR (400 MHz, D_2O): Arabinocytidine-5'-*O*-(1''-*O*-α/β-D-glucopyranose)-phosphate sodium salt 14

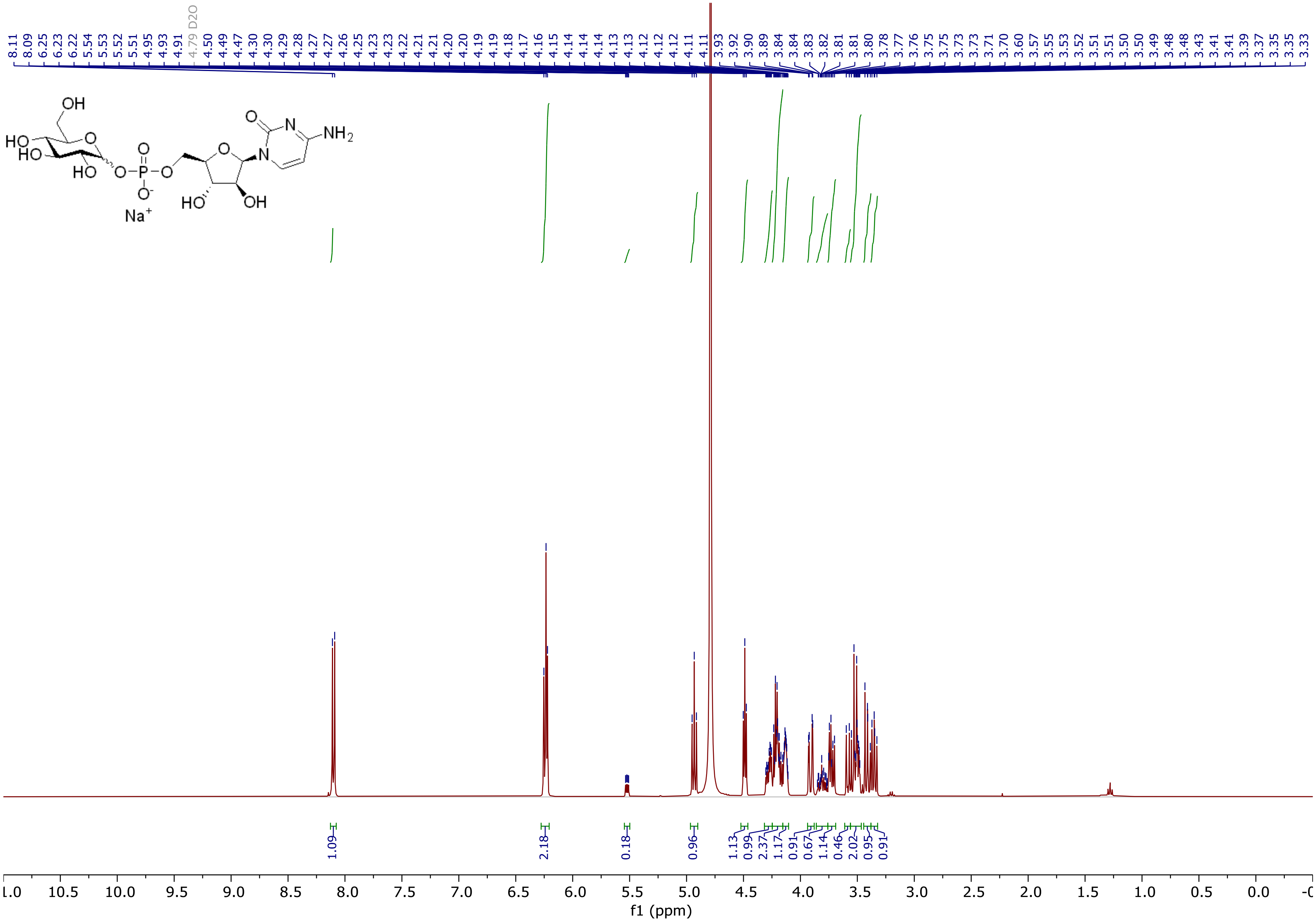

Figure S58 ^{13}C NMR (101 MHz, D_2O): Arabinocytidine-5'-*O*-(1''-*O*-α/β-D-glucopyranose)-phosphate sodium salt 14

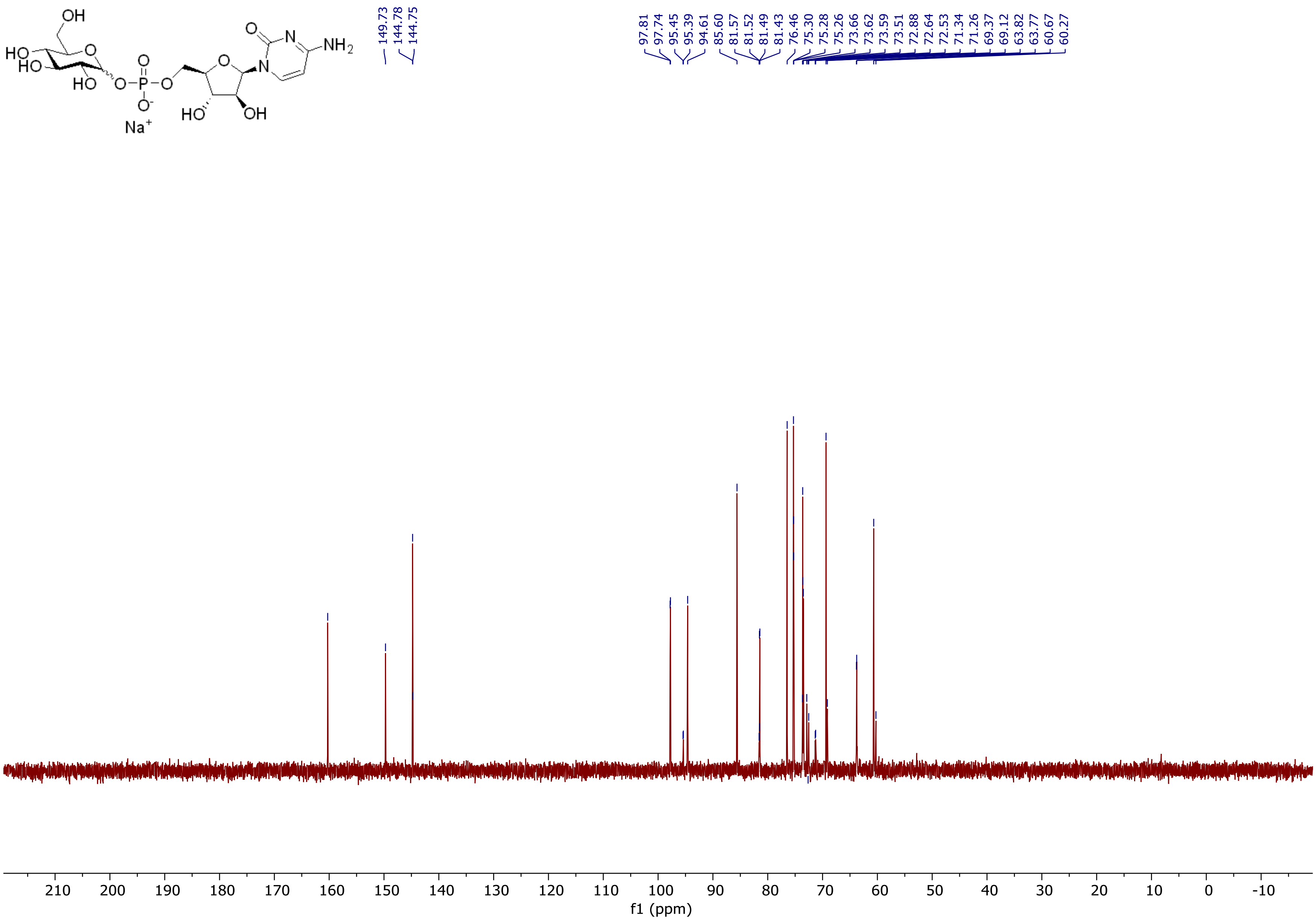

Figure S59 ^{31}P NMR (162 MHz, D_2O): Arabinocytidine-5'-*O*-(1''-*O*-α/β-D-glucopyranose)-phosphate sodium salt 14

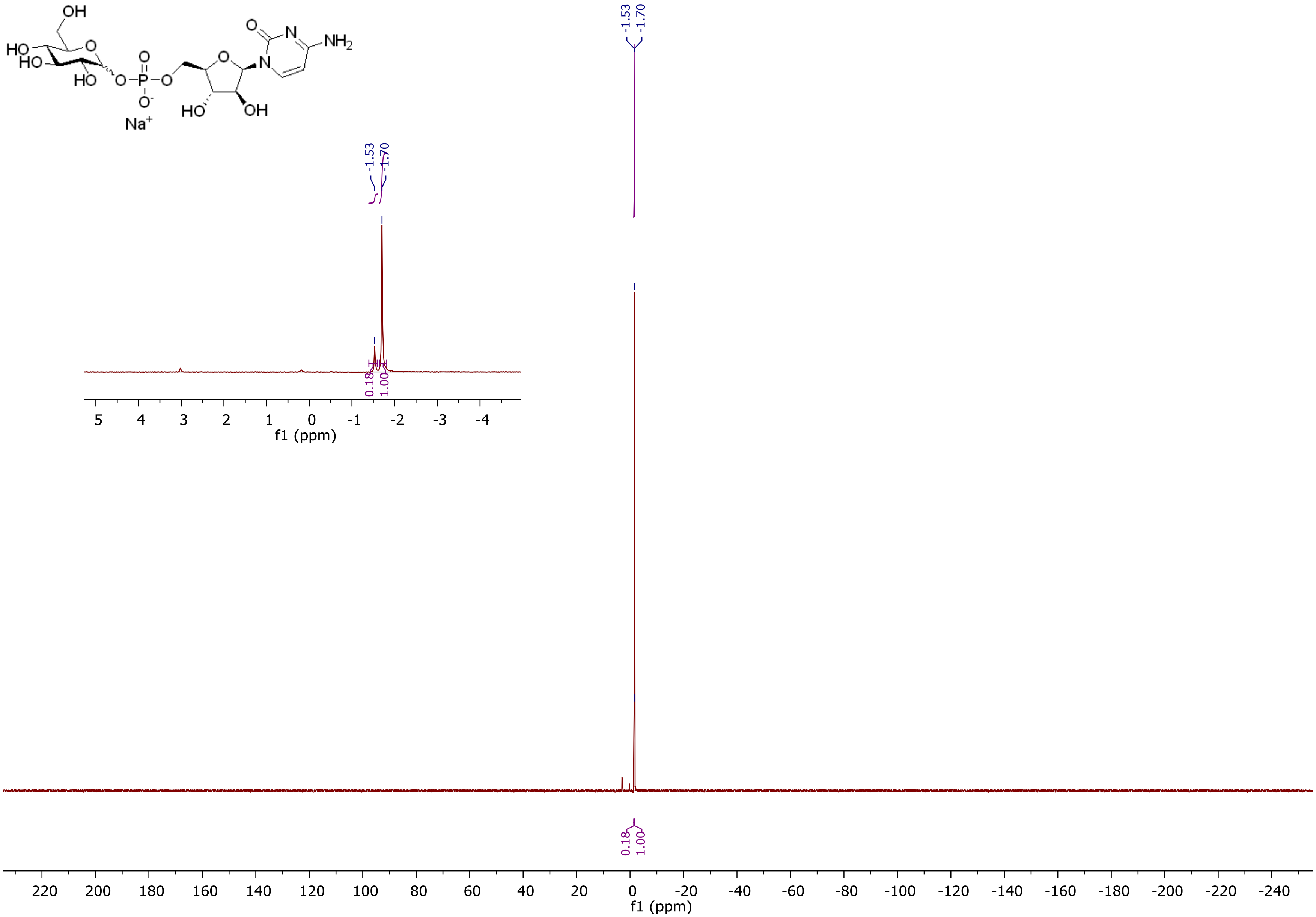

Figure S60 ^{1}H NMR (400 MHz, D_2O): 2'-Deoxy-2',2'-difluorocytidine-5'-*O*-(1''-*O*-α/β-D-glucopyranose)-phosphate sodium salt **15**

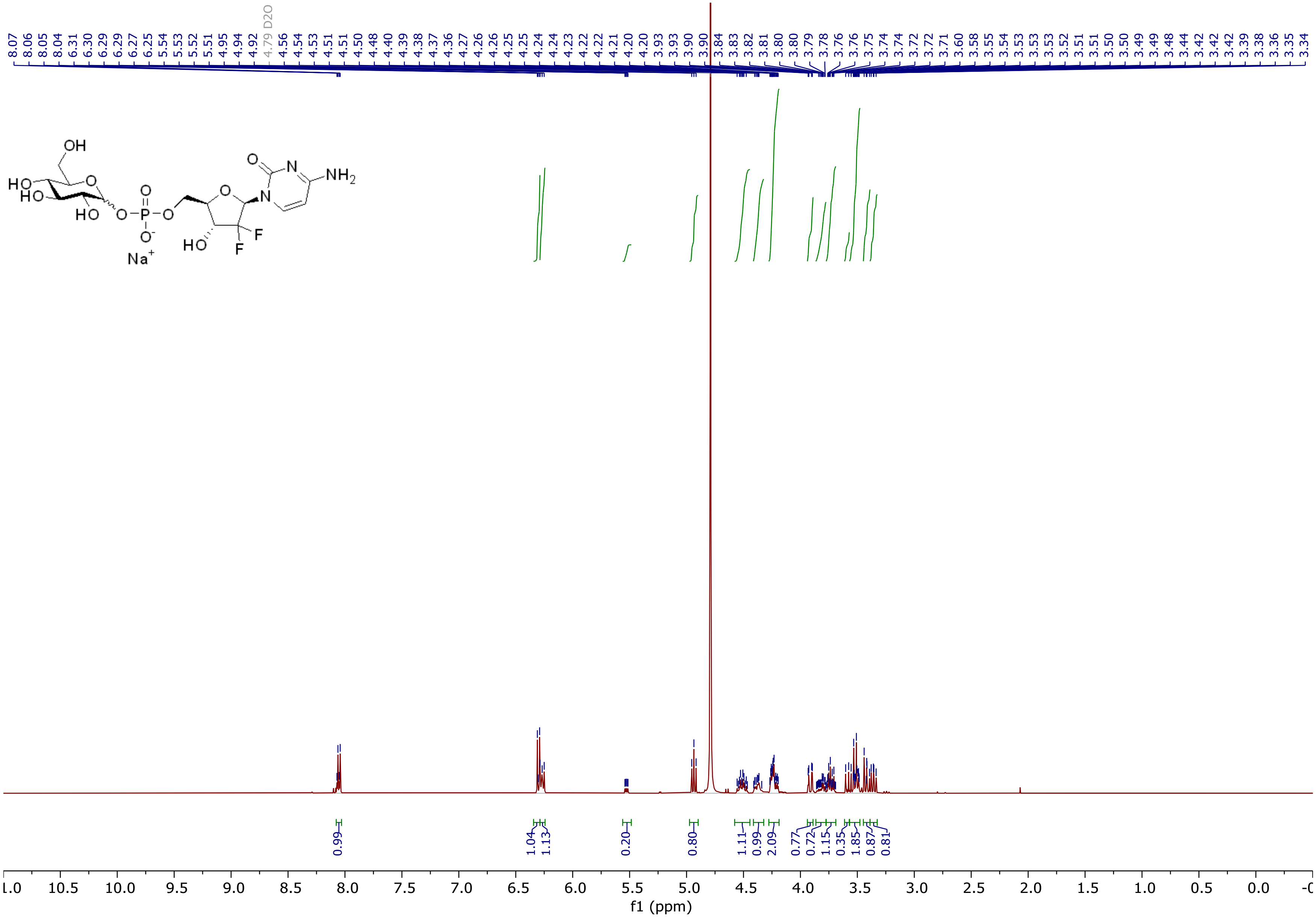

Figure S61 $^{19}F\{^1H\}$ NMR (377 MHz, D_2O): 2'-Deoxy-2',2'-difluorocytidine-5'-*O*-(1''-*O*-α/β-D-glucopyranose)-phosphate sodium salt 15

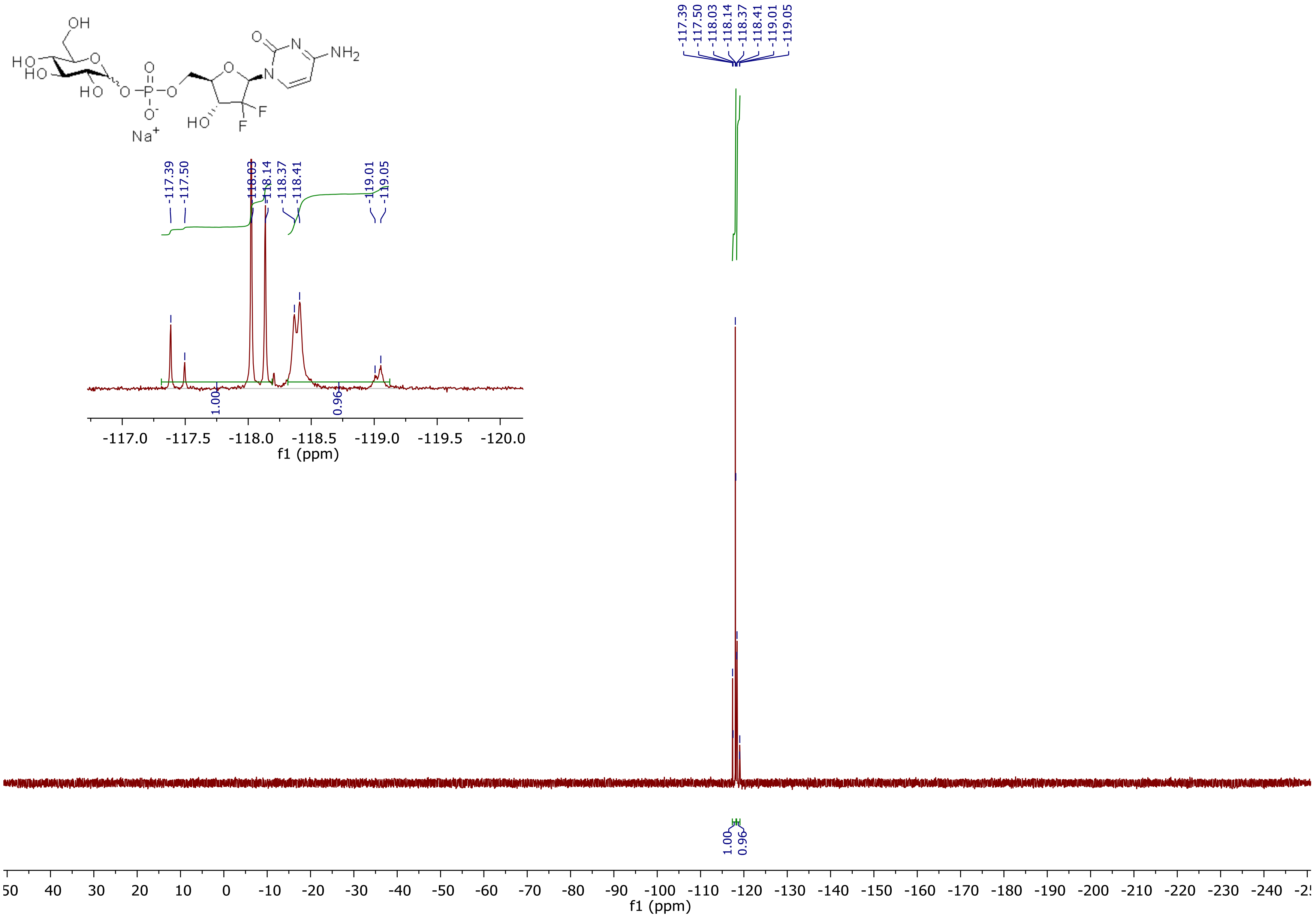

Figure S62 ^{31}P {^{1}H} NMR (162 MHz, D_2O): 2'-Deoxy-2',2'-difluorocytidine-5'-*O*-(1''-*O*-α/β-D-glucopyranose)-phosphate sodium salt 15

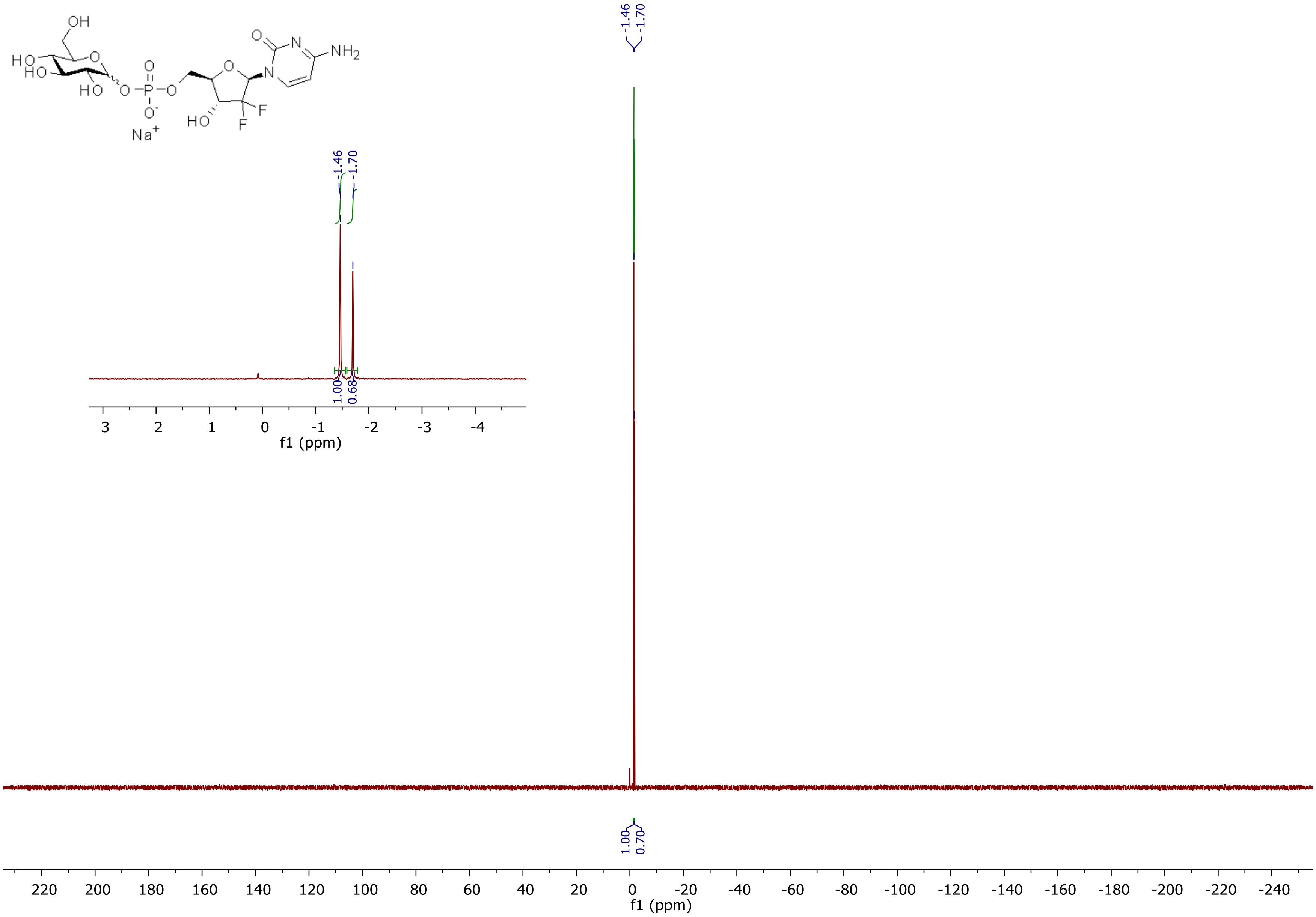

Figure S63 ^{1}H NMR (400 MHz, $CDCl_3$): 1,2,3,4-Tetra-*O*-acetyl-β-D-galctopyranosyl-6-*O*-hydrogenphosphonate triethylammonium salt 17

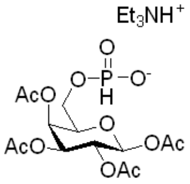

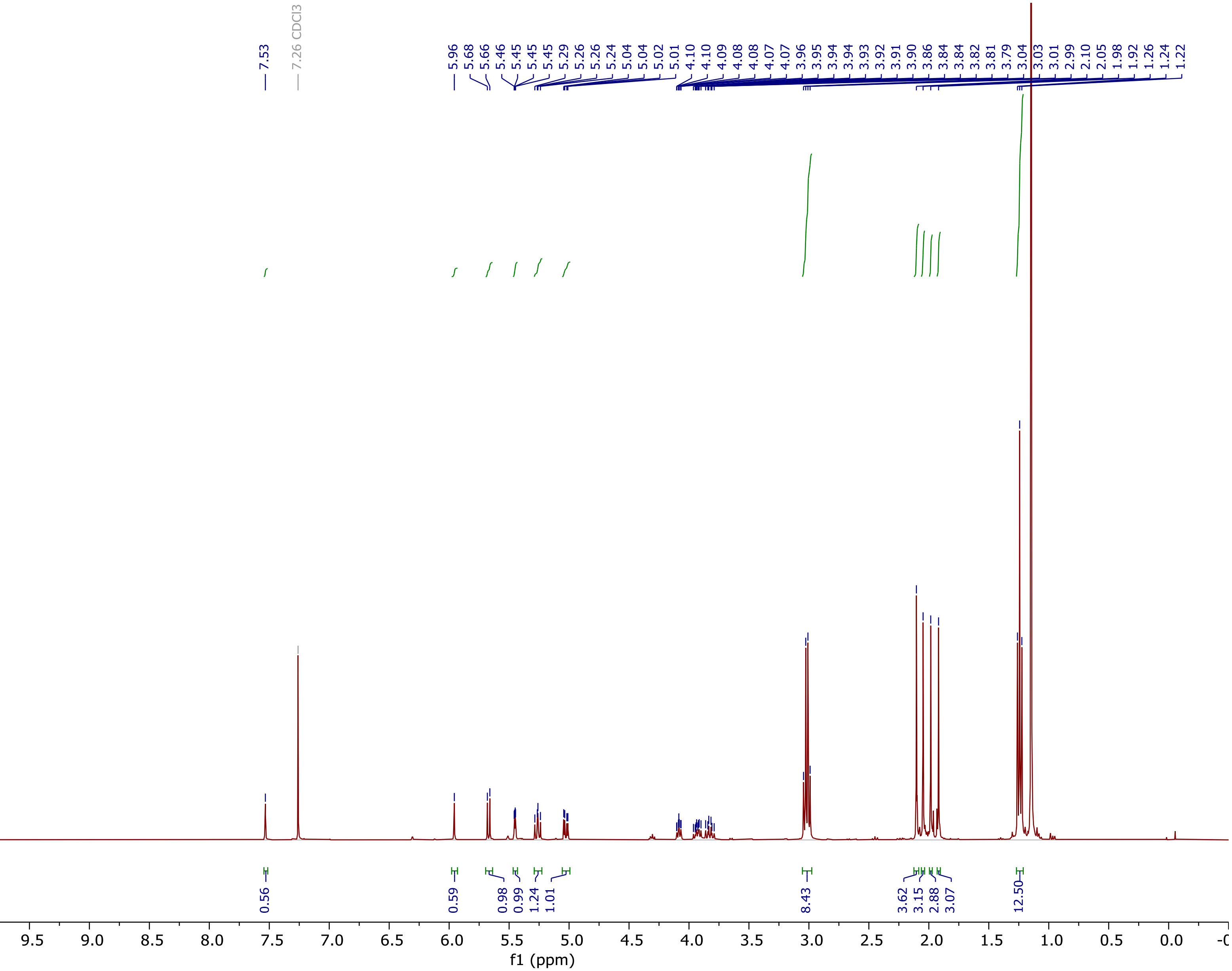

Figure S64 ^{13}C NMR (101 MHz, $CDCl_3$): 1,2,3,4-Tetra-*O*-acetyl-β-D-galctopyranosyl-6-*O*-hydrogenphosphonate triethylammonium salt 17

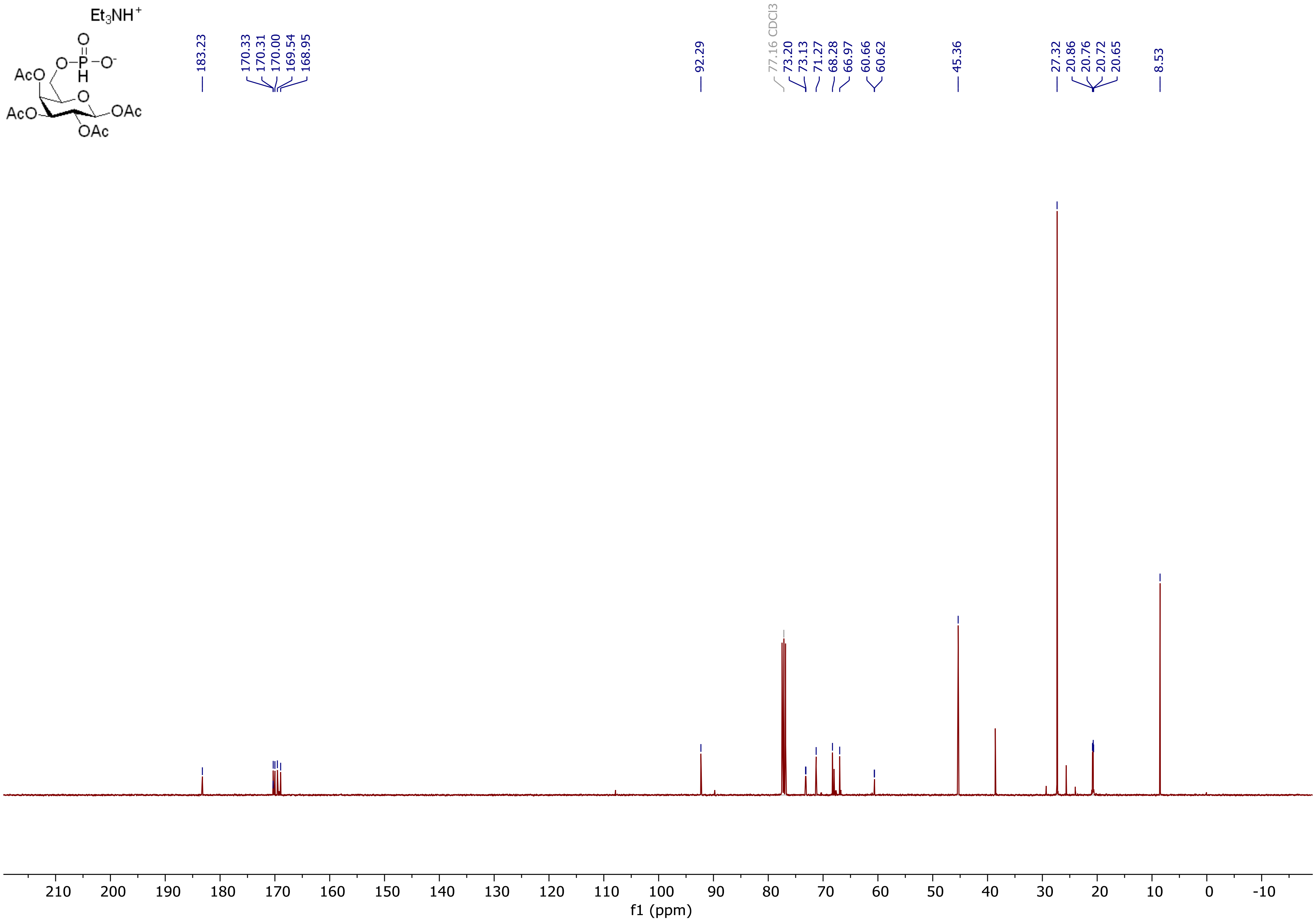

Figure S65 ^{31}P NMR (162 MHz, $CDCl_3$): 1,2,3,4-Tetra-*O*-acetyl-β-D-galctopyranosyl-6-*O*-hydrogenphosphonate triethylammonium salt 17

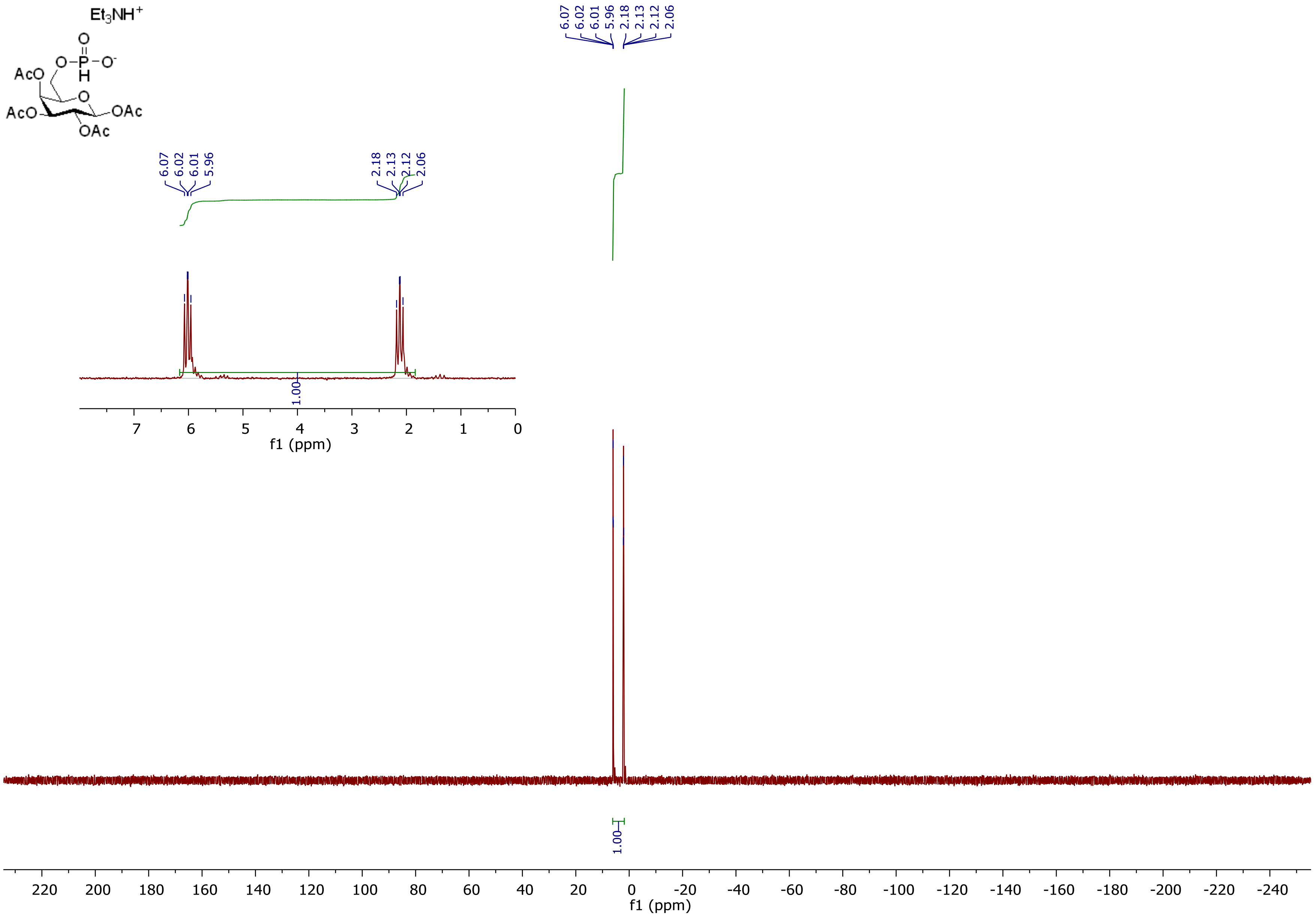

Figure S66 ^{1}H NMR (400 MHz, $CDCl_3$): 2',3'-*O*-*tert*Butyldimethylsilyl-*N*-4-benzoyl-2'-deoxy-2',2'-difluorocytidine-5'-*O*-[6''-*O*-(1'',2'',3'',4''-tetra-*O*-acetyl-β-D-galactopyranose)]-phosphate triethylammonium salt 18

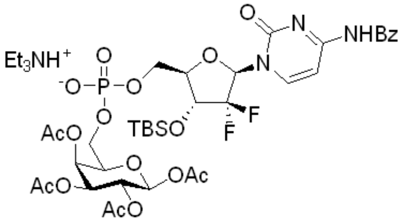

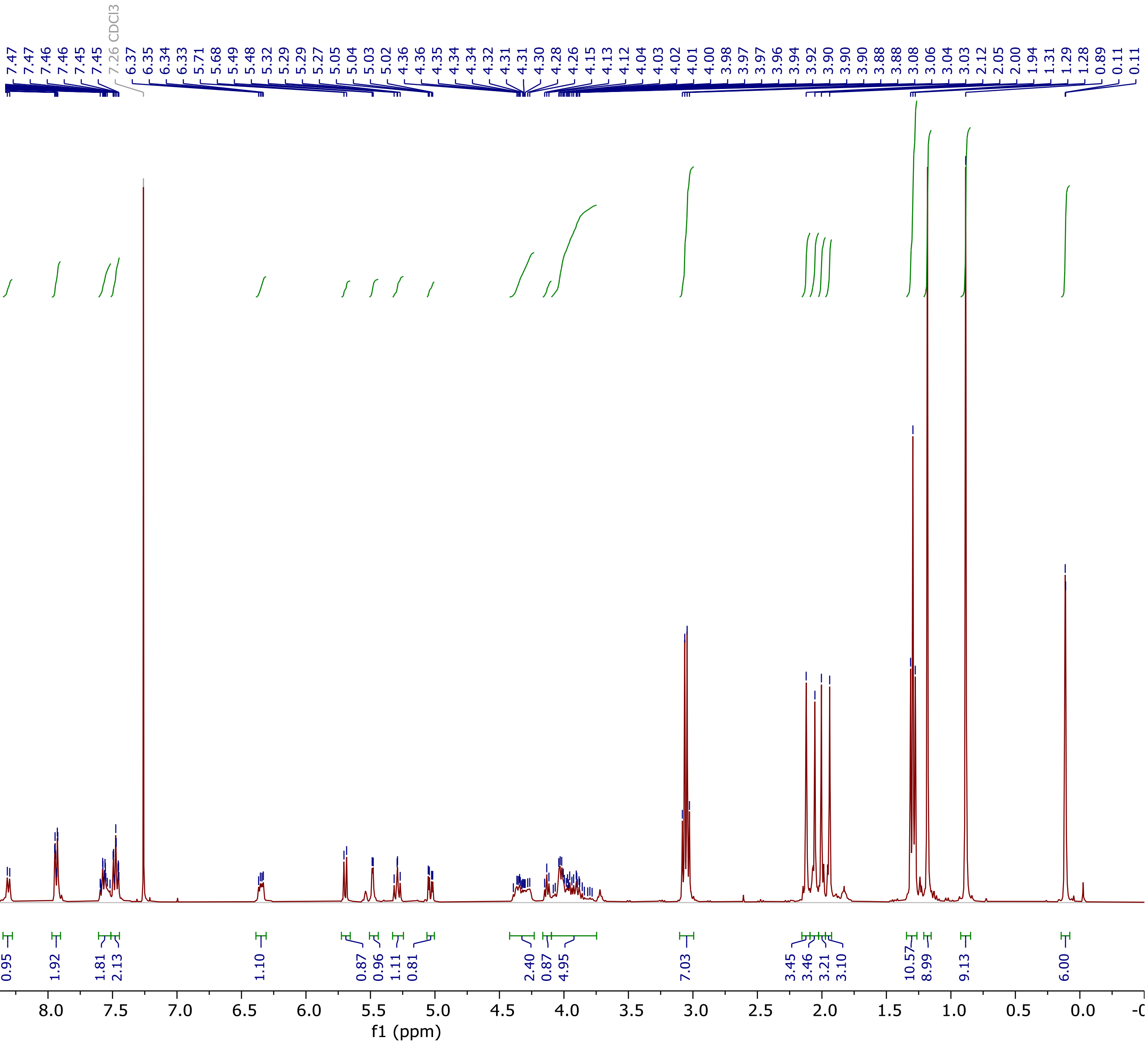

Figure S67 ^{13}C NMR (101 MHz, $CDCl_3$): 2',3'-*O*-*tert*Butyldimethylsilyl-*N*-4-benzoyl-2'-deoxy-2',2'-difluorocytidine-5'-*O*-[6''-*O*-(1'',2'',3'',4''-tetra-*O*-acetyl-β-D-galactopyranose)]-phosphate triethylammonium salt 18.

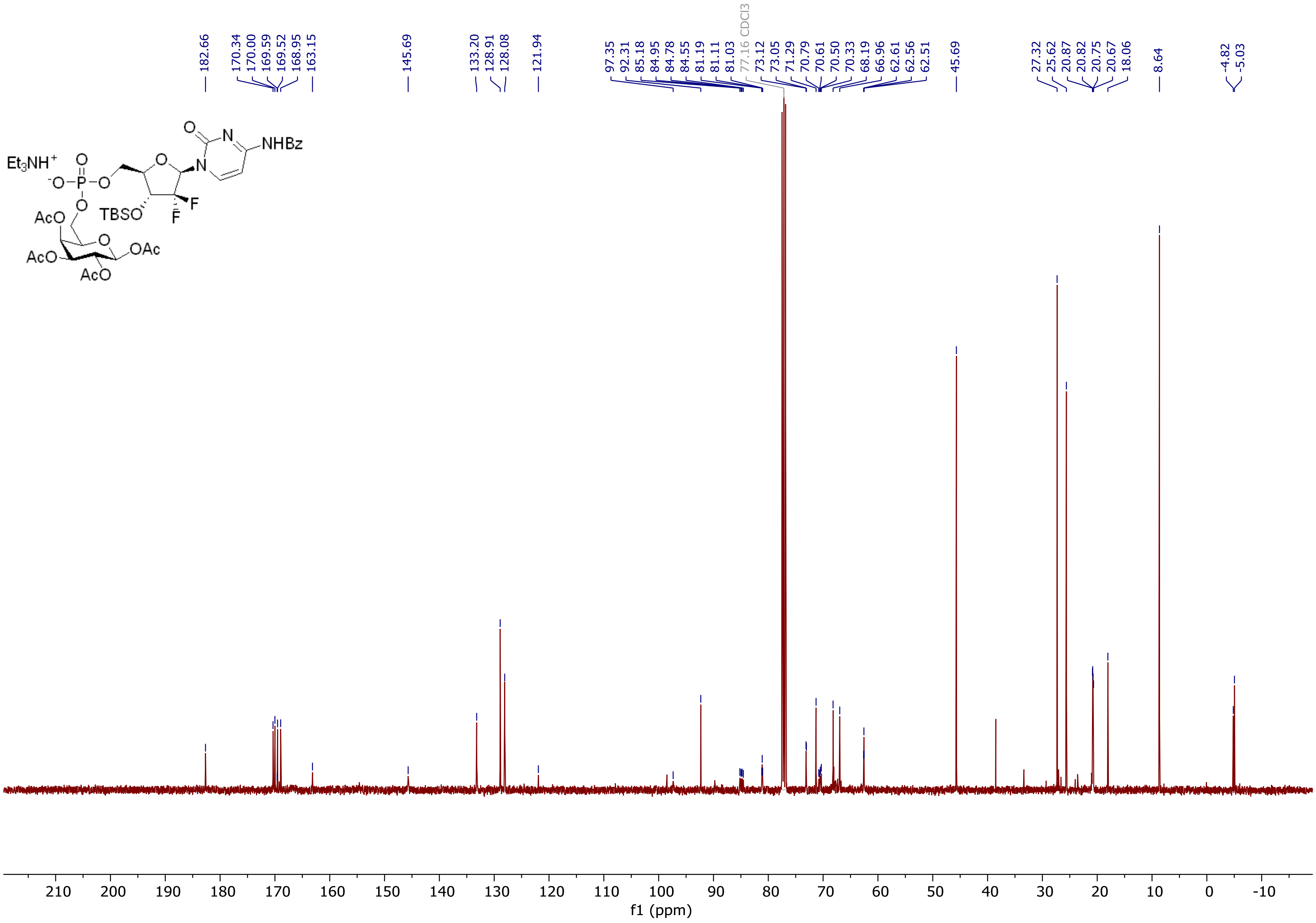

Figure S68 ^{19}F NMR (377 MHz, $CDCl_3$): 2',3'-*O*-*tert*Butyldimethylsilyl-*N*-4-benzoyl-2'-deoxy-2',2'-difluorocytidine-5'-*O*-[6''-*O*-(1'',2'',3'',4''-tetra-*O*-acetyl-β-D-galactopyranose)]-phosphate triethylammonium salt 18

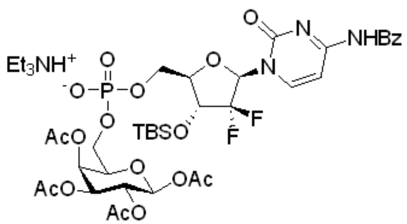

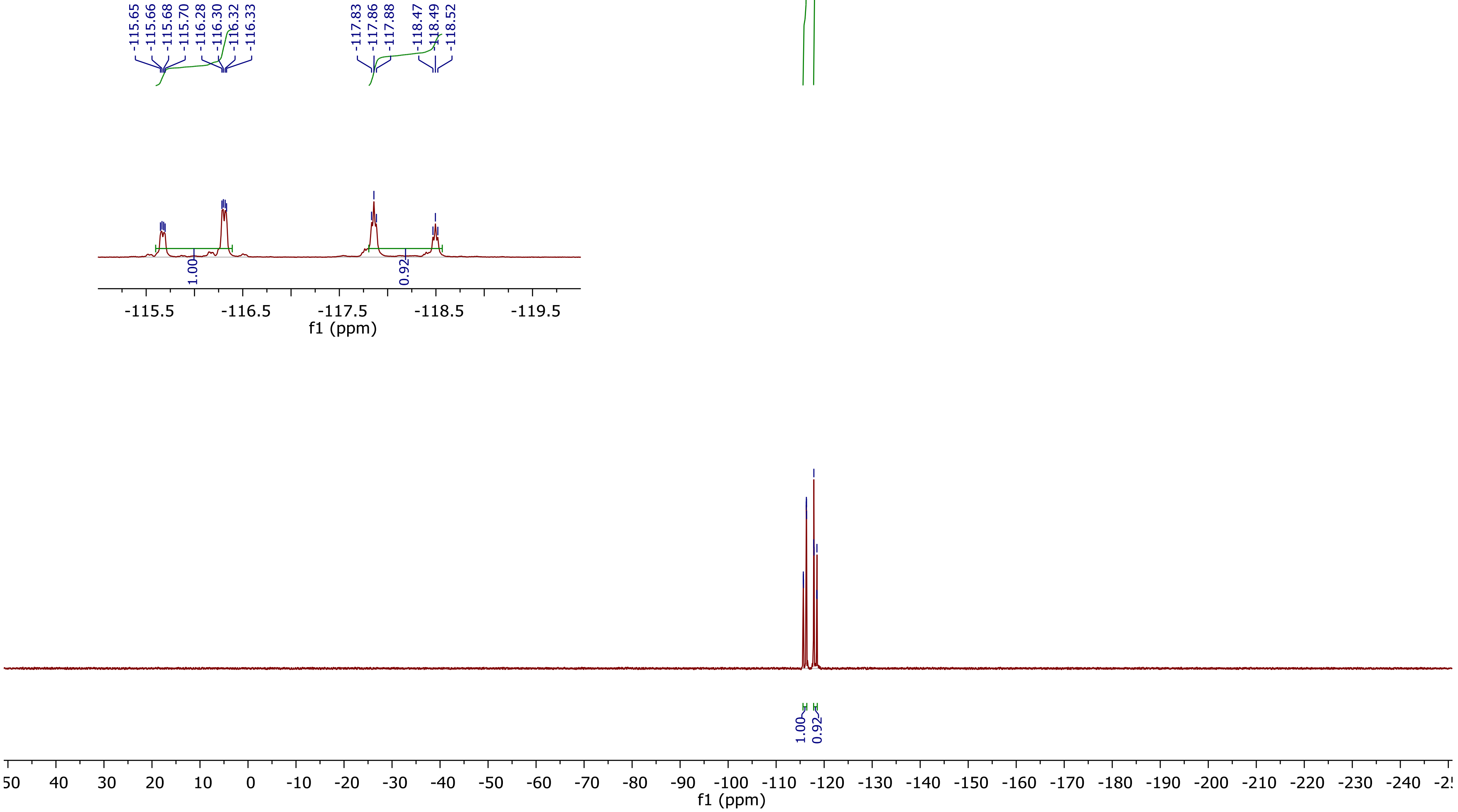

Figure S69 $^{31}P\{^1H\}$ NMR (162 MHz, $CDCl_3$): 2',3'-*O*-*tert*Butyldimethylsilyl-*N*-4-benzoyl-2'-deoxy-2',2'-difluorocytidine-5'-*O*-[6''-*O*-(1'',2'',3'',4''-tetra-*O*-acetyl-β-D-galactopyranose)]-phosphate triethylammonium salt 18

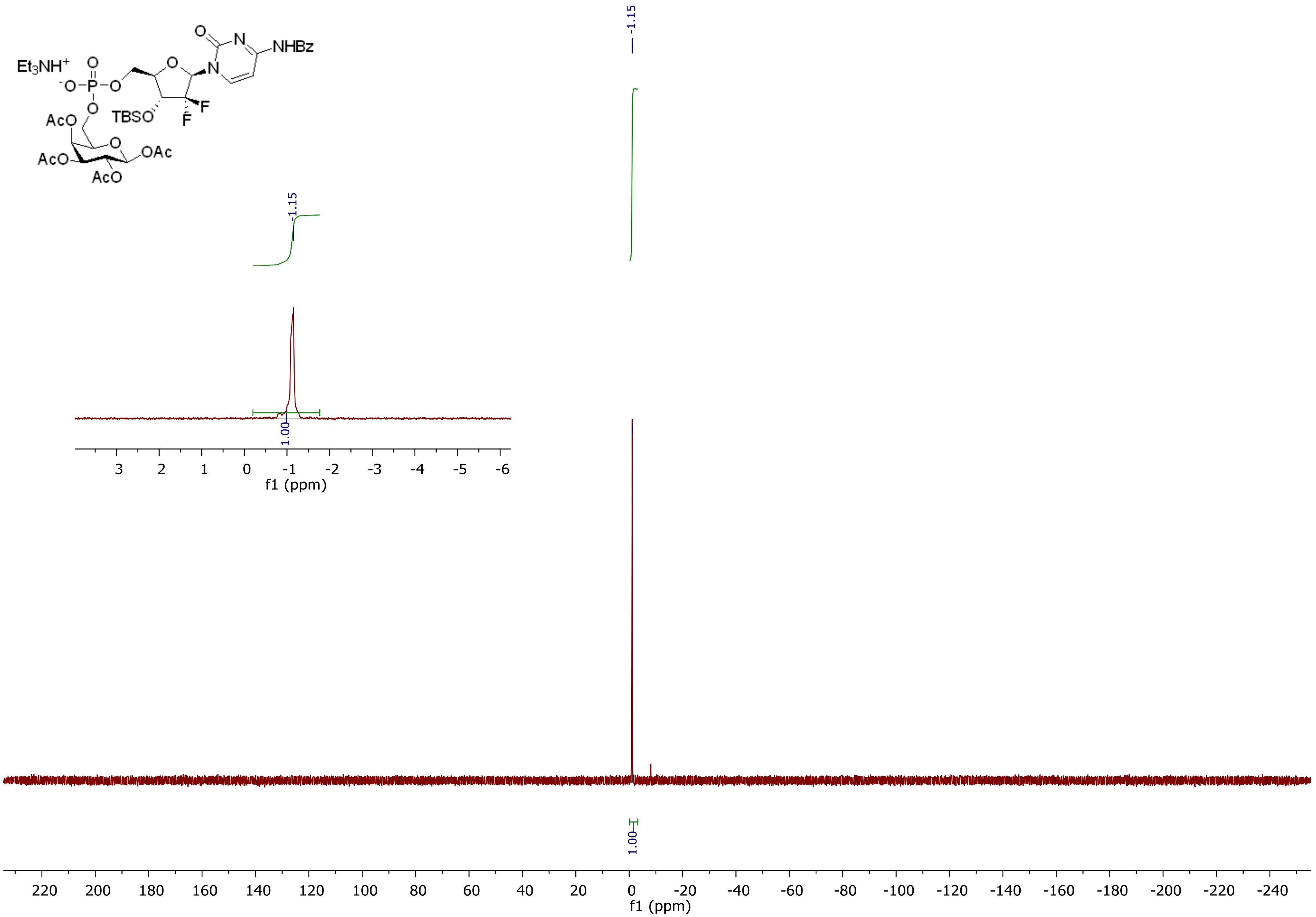

Figure S70 ^{1}H NMR (400 MHz, $CDCl_3$): 2',3'-*O*-*tert*Butyldimethylsilyl-*N*-4-benzoyl-arabinocytidine-5'-*O*-[6''-*O*-(1'',2'',3'',4''-tetra-*O*-acetyl-α/β-D-galactopyranose)]-phosphate triethylammonium salt 19

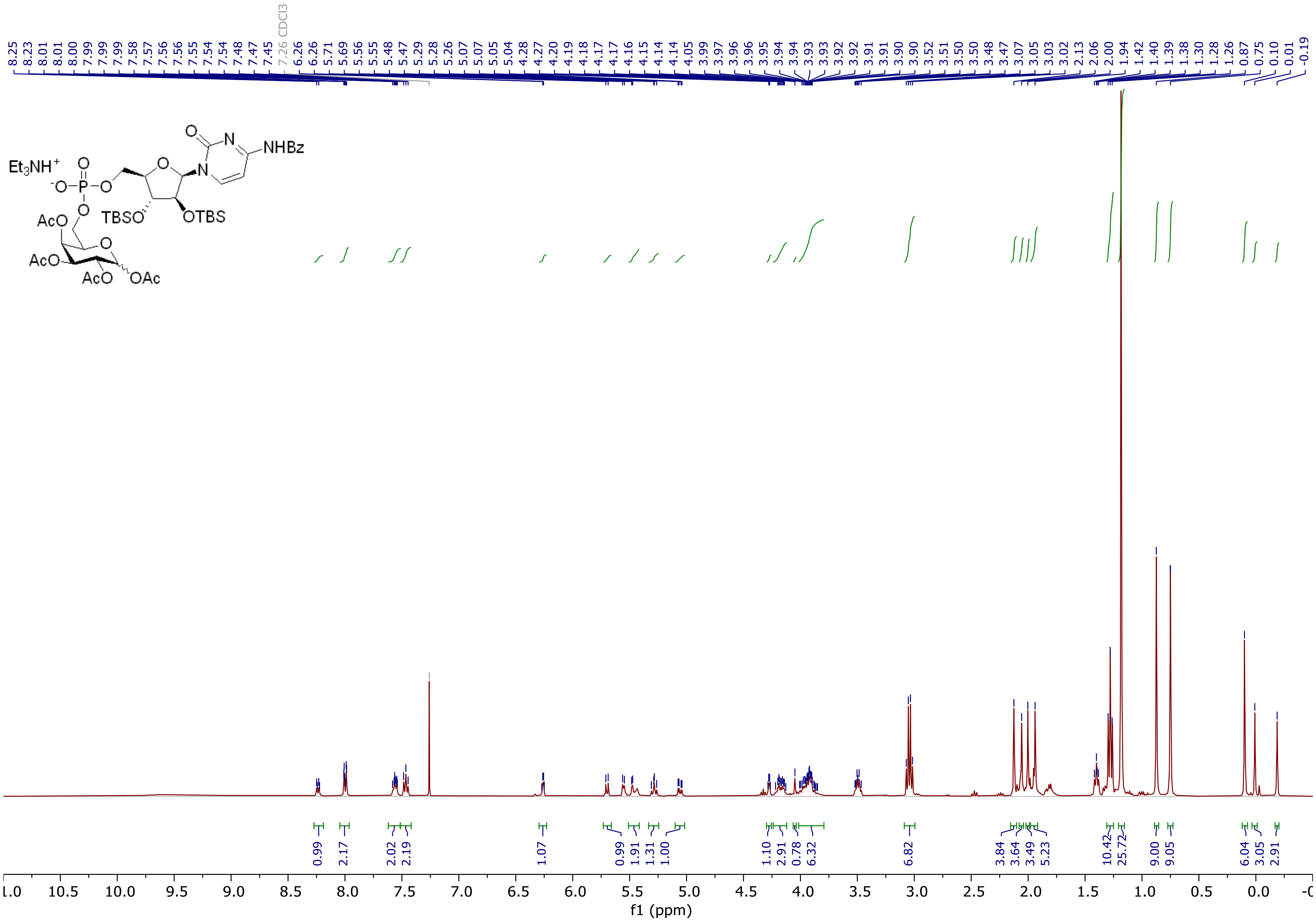

Figure S71 ^{13}C NMR (101 MHz, $CDCl_3$): 2',3'-*O*-*tert*Butyldimethylsilyl-*N*-4-benzoyl-arabinocytidine-5'-*O*-[6''-*O*-(1'',2'',3'',4''-tetra-*O*-acetyl-α/β-D-galactopyranose)]-phosphate triethylammonium salt 19

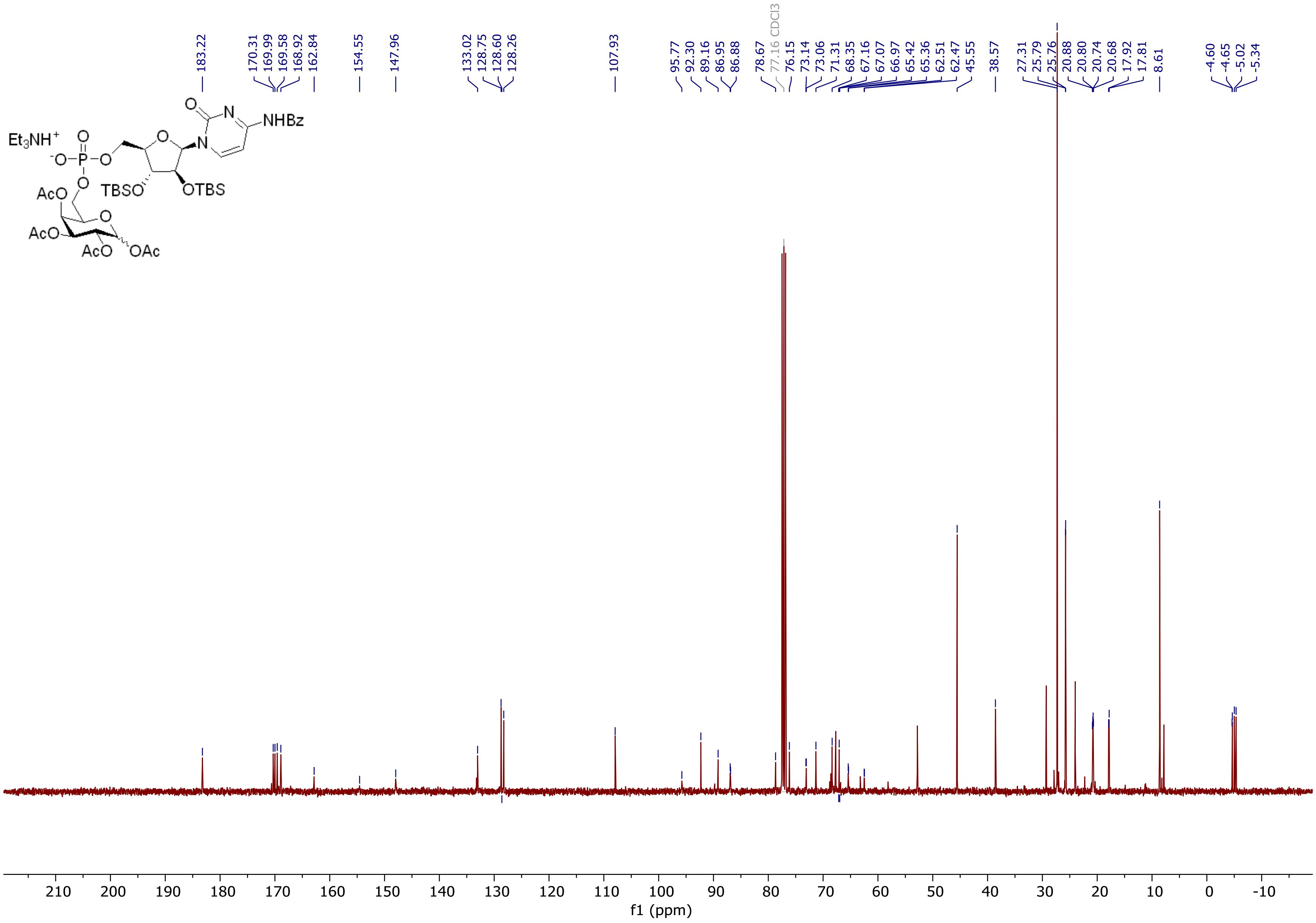

Figure S72 ^{31}P {^{1}H} NMR (162 MHz, CDCl$_3$): 2',3'-*O*-*tert*Butyldimethylsilyl-*N*-4-benzoyl-arabinocytidine-5'-*O*-[6''-*O*-(1'',2'',3'',4''-tetra-*O*-acetyl-α/β-D-galactopyranose)]-phosphate triethylammonium salt 19

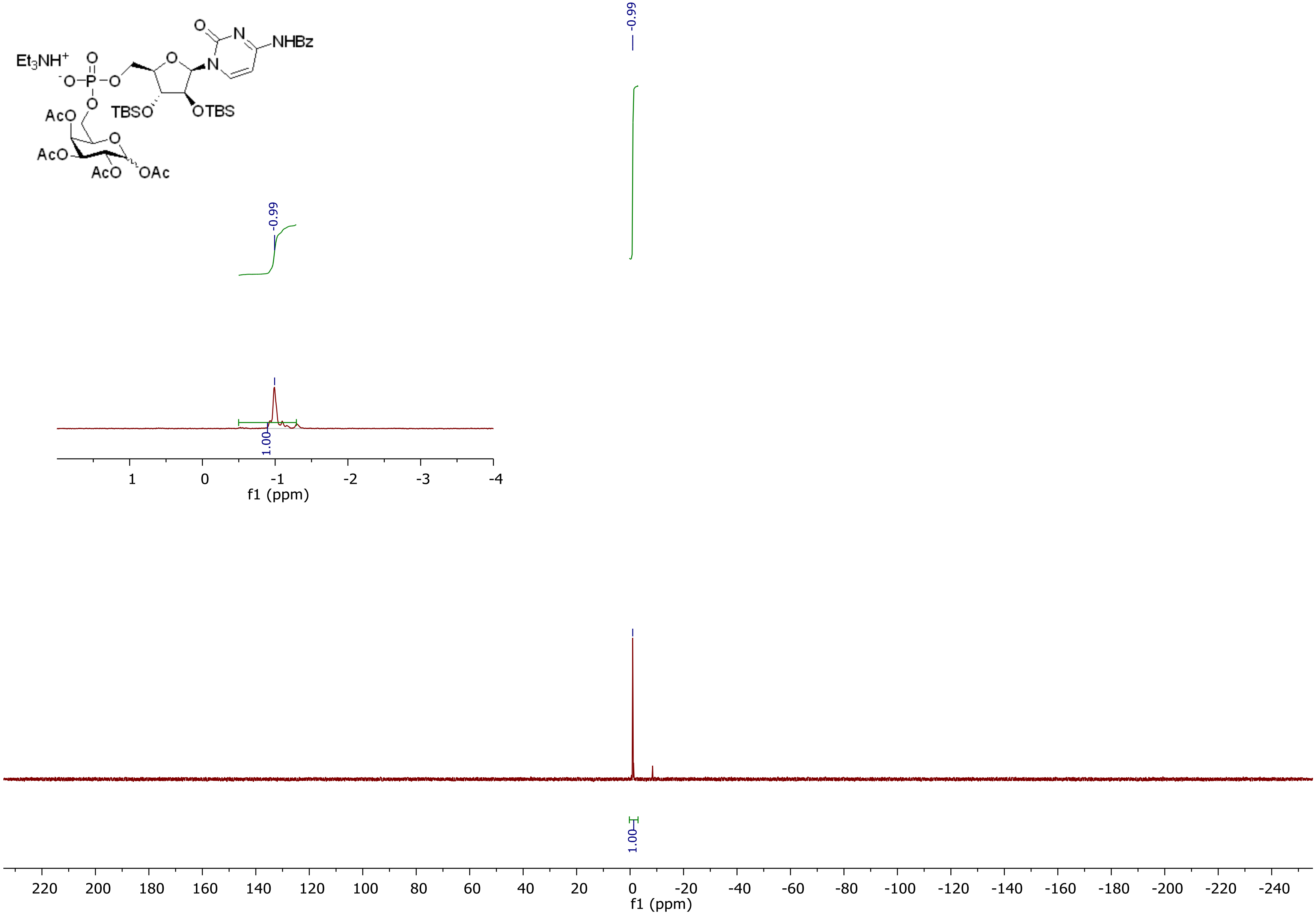

Figure S73 ^{1}H NMR (400 MHz, D_2O): 2’-Deoxy-2’,2’-difluorocytidine-5'-*O*-(6''-*O*-α/β-D-galactopyranose)-phosphate sodium salt **20**

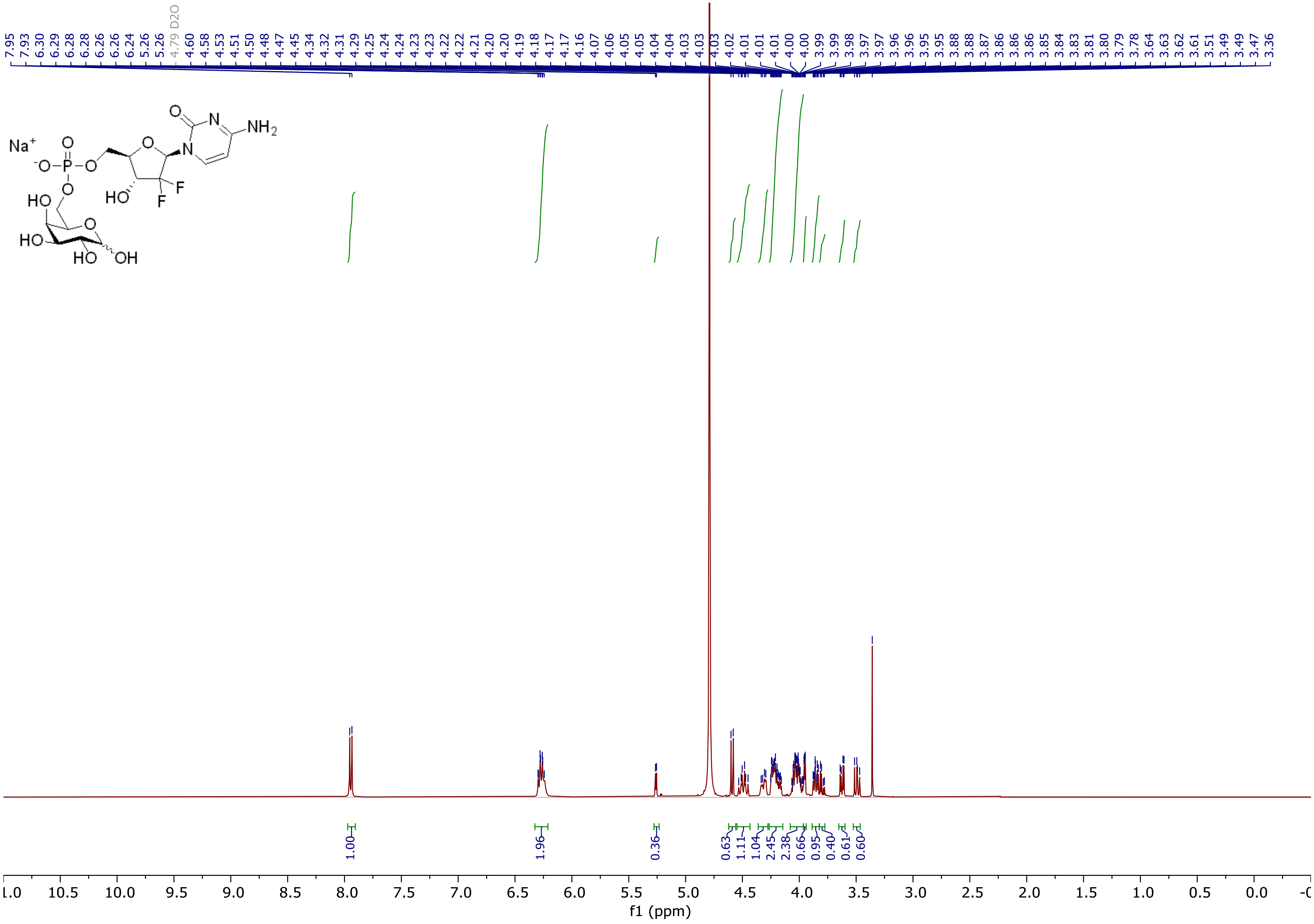

Figure S74 ^{13}C NMR (101 MHz, D_2O): 2’-Deoxy-2’,2’-difluorocytidine-5'-*O*-(6''-*O*-α/β-D-galactopyranose)-phosphate sodium salt 20

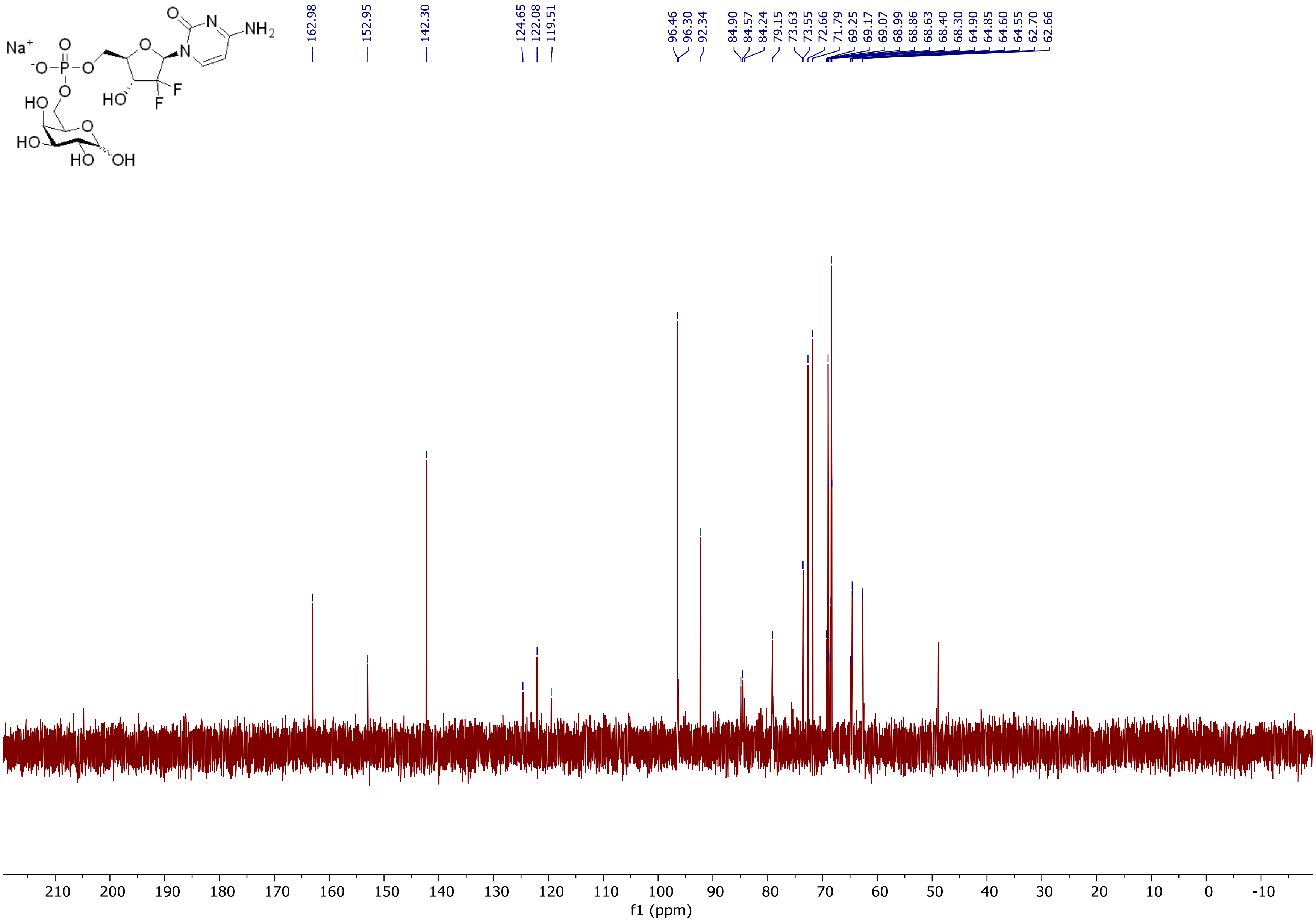

Figure S75 ^{19}F NMR (377 MHz, D_2O): 2’-Deoxy-2’,2’-difluorocytidine-5'-*O*-(6''-*O*-α/β-D-galactopyranose)-phosphate sodium salt **20**

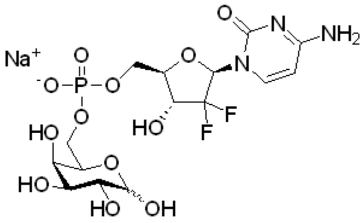

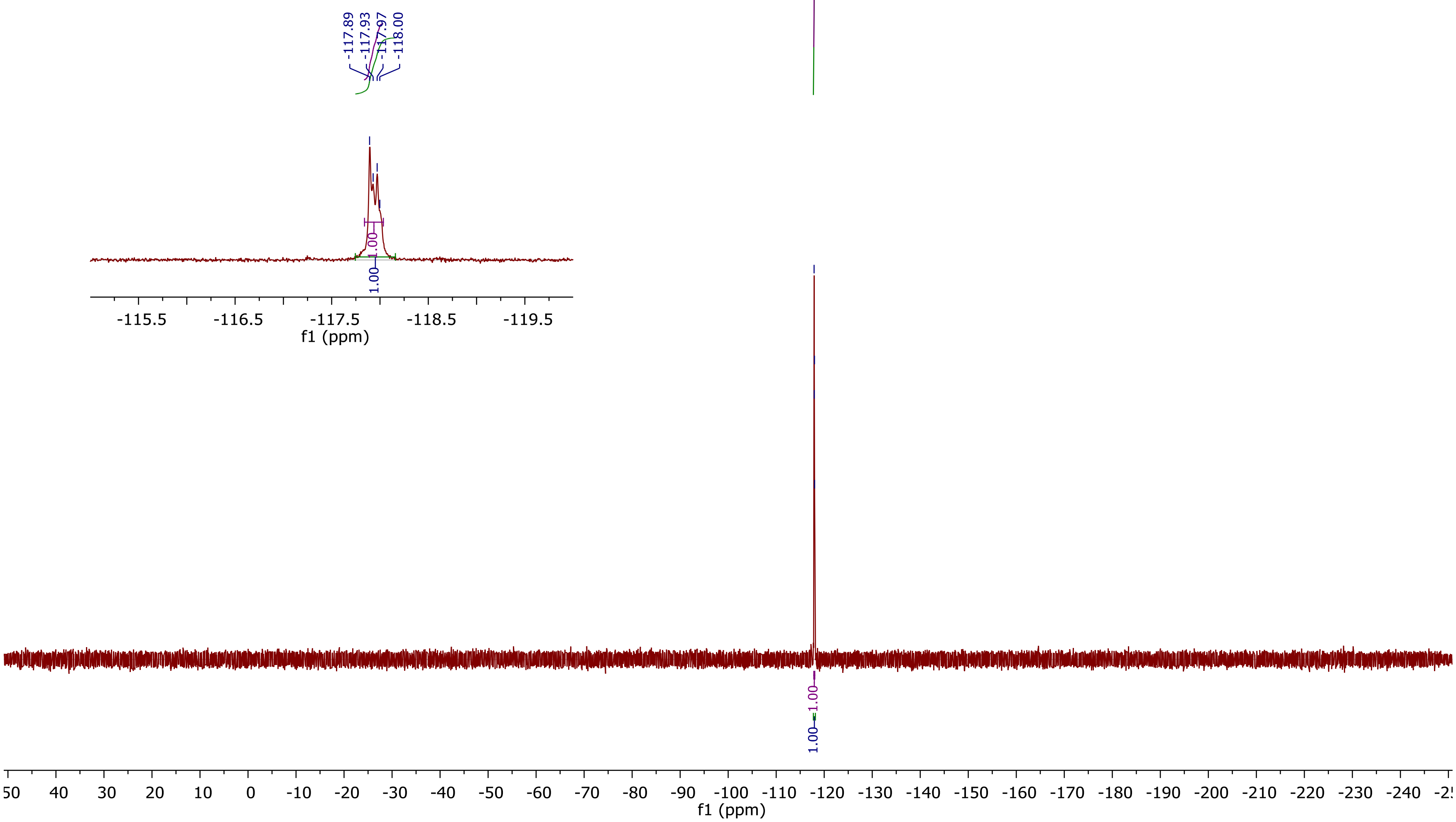

Figure S76 ^{31}P {1H} NMR (162 MHz, D_2O): 2’-Deoxy-2’,2’-difluorocytidine-5'-*O*-(6''-*O*-α/β-D-galactopyranose)-phosphate sodium salt 20

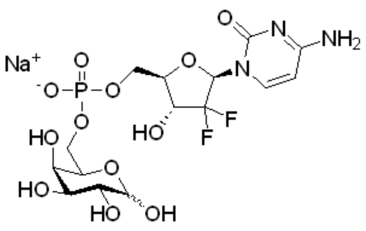

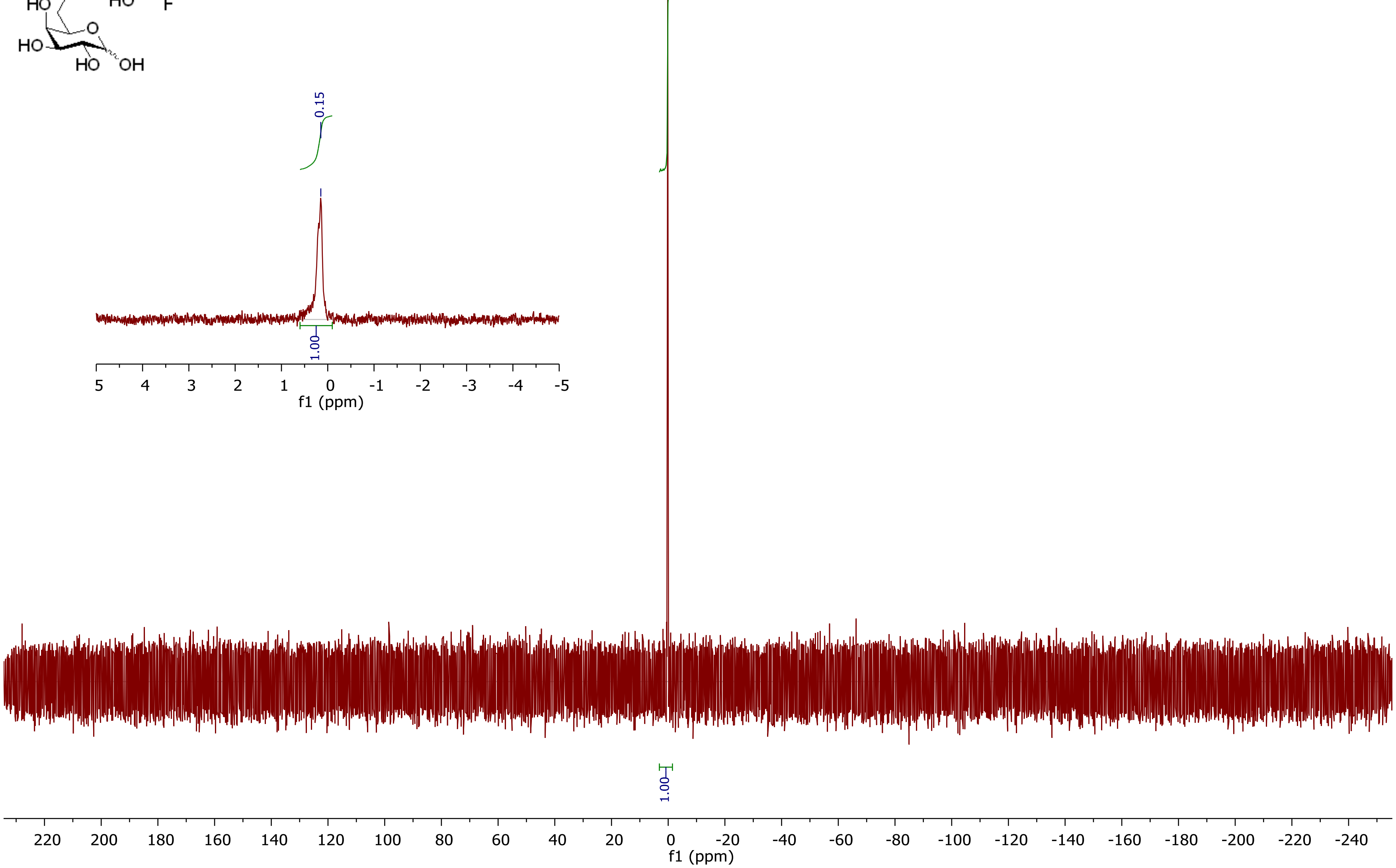

Figure S77 ^{1}H NMR (400 MHz, CDCl$_3$): 2,3,4,6-Tetra-*O*-acetyl-α/β-D-galactopyranosyl-1-*O*-hydrogenphosphonate triethylammonium salt 22

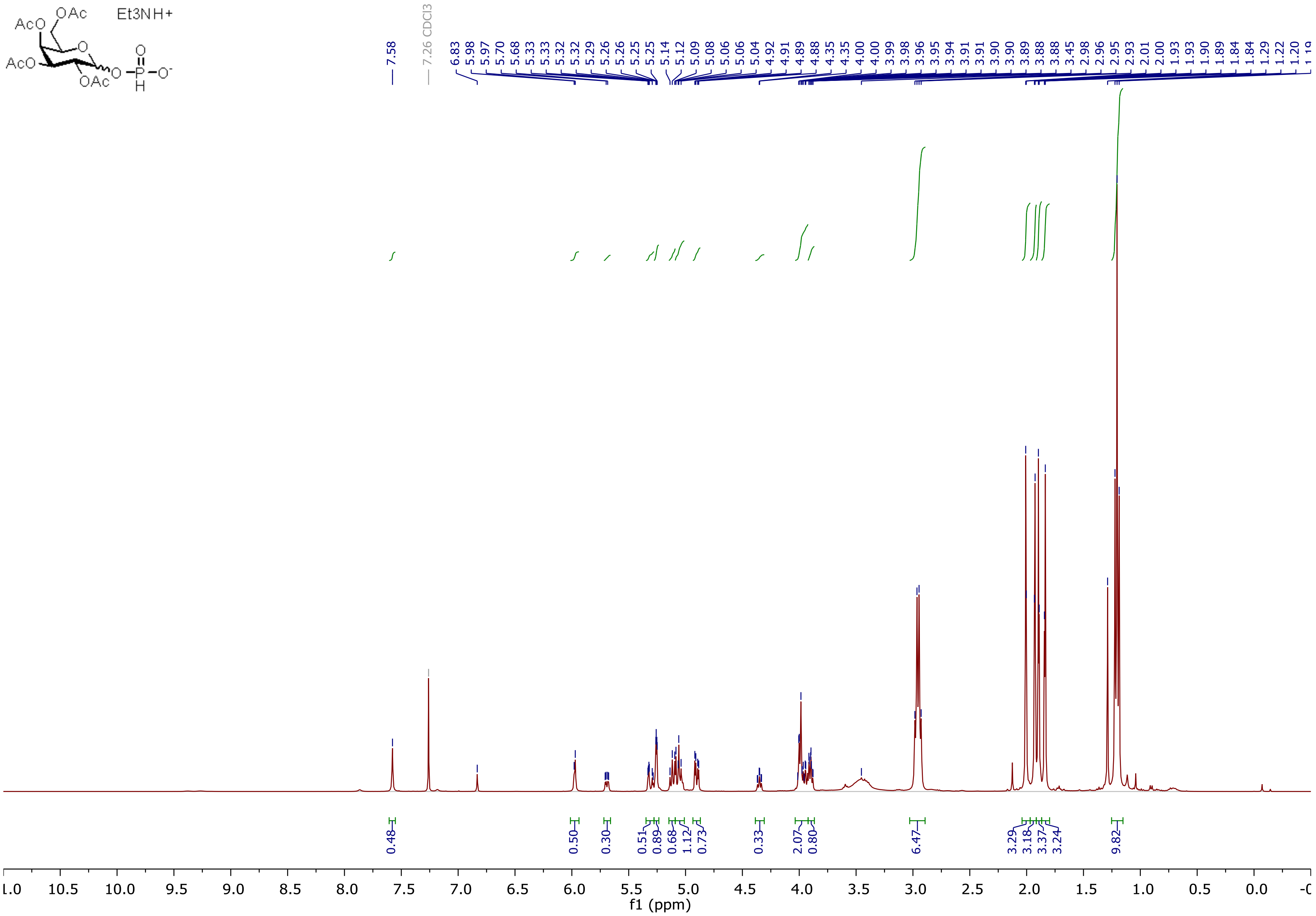

Figure S78 ^{31}P NMR (162 MHz, $CDCl_3$): 2,3,4,6-Tetra-*O*-acetyl-α/β-D-galactopyranosyl-1-*O*-hydrogenphosphonate triethylammonium salt 22

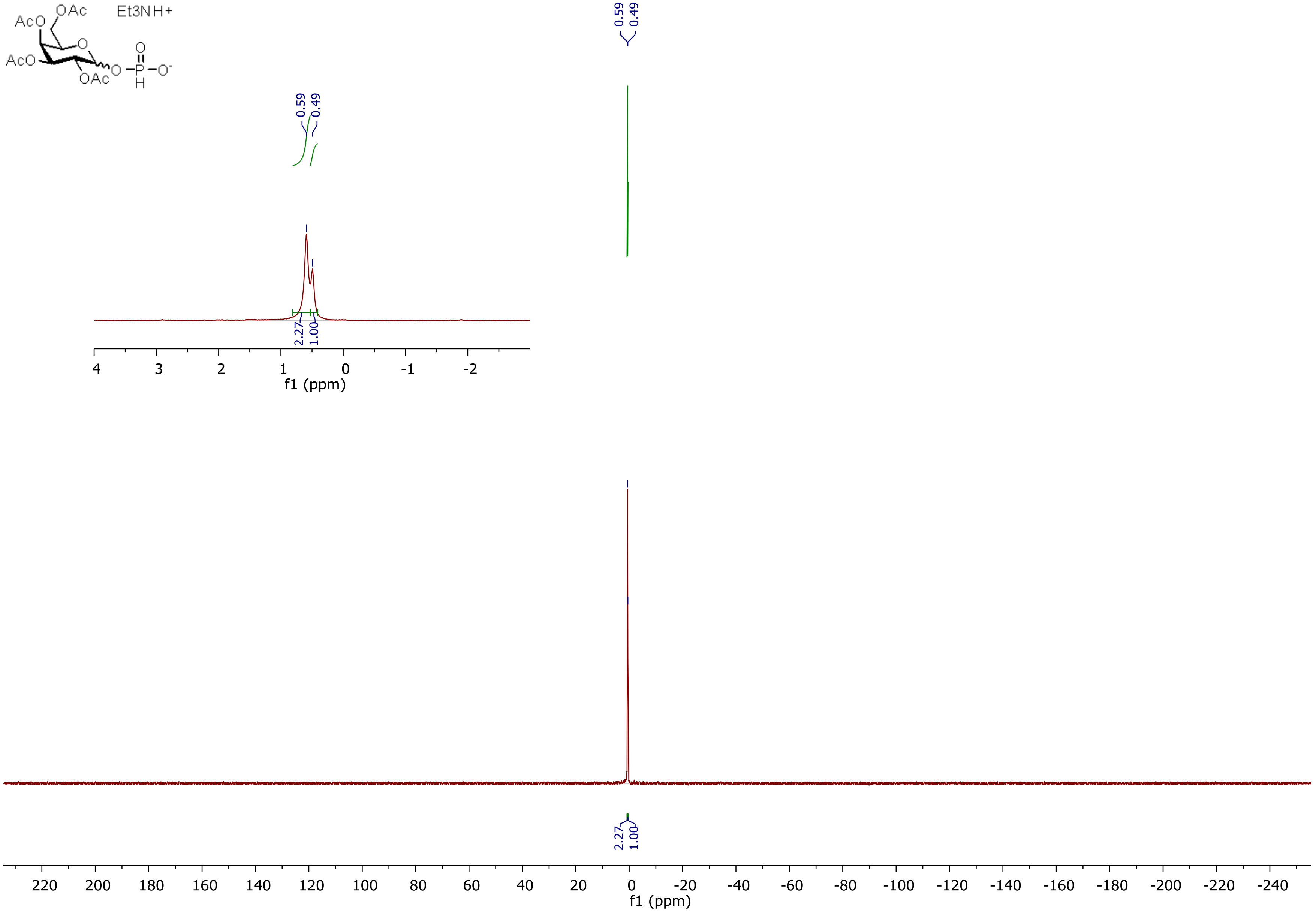

Figure S79 ^1H NMR (400 MHz, $CDCl_3$): 3'-*O*-*tert*Butyldimethylsilyl-*N*-4-benzoyl-2'-deoxy-2',2'-difluorocytidine-5'-*O*-[1''-*O*-(2'',3'',4'',6''-tetra-*O*-acetyl-α/β-D-galactopyranose)]-phosphate triethylammonium salt 23

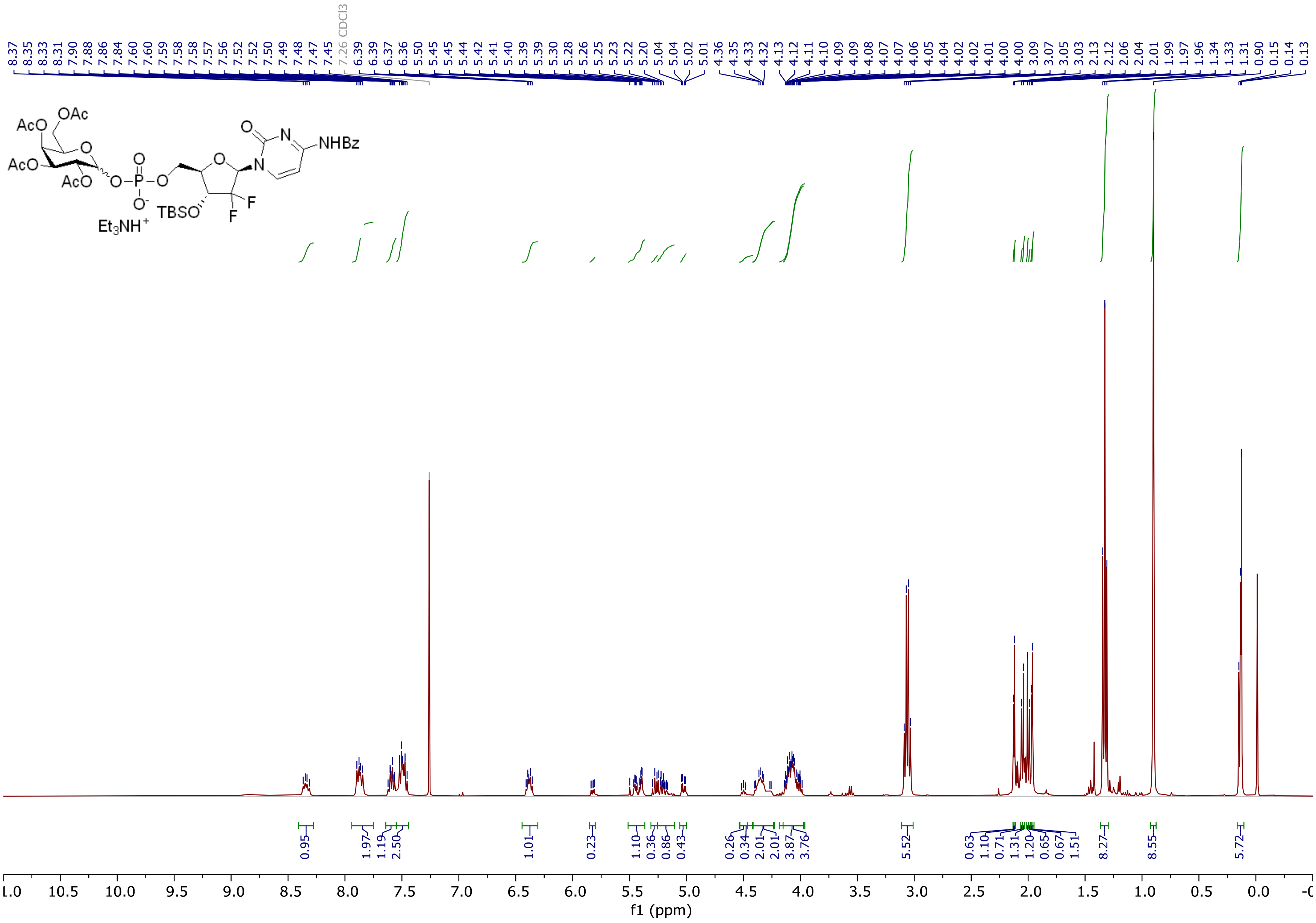

Figure S80 ^{13}C NMR (101 MHz, $CDCl_3$): 3'-*O*-*tert*Butyldimethylsilyl-*N*-4-benzoyl-2'-deoxy-2',2'-difluorocytidine-5'-*O*-[1''-*O*-(2'',3'',4'',6''-tetra-*O*-acetyl-α/β-D-galactopyranose)]-phosphate triethylammonium salt 23

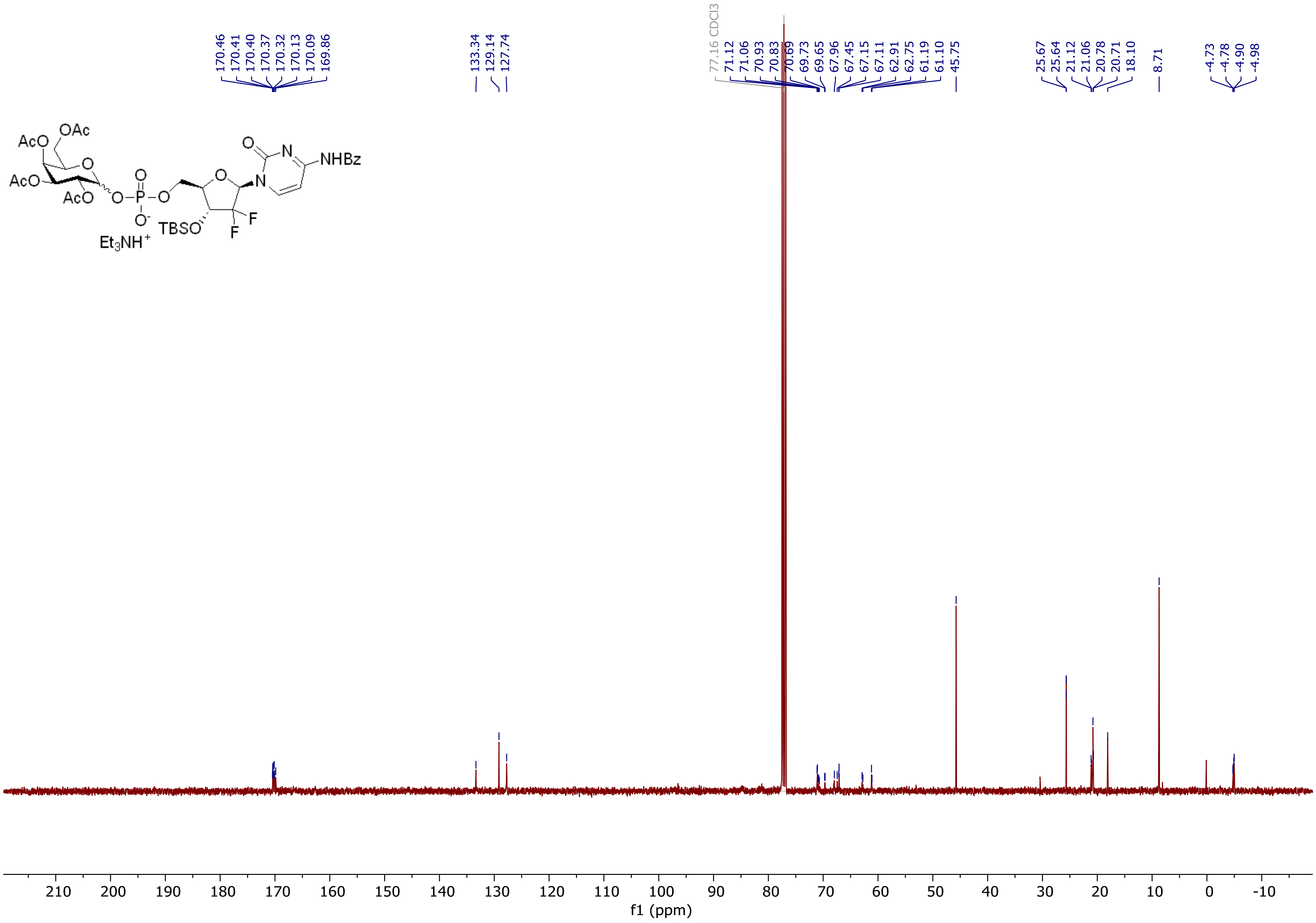

Figure S81 $^{19}F\{^{1}H\}$ NMR (377 MHz, $CDCl_3$): 3'-*O*-*tert*Butyldimethylsilyl-*N*-4-benzoyl-2’-deoxy-2’,2’-difluorocytidine-5'-*O*-[1''-*O*-(2'',3'',4'',6''-tetra-*O*-acetyl-α/β-D-galactopyranose)]-phosphate triethylammonium salt 23

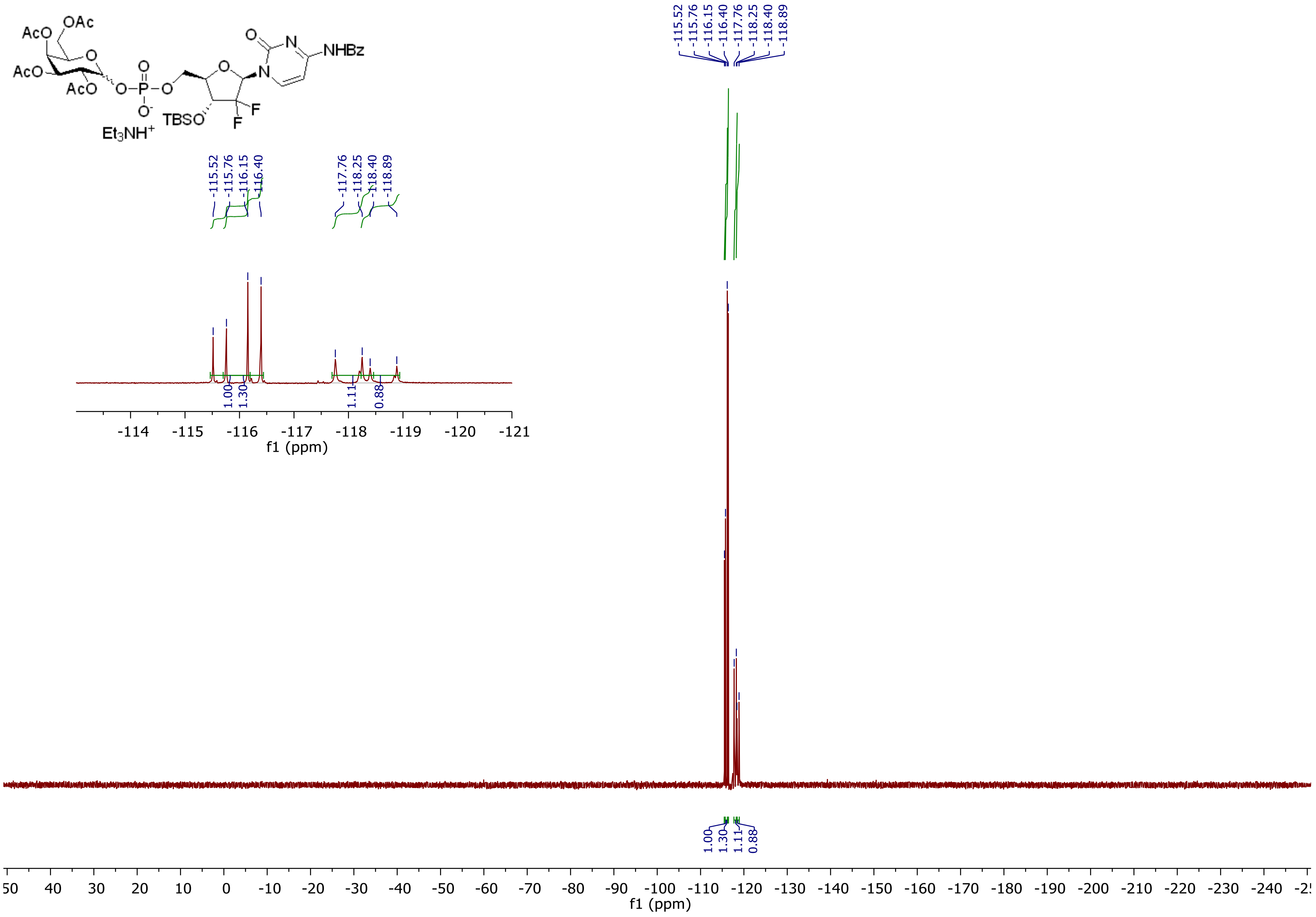

Figure S82 ^{31}P {^{1}H} NMR (162 MHz, $CDCl_3$): 3'-*O*-*tert*Butyldimethylsilyl-*N*-4-benzoyl-2'-deoxy-2',2'-difluorocytidine-5'-*O*-[1''-*O*-(2'',3'',4'',6''-tetra-*O*-acetyl-α/β-D-galactopyranose)]-phosphate triethylammonium salt 23

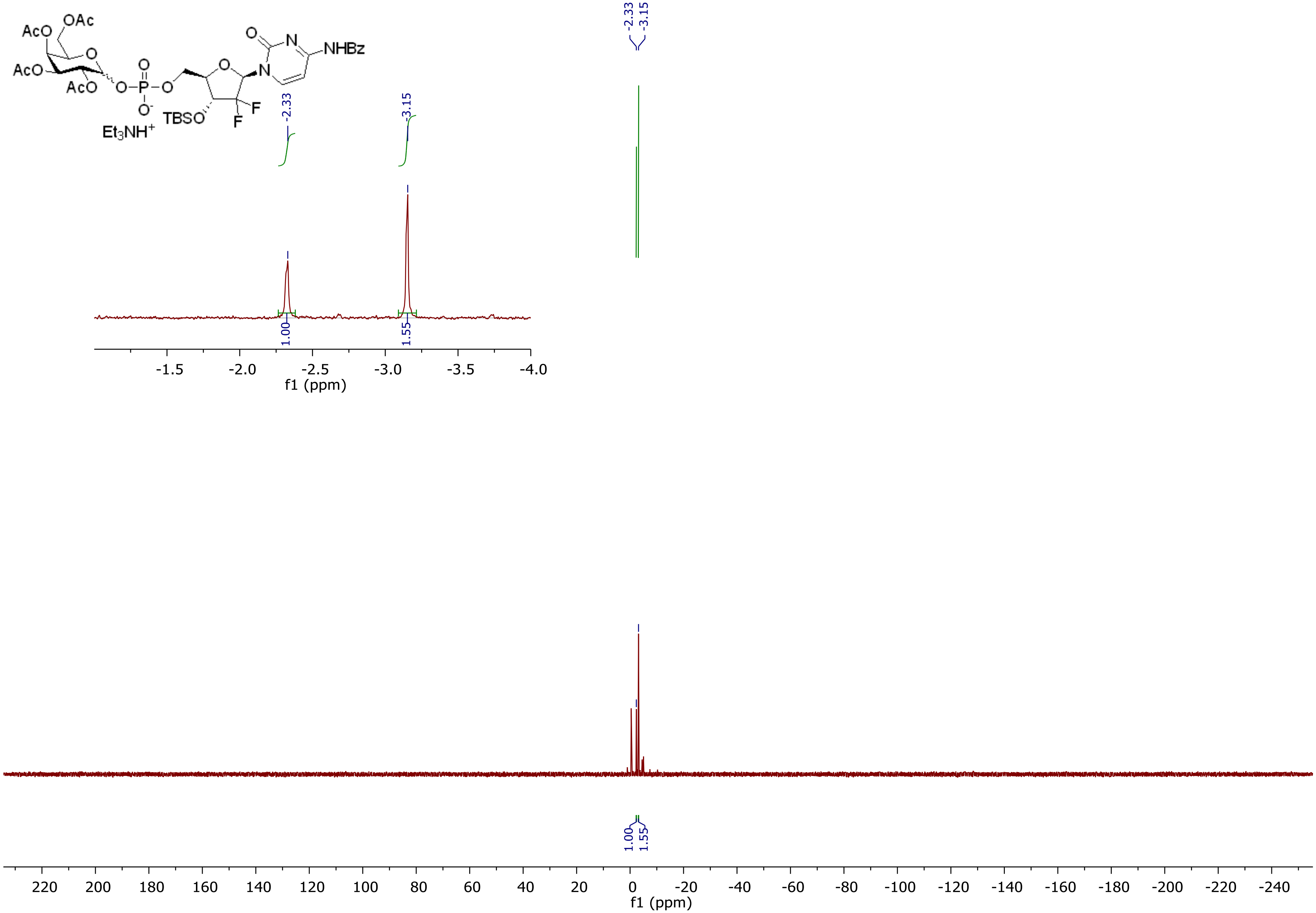

Figure S83 ^{1}H NMR (400 MHz, $CDCl_3$): 2',3'-*O*-*tert*Butyldimethylsilyl-*N*-4-benzoyl-arabinocytidine-5'-*O*-[1''-*O*-(2'',3'',4'',6''-tetra-*O*-acetyl-α/β-D-galactopyranose)]-phosphate triethylammonium salt 24

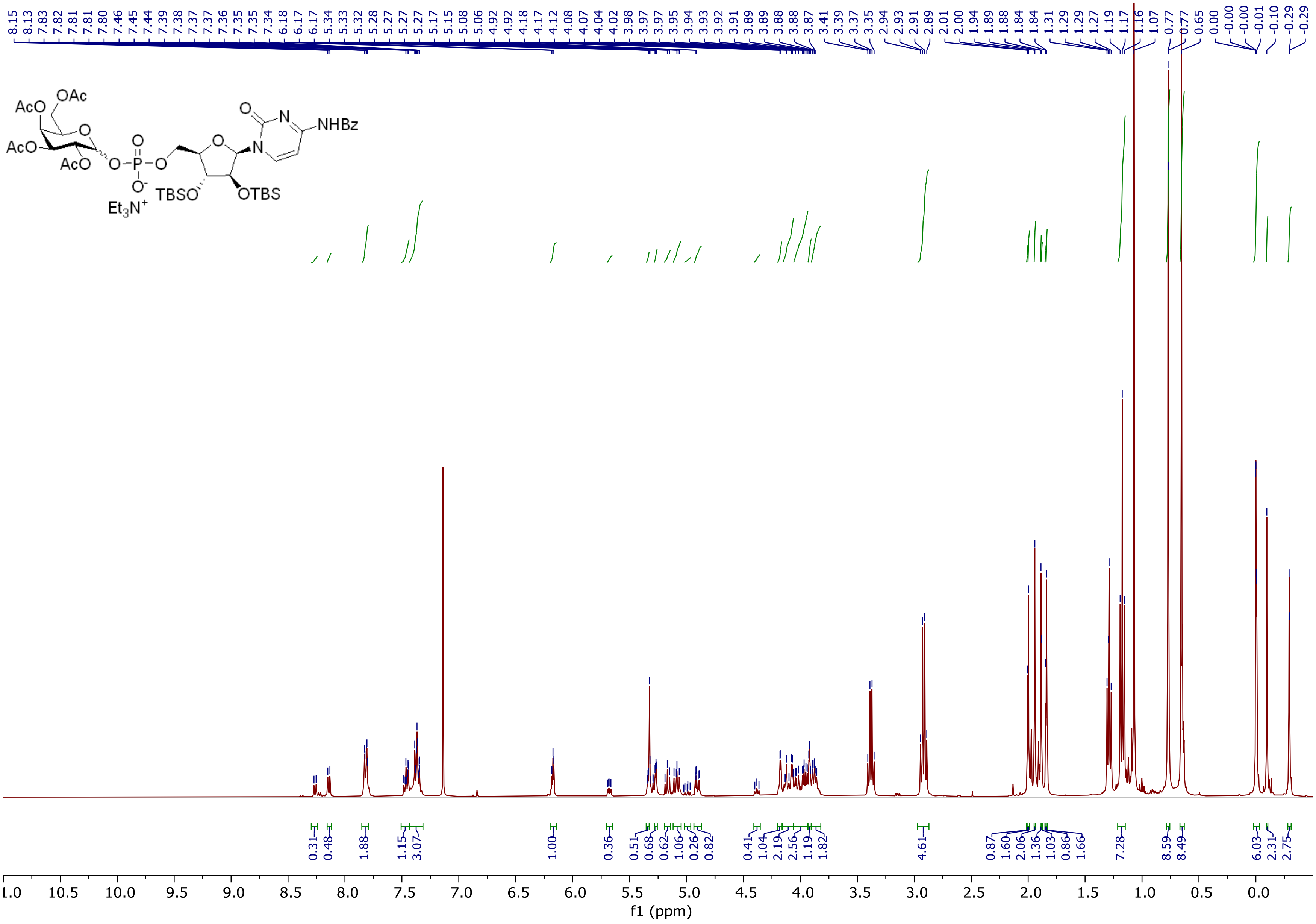

Figure S84 ^{13}C NMR (101 MHz, $CDCl_3$): 2',3'-*O*-*tert*Butyldimethylsilyl-*N*-4-benzoyl-arabinocytidine-5'-*O*-[1''-*O*-(2'',3'',4'',6''-tetra-*O*-acetyl-α/β-D-galactopyranose)]-phosphate triethylammonium salt 24

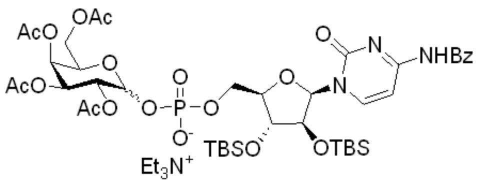

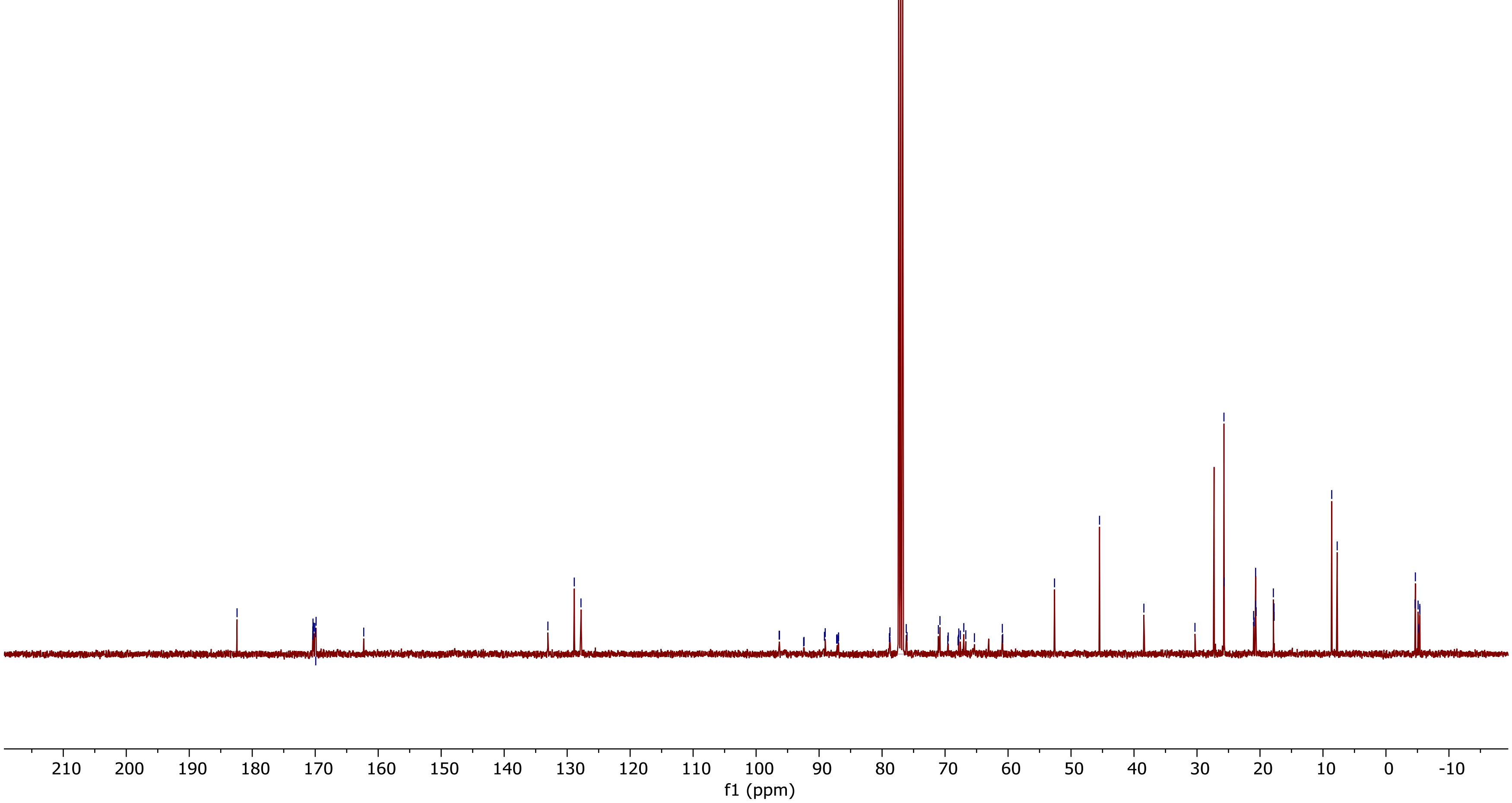

Figure S85 $^{31}P\{^{1}H\}$ NMR (162 MHz, $CDCl_3$): 2',3'-*O*-*tert*Butyldimethylsilyl-*N*-4-benzoyl-arabinocytidine-5'-*O*-[1''-*O*-(2'',3'',4'',6''-tetra-*O*-acetyl-α/β-D-galactopyranose)]-phosphate triethylammonium salt 24

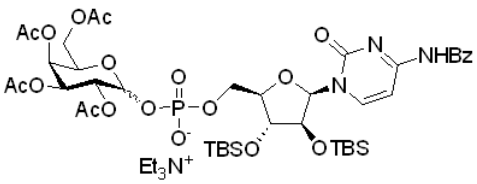

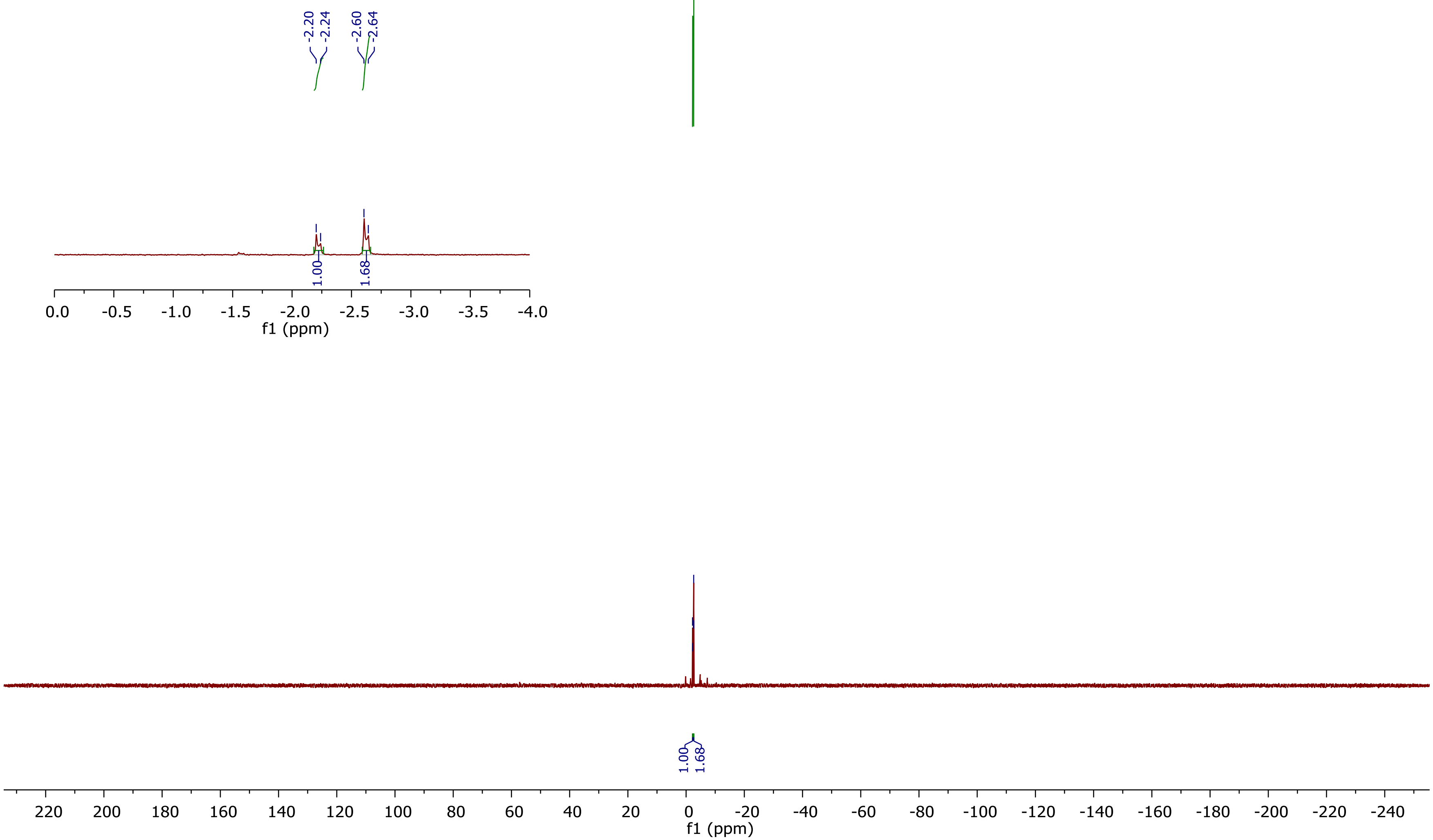

Figure S86 **^{1}H NMR (400 MHz, D_2O): 2’-Deoxy-2’,2’-difluorocytidine-5'-*O*-(1''-*O*-α/β-D-galactopyranose)-phosphate sodium salt**
25

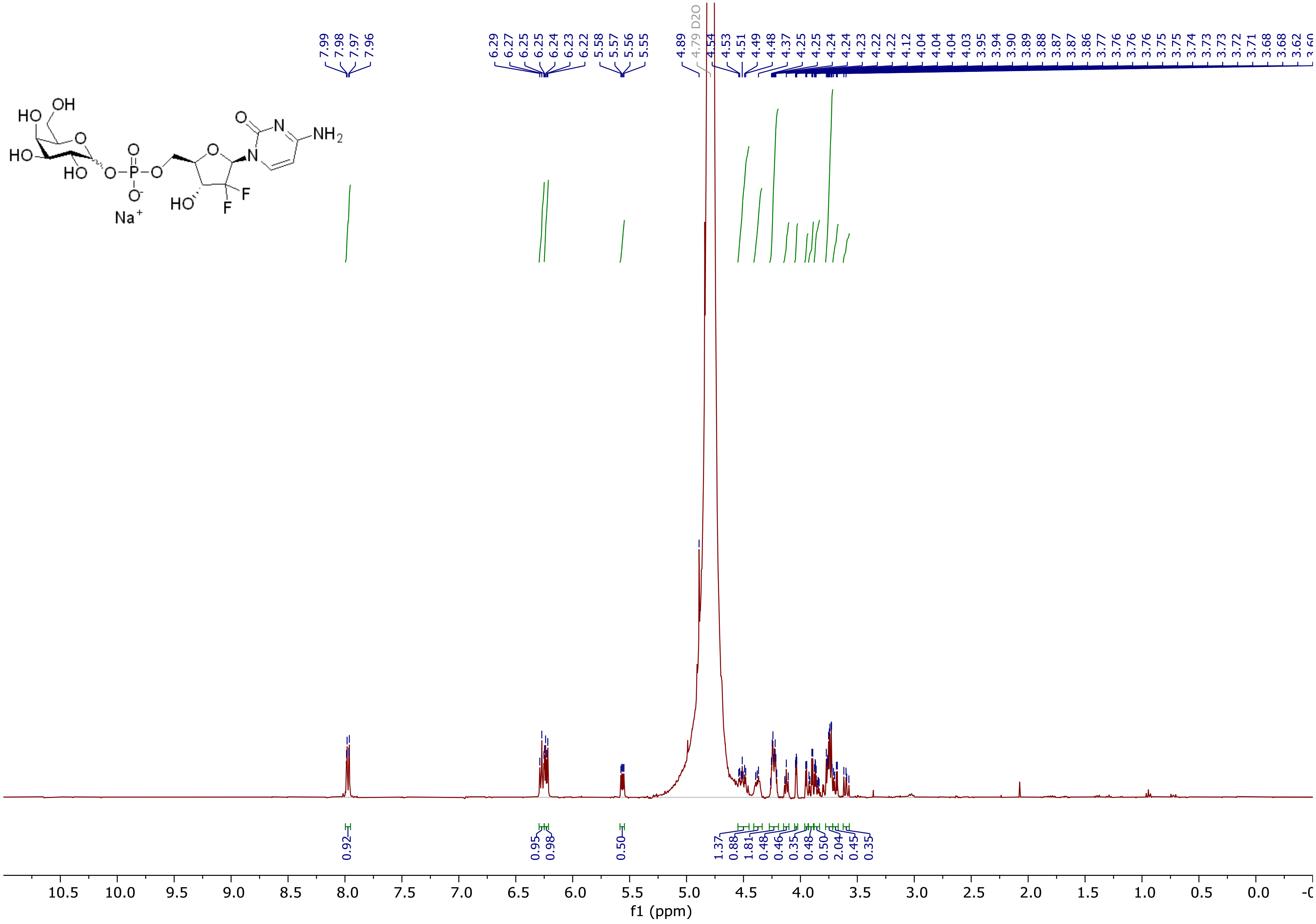

Figure S87 ^{13}C NMR (101 MHz, D_2O): 2’-Deoxy-2’,2’-difluorocytidine-5'-*O*-(1''-*O*-α/β-D-galactopyranose)-phosphate sodium salt 25

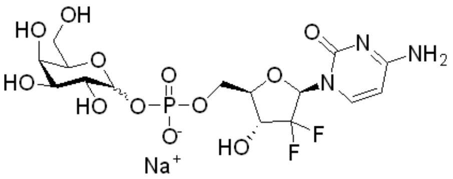

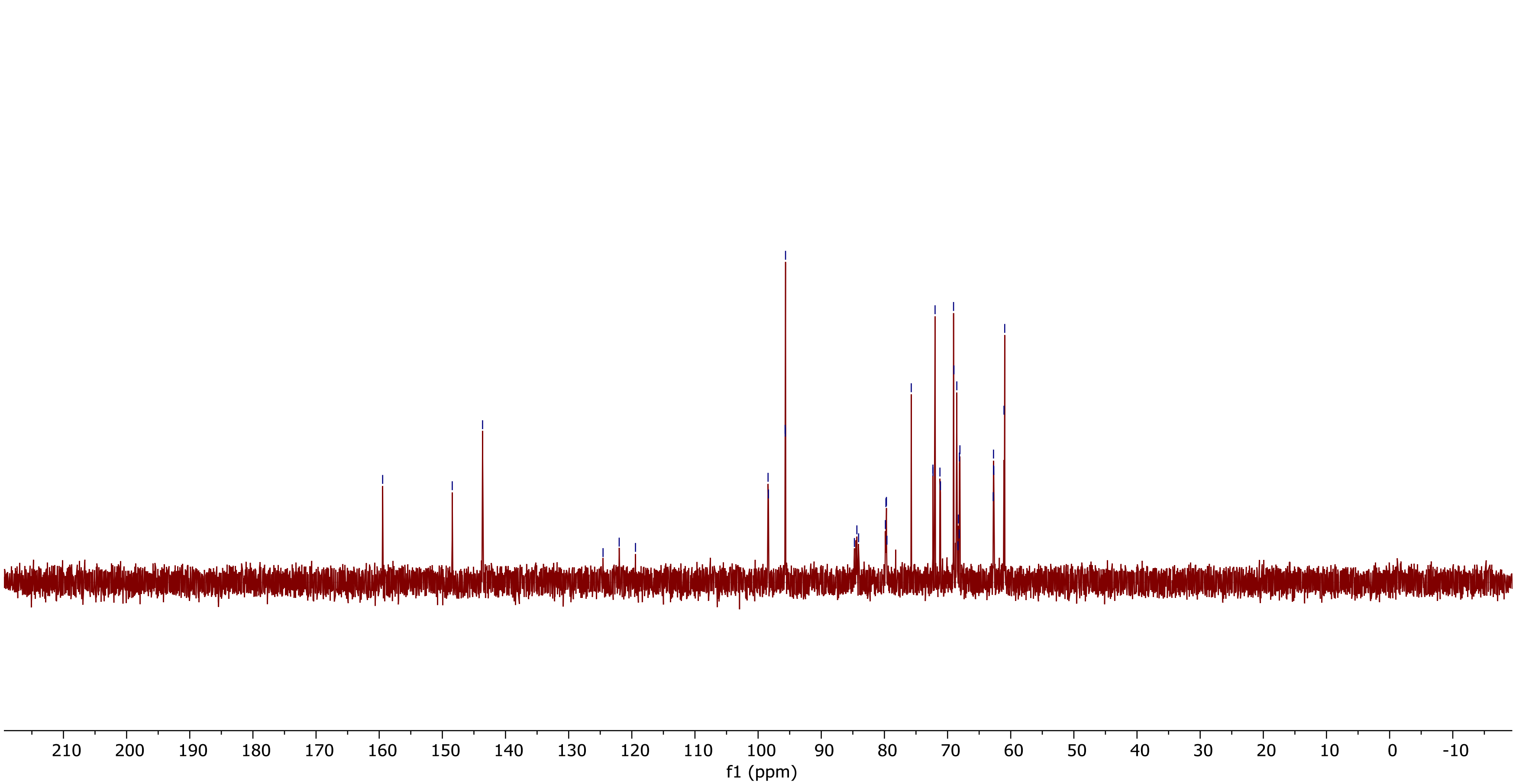

Figure S88 ^{19}F {^{1}H} NMR (377 MHz, D_2O): 2'-Deoxy-2',2'-difluorocytidine-5'-*O*-(1''-*O*-α/β-D-galactopyranose)-phosphate sodium salt 25

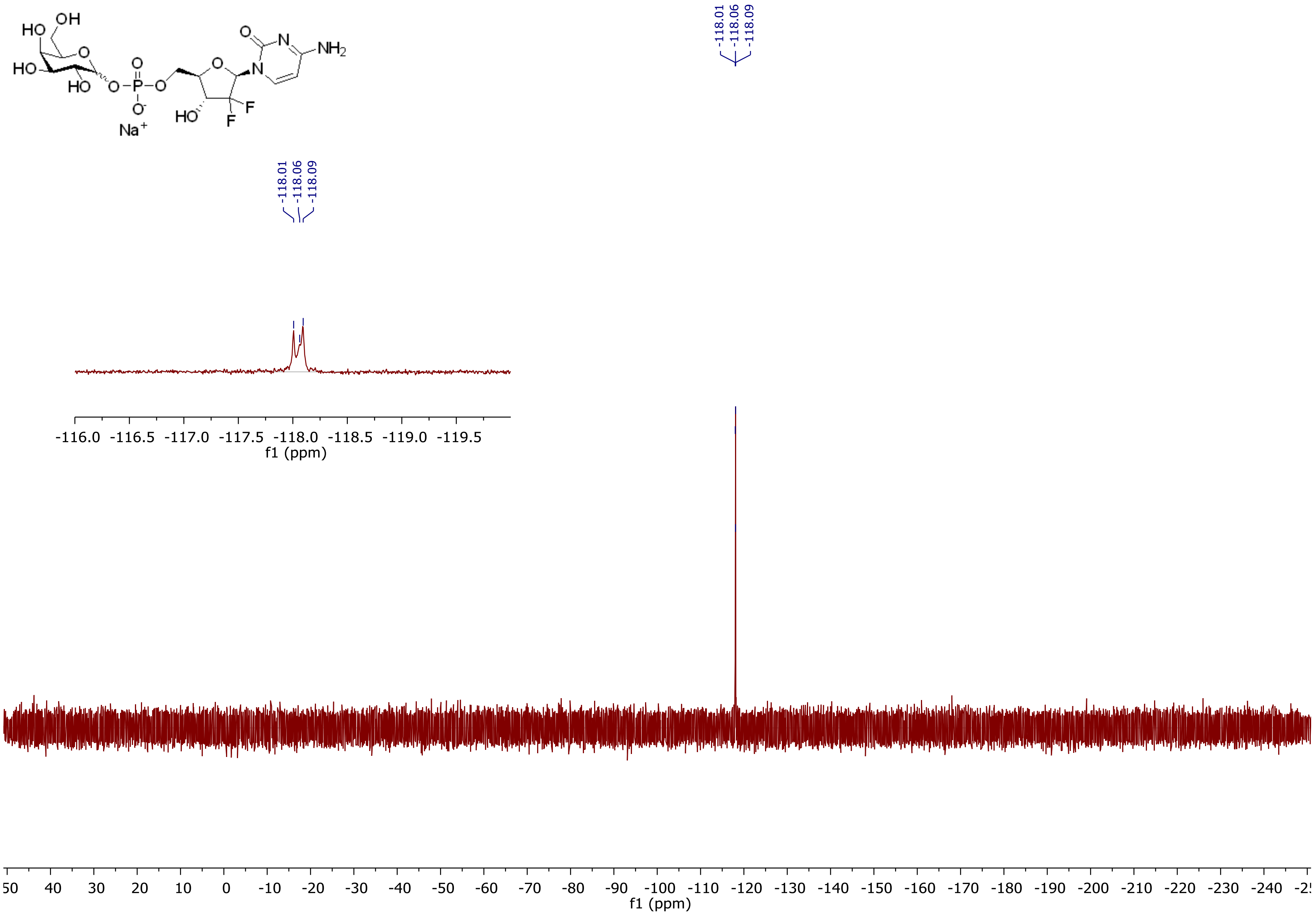

Figure S89 $^{31}P\{^{1}H\}$ NMR (162 MHz, D_2O): 2'-Deoxy-2',2'-difluorocytidine-5'-*O*-(1''-*O*-α/β-D-galactopyranose)-phosphate sodium salt 25

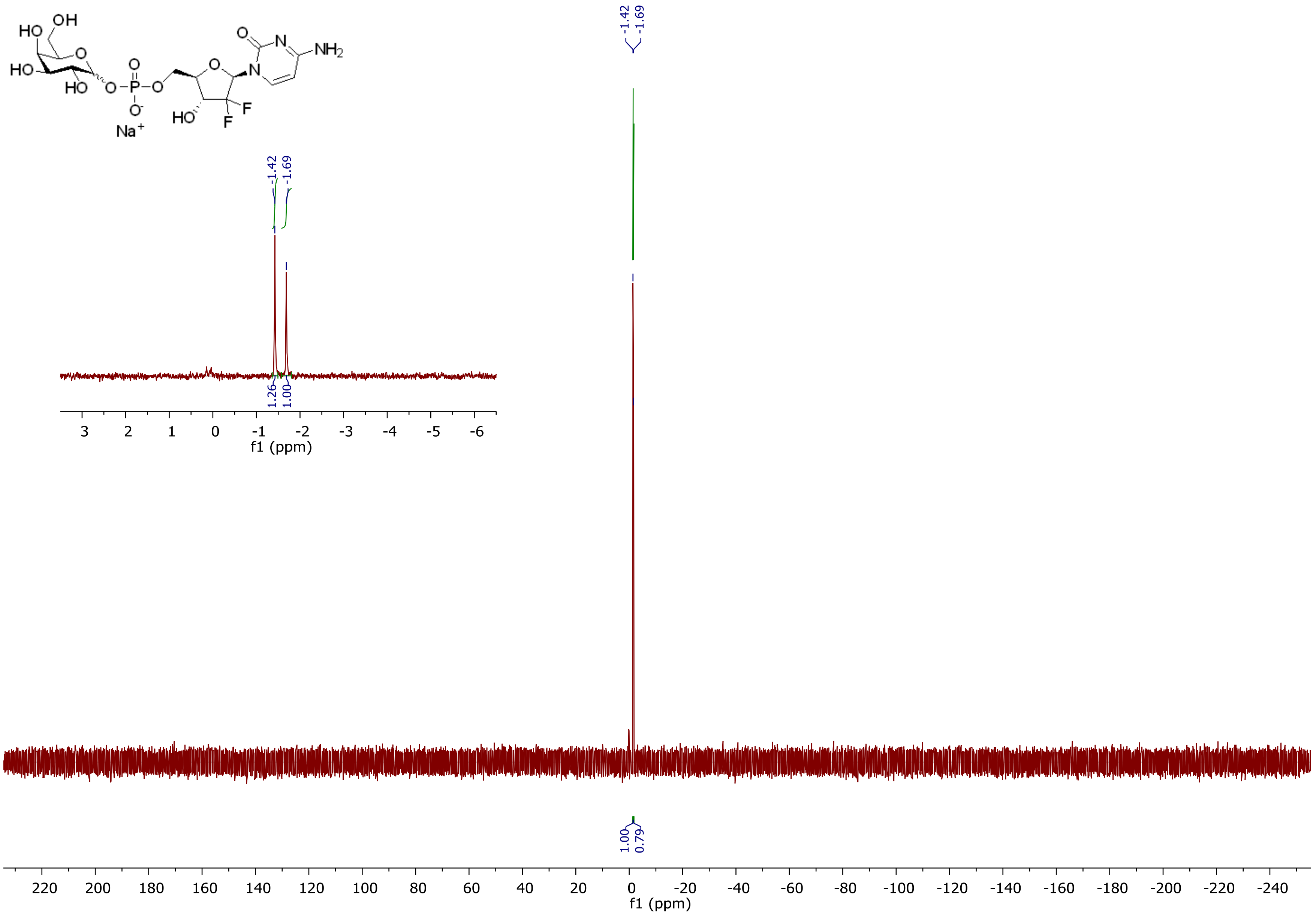

Figure S90 ^{1}H NMR (400 MHz, D_2O): Arabinocytidine-5'-*O*-(1''-*O*-α/β-D-galactopyranose)-phosphate sodium salt 26

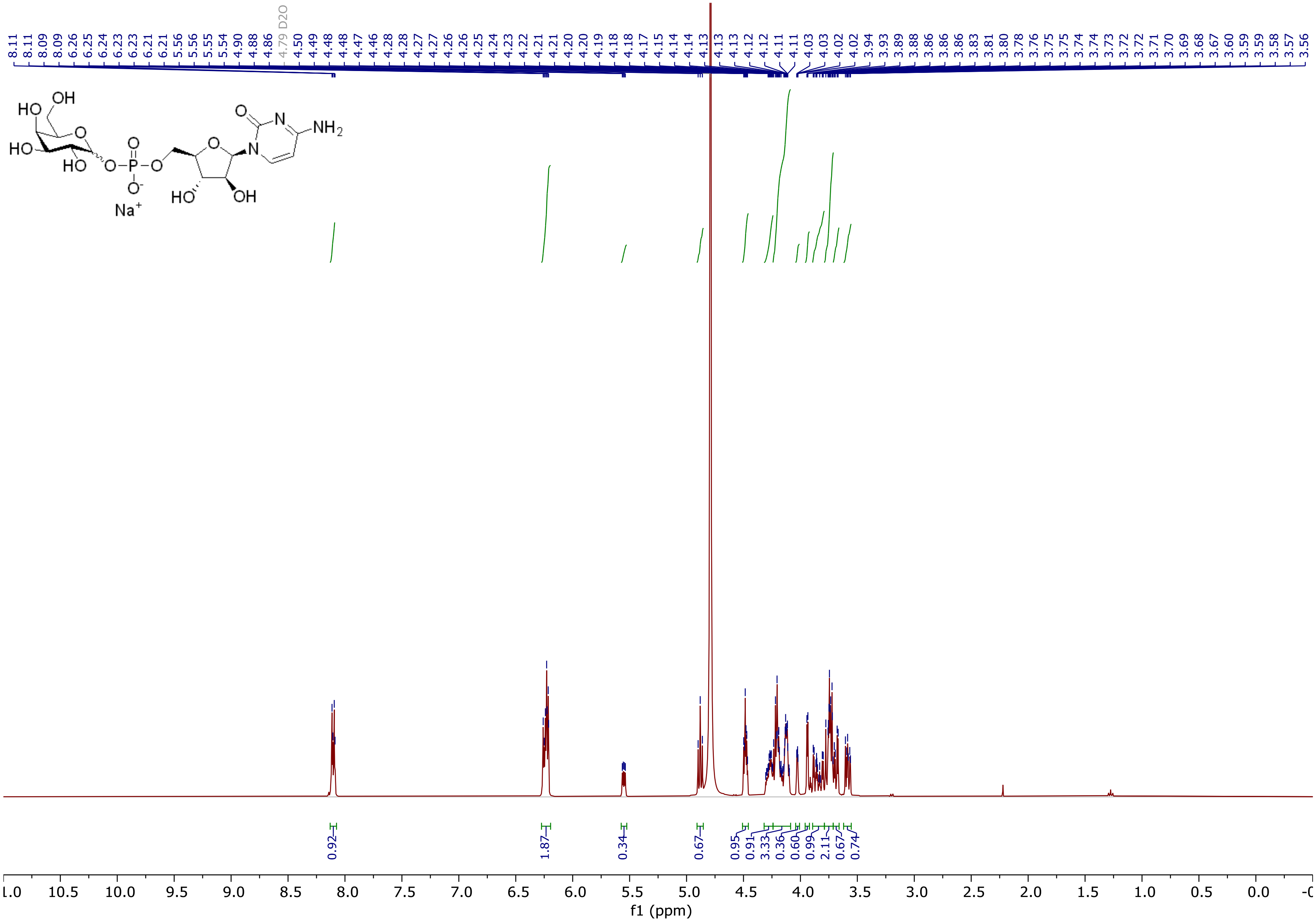

Figure S91 ^{13}C NMR (101 MHz, D_2O): Arabinocytidine-5'-*O*-(1''-*O*-α/β-D-galactopyranose)-phosphate sodium salt 26

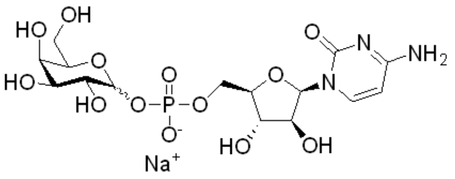

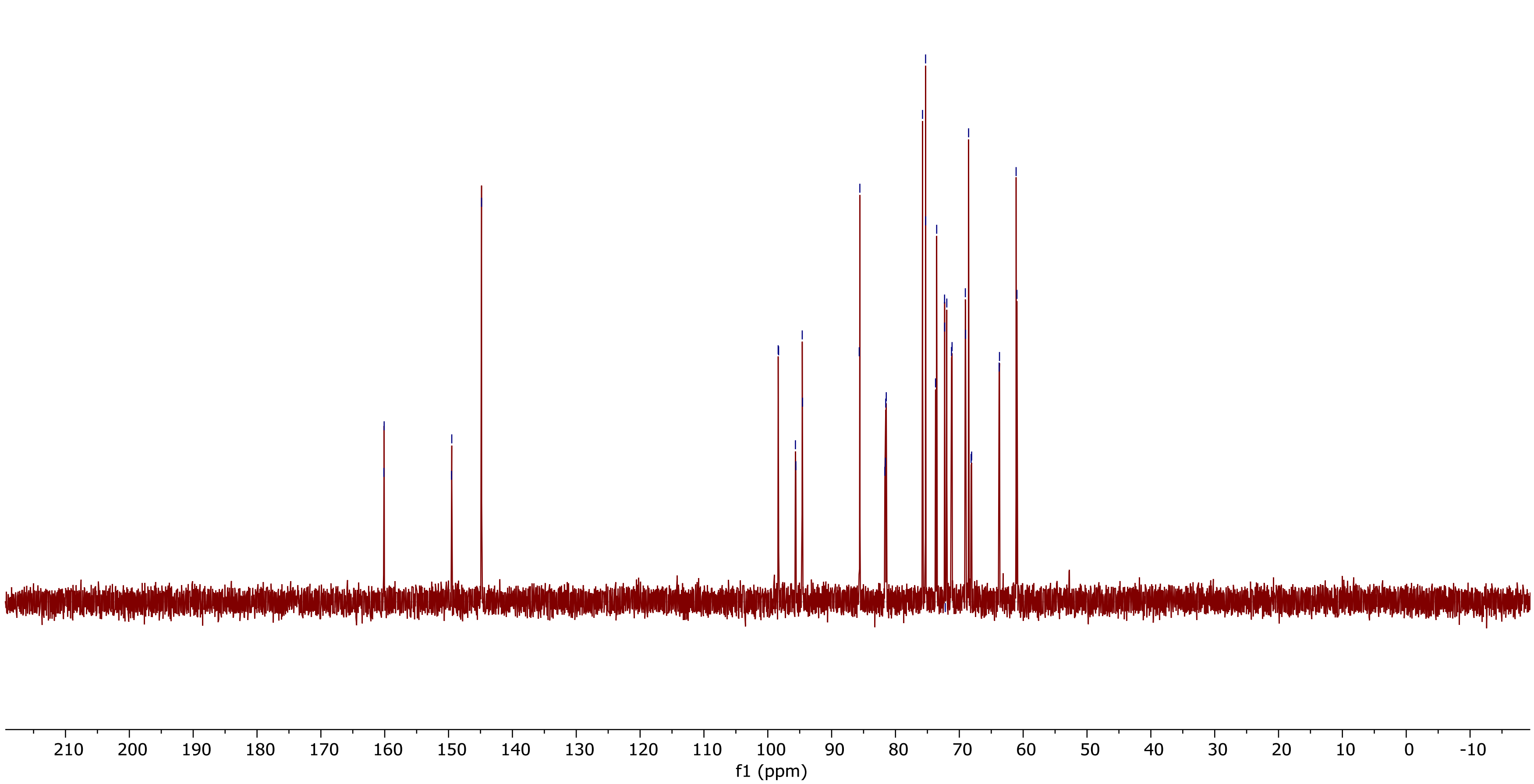

Figure S92 ^{31}P {^{1}H} NMR (400 MHz, D_2O): Arabinocytidine-5'-*O*-(1''-*O*-α/β-D-galactopyranose)-phosphate sodium salt 26

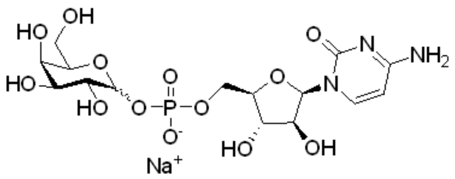

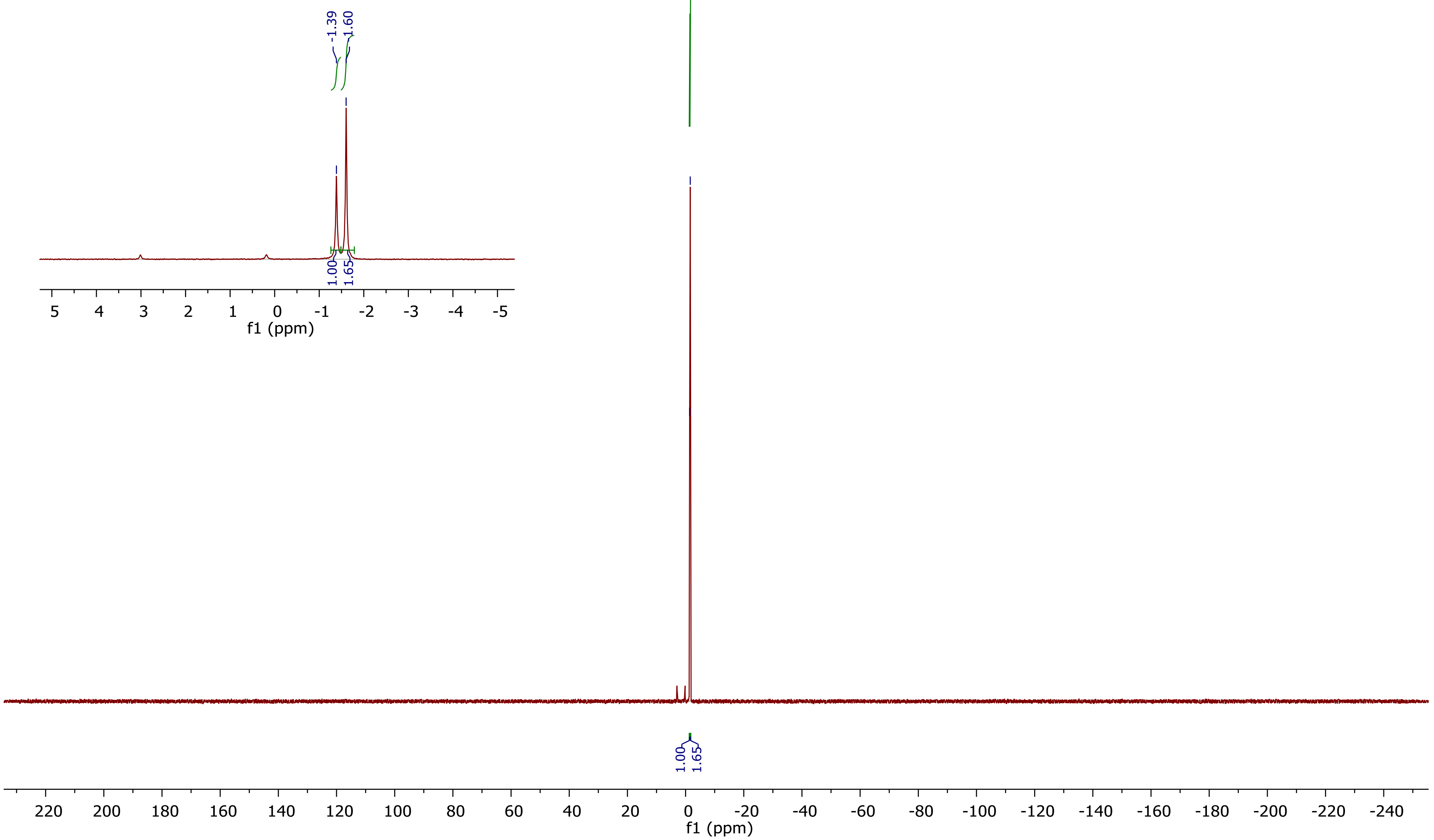

Figure S93 Analytical HPLC traces of Arabinocytidine-5'-*O*-(6''-*O*-α/β-D-glucopyranose)-phosphate sodium salt 8

Area Percent Report
TGU-Glu6Cy

Data file: C:\Users\Public\Documents\ChemStation\3\Data\Thibault\HILIC phosphate 2025-04-24 17-47-42\001-41-TGU-Glu6Cy.D
Sample name: TGU-Glu6Cy
Description:
Injection volume: 1.000 **Sample type:** Sample
Instrument: HPLC 1 UV MS
Injection date: 2025-04-24 17:51:01+01:00

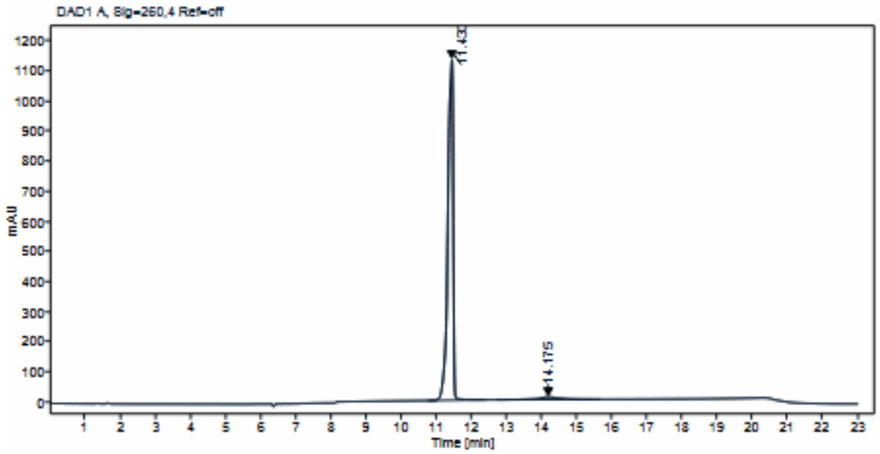

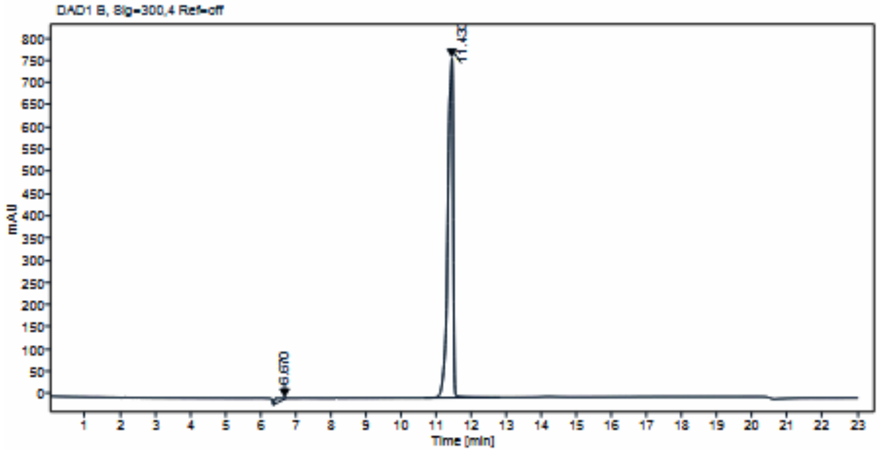

Signal: DAD1 A, Sig=260,4 Ref=off

RT [min]	Type	Width [min]	Area	Height	Area%	Name
11.433	BB	0.1403	11865.5820	1131.5858	97.1341	
14.175	BB	0.5786	350.0856	8.6060	2.8659	

Nucleotide Final Compound report.rdl [Rev. 4] Printed: 2025-05-13 13:36:21+01:00 Page 1 of 2

Figure S94 Analytical HPLC traces of 2'-Deoxy-2',2'-difluorocytidine-5'-*O*-(6''-*O*-β-D-glucopyranose)-phosphate sodium salt 9

Area Percent Report
TGU-Glu6Gem

**Data file:**	C:\Users\Public\Documents\ChemStation\3\Data\Thibault\HILIC phosphate 2024-11-12 17-08-05\006-25-TGU-Glu6Gem.D		
**Sample name:**	TGU-Glu6Gem		
**Description:**			
**Sample amount:**	0.000	**Sample type:**	Sample
**Instrument:**	HPLC 1 UV MS	**Location:**	25
**Injection date:**	2024-11-12 19:13:58+00:00	**Injection:**	1 of 1
**Acq. method:**	HILIC Gem.M	**Injection volume:**	10.000
**Analysis method:**	HILIC Gem.M	**Acq. operator:**	SYSTEM
**Last changed:**	2024-11-19 15:42:26+00:00 (modified after loading)		

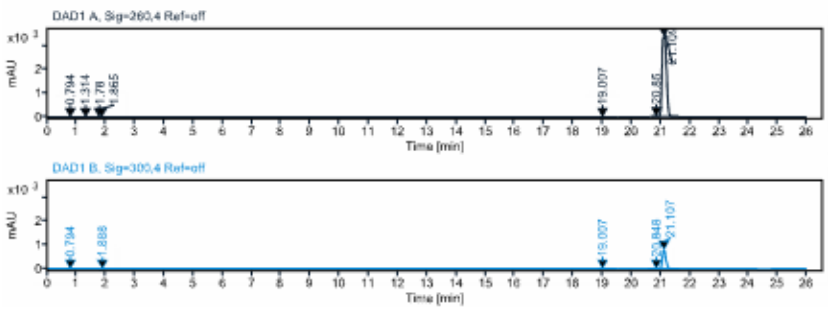

Signal: DAD1 A, Sig=260,4 Ref=off

RT [min]	Type	Width [min]	Area	Height	Area%	Name
0.794	BB	0.0632	6.4462	1.5287	0.0177	
1.314	BB	0.0781	8.2033	1.5410	0.0225	
1.780	BB	0.0467	12.6099	4.4927	0.0346	
1.865	BB	0.0639	8.4772	1.8362	0.0232	
19.007	BB	0.1077	27.8363	3.8822	0.0763	
20.850	BB	0.1179	129.5873	15.4452	0.3553	
21.109	BB	0.1733	36275.1250	3378.3450	99.4703	
		Sum	36468.2851			

Signal: DAD1 B, Sig=300,4 Ref=off

RT [min]	Type	Width [min]	Area	Height	Area%	Name
0.794	BB	0.0634	5.5052	1.2989	0.0803	
1.888	BB	0.0921	21.8118	3.7370	0.3182	
19.007	BB	0.1097	19.0043	2.5873	0.2772	
20.848	BB	0.1297	22.1450	2.3557	0.3231	

Nucleotide Final Compound report.rdl [Rev. 1] Printed: 2024-11-19 15:42:38+00:00 Page 1 of 2

Area Percent Report
TGU-Glu6Gem

RT [min]	Type	Width [min]	Area	Height	Area%	Name
21.107	BB	0.1304	6786.2847	804.9161	99.0012	
		Sum	6854.7510			

Figure S95 Analytical HPLC traces of Arabinocytidine-5'-*O*-(1''-*O*-α/β-D-glucopyranose)-phosphate sodium salt 14

Area Percent Report

TGU-Glu1Cy

**Data file:**	C:\Users\Public\Documents\ChemStation\3\Data\Thibault\HILIC phosphate 2024-11-22 14-29-33\001-23-TGU-Glu1Cy.D		
**Sample name:**	TGU-Glu1Cy		
**Description:**			
**Sample amount:**	0.000	**Sample type:**	Sample
**Instrument:**	HPLC 1 UV MS	**Location:**	23
**Injection date:**	2024-11-22 14:31:38+00:00	**Injection:**	1 of 1
**Acq. method:**	HILIC cyta.M	**Injection volume:**	2.000
**Analysis method:**	HILIC cyta.M	**Acq. operator:**	SYSTEM
**Last changed:**	2024-11-25 11:04:44+00:00 (modified after loading)		

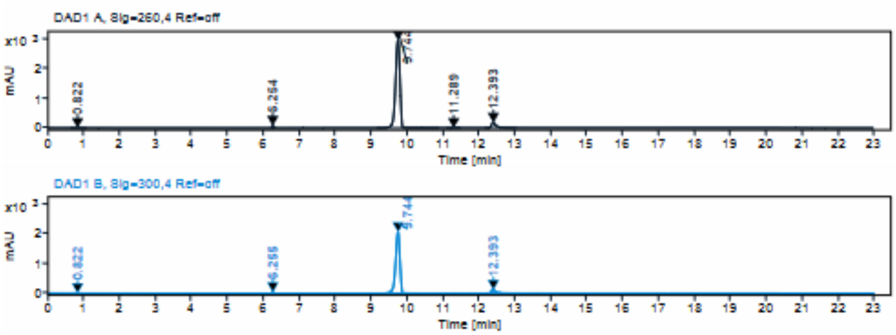

Signal: DAD1 A, Sig=260,4 Ref=off

RT [min]	Type	Width [min]	Area	Height	Area%	Name
0.822	BB	0.0527	17.6322	5.0483	0.0635	
6.254	BB	0.0409	201.8155	81.5857	0.7274	
9.744	BB	0.1354	25908.4219	2981.0474	93.3778	
11.289	BB	0.0976	12.1247	1.8239	0.0437	
12.393	BB	0.1266	1605.8245	182.6275	5.7876	
		Sum	27745.8188			

Signal: DAD1 B, Sig=300,4 Ref=off

RT [min]	Type	Width [min]	Area	Height	Area%	Name
0.822	BB	0.0572	15.5361	4.0115	0.0829	
6.255	BB	0.0406	106.9361	43.6771	0.5707	
9.744	BB	0.1283	17528.0703	2080.0125	93.5505	
12.393	BB	0.1294	1085.9388	120.2533	5.7959	
		Sum	18736.4813			

Figure S96 Analytical HPLC traces of 2’-Deoxy-2’,2’-difluorocytidine-5'-*O*-(1''-*O*-α/β-D-glucopyranose)-phosphate sodium salt 15

Area Percent Report

TGU-Glu1Gem-A

**Data file:**	C:\Users\Public\Documents\ChemStation\3\Data\Thibault\HILIC phosphate 2024-11-22 14-29-33\005-29-TGU-Glu1Gem-A.D		
**Sample name:**	TGU-Glu1Gem-A		
**Description:**			
**Sample amount:**	0.000	**Sample type:**	Sample
**Instrument:**	HPLC 1 UV MS	**Location:**	29
**Injection date:**	2024-11-22 16:22:06+00:00	**Injection:**	1 of 1
**Acq. method:**	HILIC Gem.M	**Injection volume:**	2.000
**Analysis method:**	HILIC Gem.M	**Acq. operator:**	SYSTEM
**Last changed:**	2024-11-25 11:06:04+00:00 (modified after loading)		

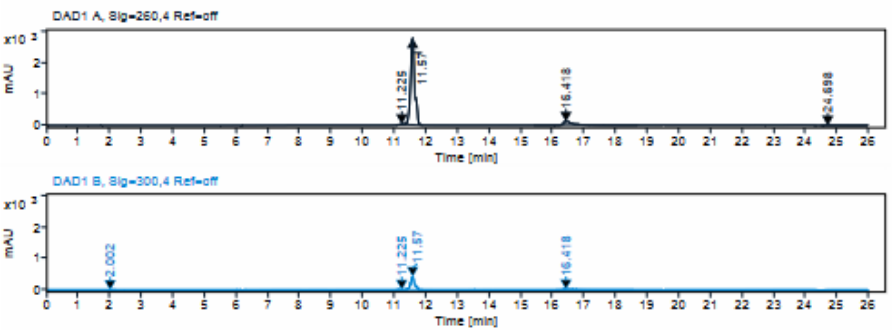

Signal: DAD1 A, Sig=260,4 Ref=off

RT [min]	Type	Width [min]	Area	Height	Area%	Name
11.225	BB	0.1066	171.1475	24.1739	0.6044	
11.570	BB	0.1343	25319.7422	2779.4465	89.4128	
16.418	BB	0.3091	2714.9673	123.1986	9.5875	
24.698	BBA	0.4680	111.9370	2.9954	0.3953	
		Sum	28317.7939			

Signal: DAD1 B, Sig=300,4 Ref=off

RT [min]	Type	Width [min]	Area	Height	Area%	Name
2.002	BB	0.1328	12.1094	1.4299	0.2884	
11.225	BB	0.1075	27.1446	3.7947	0.6466	
11.570	BB	0.1301	3763.8020	421.9116	89.6549	
16.418	BB	0.3061	395.0453	17.9984	9.4101	
		Sum	4198.1013			

Figure S97 Analytical HPLC traces of 2’-Deoxy-2’,2’-difluorocytidine-5'-*O*-(6''-*O*-α/β-D-galactopyranose)-phosphate sodium salt 20

Area Percent Report
TGU-Gal6Gem

Data file: C:\Users\Public\Documents\ChemStation\3\Data\Thibault\HILIC phosphate 2025-05-20 15-30-41\001-92-TGU-Gal6Gem.D

Sample name: TGU-Gal6Gem

Description:

Injection volume: 2.000 **Sample type:** Sample

Instrument: HPLC 1 UV MS

Injection date: 2025-05-20 15:34:09+01:00

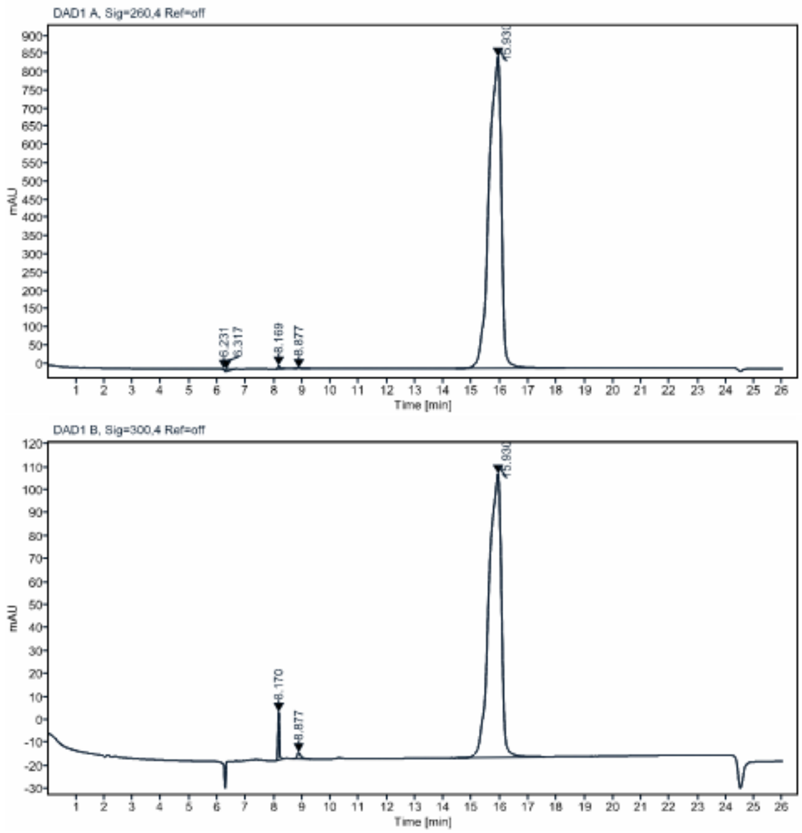

Signal: DAD1 A, Sig=260,4 Ref=off

RT [min]	Type	Width [min]	Area	Height	Area%	Name
6.231	BB	0.0406	6.9195	2.8383	0.0267	
6.317	BB	0.1107	56.9427	6.4709	0.2194	
8.169	BB	0.0620	39.0583	9.4936	0.1505	
8.877	BB	0.1245	12.8383	1.4906	0.0495	
15.930	BB	0.4006	25841.6543	855.1500	99.5540	
		Sum	25957.4131			

Signal: DAD1 B, Sig=300,4 Ref=off

RT [min]	Type	Width [min]	Area	Height	Area%	Name
8.170	BB	0.0564	76.2743	21.0050	1.9681	
8.877	BB	0.1283	24.9143	2.8425	0.6429	
15.930	BB	0.4060	3774.2568	123.6781	97.3890	
		Sum	3875.4454			

Figure S98 Analytical HPLC traces of 2’-Deoxy-2’,2’-difluorocytidine-5'-*O*-(1''-*O*-α/β-D-galactopyranose)-phosphate sodium salt 25

Area Percent Report

TGU-Gal1Gem

Agilent

Data file: C:\Users\Public\Documents\ChemStation\3\Data\Thibault\HILIC phosphate 2025-04-24 16-15-00\003-43-TGU-Gal1Gem.D

Sample name: TGU-Gal1Gem

Description:

Injection volume: 2.000 **Sample type:** Sample

Instrument: HPLC 1 UV MS

Injection date: 2025-04-24 17:11:56+01:00

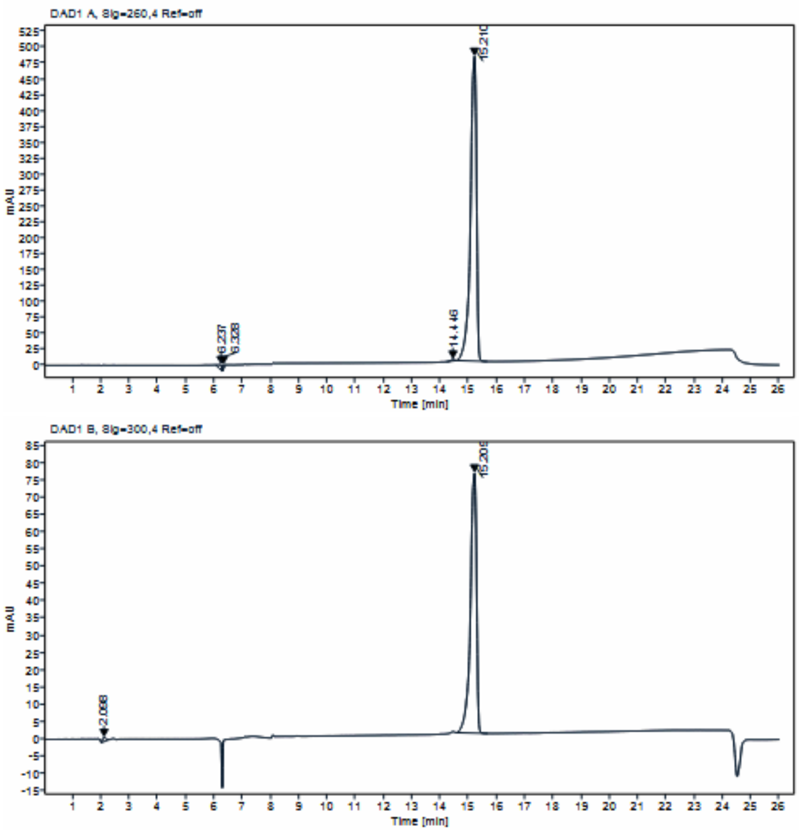

Signal: DAD1 A, Sig=260,4 Ref=off

RT [min]	Type	Width [min]	Area	Height	Area%	Name
6.237	BB	0.0916	60.4517	8.6422	0.8305	
6.328	BB	0.0559	25.9091	6.5898	0.3559	

Area Percent Report

TGU-Gal1Gem

RT [min]	Type	Width [min]	Area	Height	Area%	Name
14.446	BB	0.1227	18.9768	2.3887	0.2607	
15.210	BB	0.2327	7173.6426	479.0385	98.5529	
		Sum	7278.9802			

Signal: DAD1 B, Sig=300,4 Ref=off

RT [min]	Type	Width [min]	Area	Height	Area%	Name
2.098	BB	0.1308	10.0267	1.1845	0.8737	
15.209	BB	0.2363	1137.6188	75.2715	99.1263	
		Sum	1147.6455			

Figure S99 Analytical HPLC traces of Arabinocytidine-5'-*O*-(1''-*O*-α/β-D-galactopyranose)-phosphate sodium salt 26

Area Percent Report
TGU-Gal1Cy
Agilent

**Data file:**	C:\Users\Public\Documents\ChemStation\3\Data\Thibault\HILIC phosphate 2024-11-22 09-04-18\001-24-TGU-Gal1Cy.D		
**Sample name:**	TGU-Gal1Cy		
**Description:**			
**Sample amount:**	0.000	**Sample type:**	Sample
**Instrument:**	HPLC 1 UV MS	**Location:**	24
**Injection date:**	2024-11-22 09:05:16+00:00	**Injection:**	1 of 1
**Acq. method:**	HILIC cyta.M	**Injection volume:**	2.000
**Analysis method:**	HILIC cyta.M	**Acq. operator:**	SYSTEM
**Last changed:**	2024-11-22 10:08:56+00:00 (modified after loading)		

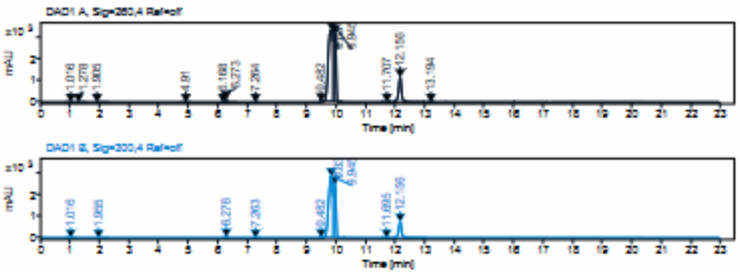

Signal: DAD1 A, Sig=260,4 Ref=off

RT [min]	Type	Width [min]	Area	Height	Area%	Name
1.016	BB	0.0555	27.9523	7.8602	0.0446	
1.278	BB	0.0971	7.8792	1.1616	0.0126	
1.905	BB	0.0790	9.3147	1.6714	0.0149	
4.910	BB	0.1491	12.3872	1.3252	0.0198	
6.168	BB	0.0650	6.0055	1.2729	0.0096	
6.273	BB	0.0423	342.2580	131.8111	0.5461	
7.264	BB	0.0520	7.0502	2.1619	0.0112	
9.482	BB	0.0699	83.9699	19.6497	0.1340	
9.831	MF	0.1824	36410.8281	3327.4604	58.0926	
9.945	FM	0.0917	17452.9531	3172.0593	27.8458	
11.707	BB	0.1568	50.7840	5.0707	0.0810	
12.156	BB	0.1067	8244.7813	1163.2773	13.1543	
13.194	BB	0.1234	21.0792	2.6893	0.0336	
		Sum	62677.2427			

Area Percent Report
TGU-Gal1Cy
Agilent

Signal: DAD1 B, Sig=300,4 Ref=off

RT [min]	Type	Width [min]	Area	Height	Area%	Name
1.016	BB	0.0559	22.4577	6.2479	0.0484	
1.955	BB	0.1706	15.9301	1.3804	0.0343	
6.276	BB	0.0430	130.0597	48.9571	0.2801	
7.263	BB	0.0567	19.9943	5.4643	0.0431	
9.482	BB	0.0697	59.2852	13.9050	0.1277	
9.830	MF	0.1643	28081.3047	2848.6643	60.4740	
9.945	FM	0.0858	12673.9922	2461.9656	27.2938	
11.695	BB	0.1537	27.7958	2.8533	0.0599	
12.156	BB	0.1077	5404.5322	753.6843	11.6388	
		Sum	46435.3520			
